# A Network Meta-Analysis to Compare Effectiveness of Baricitinib and Other Treatments in Rheumatoid Arthritis Patients with Inadequate Response to Methotrexate

**DOI:** 10.36469/jheor.2020.12273

**Published:** 2020-04-10

**Authors:** Walid Fakhouri, Xiaofei Wang, Inmaculada de La Torre, Claudia Nicolay

**Affiliations:** 1 Eli Lilly & Company, Windlesham, Surrey, United Kingdom; 2 Eli Lilly and Company, Indianapolis, IN, USA; 3 Lilly Deutschland GmbH, Bad Homburg, Germany

**Keywords:** rheumatoid arthritis, baricitinib, biologics, JAK 1/2 inhibitor, network meta-analysis, efficacy

## Abstract

**Background/Objectives:**

This article compares the effectiveness of baricitinib (BARI) 4 mg (oral, Janus kinase [JAK] 1/2 inhibitor) versus other targeted synthetic/biologic disease-modifying antirheumatic drugs, in combination with methotrexate (MTX), in moderate-to-severe rheumatoid arthritis patients with inadequate response (IR) to MTX.

**Methods:**

A systematic literature review was conducted to identify randomized controlled trials (RCTs) of the interventions of interest. Bayesian network meta-analyses (NMA) were used to compare American College of Rheumatology (ACR) responses at 24 weeks. A series of prespecified sensitivity analyses addressed the potential impact of, among others, baseline risk, treatment effect modifiers, and trial design on treatment response.

**Results:**

Nineteen RCTs were included in the NMA (primary analysis). For ACR20, BARI 4 mg + MTX was found to be more effective than adalimumab (ADA) 40 mg + MTX (Odds Ratio [OR] 1.33), abatacept (ABA) 10 mg + MTX (IV/4 weeks) (OR 1.45), infliximab (IFX) 3 mg + MTX (IV/8 wks) (OR 1.63), and rituximab (RTX) 1000 mg + MTX (OR 1.63). No differences were found on ACR50. For ACR70, BARI 4 mg + MTX was more effective than ADA 40 mg + MTX (OR 1.37), ABA 10 mg + MTX (OR 1.86), and RTX 1000 mg + MTX (OR 2.26). Sensitivity analysis including 10 additional RCTs with up to 20% of patients with prior biologic use showed BARI 4 mg + MTX to be more effective than tocilizumab (TCZ) 8 mg + MTX on ACR20 (OR 1.44). Results for all sensitivity analyses were consistent with the direction and magnitude of the primary results. Key limitations include the time span in which trials were conducted (1999–2017), during which patient characteristics and treatment approaches might have changed.

**Conclusion:**

This NMA suggests that BARI 4 mg + MTX is an efficacious treatment option in the MTX-IR population as evidenced by the robustness of results.

## INTRODUCTION

Rheumatoid arthritis (RA) is characterized by chronic systemic inflammation primarily affecting diarthrodial joints, resulting in disability and reduced quality of life as well as significant disease burden.^1^ Some recent studies have estimated the prevalence of RA in adults to be approximately 1.36 million in the United States (2014), 2.3 million in Europe (2017), and 1.24 million in Japan (2011).^2^–^4^

Currently, several treatment choices are available for RA including analgesics, nonsteroidal anti-inflammatory drugs (NSAIDs), steroids, conventional synthetic disease-modifying antirheumatic drugs (csDMARDs), biological DMARDs (bDMARDs) (such as adalimumab [ADA], certolizumab [CZP], etanercept [ETN], golimumab [GOL], infliximab [IFX], and tocilizumab [TCZ]), and targeted synthetic disease-modifying antirheumatic drugs (tsDMARDs).^5^,^6^

As per the recent update of the European League Against Rheumatism (EULAR) RA management recommendations, methotrexate (MTX) (rapid escalation to 25 mg/wk) plus short-term glucocorticoids is recommended as the first strategy, aiming at >50% improvement within 3—and target attainment within 6—months.^7^ In the absence of poor prognostic factors, switching or including another csDMARDs (plus short-term glucocorticoids) is suggested.^7^ Any bDMARD or JAK inhibitor should be added to the csDMARD in the presence of poor prognostic factors. If this fails, any other bDMARD or tsDMARD is recommended.^7^

Since MTX is recommended as first-line therapy in RA in both the American College of Rheumatology (ACR) and EULAR guidelines,^7^,^8^ comparative efficacy analyses of biologics and traditional DMARDs in MTX-inadequate response (IR) patients might provide additional insights regarding the management of RA.^9^ Recent EULAR recommendations position JAK inhibitors as equal to bDMARDs; bDMARDs are no longer the preferred option for csDMARDs-IR patients who failed MTX, though the combination with MTX is preferred.^7^ Despite the progress achieved in the treatment of RA there are still unmet needs, as well as a need for improved application of currently available treatments—including better treatment options employing novel mechanisms of actions targeting new pathways.^6^ Recently, significant efforts have been undertaken in order to understand RA pathogenesis, including the role of JAKs in RA pathology.^10^,^11^

Reliable evidence on the comparative efficacy of the biologic antirheumatic agents is crucial for informing clinical and economic decisions about their optimal use. Only a few head-to-head trials of these therapies are available in the population of interest (eg, RA-BEAM^12^ showing the superiority of baricitinib (BARI) against adalimumab; ORAL STRATEGY,^13^ not showing noninferiority against adalimumab, both in the MTX-IR population). The objective of this network meta-analysis (NMA) was to evaluate the effectiveness of BARI 4 mg (oral, JAK 1/2 inhibitor) combined with MTX compared to other targeted synthetic/biologic DMARDs combination therapy with MTX, in moderate-to-severe RA MTX-IR patients. The aim is to provide evidence that will better inform health care providers’ decisions on patients’ treatment.

## METHODS

### Methods of Trial Selection, Quality Assessment, and Appraisal

Prior to the NMA, a systematic literature review (SLR) of randomized controlled trials (RCTs) of targeted synthetic/biologic DMARDs (abatacept [ABA], ADA, CZP, ETN, GOL, IFX, rituximab (RTX), TCZ, tofacitinib [TOFA], sarilumab [SARI], MTX, csDMARDs and BARI) in adult (≥18 years) moderate-to-severe RA MTX-IR patients was conducted (see Tables S1–S3 for population, intervention, comparator, outcomes and study type criteria used to identify studies). At the time the SLR was conducted, upadacitinib had not yet been given market authorization as a treatment for RA patients, and hence was not included. The SLR searches were conducted between 1999 and 2017, in MEDLINE, MEDLINE In-Process, EMBASE, Biosciences Information Service, the Cochrane Library, and Cochrane and European Union’s trials registers. Abstracts from EULAR, ACR, and the British Society for Rheumatology meetings were searched from 2013 to 2018. Only English-language publications were included, and no geographical restrictions were applied. Two reviewers screened the abstracts and full-text articles, while the data were extracted by one reviewer and validated by a second independent reviewer. Any uncertainties were resolved by a third reviewer. The search strategy and list of search terms can be found in the Supplementary Material methods (Tables S4–S7). The Preferred Reporting Items for Systematic Reviews and Meta-Analyses (PRISMA) guidelines were used in development and reporting^14^ and quality assessment of trials was performed to standards recommended^15^ by the Centre for Reviews and Dissemination.^16^ A summary of the quality assessment of trials included in the NMA (primary analysis) is provided in the Supplementary Material (Table S8).

### Endpoints and Time Points

Endpoints analyzed were ACR responses, defined by the ACR20, ACR50, and ACR70 response criteria.^17^ The ACR20 is a combined outcome defined as 20% improvement in the number of tender and swollen joints along with a 20% improvement in three of the following five criteria: patient global assessment, physician global assessment, functional ability measure (most frequently the Health Assessment Questionnaire (HAQ)), visual analogue pain scale, and erythrocyte sedimentation rate or C-reactive protein (CRP). ACR50 and ACR70 are defined as improvement levels of 50% and 70%, respectively, on the previously listed criteria.^17^ The time point of interest was 24 weeks, as this was the time point for the primary endpoint measures for newer biologic agents, including sarilumab.^18^ The 24-weeks’ time point was defined as trial visits scheduled at 24 weeks (± 4 weeks); trial visits from Week 20 to Week 28 were included.

### Network Meta-Analyses (NMA)

NMAs were conducted using Bayesian mixed treatment comparisons as described in the National Institute for Health and Care Excellence (NICE) Decision Support Unit (DSU) Technical Support Documents (TSDs).^15^ ACR response was analyzed using logistic regression models with a binomial likelihood distribution. Both fixed-effect and random-effect simultaneous Bayesian models were fitted for the MTX-IR population. Model fit was assessed using the deviance information criterion (DIC) and residual deviance.^19^ The analysis incorporated the use of bDMARDs or tsDMARDs in combination with MTX where this was within license at 6 months, using the licensed doses for RA. For each analysis, a network diagram was drawn including the number of treatment arms contributing to each pairwise direct evidence. All results are presented here as odds ratios (ORs) with 95% credible intervals (CrI) of BARI 4 mg + MTX versus comparators, with ORs >1 indicating a better result for BARI 4 mg + MTX. A full description of the analysis methods and further results are provided in the Supplementary Material (Methodology, Results).

A series of preplanned sensitivity analyses (see the Supplementary Material, Table S10) were conducted—where possible or applicable—to evaluate the different assumptions made for the NMAs as well as the potential influence of treatment effect modifiers. These sensitivity analyses included the removal of trials (those limited to the Asian-Pacific region and/or using a low/previously unknown MTX dose, or open-label trials), addition of trials (up to 20% of prior bDMARDs), models fitting baseline and treatment effect separately, baseline risk adjustment, and frequentist NMAs.

## RESULTS

### Trial Identification

The SLR identified 10 008 articles, of which 962 were deemed to be potentially relevant after title and abstract screening. After full-text screening, 147 trials with 322 publications were included in the review, which encompassed all RA patient populations. Of those, there were 39 trials that met the SLR inclusion criteria for the MTX-IR moderate-to-severe RA population, and which were eligible for the NMA. Of these, 10 trials allowed for up to 20% of patients with prior bDMARD use, who were only eligible for sensitivity analysis (1). Of the remaining 29 trials, 5 were excluded from the primary 24-week analysis because they were either 12-week trials (n = 3, EXXELERATE,^20^ REALISTIC,^21^ and Lan^22^) or were disconnected from the network due to switching or rerandomization prior to the 24-week time point (n = 2, Abe^23^ and CNTO 148^24^). Five additional trials were excluded as either all treatment arms were monotherapy (n = 3, ADACTA,^25^ SATORI,^26^ and MONARCH^27^) or because excluding monotherapy arms only left a single treatment arm (n = 2, ACT-RAY^28^,^29^ and JESMR^30^) (Supplementary Material, Figure S1). Furthermore, for the purpose of this analysis, treatment arms investigating monotherapy or treatment doses that were out of scope were excluded from trials with more than two treatment arms (eg, ORAL SCAN^31^ and ORAL STANDARD^32^ [TOFA 10 mg + MTX]; ORAL STRATEGY^13^ [TOFA 5 mg monotherapy]; Edwards^33^ [RTX 1000 mg monotherapy]; SERENE^34^ [RTX 500 mg + MTX]; and MOBILITY^18^ [SARI 150 mg + MTX]). The only BARI trial that was conducted in the MTX-IR population was RA-BEAM,^12^ in which only the 4 mg dose of BARI was investigated.

For the primary analysis, a total of 19 trials in the MTX-IR population with moderate-to-severe RA were included. Of these, four were conducted in the Asia Pacific region or only allowed for a low dose (<7.5 mg/wk) of MTX. For the sensitivity analysis allowing for trials with up to 20% of patients with prior bDMARD use, an additional 10 trials were eligible, increasing the total number of trials to 29. It is of note that all three TOFA trials (ORAL SCAN,^31^ ORAL STANDARD,^32^ and ORAL STRATEGY^13^) and both TCZ trials (LITHE,^35^ OPTION^36^) allowed for prior bDMARD use. Consequently, TOFA and TCZ could only be included into the NMA via the corresponding sensitivity analysis. With the exception of the ATTRACT^37^ trial (IFX 3 mg + MTX vs Placebo [PBO] + MTX), which only reported results for ACR20, all other trials presented results for all three ACR response rates. The common reference treatment is PBO + MTX. The full list of trials included in the primary NMA and sensitivity analyses, together with the endpoints available for the analysis at 24 weeks in each trial, is shown in the Supplementary Material (Table S9). The ACR response data as used in the analyses are provided in the Supplementary Material (Table S11).

### Demographic Characteristics

Patient characteristics for each trial included in the NMA analysis conducted on the MTX-IR RA population are described in Table 1. In most trials included in the primary analysis, mean duration of disease was 7 to 11 years. Only one trial (AMPLE^38^) reported a mean duration of disease of less than 2 years. As was to be expected, the percentage of males was low (mostly 15 to 25%). Mean age (SD) ranged from 46.7 (12.2) to 57.20 (11.40). Across trials, the majority of patients were rheumatoid factor positive (>80%). The mean number of tender and swollen joints at baseline was influenced by corresponding inclusion criteria. Three trials reported baseline scores using the Clinical Disease Activity Index (CDAI) (mean: 36.0–38.6) and Simplified Disease Activity Index (SDAI) (mean: 21.2–40.3). Most of the trials reported either DAS28–ESR or DAS28–CRP, and mean DAS28–ESR and DAS28–CRP scores ranged from 5.5–6.9 and 5.3–6.4, respectively.

### Primary Analysis

**Figure 1:**
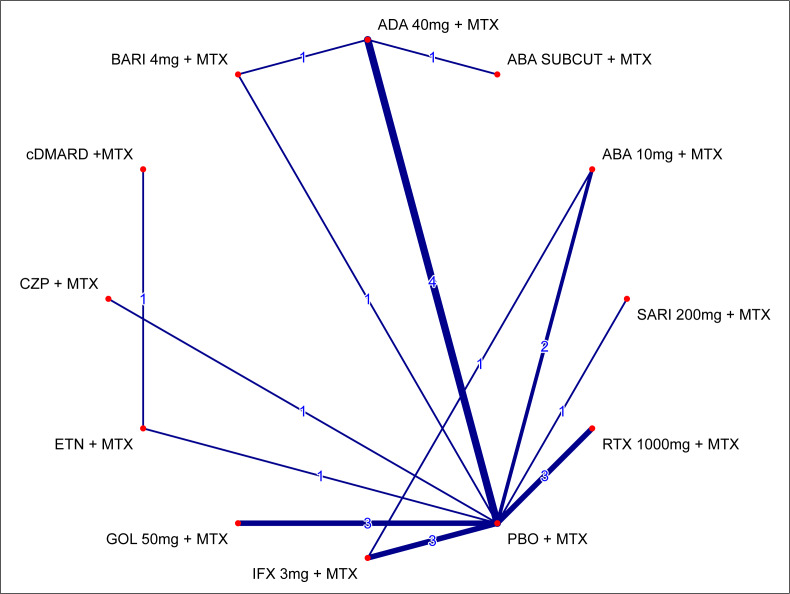
Network of Evidence for ACR20 Response at Week 24 (MTX-IR Population; Primary Analysis) Lines are weighted according to the number of trials comparing the given 2 treatments. The primary analysis includes 19 trials for ACR20 and 18 trials for ACR50/ACR70 (minus ATTRACT [IFX 3 mg + MTX vs PBO + MTX]).

The network of evidence for the ACR20 response for the primary analysis is shown in Figure 1. The only difference between this network and those for ACR50/ACR70 is due to the ATTRACT^37^ trial (IFX 3 mg + MTX vs PBO + MTX), which only reported results for ACR20.

Both fixed- and random-effects models were run, and model fit showed similar results. However, in both models autocorrelation was present and random-effects models showed problems with convergence. Therefore, burn-in and thinning values were increased, and informative instead of noninformative priors were used. These measures considerably improved autocorrelation, but convergence of the random-effects models was still problematic. Therefore, fixed-effects models were chosen as the primary analysis approach and are presented here. As baseline demographics and clinical characteristics indicate some variability between trials (see Table 1), results for the random effects models are also shown (see the Supplementary Material, Tables S15–S17). In addition, Supplementary Material Table S27 provides model fit statistics as per the DIC and the overall residual deviance for the primary analyses and main sensitivity analyses.

**Figure 2:**
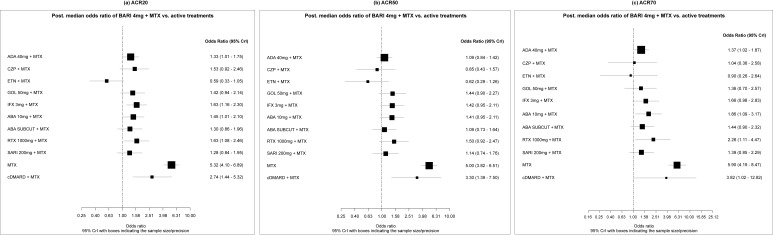
Forest Plots (ACR20/50/70) - (MTX-IR Population; Primary Analysis) Odds ratios (OR) presented refer to BARI 4 mg + MTX vs active treatments. Therefore, ORs >1 are in favor of BARI 4 mg + MTX, whereas ORs <1 are in favor of the comparator. The primary analysis includes 19 trials for ACR20 and 18 trials for ACR50/ACR70.

BARI 4 mg + MTX was found to be statistically significantly more effective than ADA 40 mg + MTX (OR: 1.33; 95% Crl: 1.01–1.75); ABA 10 mg + MTX (OR: 1.45; 95% CrI: 1.01–2.10); IFX 3 mg + MTX (OR: 1.63; 95% CrI: 1.16–2.30); and RTX 1000 mg + MTX (OR: 1.63; 95% CrI: 1.08–2.46) on the ACR20 response. No differences were found between BARI 4 mg + MTX and comparators on the ACR50 response. On the ACR70 response, BARI 4 mg + MTX was found to be statistically significantly more effective than ADA 40 mg + MTX (OR: 1.37; 95% CrI: 1.02–1.87); ABA 10 mg + MTX (OR: 1.86; 95% CrI: 1.09–3.17); and RTX 1000 mg + MTX (OR: 2.26; 95% CrI: 1.11–4.47) (Figure 2). Results for all pairwise comparisons are shown in the Supplementary Material (Tables S12–S14).

### Sensitivity Analyses

#### Baseline Risk Adjustment

Bayesian network meta-regressions adjusting for the variability in PBO response rates across trials were conducted. Scatter plots of PBO response rates (ie, baseline risk) against the treatment effects for each trial showed that increasing PBO responses were associated with a decreasing treatment effect for each of the three outcomes (see the Supplementary Material, Figure S2 [A]-[C]).

**Figure 3:**
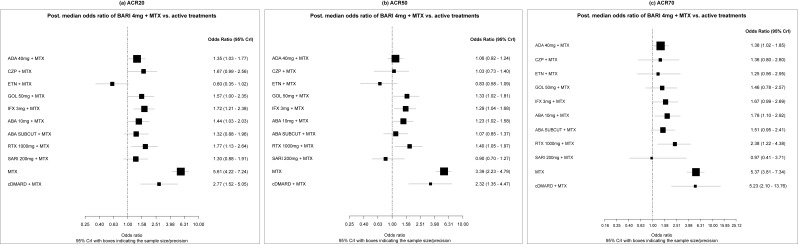
Forest Plots (ACR20/50/70) (MTX-IR Population; Sensitivity Analysis [1]) Odds ratios (ORs) presented refer to BARI 4 mg + MTX vs active treatments. Therefore, ORs >1 are in favor of BARI 4 mg + MTX, whereas ORs <1 are in favor of the comparator. Sensitivity analysis (1): Baseline-risk adjustment (19 trials for ACR20 and 18 trials for ACR50/ACR70).

Some differences were seen in the sensitivity analysis adjusting for baseline risk, where BARI 4 mg + MTX was shown to be statistically significantly more effective compared to a higher number of comparators as found in the primary analysis. This was mainly because of higher point estimates or smaller CrIs (for ACR20, the additional comparator was GOL 50 mg + MTX; for ACR50, additional comparators were GOL 50 mg + MTX, IFX 3 mg + MTX, ABA 10 mg + MTX, and RTX 1000 mg + MTX) (Figure 3).

#### Inclusion of Trials in Which up to 20% of Patients had Prior bDMARD Use

**Figure 4:**
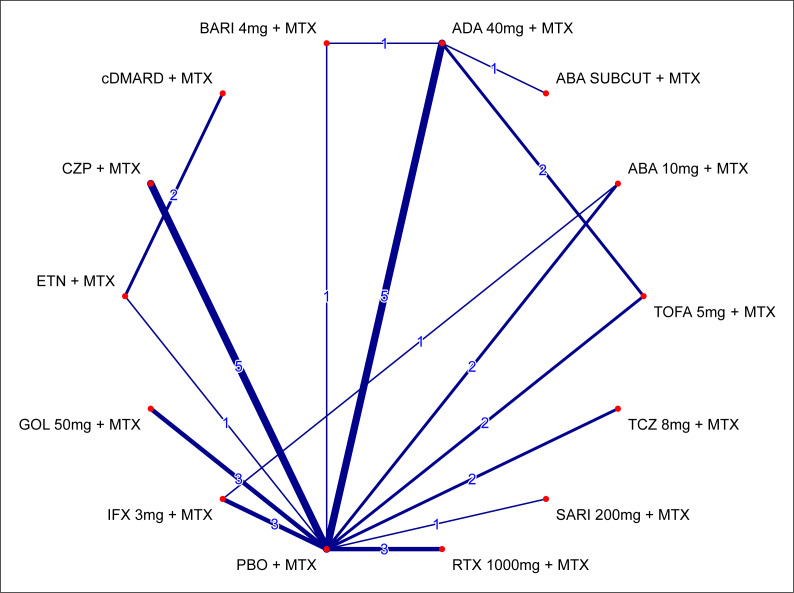
Network of Evidence for ACR20 Response at Week 24 (MTX-IR Population; Sensitivity Analysis [2]) Lines are weighted according to the number of trials comparing the given two treatments. Sensitivity analysis (2): addition of trials allowing for up to 20% of patients with prior bDMARD use (N = 29 trials for ACR20 and N = 28 trials for ACR50/70 (minus ATTRACT [IFX]).

Although up to 20% of patients with prior bDMARDs are often allowed in clinical trials for csDMARDs/MTX-IR populations, those patients might constitute a source of bias, mainly in head-to-head comparisons versus TNFs, as they have previously failed the same mode of action, and responses using a second TNF might be expected to be lower. Consequently, these trials were excluded from the primary analysis, but explored via a prespecified sensitivity analysis that followed the NICE Technology appraisal guidance TA375^57^ and its associated assessment report.^58^ This sensitivity analysis included 10 additional trials with up to 20% of patients with prior bDMARD use (J-RAPID^52^, Kang,^53^ RAPID1,^55^ RAPID2,^56^ LITHE,^35^ OPTION,^36^ ORAL SCAN,^31^ ORAL STANDARD,^32^ ORAL STRATEGY,^13^ and RACAT^54^), and allowed comparisons versus TOFA 5 mg + MTX and TCZ 8 mg + MTX. It is of note that patients in these trials had a shorter mean duration of disease, a lower percentage of being rheumatoid factor positive, and slightly higher mean DAS28-ESR or DAS28-CRP scores at baseline. The network of evidence for the ACR20 response for this sensitivity analysis is presented in Figure 4. As for the primary analysis, the only difference between networks comes from the ATTRACT^37^ trial.

**Figure 5:**
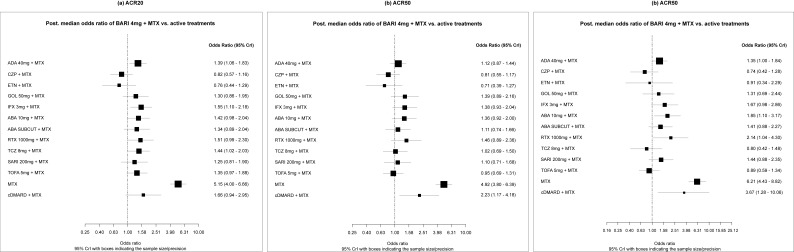
Forest Plots (ACR20/50/70) (MTX-IR Population; Sensitivity Analysis [2]) Odds ratios (ORs)presented refer to BARI 4 mg + MTX vs active treatments. Therefore, ORs >1 are in favor of BARI 4 mg + MTX, whereas ORs < 1 are in favor of the comparator. Sensitivity analysis (2): addition of trials allowing for up to 20% of patients with prior bDMARD use (29 trials for ACR20 and 28 trials for ACR50/ACR70).

No major changes from the primary model were observed for ACR20, ACR50, and ACR70 responses, but for the fact that BARI 4mg + MTX was no longer statistically significantly more effective than ABA 10 mg + MTX for the ACR20 response (OR: 1.42; 95% CrI 0.98–2.04) (Figure 5). This sensitivity analysis allowed the comparisons versus TOFA 5 mg + MTX and TCZ 8 mg + MTX. No statistically significant differences were observed for BARI 4 mg + MTX versus TOFA 5 mg + MTX, whereas BARI 4 mg + MTX was statistically significantly more effective than TCZ 8 mg + MTX on ACR20 (Figure 5). Results for all pairwise comparisons are shown in the Supplementary Material (Tables S21–S23).

#### Trials Conducted in the Asia Pacific Region / Using Low Dose MTX

This sensitivity analysis excluded trials if they were only conducted in Asian-Pacific countries or if the trials had included low (<7.5mg/wk) or unknown MTX doses. It addresses the potential impact on treatment response due to low dose background MTX use, as those patients might not be truly MTX failures. Trials allowing for lower doses of MTX as an inclusion criterion might therefore constitute a source of heterogeneity, and these are predominantly conducted in Asian-Pacific countries. This sensitivity analysis followed the NICE Technology appraisal guidance TA375.^57^ Trials excluded were GO-FORTH,^42^,^59^ Kim,^45^ Li,^60^ and RAPID-C, all conducted in Asian-Pacific countries.^61^ It is of note that patients in these trials reported lower mean numbers of tender joint count (TJC) and swollen joint count (SJC), primarily due to lower corresponding inclusion criteria. As a result, 15 trials were included for ACR20 and 14 trials for ACR50 and ACR70. Specifically, for ADA 40 mg, one out of three trials was excluded,^45^ two out of three trials were excluded for GOL 50 mg (GO-FORTH and Li^49^), and CZP was no longer part of this sensitivity analysis, in addition to TCZ 8 mg and TOFA 5 mg. No changes from the primary model were observed for ACR20, ACR50, and ACR70 responses (see the Supplementary Material, Section 10.3 for more details).

#### Further Sensitivity Analyses

Apart from the sensitivity analyses described previously in greater detail, additional sensitivity analyses (excluding open-label trials, models fitting baseline and treatment effect separately, and frequentist NMAs) were conducted to assess the robustness of the primary models in this MTX-IR population. The sensitivity analysis excluding open-label trials compensated for the potential introduction of bias. However, this only excluded one ETN trial.^47^ Results for all these sensitivity analyses were consistent with the direction and magnitude of the primary results.

To illustrate the different results across the primary and sensitivity analyses, estimated median ACR response rates, plus corresponding 95%-CrIs, are available for each of the treatments (see Supplementary Material Section 12, Figures S4–S16).

While the investigation of inconsistency through node splitting had been preplanned (see the Supplementary Material, Table S10) it was not performed as there were only two closed loops coming from one trial, respectively (ATTEST^41^, RA-BEAM^12^).

## DISCUSSION

The treatment goal in RA is to control inflammation, relieve pain, and reduce disability associated with the condition.^62^ ACR and EULAR guidelines recommend MTX as the first-line therapy for RA. However, many patients do not experience adequate or complete responses with MTX,^8^ creating the need for alternative treatment options in patients who are either refractory or intolerant to MTX. It is important to determine the efficacy of optimal treatment options for these patients; this analysis provides a comprehensive comparison of the efficacy of BARI 4 mg treatment in combination with MTX versus alternative targeted synthetic/biologic DMARDs combination therapy with MTX in moderate-to-severe RA MTX-IR patients. Sarilumab (SARI), which has been approved recently, has also been added to the list of comparators. This NMA followed best practice guidelines, using Bayesian mixed treatment comparisons, allowing simultaneous comparisons of BARI with all treatment options. All available literature was considered. Extensive predefined sensitivity analyses were conducted to test the different assumptions made for the NMAs and to assess the robustness of results.

Primary analyses were conducted for ACR response (20%, 50%, and 70% improvement) at the 24-week time point. As was to be expected, results showed that all active comparators (bDMARDs/tsDMARDs) in combination with MTX were more effective than PBO + MTX. The combination of BARI 4 mg and MTX was either superior or equally effective across all ACR responses, with the ORs favoring BARI in the majority of cases. The results are in line with other meta-analyses conducted to evaluate the efficacy of BARI in RA patients with inadequate response to csDMARDs including MTX.^63^,^64^ Sensitivity analyses showed consistent results with the primary analysis in both direction and magnitude.

It is noteworthy that two of the three ETN trials were against csDMARD + MTX (ie, not the main common comparator PBO + MTX), and one of them was the only open-label trial in this NMA.^47^ Furthermore, the analysis method in this open-label trial used lastobservation carried forward (LOCF) imputation, which differs from the nonresponder imputation used in the other trials. Consequently, ACR response rates in this open-label trial were higher than in any other trials in this NMA. Furthermore, TOFA 5 mg + MTX and TCZ 8 mg + MTX could only be analyzed via the sensitivity analysis allowing for trials with up to 20% of prior bDMARD use. This must be taken into account when interpreting the results.

Potential limitations to this analysis include the time span over which trials were conducted (1999 to 2017), during which patient characteristics and treatment approaches may have changed. As a result, ACR responses might alter over time and result in a “placebo creep.”^65^ Although methods such as the baseline risk adjustment aim to address this problem, it cannot be fully solved by analytical methods alone.

As the analyses focused on the MTX-IR population, the only BARI trial conducted in this population was RA-BEAM,^12^ in which only the 4 mg dose of BARI was investigated. Therefore, no conclusions could be drawn for BARI 2 mg. Of note, the 4 mg dose of BARI constitutes the most commonly approved dose within this population.^66^,^67^

Advantages of NMAs are the ability to combine information from different clinical trials for which direct (head-to-head) evidence may not be available. The focus on the MTX-IR population and therapies in combination with MTX reduced variability. The clinical effectiveness evidence was drawn from a comprehensive and systematic review of RCTs undertaken to assess treatments for RA patients and is, therefore, of general applicability for MTX-IR populations. Results for all sensitivity analyses were consistent with the direction and magnitude of the primary results, providing a robust basis of evidence.

In a very competitive market, BARI, a once-daily oral drug with a rapid onset of action and similar or better comparative effectiveness to other bDMARDs on the market, could be a preferred option for treating RA patients instead of TNF inhibitors. The results of this NMA are supported by the recently published 2019 EULAR guidelines regarding the position of tsDMARDs in the treatment algorithm of RA patients so that no preference is given to bDMARDs over the tsDMARD class.^7^

In conclusion, the results of the NMA suggest that BARI 4 mg + MTX is an efficacious treatment option in the MTX-IR population as evidenced by the robustness of results and differences favoring BARI.

## Supplementary Information



## Figures and Tables

**Table 1:** Population characteristics: MTX-IR

Trial Name	Treatment	Patient Number	Disease Duration (Years) Mean (SD)	Males, %	Age (Years) Mean (SD)	RF Positive, %	Baseline Scores (Mean [SD])						
							HAQ-DI	TJC	SJC	CDAI	SDAI	DAS28-ESR	DAS28-CRP
AIM^39^,^a^	ABA 10 mg + MTX	433	8.5 (7.3)	22.2	51.5 (12.9)	81.8	1.7 (0.7)	31.0 (13.2)	21.4 (8.8)	NR	NR	NR	6.40 (0.08)
	PBO + MTX	219	8.9 (7.1)	18.3	50.4 (12.4)	78.5	1.7 (0.6)	32.3 (13.6)	22.1 (8.8)	NR	NR	NR	6.40 (0.11)
AMPLE^38^	ABA 125 mg + MTX	318	1.9 (1.4)	18.6	51.4 (12.6)	75.5	1.5 (0.7)	25.4 (15.3)	15.8 (9.8)	NR	NR	NR	5.50 (1.10)
	ADA 40 mg + MTX	328	1.7 (1.4)	17.7	51.0 (12.8)	77.4	1.5 (0.7)	26.3 (15.8)	15.9 (10.0)	NR	NR	NR	5.50 (1.10)
ARMADA^40^	ADA 40 mg + MTX	67	12.2 (11.1)	25.4	57.2 (11.4)	NR	1.6 (0.6)	28.0 (12.7)	17.3 (8.6)	NR	NR	NR	NR
	PBO + MTX	62	11.1 (8.0)	17.7	56.0 (10.8)	NR	1.6 (0.6)	28.7 (15.2)	16.9 (9.5)	NR	NR	NR	NR
ATTEST^41^,^a^	ABA 10 mg+ MTX	156^c^	7.9 (8.5)	16.7	49.0 (12.5)	87.2	1.8 (0.6)	31.6 (13.9)	21.3 (8.6)	NR	NR	6.90 (1.00)	NR
	IFX 3 mg + MTX	165^c^	7.3 (6.2)	17.6	49.1 (12.0)	84.8	1.7 (0.7)	31.7 (14.5)	20.3 (8.0)	NR	NR	6.80 (0.90)	NR
	PBO + MTX	110^c^	8.4 (8.6)	12.7	49.4 (11.5)	77.3	1.8 (0.7)	30.3 (11.7)	20.1 (7.0)	NR	NR	6.80 (1.00)	NR
ATTRACT^37^	IFX 3 mg + MTX	86	10.0 (8.0)	19.0	54.0 (11.0)	84.0	1.8 (0.6)	32.0 (18.0)	22.0 (12.0)	NR	NR	NR	NR
	PBO + MTX	88	11.0 (8.0)	20.0	51.0 (12.0)	77.0	1.7 (0.6)	31.0 (18.0)	21.0 (12.0)	NR	NR	NR	NR
Edwards (2004)^33^	RTX 1000 mg + MTX	40	9.0 (6.0)	25.0	54.0 (12.0)	100	2.0 (0.6)	32 (16)	23 (13)	NR	NR	6.80 (0.92)	NR
	PBO + MTX	40	11.0 (7.0)	20.0	54.0 (11.0)	100	2.0 (0.5)	32 (13)	19 (10)	NR	NR	6.90 (0.75)	NR
GO-FORTH^42^,^b^	GOL 50 mg + MTX	89	8.8 (8.8)	15.1	50.4 (9.9)	NR	1.0 (0.61)	13.1 (8.38)	11.8 (6.7)	NR	NR	5.50 (1.18)^e^	NR
	PBO + MTX	90	8.7 (8.2)	17.0	51.1 (11.6)	NR	1.0 (0.7)	13.2 (7.8)	11.4 (6.6)	NR	NR	5.60 (0.99)^e^	NR
GO-FORWARD^43^	GOL 50 mg + MTX	89	4.5 (5.6)	19.1	52.0 (43–57)^d^	86.5	1.38 (1.0–1.9)^d^	26.0 (16–39)^d^	13.0 (8-2)^d^	NR	NR	6.11 (5.37–6.94)^d^	5.10 (4.06–5.65)^d^
	PBO + MTX	133	6.5 (6.5)	18.0	52.0 (42–58)^d^	81.2	1.25 (0.8–1.8)^d^	21.0 (14–34)^d^	12.0 (8–19)^d^	NR	NR	6.1 (5.3–6.6)^d^	4.9 (4.2–5.5)^d^
Keystone (2004)^44^	ADA 40 mg + MTX	207	11.0 (9.2)	23.7	56.1 (13.5)	81.6	1.5 (0.6)	27.30 (12.7)	19.30 (9.8)	NR	NR	NR	NR
	PBO + MTX	200	10.9 (8.8)	27.0	56.1 (12.0)	89.5	1.48 (0.6)	28.1 (13.8)	19.0 (9.5)	NR	NR	NR	NR
Kim (2007)^45^,^a^	ADA 40 mg QOW + MTX	65	6.8 (4.2)	4.6	48.5 (10.2)	76.9	1.4 (0.6)	19.2 (9.20)	12.2 (5.6)	NR	NR	NR	NR
	PBO + MTX	63	6.9 (4.5)	14.3	49.8 (10.5)	82.5	1.30 (0.6)	20.30 (8.6)	12.8 (5.8)	NR	NR	NR	NR
Li (2013)^46^	GOL 50 mg + MTX	132	7.6 (7.1)	16.7	47.7 (11.5)	87.1	1.3 (0.7)	22.9 (15.4)	10.7 (7.0)	NR	NR	NR	5.4 (1.1)
	PBO + MTX	132	8.0 (7.3)	21.2	46.7 (12.2)	92.4	1.2 (0.7)	22.5 (14.8)	11.8 (7.4)	NR	NR	NR	5.5 (1.1)
Machado (2014)^47^	ETN 50 mg + MTX	284	7.9 (7.0)	11.7	48.4 (12.0)	86.1	1.6 (0.7)	25.1 (11.9)	18.2 (8.4)	NR	NR	6.6 (0.7)	NR
	cDMARD + MTX	145	9.0 (7.5)	9.9	48.6 (11.3)	83.8	1.6 (0.7)	26.2 (12.3)	19.3 (10.1)	NR	NR	6.7 (0.7)	NR
MOBILITY^18^	SARI 200 mg + MTX	427	8.6 (7.0)	15.0	50.8 (11.8)	83.0	1.7 (0.6)	26.5 (14.5)	16.8 (9.7)	NR	NR	NR	6.0 (0.9)
	PBO + MTX	428	9.1 (8.1)	19.0	50.9 (11.2)	84.0	1.6 (0.7)	26.8 (13.7)	16.7 (9.3)	NR	NR	NR	5.9 (0.9)
RA-BEAM^12^	BARI 4 mg + MTX	487	10.3 (8.8)	23.0	53.5 (2.0)	90.1	1.6 (0.7)	23.4 (13.0)	15.0 (8.2)	38.1 (12.0)	40.3 (12.7)	6.5 (0.9)	5.8 (0.9)
	ADA 40 mg + MTX	330	9.6 (8.5)	23.9	52.9 (2.0)	91.2	1.6 (0.7)	23.4 (13.7)	15.4 (9.1)	38.0 (13.0)	40.1 (13.4)	6.4 (1.0)	5.8 (0.9)
	PBO + MTX	488	10.4 (8.7)	21.7	53.40 (2.0)	92.4	1.6 (0.7)	23.3 (13.5)	15.5 (9.4)	37.6 (12.8)	39.5 (13.3)	6.4 (1.0)	5.7 (1.0)
RA-SCORE^48^	RTX 1000 mg + MTX	60	4.9 (2.9)	16.7	50.7 (11.7)	NR	1.3 (0.7)	14.0 (6.9)	10.9 (5.9)	NR	NR	6.0 (1.1)	5.3 (1.0)
	PBO + MTX	63	4.4 (3.1)	23.8	50.3 (11.9)	NR	1.5 (0.8)	14.9 (6.7)	11.4 (6.1)	NR	NR	6.3 (1.1)	5.6 (1.1)
RAPID-C^49^	CZP 200 mg + MTX	312	NR	NR	NR	NR	NR	NR	NR	NR	NR	NR	NR
	PBO + MTX	113	NR	NR	NR	NR	NR	NR	NR	NR	NR	NR	NR
SERENE^34^	RTX 1000 mg+ MTX	168	7.1 (7.0)	20.4	51.9 (12.9)	75.4	NR	27.1 (14.1)	18.6 (9.6)	NR	NR	6.5 (1.1)	5.9 (1.0)
	PBO + MTX	172	7.5 (7.6)	14.5	52.2 (12.4)	75.0	NR	30.2 (15.9)	20.9 (11.3)	NR	NR	6.5 (1.0)	6.0 (1.0)
START^50^	IFX 3 mg + MTX	360	7.8 (12.8)^d^	20.0	53.0 (27.7)^d^	82.8	1.5 (1.3)^d^	22.0 (19.9)^d^	15.0 (12.9)^e^	NR	NR	NR	NR
	PBO + MTX	363	8.4 (12.0)^d^	16.8	52.0 (28.4)^d^	80.7	1.5 (1.3)^d^	22.0 (20.9)^d^	20.9 (11.3)^e^	NR	NR	NR	NR
Weinblatt (1999)^51^	ETN 50 mg + MTX	59	13.0 (NR)	10.0	48.0 (NR)	84.0	1.5 (NR)^d^	28.0 (NR)^d^	20.00 (NR)^e^	NR	NR	NR	NR
	PBO + MTX	30	13.0 (NR)	27.0	53.0(NR)	90.0	1.5 (NR)^d^	28.0 (NR)	17.00 (NR)^e^	NR	NR	NR	NR
J-RAPID (Yamamoto [(2011])^52^,^f^	CZP 400–200 mg QOW + MTX	82	5.6 (4.2)	15.9	50.6 (11.4)	86.6	1.1 (0.7)	19.0 (9.0)	16.0 (8.4)	NR	NR	6.2 (0.8)	NR
	PBO + MTX	77	5.8 (4.1)	14.3	51.9 (11.1)	85.7	1.2 (0.7)	19.6 (10.4)	17.4 (10.0)	NR	NR	6.5 (0.9)	NR
Kang (2013)^53^,^f^	CZP 400–200 mg QOW + MTX	81	6.2 (NR)	NR	NR	NR	NR	NR	NR	NR	NR	7.4 (NR)^e^	NR
	PBO + MTX	40	6.2 (NR)	NR	NR	NR	NR	NR	NR	NR	NR	7.4 (NR)^e^	NR
LITHE^35^,^f^	TCZ 8 mg + MTX	398	9.3 (NR)	18.0	53.4 (11.7)	83.00	1.5 (0.6)	29.3 (15.2)	17.3 (9.5)	NR	NR	NR	6.6 (1.0)^e^
	PBO + MTX	393	9.0 (NR)	17.0	51.3 (12.4)	82.00	1.5 (0.6)	27.9 (14.8)	16.6 (9.2)	NR	NR	NR	6.5 (1.0)^e^
OPTION^36^,^f^	TCZ 8 mg + MTX	205	7.5 (7.3)	15.0	50.8 (11.8)	83.00	1.6 (0.6)	31.9 (15.5)	19.5 (11.3)	NR	NR	NR	6.8 (0.9)^e^
	PBO + MTX	204	7.8 (7.2)	22.0	50.6 (12.1)	71.00	1.5 (0.6)	32.8 (16.1)	20.70 (11.7)	NR	NR	NR	6.8 (0.9)^e^
ORAL SCAN^31^,^f^	TOFA 5 mg + MTX	321	8.9 (NR)	16.2	53.7 (11.6)	75.20	1.4 (NR)	24.1 (NR)	14.1 (NR)	NR	NR	6.3 (NR)	5.2 (NR)
	TOFA 10 mg + MTX	316	9.0 (NR)	13.6	52.0 (11.4)	77.6	1.4 (NR)	23.0 (NR)	14.4 (NR)	NR	NR	6.3 (NR)	5.2 (NR)
	PBO + MTX	160	8.8 (NR)	14.4	53.2 (11.5)	77.5	NR	NR	NR	NR	NR	NR	NR
ORAL STANDARD^32^,^f^	TOFA 5 mg + MTX	204	7.6 (NR)	14.7	53.0 (11.9)	66.8	1.5 (0.6)	28.0 (NR)	16.7 (NR)	NR	NR	6.6 (NR)	5.4 (NR)
	ADA 40 mg + MTX	204	8.1 (NR)	20.6	52.5 (11.7)	68.2	1.5 (0.6)	26.7 (NR)	16.4 (NR)	NR	NR	6.4 (NR)	5.3 (NR)
	TOFA 10 mg + MTX	201	7.4 (NR)	16.4	52.9 (11.8)	66.2	1.5 (0.6)	26.1 (NR)	15.8 (NR)	NR	NR	6.5 (NR)	5.4 (NR)
	PBO + MTX	108	NR	24.1	NR	66.7	1.4 (0.7)	NR	NR	NR	NR	NR	NR
ORAL STRATEGY^13^,^f^,^g^	TOFA 5 mg + MTX	386	6.1 (NR)^d^	17.0	49.7 (12.2))	NR	1.6 (0.6)	15.4 (6.5)	11.2 (5.6)	38.6 (12.6)	40.2 (13.0)	6.5 (0.9)	5.7 (0.9)
	ADA 40mg + MTX	388	6.0 (NR)^d^	17.0	50.7 (13.4)	NR	1.6 (0.6)	15.2 (6.7)	11.0 (5.4)	38.2 (12.9)	39.8 (13.3)	6.5 (1.0)	5.7 (01.0)
RACAT^54^,^f^,^g^	ETN 50 mg + MTX	175	4.9 (8.0)	51.4	56.0 (13.2)	66.9	1.5 (0.8)	13.3 (6.4)	11.3 (5.2)	36.4 (11.2)	NR	NR	5.9 (0.9)^e^
	SSZ 1–2 g QD + HCQ 400 mg QD + HCQ + MTX	178	5.5 (9.3)	56.7	57.8 (13.0)	65.7	1.4 (0.8)	13.4 (6.6)	11.1 (5.3)	36.0 (11.5)	NR	NR	5.9 (0.9)^e^
RAPID1^55^,^f^	CZP 400–200 mg QOW + MTX	393	6.1 (4.2)	17.6	51.4 (11.6)	79.6	1.7 (0.6)	30.8 (12.4)	NR	NR	21.7 (9.9)	6.9 (NR)^d^	NR
	PBO + MTX	199	6.2 (4.4)	16.1	52.2 (11.2)	82.8	1.7 (0.6)	29.8 (13.0)	NR	NR	21.2 (9.7)	7.0 (NR)^d^	NR
RAPID2^56^,^f^	CZP 400–200 mg QOW + MTX	246	6.1 (4.1)	16.3	52.2 (11.1)	77.5	1.6 (0.6)	30.1 (14.5)	20.5 (9.6)	NR	NR	6.9 (0.8)	NR
	PBO + MTX	127	5.6 (3.9)	15.7	51.5 (11.8)	78.2	1.6 (0.6)	30.4 (13.4)	21.9 (9.7)	NR	NR	6.8 (0.9)	NR

ABA = abatacept; ADA = adalimumab; AZA = azathioprine; BARI = baricitinib; CDAI = Clinical Disease Activity Index; CRP = C-reactive protein; CZP = certolizumab pegol; DAS = Disease Activity Score; DAS28 = Disease Activity Score modified to include the 28 diarthrodial joint count; ESR = erythrocyte sedimentation rate; ETN = etanercept; GOL = golimumab; HAQ-DI = Health Assessment Questionnaire without Disability Index; HCQ = hydroxychloroquine; hs-CRP = high-sensitivity C-reactive protein; ID = identification; IFX = infliximab; MTX = methotrexate; NR = not reported; PBO = placebo RF = rheumatoid factor; RTX = rituximab; SARI = sarilumab; SD = standard deviation; SDAI = Simplified Disease Activity Index; SJC = Swollen joint count based on 66 joints; SSZ = sulfasalazine; TJC = Total joint count based on 68 joints; TCZ = tocilizumabTOFA = tofacitinib;

a In this study SJC was based on 28 joint counts;

b Japanese patients only; inclusion criteria for TJC and SJC was >=4;

c Number treated; number randomized not reported;

d Median (range) values;

e Does not report if ESR or CRP was used;

f Addition of trials that allowed for up to 20% of patients with prior bDMARD use;

g For this trial TJC was based on how many joint counts were not given; for the Weinblatt trial it was based on 71 joint counts; and for ORAL STRATEGY on 28 joint counts.

## References

[1] Guo Q, Wang Y, Xu D, Nossent J, Pavlos NJ, Xu J (2018). Rheumatoid arthritis: pathological mechanisms and modern pharmacologic therapies. Bone Res.

[2] Hunter TM, Boytsov NN, Zhang X, Schroeder K, Michaud K, Araujo AB (2017). Prevalence of rheumatoid arthritis in the United States adult population in healthcare claims databases, 2004–2014. Rheumatol Int.

[3] O’Hara J, Rose A, Jacob I, Burke T, Walsh S (2017). The burden of rheumatoid arthritis across Europe: a socioeconomic survey (BRASS).

[4] Yamanaka H, Sugiyama N, Inoue E, Taniguchi A, Momohara S (2014). Estimates of the prevalence of and current treatment practices for rheumatoid arthritis in Japan using reimbursement data from health insurance societies and the IORRA cohort (I). Mod Rheumatol.

[5] Smolen JS, Landewe R, Bijlsma J (2017). EULAR recommendations for the management of rheumatoid arthritis with synthetic and biological disease-modifying antirheumatic drugs: 2016 update. Ann Rheum Dis.

[6] Chatzidionysiou K, Emamikia S, Nam J (2017). Efficacy of glucocorticoids, conventional and targeted synthetic disease-modifying antirheumatic drugs: a systematic literature review informing the 2016 update of the EULAR recommendations for the management of rheumatoid arthritis. Ann Rheum Dis.

[7] Smolen JS, Landewe RBM, Bijlsma JWJ (2020). EULAR recommendations for the management of rheumatoid arthritis with synthetic and biological disease-modifying antirheumatic drugs: 2019 update. Ann Rheum Dis.

[8] Singh JA, Furst DE, Bharat A (2012). 2012 update of the 2008 American College of Rheumatology recommendations for the use of disease-modifying antirheumatic drugs and biologic agents in the treatment of rheumatoid arthritis. Arthritis Care Res (Hoboken).

[9] Choi M, Hyun MK, Choi S (2017). Comparative efficacy of biological agents in methotrexate-refractory rheumatoid arthritis patients: a Bayesian mixed treatment comparison. Korean J Intern Med.

[10] Fridman JS, Scherle PA, Collins R (2010). Selective inhibition of JAK1 and JAK2 is efficacious in rodent models of arthritis: preclinical characterization of INCB028050. J Immunol.

[11] Nakayamada S, Kubo S, Iwata S, Tanaka Y (2016). Chemical JAK inhibitors for the treatment of rheumatoid arthritis. Expert Opin Pharmacother.

[12] Taylor PC, Keystone EC, Van Der Heijde D (2017). Baricitinib versus placebo or adalimumab in rheumatoid arthritis. N Engl J Med.

[13] Fleischmann R, Mysler E, Hall S (2017). Efficacy and safety of tofacitinib monotherapy, tofacitinib with methotrexate, and adalimumab with methotrexate in patients with rheumatoid arthritis (ORAL Strategy): a phase 3b/4, double-blind, head-to-head, randomised controlled trial. Lancet.

[14] Moher D, Liberati A, Tetzlaff J, Altman DG (2009). Preferred reporting items for systematic reviews and meta-analyses: the PRISMA statement. PLoS Med.

[15] Excellence NNIfHaC Methods for development of NICE public health guidance.

[16] (2009). Systematic Reviews CRD’s Guidance for Undertaking Reviews in Health Care.

[17] Felson DT, Anderson JJ, Boers M, American College of Rheumatology (1995). Preliminary definition of improvement in rheumatoid arthritis. Arthritis Rheum.

[18] Genovese MC, Fleischmann R, Kivitz AJ (2015). Sarilumab plus methotrexate in patients with active rheumatoid arthritis and inadequate response to methotrexate: results of a phase III Study. Arthritis Rheumatol.

[19] Spiegelhalter DJ, Best NJ, Carlin BP, van der Linde A (2002). Bayesian measures of model complexity and fit. J Statist Soc B.

[20] Smolen JS, Burmester G-R, Combe B (2016). Head-to-head comparison of certolizumab pegol versus adalimumab in rheumatoid arthritis: 2-year efficacy and safety results from the randomised EXXELERATE study. Lancet.

[21] Weinblatt ME, Fleischmann R, Huizinga TWJ (2012). Efficacy and safety of certolizumab pegol in a broad population of patients with active rheumatoid arthritis: results from the REALISTIC phase IIIb study. Rheumatology (Oxford).

[22] Lan J-L, Chou S-J, Chen D-Y, Chen Y-H, Hsieh T-Y, Young M (2004). A comparative study of etanercept plus methotrexate and methotrexate alone in Taiwanese patients with active rheumatoid arthritis: a 12-week, double-blind, randomized, placebo-controlled study. J Formos Med Assoc.

[23] Abe T, Takeuchi T, Miyasaka N (2006). A multicenter, double-blind, randomized, placebo controlled trial of infliximab combined with low dose methotrexate in Japanese patients with rheumatoid arthritis. J Rheumatol.

[24] Kay J, Matteson EL, Dasgupta B (2008). Golimumab in patients with active rheumatoid arthritis despite treatment with methotrexate: a randomized, double-blind, placebo-controlled, dose-ranging study. Arthritis Rheum.

[25] Taylor DM, Cornelius V, Smith L, Young AH (2014). Comparative efficacy and acceptability of drug treatments for bipolar depression: a multiple-treatments meta-analysis. Acta Psychiatr Scand.

[26] Wang HR, Woo YS, Bahk WM (2014). The potential role of atypical antipsychotics in the treatment of panic disorder. Hum Psychopharmacol.

[27] Kerbusch-Herben V, Cleton A, Berwaerts J, Vandebosch A, Remmerie B (2014). Effect of carbamazepine on the pharmacokinetics of paliperidone extended-release tablets at steady-state. Clin Pharmacol Drug Dev.

[28] Poo SXW, Agius M (2014). Atypical anti-psychotics in adult bipolar disorder: current evidence and updates in the NICE guidelines. Psychiatr Danub.

[29] Moosavi SM, Ahmadi M, Monajemi MB (2014). Risperidone versus risperidone plus sodium valproate for treatment of bipolar disorders: a randomized, double-blind clinical-trial. Glob J Health Sci.

[30] Sajatovic M, Dines P, Fuentes-Casiano E (2015). Asenapine in the treatment of older adults with bipolar disorder. Int J Geriatr Psychiatry.

[31] Van der Heijde D, Tanaka Y, Fleischmann R (2013). Tofacitinib (CP-690,550) in patients with rheumatoid arthritis receiving methotrexate: twelve-month data from a twenty-four-month phase III randomized radiographic study. Arthritis Rheum.

[32] Van Vollenhoven RF, Fleischmann R, Cohen S (2012). Tofacitinib or adalimumab versus placebo in rheumatoid arthritis. N Engl J Med.

[33] Edwards JCW, Szczepanski L, Szechinski J (2004). Efficacy of B-cell-targeted therapy with rituximab in patients with rheumatoid arthritis. N Engl J Med.

[34] Emery P, Deodhar A, Rigby WF (2010). Efficacy and safety of different doses and retreatment of rituximab: a randomised, placebo-controlled trial in patients who are biological naive with active rheumatoid arthritis and an inadequate response to methotrexate (Study Evaluating Rituximab’s Efficacy in MTX iNadequate rEsponders (SERENE)). Ann Rheum Dis.

[35] Kremer JM, Blanco R, Brzosko M (2011). Tocilizumab inhibits structural joint damage in rheumatoid arthritis patients with inadequate responses to methotrexate: results from the double-blind treatment phase of a randomized placebo-controlled trial of tocilizumab safety and prevention of structural joint damage at one year. Arthritis Rheum.

[36] Smolen JS, Beaulieu A, Rubbert-Roth A (2008). Effect of interleukin-6 receptor inhibition with tocilizumab in patients with rheumatoid arthritis (OPTION study): a double-blind, placebocontrolled, randomised trial. Lancet.

[37] Maini R, St Clair EW, Breedveld F (1999). Infliximab (chimeric anti-tumour necrosis factor alpha monoclonal antibody) versus placebo in rheumatoid arthritis patients receiving concomitant methotrexate: a randomised phase III trial. ATTRACT Study Group. Lancet.

[38] Schiff M, Weinblatt ME, Valente R (2014). Head-to-head comparison of subcutaneous abatacept versus adalimumab for rheumatoid arthritis: two-year efficacy and safety findings from AMPLE trial. Ann Rheum Dis.

[39] Kremer JM, Genant HK, Moreland LW (2006). Effects of abatacept in patients with methotrexate-resistant active rheumatoid arthritis: a randomized trial. Ann Intern Med.

[40] Weinblatt ME, Keystone EC, Furst DE (2003). Adalimumab, a fully human anti-tumor necrosis factor alpha monoclonal antibody, for the treatment of rheumatoid arthritis in patients taking concomitant methotrexate: the ARMADA trial. Arthritis Rheum.

[41] Schiff M, Keiserman M, Codding C (2008). Efficacy and safety of abatacept or infliximab vs placebo in ATTEST: a phase III, multi-centre, randomised, double-blind, placebo-controlled study in patients with rheumatoid arthritis and an inadequate response to methotrexate. Ann Rheum Dis.

[42] Tanaka Y, Harigai M, Takeuchi T (2012). Golimumab in combination with methotrexate in Japanese patients with active rheumatoid arthritis: results of the GO-FORTH study. Ann Rheum Dis.

[43] Keystone EC, Genovese MC, Klareskog L (2009). Golimumab, a human antibody to tumour necrosis factor {alpha} given by monthly subcutaneous injections, in active rheumatoid arthritis despite methotrexate therapy: the GO-FORWARD Study. Ann Rheum Dis.

[44] Keystone EC, Kavanaugh AF, Sharp JT (2004). Radiographic, clinical, and functional outcomes of treatment with adalimumab (a human anti-tumor necrosis factor monoclonal antibody) in patients with active rheumatoid arthritis receiving concomitant methotrexate therapy: a randomized, placebo-controlled, 52-week trial. Arthritis Rheum.

[45] Kim HY, LS, Song YW, Yoo DH, Koh EM, Yoo B (2007). A randomized, double-blind, placebo-controlled, phase III study of the human anti-tumor necrosis factor antibody adalimumab administered as subcutaneous injections in Korean rheumatoid arthritis patients treated with methotrexate. APLAR J Rheumatol.

[46] Li Z, Zhang F, Kay J (2013). Safety and efficacy of subcutaneous golimumab in Chinese patients with active rheumatoid arthritis despite MTX therapy: results from a randomized, placebo-controlled, phase 3 trial. Arthritis and Rheumatism.

[47] Machado DA, Guzman RM, Xavier RM (2014). Open-label observation of addition of etanercept versus a conventional disease-modifying antirheumatic drug in subjects with active rheumatoid arthritis despite methotrexate therapy in the Latin American region. J Clin Rheumatol.

[48] Peterfy C, Emery P, Tak PP (2016). MRI assessment of suppression of structural damage in patients with rheumatoid arthritis receiving rituximab: results from the randomised, placebo-controlled, double-blind RA-SCORE study. Ann Rheum Dis.

[49] Bi L, Li Y, He L (2018). Efficacy and safety of certolizumab pegol in combination with methotrexate in methotrexate-inadequate responder Chinese patients with active rheumatoid arthritis: 24-week results from a randomised, double-blind, placebo-controlled phase 3 study. Clin Exp Rheumatol.

[50] Westhovens R, Cole JC, Li T (2006). Improved health-related quality of life for rheumatoid arthritis patients treated with abatacept who have inadequate response to anti-TNF therapy in a double-blind, placebocontrolled, multicentre randomized clinical trial. Rheumatology (Oxford).

[51] Weinblatt ME, Kremer JM, Bankhurst AD (1999). A trial of etanercept, a recombinant tumor necrosis factor receptor:Fc fusion protein, in patients with rheumatoid arthritis receiving methotrexate. N Engl J Med.

[52] Yamamoto K, Takeuchi T, Yamanaka H (2014). Efficacy and safety of certolizumab pegol plus methotrexate in Japanese rheumatoid arthritis patients with an inadequate response to methotrexate: the J-RAPID randomized, placebo-controlled trial. Mod Rheumatol.

[53] Kang YM, Park W, Park YE (2013). Efficacy and safety of certolizumab pegol (CZP) with concomitant methotrexate (MTX) in Korean rheumatoid arthritis (RA) patients (PTS) with an inadequate response to MTX. Ann Rheum Dis.

[54] ODell JR, Mikuls TR, Taylor TH (2013). Therapies for active rheumatoid arthritis after methotrexate failure. N Engl J Med.

[55] Keystone E, Burmester GR, Furie R (2008). Improvement in patient-reported outcomes in a rituximab trial in patients with severe rheumatoid arthritis refractory to anti-tumor necrosis factor therapy. Arthritis Rheum.

[56] Smolen J, Landewe RB, Mease P (2009). Efficacy and safety of certolizumab pegol plus methotrexate in active rheumatoid arthritis: the RAPID 2 study. A randomised controlled trial. Ann Rheum Dis.

[57] (2016). NICE Technology appraisal guidance TA375. Adalimumab, etanercept, infliximab, certolizumab pegol, golimumab, tocilizumab and abatacept for rheumatoid arthritis not previously treated with DMARDs or after conventional DMARDs only have failed.

[58] NICE guidance.

[59] Tanaka Y, Harigai M, Takeuchi T (2016). Clinical efficacy, radiographic progression, and safety through 156 weeks of therapy with subcutaneous golimumab in combination with methotrexate in Japanese patients with active rheumatoid arthritis despite prior methotrexate therapy: final results of the randomized GO-FORTH trial. Mod Rheumatol.

[60] Li Z, Zhang F, Kay J (2016). Efficacy and safety results from a Phase 3, randomized, placebo-controlled trial of subcutaneous golimumab in Chinese patients with active rheumatoid arthritis despite methotrexate therapy. Int J Rheum Dis.

[61] Zhou Q, Zhou Y, Chen H, Wang Z, Tang Z, Liu J (2014). The efficacy and safety of certolizumab pegol (CZP) in the treatment of active rheumatoid arthritis (RA): a meta-analysis from nine randomized controlled trials. Int J Clin Exp Med.

[62] Smolen JS, Aletaha D (2006). What should be our treatment goal in rheumatoid arthritis today?. Clin Exp Rheumatol.

[63] Kunwar S, Collins CE, Constantinescu F (2018). Baricitinib, a Janus kinase inhibitor, in the treatment of rheumatoid arthritis: a systematic literature review and meta-analysis of randomized controlled trials. Clin Rheumatol.

[64] Wu Z-P, Zhang P, Bai J-Z, Liang Y, He J-S, Wang JC (2018). Efficacy and safety of baricitinib for active rheumatoid arthritis in patients with an inadequate response to conventional synthetic or biological disease-modifying anti-rheumatic drugs: a meta-analysis of randomized controlled trials. Exp Ther Med.

[65] Julious SA, Wang SJ (2008). How biased are indirect comparisons, particularly when comparisons are made over time in controlled trials?. Drug Information Journal.

[66] European Medicines Agency Assessment report. Olumiant. International non-proprietary name: baricitinib.

[67] European Medicines Agency Assessment report. Olumiant. International non-proprietary name: baricitinib. Procedure No. EMEA/H/C/004085/0000. Annex 1. Summary of Product Characteristics.

