# Supplementary material for: A Network Meta-Analysis to Compare Effectiveness of Baricitinib and Other Treatments in Rheumatoid Arthritis Patients with Inadequate Response to Methotrexate
Source: J Health Econ Outcomes Res. 2020 Apr 10;7(1):10–23. doi: 10.36469/jheor.2020.12273 (PMC7299462; doi:10.36469/jheor.2020.12273)
Supplement: Supplementary file 1 [file jheor-7-1-12273-s01.pdf]

Journal of Health Economics and Outcomes Research

Supplementary Online Material

Fakhouri W, Wang X, de La Torre I, Nicolay C. A network meta-analysis to compare effectiveness of baricitinib and other treatments in rheumatoid arthritis patients with inadequate response to methotrexate. *JHEOR*. 2020;7(1):10-23.
doi: 10.36469/jheor.2020.12273

Table S1. List of criteria for the inclusion and exclusion of studies during the initial screening process (Level 1)
Table S2. List of criteria for the inclusion and exclusion of studies during the Level 1b Screening process
Table S3. List of criteria for the inclusion and exclusion of studies during the Full-Text Review Process (Level 2)
Table S4. Embase search strategy (Search Conducted on 11 December 2017)
Table S5. Cochrane search strategy (Search Conducted on 11 December 2017)
Table S6. Biosis search strategy (Search Conducted on 11 December 2017)
Table S7. Medline search strategy (Search Conducted on 11 December 2017)
Table S8. Summary of Quality Assessment of included Randomized Controlled Trials (primary analysis, N=19)
Table S9. All trials included in the analysis in MTX-IR population (N=29) - Overview
Table S10. Overview of pre-planned sensitivity analyses
Table S11. Percentage of patients achieving ACR20/50/70 response per trial and treatment arm: MTX-IR population
Table S12. Primary Analysis: Relative treatment effect of pairwise comparisons expressed as Post. Median odds ratios (with 95% CrIs) - ACR20 response at week 24: MTX-IR (Simultaneous Fixed-effects model)
Table S13. Primary Analysis: Relative treatment effect of pairwise comparisons expressed as post. Median odds ratios (with 95% CrIs) - ACR50 response at week 2 - MTX-IR (Simultaneous Fixed-effects model)
Table S14. Primary Analysis: Relative treatment effect of pairwise comparisons expressed as Post. Median odds ratios (with 95% CRIs) - ACR70 response at week 24- MTX-IR (Simultaneous Fixed-effects model)
Table S15. Primary Analysis: Relative treatment effect of pairwise comparisons expressed as Post. Median odds ratios (with 95% CrIs) - ACR20 response at week 24: MTX-IR (Simultaneous Random-effects model)
Table S16. Primary Analysis: Relative treatment effect of pairwise comparisons expressed as Post. Median odds ratios (with 95% CrIs) – ACR50 response at week 24: MTX-IR (Simultaneous Random effects-model)
Table S17. Primary Analysis: Relative treatment effect of pairwise comparisons expressed as Post. Median odds ratios (with 95% CrIs) – ACR70 response at week 24: MTX-IR (Simultaneous Random-effects model)
Table S18. Baseline Risk-adjustment (Primary analysis): Relative treatment effect of pairwise comparisons expressed as Post. Median odds ratios (with 95% CrIs) - ACR20 response at week 24: MTX-IR (Simultaneous Fixed-effects model)
Table S19. Baseline Risk-adjustment (Primary analysis): Relative treatment effect of pairwise comparisons expressed as Post. Median odds ratios (with 95% CrIs) – ACR50 response at week 24: MTX-IR (Simultaneous Fixed-effects model)
Table S20. Baseline Risk-adjustment (Primary analysis): Relative treatment effect of pairwise comparisons expressed as Post. Median odds ratios (with 95% CrIs) – ACR70 response at week 24: MTX-IR (Simultaneous Fixed-effects model)
Table S21. Sensitivity analysis including trials with prior bDMARD use of up to 20%: Relative treatment effect of pairwise comparisons expressed as Post. Median odds ratios (with 95% CrIs) - ACR20 response at week 24: MTX-IR (Simultaneous Fixed-effects model)
Table S22. Sensitivity analysis including trials with prior bDMARD use of up to 20%: Relative treatment effect of pairwise comparisons expressed as Post. Median odds ratios (with 95% CrIs) – ACR50 response at week 24: MTX-IR (Simultaneous Fixed-effects model)
Table S23. Sensitivity analysis including trials with prior bDMARD use of up to 20%: Relative treatment effect of pairwise comparisons expressed as Post. Median odds ratios (with 95% CrIs) – ACR70 response at week 24: MTX-IR (Simultaneous Fixed-effects model)
Table S24. Sensitivity analysis excluding trials conducted solely in Asia-Pacific and/or low MTX dose: Relative treatment effect of pairwise comparisons expressed as Post. Median odds ratios (with 95% CrIs) – ACR20 response at week 24: MTX-IR (Simultaneous Fixed-effects model)
Table S25. Sensitivity analysis excluding trials conducted solely in Asia-Pacific and/or low MTX dose: Relative treatment effect of pairwise comparisons expressed as Post. Median odds ratios (with 95% CrIs) – ACR50 response at week 24: MTX-IR (Simultaneous Fixed-effects model)

This is an open-access article distributed under the terms of the Creative Commons Attribution 4.0 International License (CCBY-4.0). View this license's legal deed at http://creativecommons.org/licenses/by/4.0 and legal code at http://creativecommons.org/licenses/by/4.0/legalcode for more information.

Table S26. Sensitivity analysis excluding trials conducted solely in Asia-Pacific and/or low MTX dose: Relative treatment effect of pairwise comparisons expressed as Post. Median odds ratios (with 95% CrIs) – ACR70 response at week 24: MTX-IR (Simultaneous Fixed-effects model)
Table S27. Model Fit Summary
Figure S1. PRISMA Diagram
Figure S2. Response rate in PBO+MTX arm vs. ln (Risk Ratio) - Primary analysis
Figure S3. Sensitivity analysis excluding trials conducted solely in Asia-Pacific and/or low MTX dose: Network of Evidence - Simultaneous fixed effects: ACR20
Figure S4. BARI 4mg + MTX: Estimated posterior median ACR response rates across primary and main sensitivity analyses
Figure S5. TOFA 5mg + MTX: Estimated posterior median ACR response rates across primary and main sensitivity analyses
Figure S6. ADA 40mg + MTX: Estimated posterior median ACR response rates across primary and main sensitivity analyses
Figure S7. CZP + MTX: Estimated posterior median ACR response rates across primary and main sensitivity analyses
Figure S8. ETN + MTX: Estimated posterior median ACR response rates across primary and main sensitivity analyses
Figure S9. GOL 50mg + MTX: Estimated posterior median ACR response rates across primary and main sensitivity analyses
Figure S10. IFX 3mg + MTX: Estimated posterior median ACR response rates across primary and main sensitivity analyses
Figure S11. ABA 10mg + MTX: Estimated posterior median ACR response rates across primary and main sensitivity analyses
Figure S12. ABA SUBCUT + MTX: Estimated posterior median ACR response rates across primary and main sensitivity analyses
Figure S13. RTX + MTX: Estimated posterior median ACR response rates across primary and main sensitivity analyses
Figure S14. TCZ + MTX: Estimated posterior median ACR response rates across primary and main sensitivity analyses
Figure S15. SARI 200mg + MTX: Estimated posterior median ACR response rates across primary and main sensitivity analyses
Figure S16. PBO + MTX: Estimated posterior median ACR response rates across primary and main sensitivity analyses

This supplementary material has been provided by the authors to give readers additional information about their work.

1. Contents

1. Contents 1
2. Inclusion and Exclusion criteria 6
3. Search Terms 12
4. PRISMA diagram 22
5. Quality assessment of the included trials 23
6. List of trials included in the analysis 25
7. Methodology 27
   7.1. Endpoints 27
   7.2. Overview of Analyses 27
   7.3. Bayesian Mixed Treatment Comparisons 30
   7.4. Sensitivity Analyses 33
8. ACR Response Data 36
9. Results – Primary Analyses 38
   9.1. Primary analyses (Simultaneous Fixed – effects) 38
      9.1.1. ACR20 Response 38
      9.1.2. ACR50 Response 41
      9.1.3. ACR70 response 44
   9.2. Primary analyses (Simultaneous Random – effects) 47
      9.2.1. ACR20 Response 47
      9.2.2. ACR50 Response 50
      9.2.3. ACR70 Response 53
10. Results – Sensitivity Analyses 56
   10.1. Baseline-risk adjustment 56
      10.1.1. ACR20 Response 58
      10.1.2. ACR50 Response 61
      10.1.3. ACR70 Response 64
   10.2. Inclusion of Trials with Prior bDMARD use of up to 20% 67
      10.2.1. ACR20 Response 67
      10.2.2. ACR50 Response 71
      10.2.3. ACR70 Response 75
   10.3. Trials Conducted in Asia Pacific/Low Dose MTX 79
      10.3.1. ACR20 response 79
      10.3.2. ACR50 response 83
      10.3.3. ACR70 response 86
11. Model Fit 89

12. Posterior Median ACR response, by Treatment and Analysis (Forest Plots)....90
13. References 97

Table S1 List of criteria for the inclusion and exclusion of studies during the initial screening process (Level 1)....6
Table S2 List of criteria for the inclusion and exclusion of studies during the Level 1b Screening process....7
Table S3 List of criteria for the inclusion and exclusion of studies during the Full-Text Review Process (Level 2)....10
Table S4 Embase search strategy (Search Conducted on 11 December 2017)....12
Table S5 Cochrane search strategy (Search Conducted on 11 December 2017)....15
Table S6 Biosis search strategy (Search Conducted on 11 December 2017) 17
Table S7 Medline search strategy (Search Conducted on 11 December 2017)....19
Table S8 Summary of Quality Assessment of included Randomized Controlled Trials (primary analysis, N=19)....23
Table S9 All trials included in the analysis in MTX-IR population (N=29) - Overview 25
Table S10 Overview of pre-planned sensitivity analyses 34
Table S11 Percentage of patients achieving ACR20/50/70 response per trial and treatment arm: MTX-IR population 36
Table S12 Primary Analysis: Relative treatment effect of pairwise comparisons expressed as Post. Median odds ratios (with 95% CrIs) - ACR20 response at week 24: MTX-IR (Simultaneous Fixed-effects model)....38
Table S13 Primary Analysis: Relative treatment effect of pairwise comparisons expressed as post. Median odds ratios (with 95% CrIs) - ACR50 response at week 2 - MTX-IR (Simultaneous Fixed-effects model)....41
Table S14 Primary Analysis: Relative treatment effect of pairwise comparisons expressed as Post. Median odds ratios (with 95% CRIs) - ACR70 response at week 24- MTX-IR (Simultaneous Fixed-effects model)....44
Table S15 Primary Analysis: Relative treatment effect of pairwise comparisons expressed as Post. Median odds ratios (with 95% CrIs) - ACR20 response at week 24: MTX-IR (Simultaneous Random-effects model) 47

Table S16 Primary Analysis: Relative treatment effect of pairwise comparisons expressed as Post. Median odds ratios (with 95% CrIs) – ACR50 response at week 24: MTX-IR (Simultaneous Random effects-model) 50

Table S17 Primary Analysis: Relative treatment effect of pairwise comparisons expressed as Post. Median odds ratios (with 95% CrIs) – ACR70 response at week 24: MTX-IR (Simultaneous Random-effects model) 53

Table S18 Baseline Risk-adjustment (Primary analysis): Relative treatment effect of pairwise comparisons expressed as Post. Median odds ratios (with 95% CrIs) - ACR20 response at week 24: MTX-IR (Simultaneous Fixed-effects model) 58

Table S19 Baseline Risk-adjustment (Primary analysis): Relative treatment effect of pairwise comparisons expressed as Post. Median odds ratios (with 95% CrIs) – ACR50 response at week 24: MTX-IR (Simultaneous Fixed-effects model) 61

Table S20 Baseline Risk-adjustment (Primary analysis): Relative treatment effect of pairwise comparisons expressed as Post. Median odds ratios (with 95% CrIs) – ACR70 response at week 24: MTX-IR (Simultaneous Fixed-effects model) 64

Table S21 Sensitivity analysis including trials with prior bDMARD use of up to 20%: Relative treatment effect of pairwise comparisons expressed as Post. Median odds ratios (with 95% CrIs) - ACR20 response at week 24: MTX-IR (Simultaneous Fixed-effects model) 67

Table S22 Sensitivity analysis including trials with prior bDMARD use of up to 20%: Relative treatment effect of pairwise comparisons expressed as Post. Median odds ratios (with 95% CrIs) – ACR50 response at week 24: MTX-IR (Simultaneous Fixed-effects model) 71

Table S23 Sensitivity analysis including trials with prior bDMARD use of up to 20%: Relative treatment effect of pairwise comparisons expressed as Post. Median odds ratios (with 95% CrIs) – ACR70 response at week 24: MTX-IR (Simultaneous Fixed-effects model) 75

Table S24 Sensitivity analysis excluding trials conducted solely in Asia-Pacific and/or low MTX dose: Relative treatment effect of pairwise comparisons expressed as Post. Median odds ratios (with 95% CrIs) – ACR20 response at week 24: MTX-IR (Simultaneous Fixed-effects model)................80

Table S25 Sensitivity analysis excluding trials conducted solely in Asia-Pacific and/or low MTX dose: Relative treatment effect of pairwise comparisons expressed as Post. Median odds ratios (with 95% CrIs) – ACR50 response at week 24: MTX-IR (Simultaneous Fixed-effects model)................83

Table S26 Sensitivity analysis excluding trials conducted solely in Asia-Pacific and/or low MTX dose: Relative treatment effect of pairwise comparisons expressed as Post. Median odds ratios (with 95% CrIs) – ACR70 response at week 24: MTX-IR (Simultaneous Fixed-effects model)................86

Table S27 Model Fit Summary 89

Figure S1 PRISMA Diagram 22

Figure S2 Response rate in PBO+MTX arm vs. ln (Risk Ratio) - Primary analysis 56

Figure S3 Sensitivity analysis excluding trials conducted solely in Asia-Pacific and/or low MTX dose: Network of Evidence - Simultaneous fixed effects: ACR20..79

Figure S4 BARI 4mg + MTX: Estimated posterior median ACR response rates across primary and main sensitivity analyses..90

Figure S5 TOFA 5mg + MTX: Estimated posterior median ACR response rates across primary and main sensitivity analyses..90

Figure S6 ADA 40mg + MTX: Estimated posterior median ACR response rates across primary and main sensitivity analyses..91

Figure S7 CZP + MTX: Estimated posterior median ACR response rates across primary and main sensitivity analyses 91

Figure S8 ETN + MTX: Estimated posterior median ACR response rates across primary and main sensitivity analyses 92

Figure S9 GOL 50mg + MTX: Estimated posterior median ACR response rates across primary and main sensitivity analyses..92

Figure S10 IFX 3mg + MTX: Estimated posterior median ACR response rates across primary and main sensitivity analyses..93

Figure S11 ABA 10mg + MTX: Estimated posterior median ACR response rates across primary and main sensitivity analyses..93

Figure S12 ABA SUBCUT + MTX: Estimated posterior median ACR response rates across primary and main sensitivity analyses..94

Figure S13 RTX + MTX: Estimated posterior median ACR response rates across primary and main sensitivity analyses..94

Figure S14 TCZ + MTX: Estimated posterior median ACR response rates across primary and main sensitivity analyses..95

Figure S15 SARI 200mg + MTX: Estimated posterior median ACR response rates across primary and main sensitivity analyses..95

Figure S16 PBO + MTX: Estimated posterior median ACR response rates across primary and main sensitivity analyses...96

2. Inclusion and Exclusion criteria

The inclusion and exclusion criteria was based on a strategy (Table 1) that identified the population and disease condition, interventions, comparators, outcomes, and study types of interest (also known as the PICOS criteria). The criteria listed in Table 1 were used after the initial, broad searches were completed and after the top-level list of articles (titles and abstracts) was identified.

However, due to the large number of studies included at the level 1 screen, the studies identified as being relevant for the review were re-screened using the more stringent criteria presented in Table 2 (Protocol Amendment). The criteria listed in Table 3 were used at the level 2 screening the full-text articles.

Table S1 List of criteria for the inclusion and exclusion of studies during the initial screening process (Level 1)

Criteria	Included	Excluded
Population	▪ Adult (≥ 18 years) patients with moderately to severely active RA (including patients with early and established RA)<br>▪ Treatment-naïve patients<br>▪ Patients who had intolerance or inadequate response to prior conventional DMARDs<br>▪ Patients who had intolerance or inadequate response to previous bDMARDs	▪ Juvenile idiopathic arthritis<br>▪ Studies that include only juveniles<br>▪ Patients with mild RA[a]; if the study population is mixed (i.e., mild to severe), exclude those studies in which data are not reported separately for moderate or severely active RA
Interventions	▪ Baricitinib (Olumiant)	▪ Studies that do not have an intervention of interest in at least 1 arm
Comparators	▪ Methotrexate (Trexall, Rheumatrex, amethopterin, Rasuvo, Otrexup)<br>▪ Sulfasalazine (Azulfidine, Salazopyrin, Sulazine, sulfazine)<br>▪ Leflunomide (Arabloc, Arava, Lunava, Respo)<br>▪ Hydroxychloroquine (Plaquenil, Axemal, Dolquine, Quensyl, Quineprox)<br>▪ Azathioprine (Azasan, Imuran, Azamun, Imurel)<br>▪ Infliximab (Remicade, Inflectra)<br>▪ Adalimumab (Humira, Trudexa, ABP 501, BI695501, CHS-1420, GP2017, M923, PF-06410293)<br>▪ Certolizumab pegol (Cimzia)<br>▪ Golimumab (Simponi)<br>▪ Etanercept (Enbrel, Avent, BX2922, CHS-0214, ENIA11, Etacept, Etanar, GP2013, GP2015, HD203, LBEC0101, M923, PRX-106, SB4, TuNEX, Yisaipu)<br>▪ Abatacept (Orencia)<br>▪ Anakinra (Kineret)<br>▪ Rituximab (Rituxan, MabThera, Zytux, Reditux)<br>▪ Tocilizumab (Actemra, RoActemra, atlizumab)<br>▪ Sarilumab (Kevzara)<br>▪ Sirukumab (proposed brand name: Plivensia)<br>▪ Tofacitinib (Xeljanz, Jakvinus, tasocitinib)	▪ Studies that do not have a comparator of interest in at least 1 arm<br>▪ Non-pharmacological studies, e.g., exercise, Chinese medicine, etc.

Criteria	Included	Excluded
	At the level 1 screening, all therapy versions (i.e., any dose or combination) of the interventions listed above will be included	
Study design	▪ Randomised, controlled, prospective clinical trials<br>▪ Randomised clinical trials that compare interventions in clinical settings<br>▪ Long-term follow-up studies (e.g., open-label follow-up studies with continuation of treatments in their respective randomised group)<br>▪ Systematic reviews (including meta-analyses)[b]	▪ Non-randomised clinical trials<br>▪ Preclinical studies<br>▪ Phase 1 studies<br>▪ Prognostic studies<br>▪ Retrospective studies<br>▪ Prospective observational studies<br>▪ Case reports<br>▪ Commentaries and letters (publication type)<br>▪ Consensus reports<br>▪ Non-systematic reviews<br>▪ Secondary analyses<br>▪ Animal models
Language	▪ All languages	▪ None
Date	▪ 1999 to present	▪ None

bDMARD = biologic disease-modifying antirheumatic drug; DAS-28 = Disease Activity Score in 28 Joints; DMARD = disease-modifying antirheumatic drug; RA = rheumatoid arthritis.

[a] If the disease severity of included patients was not clearly stated in the article, the following approach was used and validated by Lilly: if DAS-28 scores were reported, then DAS-28 scores of > 3.2 were considered to be moderate RA; DAS-28 scores of > 5.1 were considered to be severe RA. If DAS-28 scores were not reported, then swollen and tender joint counts both > 6 was considered to be a good proxy for moderate to severe RA.

[b] Systematic reviews and meta-analyses will be used only for identification of primary studies that may have been missed in the electronic searches.

Table S2 List of criteria for the inclusion and exclusion of studies during the Level 1b Screening process

Criteria	Included	Excluded
Population	▪ Adult (≥ 18 years) patients with moderately to severely active RA (including patients with early and established RA)<br>▪ Treatment-naïve patients<br>▪ Patients who had intolerance or inadequate response to prior conventional DMARDs<br>▪ Patients who had intolerance or inadequate response to previous bDMARDs	▪ Juvenile idiopathic arthritis<br>▪ Studies that include only juveniles<br>▪ Patients with mild RA[a]; if the study population is mixed (i.e., mild to severe), exclude those studies in which data are not reported separately for moderate or severely active RA<br>▪ Patients with low disease activity

Criteria	Included	Excluded
Interventions and comparators	▪ Baricitinib (Olumiant)<br>▪ Methotrexate (Trexall)<br>▪ Sulfasalazine (Azulfidine)<br>▪ Leflunomide (Arabloc)<br>▪ Hydroxychloroquine (Plaquenil)<br>▪ Infliximab (Remicade, Inflectra)<br>▪ Adalimumab (Humira, Trudexa)<br>▪ Certolizumab (Cimzia)<br>▪ Golimumab (Simponi)<br>▪ Etanercept (Enbrel)<br>▪ Abatacept (Orencia)<br>▪ Anakinra (Kineret)<br>▪ Rituximab (Rituxan)<br>▪ Tocilizumab (Actemra, RoActemra)<br>▪ Sarilumab (Kevzara)<br>▪ Sirukumab (proposed brand name: Plivensia)<br>▪ Tofacitinib (Xeljanz, Jakvinus)<br>▪ Placebo<br>At the level 1 screening, all therapy versions (i.e., any dose or combination) of the interventions listed above will be included	▪ Studies that do not have an intervention or comparator of interest in at least 2 of the study arms<br>▪ Non-pharmacological studies, e.g., exercise, Chinese medicine, etc.<br>▪ **Protocol Amendment:** Biosimilars<br>▪ **Protocol Amendment:** Azathioprine (Azasan, Imuran)<br>▪ Studies comparing conventional DMARDs to NSAIDs, glucocorticoids, Diclofenac, etc.
Study design	▪ Phase 3, randomised, controlled, prospective clinical trials<br>▪ Randomised clinical trials that compare interventions in clinical settings<br>▪ Long-term follow-up or extension studies of RCTs where patients remain in their respective randomised group<br>▪ Systematic reviews (including meta-analyses) published in 2014 or 2015[b]	▪ **Protocol Amendment:** Phase 2, randomised, controlled, prospective clinical trials<br>▪ Non-randomised clinical trials<br>▪ Single-arm studies<br>▪ **Protocol Amendment:** Long-term follow-up or extension studies of RCTs in which patients do not remain in their respective randomised group<br>▪ **Protocol Amendment:** Maintenance studies and step-down treatment studies<br>▪ Preclinical studies<br>▪ Phase 1 studies<br>▪ Prognostic studies<br>▪ Retrospective studies<br>▪ Prospective observational studies<br>▪ Case reports<br>▪ Commentaries and letters (publication type)<br>▪ Consensus reports<br>▪ Pooled analyses<br>▪ **Protocol Amendment:** Post hoc analyses<br>▪ Non-systematic reviews

Criteria	Included	Excluded
		▪ **Protocol Amendment:** Systematic reviews (including meta-analyses) published prior to 2014<br>– Secondary analyses<br>– Animal models
Outcomes	▪ Studies reporting efficacy and safety data, HRQoL, WPAI-RA, or health care resource utilisation<br>▪ MRI studies that specifically mention the Sharp/Van der Heijde bone erosion score	▪ **Protocol Amendment:** Studies that report only MRI outcomes and do not specifically mention the Sharp/Van der Heijde bone erosion score<br>▪ **Protocol Amendment:** Studies that report only bone mineral density<br>▪ **Protocol Amendment:** Studies that investigate ultrasound and radiography in assessing bone damage
Language	▪ English-language publications	▪ Non–English-language publications
Date	▪ 1999 to present	▪ None

bDMARD = biologic disease-modifying antirheumatic drug; DAS-28 = Disease Activity Score in 28 Joints; DMARD = disease-modifying antirheumatic drug; HRQoL = health-related quality of life; MRI = magnetic resonance imaging; NSAID = non-steroidal anti-inflammatory drug; RA = rheumatoid arthritis; RCT = randomised, controlled trial; WPAI-RA = Work Productivity and Activity Impairment Questionnaire–Rheumatoid Arthritis.

[a] If the disease severity of included patients was not clearly stated in the article, the following approach was used and validated by Lilly: if DAS-28 scores were reported, then DAS-28 scores of > 3.2 were considered to be moderate RA; DAS-28 scores of > 5.1 were considered to be severe RA. If DAS-28 scores were not reported, then swollen and tender joint counts both > 6 was considered to be a good proxy for moderate to severe RA.

[b] Systematic reviews and meta-analyses will be used only for identification of primary studies that may have been missed in the electronic searches.

Table S3 List of criteria for the inclusion and exclusion of studies during the Full-Text Review Process (Level 2)

Criteria	Included	Excluded
Population	▪ Same as Table 2	▪ Same as Table 2
Interventions	▪ Interventions listed in Table 2 that meet the following criteria:<br>– Licensed treatments at the labelled doses<br>– Treatments not yet licensed in any form or dose	▪ Same as Table 2
Comparators	▪ Same as Table 2	▪ Same as Table 2
Outcomes[a]	To be included in the review, a study must report at least 1 of the outcomes of interest.<br>▪ Efficacy measurements:<br>– ACR criteria<br>– ACR score<br>– Proportion of patients achieving an ACR 20 response<br>– Proportion of patients achieving an ACR 50 response<br>– Proportion of patients achieving an ACR 70 response<br>– ACR remission<br>– Proportion of patients achieving an ACR 50 response in the subgroup of patients who are TNFα inhibitor naïve, have inadequate response to TNFα or other biologics, or who are intolerant to TNFα or other biologics (if reported)<br>– Proportion of patients achieving an ACR 20 response in the subgroup of patients who are TNFα inhibitor naïve, have inadequate response to TNFα or other biologics or who are intolerant to TNFα or other biologics (if reported)<br>– Individual components of the ACR:<br>• HAQ-DI<br>• Pain VAS<br>• Tender joint count<br>• Swollen joint count<br>• Physician global assessment<br>• Patient global assessment<br>• Modified Sharp score<br>• Erosion score<br>• Joint space narrowing score<br>• DAS-28 ESR for RA<br>• DAS-28 CRP for RA<br>• SDAI<br>• CDAI<br>• Physical function assessed by HAQ or HAQ-DI<br>– Endpoints measuring the following:<br>• Morning joint stiffness and/or joint pain (severity and duration) (may be assessed by different instruments)<br>• Tiredness or fatigue (may be assessed by different instruments)<br>– EULAR or ACR remission defined as:<br>• CDAI score ≤ 2.8<br>• SDAI score ≤ 3.3<br>• DAS-28 < 2.6	▪ None

Criteria	Included	Excluded
	• RAPID3 ≤ 1<br>• DAS-44 < 1.6<br>• Boolean definition of remission (EULAR or ACR where all measures must be < 1)<br>• WPAI-RA<br>• Health care resource utilisation<br>▪ HRQoL outcomes from the following:<br>– EQ-5D<br>– SF-36<br>Safety outcomes reported at study endpoint:<br>▪ Overall rate of AEs<br>▪ Overall rate of serious AEs<br>▪ Discontinuations due to<br>– Lack of efficacy<br>– AEs<br>▪ Individual AEs, such as the following:<br>– Specific myelosuppressive events, e.g., anaemia, leukopaenia, neutropaenia, or thrombocytopaenia or lymphopaenia or lymphocytopaenia<br>– Thrombocytosis<br>– Serious infections<br>– Opportunistic infections<br>– Malignancies<br>– Cardiovascular events<br>– Elevations in ALT or AST (> 3 times upper limit of normal) with total bilirubin (> 2 times upper limit of normal)<br>– Injection-related combinations<br>– Intravenous reactions<br>▪ Death<br>▪ Initial or prolonged inpatient hospitalisation	
Study design	▪ Randomised, controlled, prospective clinical trials<br>▪ Randomised clinical trials that compare interventions in clinical settings<br>▪ Long-term follow-up studies (e.g., open-label follow-up studies if patients continued in the group to which they were randomised)	▪ Same as Table 2<br>▪ Systematic reviews and meta-analyses
Language	▪ English-language and German-language publications	▪ Other non–English-language publications
Date	▪ 1999 to present	▪ None

ACR = American College of Rheumatology; AE = adverse event; ALT = alanine aminotransferase; AST = aspartate aminotransferase; CDAI = Clinical Disease Activity Index; CRP = C-reactive protein; DAS-28 = Disease Activity Score in 28 Joints; DAS-44 = Disease Activity Score in 44 Joints; ESR = erythrocyte sedimentation rate; EULAR = European League Against Rheumatism; HAQ = Health Assessment Questionnaire; HAQ-DI = Health Assessment Questionnaire–Disability Index; HRQoL = health-related quality of life; RA = rheumatoid arthritis; RAPID3 = Routine Assessment of Patient Index Data 3; SDAI = Simplified Disease Activity Index; SF-36 = SF-36 Health Survey; TNFα = tumour necrosis factor alpha; VAS = visual analogue scale; WPAI-RA = Work Productivity and Activity Impairment Questionnaire–Rheumatoid Arthritis.

[a] Time points of interest include 12, 24, and 52 weeks. If any of these specific time points are not available, we will collect data at the following time points: 12 weeks ± 2 weeks, 24 weeks ± 4 weeks, and 52 weeks ± 8 weeks.

3. Search Terms

Table S4 Embase search strategy (Search Conducted on 11 December 2017)

Search No.	Terms	Results
**Population and/or patients**		
#1	'rheumatoid arthritis'/exp	187,860
#2	(rheumatoid:ti,ab,de OR reumatoid:ti,ab,de) AND (arthrit*:ti,ab,de OR disease*:ti,ab,de)	209,600
#3	#1 OR #2	211,674
**Intervention**		
#4	'baricitinib'/exp	410
#5	baricitinib:ti,ab,de,tn OR olumiant:ti,ab,de,tn	413
#6	#4 OR #5	413
**Comparator: cDMARDs**		
#7	'methotrexate'/exp OR 'salazosulfapyridine'/exp OR 'leflunomide'/exp OR 'hydroxychloroquine'/exp	184,376
#8	Methotrexate:ti,ab,de,tn OR MTX:ti,ab,de,tn OR rheumatrex:ti,ab,de,tn OR amethopterin:ti,ab,de,tn OR 'methotrexate sodium':ti,ab,de,tn OR rasuvo:ti,ab,de,tn OR trexall:ti,ab,de,tn OR otrexup:ti,ab,de,tn OR sulfasalazine:ti,ab,de,tn OR azulfidine:ti,ab,de,tn OR salazopyrin:ti,ab,de,tn OR sulazine:ti,ab,de,tn OR sulfazine:ti,ab,de,tn OR leflunomide:ti,ab,de,tn OR arabloc:ti,ab,de,tn OR arava:ti,ab,de,tn OR lunava:ti,ab,de,tn OR repso:ti,ab,de,tn OR hydroxychloroquine:ti,ab,de,tn OR plaquenil:ti,ab,de,tn OR axemal:ti,ab,de,tn OR dolquine:ti,ab,de,tn OR quensyl:ti,ab,de,tn OR quineprox:ti,ab,de,tn	183,171
#9	#7 OR #8	191,491
**Comparator: bDMARDs**		
#10	'infliximab'/exp OR 'adalimumab'/exp OR 'certolizumab pegol'/exp OR 'golimumab'/exp OR 'etanercept'/exp OR 'abatacept'/exp OR 'recombinant interleukin 1 receptor blocking agent'/exp OR 'rituximab'/exp OR 'tocilizumab'/exp OR 'sarilumab'/exp OR 'sirukumab'/exp OR 'tofacitinib'/exp	124,151

Search No.	Terms	Results
#11	infliximab:ti,ab,de,tn OR remicade:ti,ab,de,tn OR 'monoclonal antibody cA2':ti,ab,de,tn OR 'MAb cA2':ti,ab,de,tn OR 'CT-P13':ti,ab,de,tn OR Remsima:ti,ab,de,tn OR Inflectra:ti,ab,de,tn OR adalimumab:ti,ab,de,tn OR humira:ti,ab,de,tn OR trudexa:ti,ab,de,tn OR '331731-18-1':ti,ab,de,tn,rn OR 'D2E7 antibody':ti,ab,de,tn OR LU200134:ti,ab,de,tn OR 'ABP 501':ti,ab,de,tn OR BI695501:ti,ab,de,tn OR M923:ti,ab,de,tn OR 'PF-06410293':ti,ab,de,tn OR GP2017:ti,ab,de,tn OR 'CHS-1420':ti,ab,de,tn OR certolizumab:ti,ab,de,tn OR 'certolizumab pegol':ti,ab,de,tn OR cimzia:ti,ab,de,tn OR golimumab:ti,ab,de,tn OR simponi:ti,ab,de,tn OR 'TNFR-Fc fusion protein':ti,ab,de,tn OR etanercept:ti,ab,de,tn OR enbrel:ti,ab,de,tn OR Avent:ti,ab,de,tn OR BX2922:ti,ab,de,tn OR 'CHS-0214':ti,ab,de,tn OR ENIA11:ti,ab,de,tn OR Etacept:ti,ab,de,tn OR Etanar:ti,ab,de,tn OR GP2013:ti,ab,de,tn OR GP2015:ti,ab,de,tn OR HD203:ti,ab,de,tn OR LBEC0101:ti,ab,de,tn OR M923:ti,ab,de,tn OR 'PRX-106':ti,ab,de,tn OR SB4:ti,ab,de,tn OR TuNEX:ti,ab,de,tn OR Yisaipu:ti,ab,de,tn OR abatacept:ti,ab,de,tn OR orencia:ti,ab,de,tn OR anakinra:ti,ab,de,tn OR kineret:ti,ab,de,tn OR rituximab:ti,ab,de,tn OR rituxan:ti,ab,de,tn OR mabthera:ti,ab,de,tn OR zytux:ti,ab,de,tn OR reditux:ti,ab,de,tn OR tocilizumab:ti,ab,de,tn OR atlizumab:ti,ab,de,tn OR actemra:ti,ab,de,tn OR roactemra:ti,ab,de,tn OR sarilumab:ti,ab,de,tn OR sirukumab:ti,ab,de,tn OR tofacitinib:ti,ab,de,tn OR tasocitinib:ti,ab,de,tn OR xeljanz:ti,ab,de,tn OR jakvinus:ti,ab,de,tn OR kevzara:ti,ab,de,tn OR plivensia:ti,ab,de,tn	127,327
#12	#10 OR #11	127,327
**All interventions and comparators**		
#13	#6 OR #9 OR #12	283,407
**Study type (RCTs)**		
#14	'randomized controlled trial (topic)'/exp OR 'phase 3 clinical trial (topic)'/exp OR 'phase 2 clinical trial (topic)'/exp OR 'controlled clinical trial (topic)'/exp OR 'randomization'/exp OR 'clinical trial (topic)'/de	311,082
#15	'randomized controlled trial'/exp OR 'controlled clinical trial'/exp OR 'phase 2 clinical trial'/exp OR 'phase 3 clinical trial'/exp OR 'phase 4 clinical trial'/exp OR 'multicenter study'/exp	762,802
#16	Randomized:ti,ab OR randomised:ti,ab OR randomly:ti,ab	1,000,302
#17	('randomized controlled' NEXT/1 trial*):ti,ab,de OR ('randomised controlled' NEXT/1 trial*):ti,ab,de OR ('randomized clinical' NEXT/1 trial*):ti,ab,de OR ('randomised clinical' NEXT/1 trial*):ti,ab,de OR (randomized NEXT/1 trial*):ti,ab,de OR (randomised NEXT/1 trial*):ti,ab,de OR 'random allocation':ti,ab,de OR 'double blind method':ti,ab,de OR 'single blind method':ti,ab,de OR ((singl*:ti,ab,de OR doubl*:ti,ab,de OR treb*:ti,ab,de OR tripl*:ti,ab,de) AND (blind*:ti,ab,de OR mask*:ti,ab,de)) OR (allocated NEXT/1 random*):ti,ab,de OR (random NEXT/1 assignment*):ti,ab,de OR (open-label NEXT/1 trial*):ti,ab,de OR (open-label NEXT/1 stud*):ti,ab,de OR 'open label trial':ti,ab,de OR ('non-blinded' NEXT/1 stud*):ti,ab,de	871,158
#18	#14 OR #15 OR #16 OR #17	1,630,752
**Exclusion terms, excluding animals**		
#19	'animal'/exp NOT 'human'/exp	4,953,190
**Exclusion terms, by study type**		

Search No.	Terms	Results
#20	Comment*:ti OR Letter:it OR Editorial:it OR 'case report'/exp OR 'phase 1 clinical trial'/exp	3,732,046
#21	'case study':ti OR 'case studies':ti OR 'case report':ti OR 'case reports':ti OR 'case series':ti	334,134
#22	#20 OR #21	3,804,477
**Exclusion terms, population**		
#23	'juvenile rheumatoid arthritis'/exp	18,670
#24	'juvenile idiopathic arthritis':ti,ab,de OR (juvenile:ti,ab,de AND arthritis:ti,ab,de)	21,044
#25	#23 OR #24	21,044
**All relevant studies**		
#26	#3 AND #13 AND #18	9,180
#27	#26 NOT (#19 OR #22 OR #25)	7,676
**Limits**		
#28	#27 AND [1-5-2016]/sd NOT [11-12-2017]/sd AND [2016-2017]/py Filters applied: Dates limited from 2015/03/01 to present	1,387

bDMARD = biologic disease-modifying antirheumatic drug; cDMARD = conventional disease-modifying antirheumatic drug;

RCT = randomised, controlled trial.

Table S5 Cochrane search strategy (Search Conducted on 11 December 2017)

Search No.	Terms	Results
**Population and/or patients**		
#1	#1 MeSH descriptor: [Arthritis, Rheumatoid] explode all trees	4,811
#2	#2 (rheumatoid or reumatoid) and (arthrit* or disease*) (Word variations have been searched)	10,547
#3	#1 OR #2	10,724
**Intervention**		
#4	baricitinib:kw (Word variations have been searched)	108
#5	Baricitinib OR olumiant (Word variations have been searched)	115
#6	#4 OR #5	115
**Comparator: cDMARDs**		
#7	methotrexate OR sulfasalazine OR leflunomide OR hydroxychloroquine:kw (Word variations have been searched)	7,468
#8	methotrexate OR MTX OR rheumatrex OR amethopterin OR "methotrexate sodium" OR rasuvo OR trexall OR otrexup OR sulfasalazine OR azulfidine OR salazopyrin OR sulazine OR sulfazine OR leflunomide OR arabloc OR arava OR lunava OR repso OR hydroxychloroquine OR plaquenil OR axemal OR dolquine OR quensyl OR quineprox (Word variations have been searched)	9,745
#9	#7 OR #8	9,745
**Comparator: bDMARDs**		
#10	infliximab OR adalimumab OR "certolizumab pegol" OR golimumab OR "TNFR-Fc fusion protein" OR abatacept OR anakinra OR rituximab OR tocilizumab OR atlizumab OR sarilumab OR sirukumab OR tofacitinib OR tasocitinib:kw (Word variations have been searched)	5,461
#11	infliximab OR remicade OR "monoclonal antibody cA2" OR "MAb cA2" OR "CT-P13" OR Remsima OR Inflectra OR adalimumab OR humira OR trudexa OR "331731-18-1" OR "D2E7 antibody" OR LU200134 OR "ABP 501" OR BI695501 OR M923 OR "PF-06410293" OR GP2017 OR "CHS-1420" OR certolizumab OR "certolizumab pegol" OR cimzia OR golimumab OR simponi OR "TNFR-Fc fusion protein" OR etanercept OR enbrel OR Avent OR BX2922 OR "CHS-0214" OR ENIA11 OR Etacept OR Etanar OR GP2013 OR GP2015 OR HD203 OR LBEC0101 OR M923 OR "PRX-106" OR SB4 OR TuNEX OR Yisaipu OR abatacept OR orencia OR anakinra OR kineret OR rituximab OR rituxan OR mabthera OR zytux OR reditux OR tocilizumab OR atlizumab OR actemra OR roactemra OR sarilumab OR sirukumab OR tofacitinib OR tasocitinib OR xeljanz OR jakvinus OR kevzara OR plivensia (Word variations have been searched)	8,062
#12	#10 OR #11	8,062
**All interventions and comparators**		
#13	#6 OR #9 OR #12	15,525
**Study type (RCTs)**		
#14	MeSH descriptor: [Randomized Controlled Trials as Topic] explode all trees	22,828
#15	MeSH descriptor: [Clinical Trials, Phase III as Topic] explode all trees	311
#16	MeSH descriptor: [Clinical Trials, Phase II as Topic] explode all trees	178

Search No.	Terms	Results
#17	MeSH descriptor: [Controlled Clinical Trials as Topic] explode all trees	23,205
#18	MeSH descriptor: [Random Allocation] explode all trees	20,636
#19	MeSH descriptor: [Clinical Trials as Topic] this term only	57,778
#20	#14 or #15 or #16 or #17 or #18 or #19	67,695
#21	"Randomized Controlled Trial" OR "Controlled Clinical Trial" OR "Clinical Trial Phase II" OR "Clinical Trial Phase III" OR "Clinical Trial Phase IV" OR "Multicenter Study":pt (Word variations have been searched)	521,085
#22	randomized or randomised or randomly:ti,ab,kw (Word variations have been searched)	624,981
#23	"randomized controlled" NEXT trial* OR "randomised controlled" NEXT trial* OR "randomized clinical" NEXT trial* OR "randomised clinical" NEXT trial* OR randomized NEXT trial* OR randomised NEXT trial* OR "random allocation" OR "double blind method" OR "single blind method" OR ((singl* OR doubl* OR treb* OR tripl*) AND (blind* OR mask*)) OR allocated NEXT random* OR random NEXT assignment* OR "open-label" NEXT trial* OR "open-label" NEXT stud* OR "open label trial" OR "non-blinded" NEXT stud* (Word variations have been searched)	723,579
#24	#20 OR #21 OR #22 OR #23	855,024
**Exclusion terms, excluding animals**		
#25	MeSH descriptor: [Animals] explode all trees	8,407
#26	MeSH descriptor: [Humans] explode all trees	1,621
#27	#25 not #26	6,786
**Exclusion terms, by study type**		
#28	Comment OR Letter OR Editorial OR "Case Reports" OR "Clinical Trial Phase I":pt (Word variations have been searched)	14,382
#29	"case study" OR "case studies" OR "case report" OR "case reports" OR "case series":ti (Word variations have been searched)	1,284
#30	#28 OR #29	15,573
**Exclusion terms, population**		
#31	MeSH descriptor: [Arthritis, Juvenile] explode all trees	219
#32	"juvenile idiopathic arthritis" OR (juvenile AND arthritis) (Word variations have been searched)	687
#33	#31 OR #32	687
**All relevant studies**		
#34	#3 AND #13 AND #24	3,508
#35	#34 NOT (#27 OR #30 OR #33)	3,266
**Limits**		
#36	#35 AND Publication date from 2016/05/01 Filters applied: Dates limited from 2016/05/01 to present	**528**

bDMARD = biologic disease-modifying antirheumatic drug; cDMARD = conventional disease-modifying antirheumatic drug;

MeSH = Medical Subject Heading; RCT = randomized, controlled trial.

Table S6 Biosis search strategy (Search Conducted on 11 December 2017)

Search No.	Terms	Results
**Population and/or patients**		
#1	su("Arthritis Rheumatoid" OR "Rheumatoid Arthritis")	66,744
#2	ti,ab,su(rheumatoid OR reumatoid) AND ti,ab,su(arthrit* OR disease*)	112,321
#3	#1 OR #2	112,321
**Intervention**		
#4	subst(baricitinib)	41
#5	ti,ab,su,subst(baricitinib OR Olumiant)	92
#6	#4 OR #5	92
**Comparator: cDMARDs**		
#7	subst(methotrexate OR sulfasalazine OR leflunomide OR hydroxychloroquine)	52,703
#8	ti,ab,su,subst(methotrexate OR MTX OR rheumatrex OR amethopterin OR "methotrexate sodium" OR rasuvo OR trexall OR otrexup OR sulfasalazine OR azulfidine OR salazopyrin OR sulazine OR sulfazine OR leflunomide OR arabloc OR arava OR lunava OR repso OR hydroxychloroquine OR plaquenil OR axemal OR dolquine OR quensyl OR quineprox)	60,760
#9	#7 OR #8	60,760
**Comparator: bDMARDs**		
#10	subst(infliximab OR adalimumab OR "certolizumab pegol" OR golimumab OR "TNFR-Fc fusion protein" OR abatacept OR anakinra OR rituximab OR tocilizumab OR atlizumab OR sarilumab OR sirukumab OR tofacitinib OR tasocitinib)	34,213
#11	ti,ab,su,subst(infliximab OR remicade OR "monoclonal antibody cA2" OR "MAb cA2" OR "CT-P13" OR Remsima OR Inflectra OR adalimumab OR humira OR trudexa OR "331731-18-1" OR "D2E7 antibody" OR LU200134 OR "ABP 501" OR BI695501 OR M923 OR "PF-06410293" OR GP2017 OR "CHS-1420" OR certolizumab OR "certolizumab pegol" OR cimzia OR golimumab OR simponi OR "TNFR-Fc fusion protein" OR etanercept OR enbrel OR Avent OR BX2922 OR "CHS-0214" OR ENIA11 OR Etacept OR Etanar OR GP2013 OR GP2015 OR HD203 OR LBEC0101 OR M923 OR "PRX-106" OR SB4 OR TuNEX OR Yisaipu OR abatacept OR orencia OR anakinra OR kineret OR rituximab OR rituxan OR mabthera OR zytux OR reditux OR tocilizumab OR atlizumab OR actemra OR roactemra OR sarilumab OR Kevzara OR sirukumab OR Plivensia OR tofacitinib OR tasocitinib OR xeljanz OR jakvinus) OR rn("331731-18-1")	41,237
#12	#10 OR #11	41,237
**All interventions and comparators**		
#13	#6 OR #9 OR #12	95,539
**Study type (RCTs)**		
#14	su("Randomized Controlled Trials" OR "Randomized Controlled Trial" OR "Clinical Trials Phase III" OR "Clinical Trials Phase II" OR "Controlled Clinical Trials" OR "Controlled Clinical Trial" OR "Random Allocation" OR "Clinical Trials" OR "Clinical Trial") OR ti,su(randomized AND trial*) OR ti,su("phase 3" AND trial) OR ti,su("phase III" AND trial*) OR ti,su("phase 2" AND trial*) OR ti,su("phase II" AND trial*)	122,361

Search No.	Terms	Results
#15	dtype,su("Randomized Controlled Trial" OR "Controlled Clinical Trial" OR "Clinical Trial Phase II" OR "Clinical Trial Phase III" OR "Clinical Trial Phase IV" OR "Multicenter Study") OR ti,su("phase IV" AND trial*) OR ti,su("phase 4" AND trial*)	6,035
#16	ti,ab(randomized OR randomised OR randomly)	463,144
#17	ti,ab,su("randomized controlled" P/0 trial* OR "randomised controlled" P/0 trial* OR "randomized clinical" P/0 trial* OR "randomised clinical" P/0 trial* OR randomized P/0 trial* OR randomised P/0 trial* OR "random allocation" OR "double blind method" OR "single blind method" OR ((singl* OR doubl* OR treb* OR tripl*) AND (blind* OR mask*)) OR allocated P/0 random* OR random P/0 assignment* OR "open-label" P/0 trial* OR "open-label" P/0 stud* OR "open label trial" OR "non-blinded" P/0 stud*)	224,591
#18	#14 OR #15 OR #16 OR #17	553,952
**Exclusion terms, excluding animals**		
#19	su(animal) NOT su(human)	8,620,434
**Exclusion terms, by study type**		
#20	dtype(Comment* OR Letter OR Editorial) OR su("Case Report" OR "Case Reports" OR "Clinical Trial Phase I") OR ti,su("phase 1" AND trial*) OR ti,su("phase I" AND trial*)	209,404
#21	ti("case study" OR "case studies" OR "case report" OR "case reports" OR "case series")	119,743
#22	#20 OR #21	327,949
**Exclusion terms, population**		
#23	su("Arthritis Juvenile" OR "Juvenile Arthritis")	4,822
#24	ti,ab,su("juvenile idiopathic arthritis") OR ti,ab,su(juvenile AND arthritis)	9,387
#25	#23 OR #24	9,387
**All relevant studies**		
#26	#3 AND #13 AND #18	1,984
#27	#26 NOT (#19 OR #22 OR #25)	1,842
#28	#27 AND Filters applied: Dates limited from 1 May 2016 to present	180

bDMARD = biologic disease-modifying antirheumatic drug; BIOSIS = BioSciences Information Service of Biological Abstracts;

cDMARD = conventional disease-modifying antirheumatic drug; RCT = randomized, controlled trial.

Table S7 Medline search strategy (Search Conducted on 11 December 2017)

Search No.	Terms	Results
**Population and/or patients**		
#1	"Arthritis, Rheumatoid"[MeSH]	103,822
#2	(rheumatoid OR reumatoid) AND (arthrit* OR disease*)	127,239
#3	#1 OR #2	138,472
**Intervention**		
#4	"baricitinib"[Supplementary Concept]	29
#5	"baricitinib"[All Fields] OR "Olumiant"[All Fields]	73
#6	#4 OR #5	73
**Comparator: cDMARDs**		
#7	"methotrexate"[Supplementary Concept] OR "sulfasalazine"[Supplementary Concept] OR "leflunomide"[Supplementary Concept] OR "hydroxychloroquine"[Supplementary Concept]	41,680
#8	"Methotrexate"[All Fields] OR "MTX"[All Fields] OR "rheumatrex"[All Fields] OR "amethopterin"[All Fields] OR "methotrexate sodium"[All Fields] OR "rasuvo"[All Fields] OR "trexall"[All Fields] OR "otrexup"[All Fields] OR "sulfasalazine"[All Fields] OR "azulfidine"[All Fields] OR "salazopyrin"[All Fields] OR "sulazine"[All Fields] OR "sulfazine"[All Fields] OR "leflunomide"[All Fields] OR "arabloc"[All Fields] OR "arava"[All Fields] OR "lunava"[All Fields] OR "repso"[All Fields] OR "hydroxychloroquine"[All Fields] OR "plaquenil"[All Fields] OR "axemal"[All Fields] OR "dolquine"[All Fields] OR "quensyl"[All Fields] OR "quineprox"[All Fields]	59,542
#9	#7 OR #8	56,424
**Comparator: bDMARDs**		
#10	"infliximab"[Supplementary Concept] OR "adalimumab"[Supplementary Concept] OR "certolizumab pegol"[Supplementary Concept] OR "golimumab"[Supplementary Concept] OR "TNFR-Fc fusion protein"[Supplementary Concept] OR "abatacept"[Supplementary Concept] OR "anakinra"[Supplementary Concept] OR "rituximab"[Supplementary Concept] OR "tocilizumab"[Supplementary Concept] OR "atlizumab"[Supplementary Concept] OR "sarilumab"[Supplementary Concept] OR "sirukumab"[Supplementary Concept] OR "tofacitinib"[Supplementary Concept] OR "tasocitinib"[Supplementary Concept]	25,980

Search No.	Terms	Results
#11	“infliximab”[All Fields] OR “remicade”[All Fields] OR “monoclonal antibody cA2”[All Fields] OR “MAb cA2”[All Fields] OR “CT-P13”[All Fields] OR “Remsima”[All Fields] OR “Inflectra”[All Fields] OR “adalimumab”[All Fields] OR “humira”[All Fields] OR trudexa[All Fields] OR “331731-18-1”[All Fields] OR “D2E7 antibody”[All Fields] OR “LU200134”[All Fields] OR “ABP 501”[All Fields] OR “BI695501”[All Fields] OR “M923”[All Fields] OR “PF-06410293”[All Fields] OR “GP2017”[All Fields] OR “CHS-1420”[All Fields] OR “certolizumab”[All Fields] OR “certolizumab pegol”[All Fields] OR “cimzia”[All Fields] OR “golimumab”[All Fields] OR “simponi”[All Fields] OR “TNFR-Fc fusion protein”[All Fields] OR “etanercept”[All Fields] OR “enbrel”[All Fields] OR Avent[All Fields] OR “BX2922”[All Fields] OR “CHS-0214”[All Fields] OR “ENIA11”[All Fields] OR “Etacept”[All Fields] OR “Etanar”[All Fields] OR “GP2013”[All Fields] OR “GP2015”[All Fields] OR “HD203”[All Fields] OR “LBEC0101”[All Fields] OR “M923”[All Fields] OR “PRX-106”[All Fields] OR “SB4”[All Fields] OR “TuNEX”[All Fields] OR “Yisaipu”[All Fields] OR “abatacept”[All Fields] OR “orencia”[All Fields] OR “anakinra”[All Fields] OR “kineret”[All Fields] OR “rituximab”[All Fields] OR “rituxan”[All Fields] OR “mabthera”[All Fields] OR “zytux”[All Fields] OR “reditux”[All Fields] OR “tocilizumab”[All Fields] OR “atlizumab”[All Fields] OR “actemra”[All Fields] OR “roactemra”[All Fields] OR “sarilumab”[All Fields] OR “Kevzara”[All Fields] OR “sirukumab”[All Fields] OR “Plivensia”[All Fields] OR “tofacitinib”[All Fields] OR “tasocitinib”[All Fields] OR “xeljanz”[All Fields] OR “jakvinus”[All Fields]	43,222
#12	#10 OR #11	43,222
**All interventions and comparators**		
#13	#6 OR #9 OR #12	97,786
**Study type (RCTs)**		
#14	“Randomized Controlled Trials as Topic”[MeSH] OR “Clinical Trials, Phase III as Topic”[MeSH] OR “Clinical Trials, Phase II as Topic”[MeSH] OR “Controlled Clinical Trials as Topic “[MeSH] OR “Random Allocation”[MeSH] OR “Clinical Trials as Topic”[MeSH:NoExp]	383,037
#15	“Randomized Controlled Trial”[Publication Type] OR “Controlled Clinical Trial”[Publication Type] OR “Clinical Trial, Phase II”[Publication Type] OR “Clinical Trial, Phase III”[Publication Type] OR “Clinical Trial, Phase IV”[Publication Type] OR “Multicenter Study”[Publication Type]	699,055
#16	“randomized”[Title/Abstract] OR “randomised”[Title/Abstract] OR “randomly”[Title/Abstract]	735,279
#17	randomized controlled trial*[Text Word] OR randomised controlled trial*[Text Word] OR randomized clinical trial*[Text Word] OR randomised clinical trial*[Text Word] OR randomized trial*[Text Word] OR randomised trial*[Text Word] OR “random allocation”[Text Word] OR “double blind method”[Text Word] OR “single blind method”[Text Word] OR ((singl*[Text Word] OR doubl*[Text Word] OR treb*[Text Word] OR tripl*[Text Word]) AND (blind*[Text Word] OR mask*[Text Word])) OR allocated random*[Text Word] OR random assignment*[Text Word] OR open-label trial*[Text Word] OR open-label stud*[Text Word] OR “open label trial”[Text Word] OR non-blinded stud*[Text Word]	735,401
#18	#14 OR #15 OR #16 OR #17	1,380,304

Search No.	Terms	Results
**Exclusion terms, excluding animals**		
#19	“Animals”[MeSH] NOT “Humans”[MeSH]	4,401,377
**Exclusion terms, by study type**		
#20	“Comment”[Publication Type] OR “Letter”[Publication Type] OR “Editorial”[Publication Type] OR “Case Reports”[Publication Type] OR “Clinical Trial, Phase I”[Publication Type]	3,268,852
#21	“case study”[Title] OR “case studies”[Title] OR “case report”[Title] OR “case reports”[Title] OR “case series”[Title]	260,831
#22	#20 OR #21	3,355,740
**Exclusion terms, population**		
#23	"Arthritis, Juvenile"[MeSH]	9,457
#24	“juvenile idiopathic arthritis”[Text word] OR (juvenile AND arthritis)	12,816
#25	#23 OR #24	12,816
**All relevant studies**		
#26	#3 AND #13 AND #18	3,799
#27	#26 NOT (#19 OR #22 OR #25)	3,423
**Limits**		
#28	#27 AND Filters applied: Dates limited from 1 May 2016 to present	**394**

bDMARD = biologic disease-modifying antirheumatic drug; cDMARD = conventional disease-modifying antirheumatic drug;

MeSH = Medical Subject Heading; RCT = randomized, controlled trial.

4. PRISMA diagram

Figure S1 PRISMA Diagram

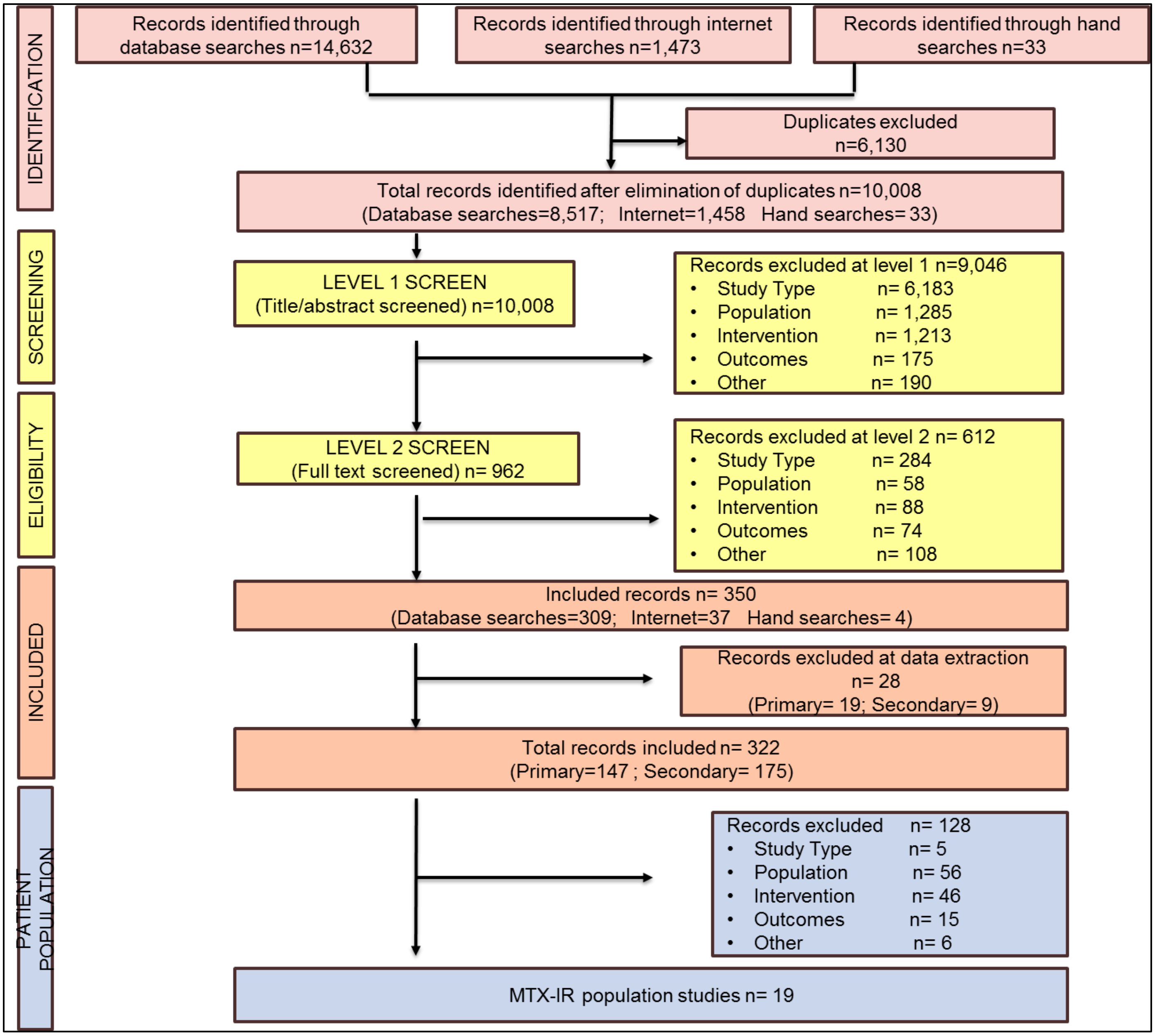

PRISMA = Preferred Reporting Items for Systematic Reviews and Meta-Analyses

5. Quality assessment of the included trials

Table S8 Summary of Quality Assessment of included Randomized Controlled Trials (primary analysis, N=19)

Trial Name (Reference)	Randomized Appropriately	Concealment of Allocation	Groups Similar for Prognostic Factors?	Were Care Providers, Participants, and Outcome Assessors Blind to Treatment Allocation?	Any Unexpected Imbalances in Drop-outs?	More Outcomes Measured Than Reported?	Did the Analysis Include an Intention-to-Treat Analysis? If so, Was This Appropriate, and Were Appropriate Methods Used to Account for Missing Data?
AIM[1]	Yes	Yes	Yes	Yes	No	No	Yes
AMPLE[2]	Unclear	Unclear	Yes	No	No	No	Yes
ARMADA[3]	Unclear	Yes	Yes	Yes	Unclear	No	No
ATTEST[4]	Unclear	Unclear	Yes	Yes	No	No	Yes
ATTRACT[5]	Unclear	Yes	Yes	Yes	Yes	No	Unclear
Edwards et al. (2004)[6]	Unclear	Unclear	Yes	Yes	Yes	No	Yes
GO-FORTH[7]	Unclear	Unclear	Yes	Unclear	No	No	Yes
GO-FORWARD[8]	Yes	Yes	Yes	Unclear	Unclear	No	Yes
Keystone et al. (2004)[9]	Unclear	Unclear	Yes	Unclear	No	No	Yes
Kim et al. (2007)[10]	Unclear	Unclear	Yes	Unclear	No	No	Yes
Li et al. (2013)[11]	Unclear	Unclear	Yes	Unclear	No	No	Yes
Machado et al. (2015)[12]	Unclear	No	Yes	No	No	No	Yes
MOBILITY[13]	Unclear	Yes	Yes	Yes	No	No	Yes
RA-SCORE[14]	Yes	Yes	Yes	Yes	Yes	No	Yes
**RA-BEAM**	Unclear	Unclear	Unclear	Yes	No	No	Unclear

Trial Name (Reference)	Randomized Appropriately	Concealment of Allocation	Groups Similar for Prognostic Factors?	Were Care Providers, Participants, and Outcome Assessors Blind to Treatment Allocation?	Any Unexpected Imbalances in Drop-outs?	More Outcomes Measured Than Reported?	Did the Analysis Include an Intention-to-Treat Analysis? If so, Was This Appropriate, and Were Appropriate Methods Used to Account for Missing Data?
RAPID-C[15]	Unclear	Unclear	Unclear	Unclear	Unclear	No	No
SERENE[16]	Unclear	Unclear	Yes	unclear	No	No	Yes
START[17]	Unclear	Unclear	Yes	Yes	No	No	Yes
Weinblatt et al. (1999)[18]	Unclear	Yes	No	Yes	Unclear	No	Unclear

6. List of trials included in the analysis

Table S9 All trials included in the analysis in MTX-IR population (N=29) - Overview

Trial name	Trial No.	Primary Analysis (Yes/No)	Sensitivity 1[a] (Yes/NA)	Sensitivity 2[b] (Yes/No)	Treatment 1	Treatments 2 / 3	Control	ACR Endpoints 24 weeks
AIM[1]	NCT00048568	Yes	NA	Yes	ABA 10mg + MTX (n=433)	---	PBO + MTX (n=219)	ACR20/50/70
AMPLE[2]	NCT00929864	Yes	NA	Yes	ABA 125mg + MTX (n=318)[c]	ADA 40mg + MTX (n=328)	---	ACR20/50/70
ARMADA[3]		Yes	NA	Yes	ADA 40mg + MTX (n=67)	---	PBO + MTX (n=62)	ACR20/50/70
ATTEST[4]	NCT00095147	Yes	NA	Yes	ABA 10mg + MTX (n=156)	IFX 3mg + MTX (n=165)	PBO + MTX (n=110)	ACR20/50/70
ATTRACT[5]		Yes	NA	Yes	IFX 3mg + MTX (n=86)	---	PBO + MTX (n=88)	ACR20
**RA-BEAM[19]**	NCT01710358	Yes	NA	Yes	BARI 4mg + MTX (n=487)	ADA 40mg + MTX (n=330)	PBO + MTX (n=488)	ACR20/50/70
Edwards (2004)[6]		Yes	NA	Yes	*RTX 1000mg (n=40)[d]*	RTX 1000mg + MTX (n=40)	PBO + MTX (n=40)	ACR20/50/70
GO-FORTH[7]	NCT00727987	Yes	NA	**No**	GOL 50mg + MTX (n=89)	---	PBO + MTX (n=90)	ACR20/50/70
GO-FORWARD[8]	NCT00264550	Yes	NA	Yes	GOL 50mg + MTX (n=89)	---	PBO + MTX (n=133)	ACR20/50/70
Keystone (2004)[9] *[DE019]*		Yes	NA	Yes	ADA 40mg + MTX (n=207)	---	PBO + MTX (n=200)	ACR20/50/70
Kim (2007)[10]		Yes	NA	**No**	ADA 40 mg + MTX (n=65)	---	PBO + MTX (n=63)	ACR20/50/70
Li (2013)[11]	NCT01248780	Yes	NA	**No**	GOL 50mg + MTX (n=132)	---	PBO + MTX (n=132)	ACR20/50/70
Machado (2014)[12] *[LARA]*[e]	NCT00848354	Yes	NA	Yes	ETN + MTX (n=284)	cDMARD + MTX (n=145)	---	ACR20/50/70
MOBILITY[13]	NCT01061736	Yes	NA	Yes	SARI 200mg + MTX (n=399)	*SARI 150mg + MTX (n=400)[d]*	PBO + MTX (n=398)	ACR20/50/70
RA-SCORE[14]	NCT00578305	Yes	NA	Yes	RTX 1000mg + MTX (n=63)	---	PBO + MTX (n=60)	ACR20/50/70
RAPID-C[15]	NCT02151851	Yes	NA	**No**	CZP + MTX (n=312)	---	PBO + MTX (n=113)	ACR20/50/70

Trial name	Trial No.	Primary Analysis (Yes/No)	Sensitivity 1[a] (Yes/NA)	Sensitivity 2[b] (Yes/No)	Treatment 1	Treatments 2 / 3	Control	ACR Endpoints 24 weeks
SERENE[16]		Yes	NA	Yes	*RTX 500mg + MTX (n=168)[d]*	RTX 1000mg + MTX (n=172)	PBO + MTX (n=172)	ACR20/50/70
START[17]		Yes	NA	Yes	IFX 3mg + MTX (n=360)	---	PBO + MTX (n=363)	ACR20/50/70
Weinblatt (1999)[18]		Yes	NA	Yes	ETN + MTX (n=59)	---	PBO + MTX (n=30)	ACR20/50/70
J-RAPID[20] *[Yamamoto 2011]*	NCT00791999	No	Yes	No	CZP + MTX (n=82)	---	PBO + MTX (n=77)	ACR20/50/70
Kang (2013)[21] *[RA0025]*		No	Yes	No	CZP + MTX (n=81)	---	PBO + MTX (n=40)	ACR20/50/70
LITHE[22]	NCT00106535	No	Yes	No	TCZ 8mg + MTX (n=398)	---	PBO + MTX (n=393)	ACR20/50/70
OPTION[23]	NCT00106548	No	Yes	No	TCZ 8mg + MTX (N=205)	---	PBO + MTX (N=204)	ACR20/50/70
ORAL SCAN[24]	NCT00847613	No	Yes	No	TOFA 5mg + MTX (n=321)	*TOFA 10mg + MTX (n=316)[d]*	PBO + MTX (n=160)	ACR20/50/70
ORAL STANDARD[25]	NCT00853385	No	Yes	No	TOFA 5mg + MTX (n=204)	*TOFA 10mg + MTX (n=201)[d]*<br>ADA 40mg + MTX (n=204)	PBO + MTX (n=108)	ACR20/50/70
ORAL STRATEGY[26]	NCT02187055	No	Yes	No	TOFA 5mg + MTX (n=376)	*TOFA 5mg (n=384)[d]*<br>ADA 40mg + MTX (n=386)	---	ACR20/50/70
RACAT[27]	NCT00405275	No	Yes	No	ETN + MTX (n=175)	SSZ + HCQ + MTX (n=178)[f]	---	ACR20/50/70
RAPID1[28]	NCT00152386	No	Yes	No	CZP + MTX (n=393)	---	PBO + MTX (n=199)	ACR20/50/70
RAPID2[29]	NCT00175877	No	Yes	No	CZP + MTX (n=246)	---	PBO + MTX (n=127)	ACR20/50/70

Abbreviations: ABA abatacept; ADA adalimumab; BARI baricitinib; CZP certolizumab; ETN eternacept; GOL golimumab; IFX infliximab; MTX methotrexate; PBO placebo; RTX rituximab; SARI sarilumab; TCZ tocilizumab; TOFA tofacitinib.

a- addition of trials that allowed for up to 20% of patients with prior bDMARD use (NA = not applicable as already part of primary analysis; Yes = added in analysis)
b- exclusion of trials solely conducted in Asia Pacific and/or low/unknown dose of MTX (<7.5 mg/week) (No = excluded from analysis; Yes = included in analysis)
c- labeled as "SUBCUT" (subcutaneous) in the analyses
d- treatment arms not included in the analysis, i.e. only presented for reasons of completeness
e- open-label trial, excluded via corresponding sensitivity analysis
f- labeled as "cDMARD + MTX" in the analyses

7. Methodology

7.1. Endpoints

The following endpoints were chosen for the analysis:

- ACR response (20%, 50%, and 70% improvement in criteria)[30]

Safety endpoints were not included as part of the NMA, as the majority of trials allowed the use of rescue therapy for the control arm if an ACR response of 20% was not observed. Hence, once patients on the control arm are allowed to be switched to the active treatment, there is no longer a common comparator for the network.

Therefore, only endpoints that are measured prior to rescue therapy (generally the 12-week outcomes) would have had a common comparator; however, in most of the publications, safety endpoints are only reported for the duration of the trial and not at intermediate endpoints.

Discontinuations were not included as part of the NMA for reasons that are also linked to rescue therapy. Discontinuation rates are included in many of the trials for ACR response, as most of the trials use an imputation method of no response for patients who have missing data. Therefore, discontinuations are already considered in the ACR response outcomes.

7.2. Overview of Analyses

NMA was conducted using Bayesian mixed treatment comparisons as described in the National Institute for Health and Care Excellence (NICE) Decision Support Unit (DSU) Technical Support Documents (TSDs)[31].

Two classes of models were assessed:[32]

i. separate models for baseline and treatment effect

ii. simultaneous modelling of baseline and treatment effects

It was decided that the simultaneous model was to be used for several reasons: a) the data for both baseline and treatment effects come from the same sources, b) there were some networks that had zero cells and fitting this type of model increased the stability of the relevant models, and c) the evidence for several networks was sparse.

In addition, for sensitivity analysis, frequentist NMA using the Rücker method was conducted to assess the robustness of the results[33]. Note for cells with zero counts the following rule was implemented: if 1 arm in trial had 0 count then 0.5 was added to all arms for that trial.

The method based on Bayesian models was first proposed in 1996 by Higgins and Whitehead[34]. The results of the different interventions from the included trials were combined by means of a Bayesian NMA using a logistic-regression model with a binomial likelihood distribution for the categorical (ACR response) outcomes.[35,36]

As with any meta-analysis, NMA can be performed with a fixed-effects approach or a random-effects approach[35,37]. With a fixed-effects model, it is assumed that differences in true relative treatment effects (whether estimated directly or indirectly) are only caused by the difference in treatment and no other factors. There is no heterogeneity in true relative treatment effects beyond differences in treatment effect caused by the differences in type of interventions compared. With a random-effects assumption, differences in trial-specific response rates (beyond the differences attributable to the actual interventions compared) are exchangeable and the heterogeneity is constant between the different comparisons. The choice for a fixed or

random-effects model for the final analysis was evaluated on the basis of model fit (as measured by Deviance Information Criterion [DIC]), sensitivity of results, and assessment of residual deviance[35,37].

For assessment of between-trial variance used in the random-effects model, NICE[38,39] recommend vague priors for the between trial variances [σ ~ Uniform (0, 2)]. The Agency for Healthcare Research and Quality (AHRQ)[40] recommend a similar approach with an upper limit of 5. The upper limits reflect a between-trial variability that allows for a wide range of treatment effects. For the present analysis, for binary models, priors were tested at σ Uniform (0, σ^2), where σ=5. Appropriate priors for trial variance were assessed on a case-by-case basis. Baseline between trial σ was set at Uniform (0, 2). It might be that the random-effects models were sensitive to the choice of vague priors resulting in unstable (i.e., wide) credible intervals (CrIs). This could be due to the combination of a low number of included trials, trials with small sample size, and a high degree of heterogeneity. In that case, informative priors [Log normal (-2.29, 1.58)] were to be used instead.[41]

The initial model runs used 3 chains, with a burn-in of 10,000 simulations, and estimating the posterior probabilities from a sample of further 10,000 simulations. In order to address insufficient convergence and auto-correlation, this was increased to 4 chains, a burn-in of 60,000 and a sample of 120,000.

Bayesian NMA analyses were performed in JAGS via R version 3.4.2[42] using the R2JAGS package, and Frequentist NMA analysis was run using R version 3.4.2 using the netmeta package.[33,43]

7.3. Bayesian Mixed Treatment Comparisons

Binary Endpoint Model for Network Meta-Analysis:

For the primary analysis we modelled the proportion of patients who experienced an ACR response (20%, 50%, and 70%). This follows a binary endpoint model where the underlying model is that of a logistic regression. Observed data were included in the model using a binomial likelihood where the probability (p) of response for study i and treatment k is as follows:

$$r_{i,k} \sim Binomial(p_{i,k}, n_{i,k})$$

Where $r_{i,k}$ is the number of events in treatment arm k of study i, and $n_{i,k}$ is the total number of subjects in treatment arm k of study$i$.

Treatments k included in the FE model were indexed as positive integers with the baseline treatment (b) being the lowest index treatment in study i. A logit link function was used to map the probability of response to the linear model such that for treatment arm k of study:

$$\text{logit}(p_{i,k}) = \alpha_i + (\beta_{i,k} - \beta_{i,b})$$

Where α_i is the study-specific baseline term, and $\beta_{i,k} - \beta_{i,b}$ is the study-specific log odds ratio of treatment k compared to baseline b. For study arms receiving the baseline treatment (ie, $k = b$), this simplifies to the study-specific baseline term α_i. A vague prior $\beta \sim N(0, 100^2)$ was used for the treatment effect coefficients.

The corresponding RE model replaces the constant treatment effect with the study-specific treatment effect $\delta_{i,k}$. This is normally distributed with mean $mb_{i,k} = (\beta_{i,k} - \beta_{i,b})$ and variance

σ^2, where σ^2 is the RE variance and assumed to be constant across all treatment comparisons. (Note that this model was be equivalent to a FE model when $\sigma^2 = 0$). The following changes were made for the RE model:

$$\text{logit}(p_{i,k}) = \alpha_i + \delta_{i,k}$$

$$\delta_{i,k} \sim N(mb_{i,k}, \sigma^2)$$

$$mb_{i,k} = (\beta_{i,k} - \beta_{i,b})$$

The parameters of interest modelled were the log odds ratios (β) which provide the relative treatment effect for each treatment compared to the reference treatment in the analyses. Estimates of these parameters were iteratively sampled using Bayesian methods (as described in the heading below). The parameter value can be summarised by calculating the mean and standard error of these samples (i.e., mean log odds ratio and corresponding standard error which can be converted to odds ratios). In addition, the CrIs can be estimated from these samples. These are similar to confidence intervals (CIs) in a Frequentist analysis; however, the interpretation differs as described below for a 5% significance level:

Frequentist 95% CIs: 95% probability that the true value lies within 95% of these intervals in the long run, if many samples were taken of the data

Bayesian 95% CrI: 95% probability that the true value of the parameter lies within the interval

The 95% CrI in Bayesian analyses are the values corresponding to the lower 2.5 and upper 97.5 percentiles of samples taken for each parameter modelled.

Treatment effects are presented as odds ratios with associated 95% CrIs. If not noted otherwise, the reference treatment is placebo + methotrexate (PBO + MTX). For other estimates of interest, medians and associated 95% CrIs are reported.

Zero Cells

Bayesian models with zero cells do not usually require special precautions (refer to [39]). However, in the case of the frequentist models (for the analysis of ACR70), we had to add the continuity correction of 0.5 to 0 cells for them to converge. This is known to generate biased estimates of effect size so these results should be interpreted with precaution.[44,45]

Choice of the treatment effect: random vs fixed effects

As with any MA, NMA can be performed with a fixed effects approach or a random effects approach[35,36]. In Bayesian NMA, it is assumed that differences in trial-specific relative treatment effect (beyond the differences attributable to the actual interventions compared) are exchangeable and the heterogeneity is constant between the different comparisons. Initially, both fixed and random effects models were performed for each endpoint.

For Bayesian NMA the choice for a fixed or random effects model for the primary analysis was evaluated on:

- The model fit as measured by Deviance Information Criterion (DIC),
- Assessment of the residual deviance,
- Convergence of the models,
- Sensitivity of the results,

- Whether there is limited data to inform the random effects variance,
- Whether there is evidence of the random-effects prior dominating the posterior simulations indicating that there is not enough data in the analysis to inform this additional parameter.

Inconsistency, Model Fit, and Convergence

It was pre-planned to explore the consistency assumption via the “node-splitting” approach as defined by Dias[46]. However, networks were primarily star-shaped, with only 2 closed loops. Both loops were informed by one trial, respectively. Therefore, this analysis was not performed.

Model fit was assessed with the DIC and the posterior mean of the total residual deviance.[47] Deviance measures the fit of the model to the data using the likelihood function. A good model fit is indicated by a total residual deviance approximately equal to the number of data points available. The DIC is a statistic that measures Bayesian model fit and penalizes the deviance by the model complexity. When comparing 2 DIC values, a difference of 5 or more is regarded as a meaningful difference.[48]

Convergence was verified by trace plots, monitoring the Monte Carlo error, and with Gelman-Rubin diagnostics.[49]

7.4. Sensitivity Analyses

In addition to the analyses described above, the following sensitivity analyses were pre-planned and performed:

Baseline-risk adjustment

In order to account for potential heterogeneity amongst ACR response rates in the PBO + MTX treatment arms, meta-regression models adjusting for baseline-risk were performed.[38,50]

Inclusion / Removal of Trial Types

Additional sensitivity analyses were conducted, which included or removed specific trial types, to investigate the potential impact of treatment effect modifiers. These were:

- Inclusion of trials with prior bDMARD use in up to 20% of patients
- Removal of trials solely conducted in Asian-Pacific; or low (<7.5 mg/week) / unknown dose of methotrexate
- Removal of open-label trials.

Table S10 provides an overview of all pre-planned sensitivity analyses.

Table S10 Overview of pre-planned sensitivity analyses

No.	Description	Comments
0	Primary model<br>(Simultaneous Fixed-effects / Random-effects models)	**Fixed-effects** model chosen as main analysis approach<br>Random-effects model presented as supplementary information
0a	**Baseline-risk** adjustment (for primary model)<br>(Simultaneous Fixed-effects / Random-effects models)	**Fixed-effects** model as main analysis approach
1	Inclusion of trials that allowed for up to 20% of patients with prior bDMARD use	List of trials included: J-RAPID, Kang (2013), RAPID1, RAPID2, LITHE, OPTION, ORAL SCAN, ORAL STANDARD, ORAL STRATEGY, RACAT<br>Note: this increased the number of CZP trials in the networks from 1 (primary model) to 5.<br>Note: These were the only networks that included TOFA and TCZ.<br>**Fixed-effects** model as main analysis approach
2	Removal of trials that were only conducted in Asian-Pacific countries and/or trials with previous low (<7.5 mg/week) or unknown dose MTX	List of trials excluded: Kim (2007), GO-FORTH, Li (2013), RAPID-C<br>Note: As a result, CZP was no longer part of the network.<br>**Fixed-effects** model as main analysis approach
3	Removal of open-label trials	List of trials excluded: Machado (2014)<br>**Fixed-effects** model as main analysis approach
Additional sensitivity analyses		
--	**Independent baseline** model (for primary model)<br>(Fixed-effects / Random-effects models)	---
--	**Frequentist NMA** (for primary model)<br>(Fixed-effects / Random-effects models)	---
Sensitivity analyses that were pre-planned but not performed in the context of this manuscript		

No.	Description	Comments
4	Removal of trials due to inconsistency (node splitting)	Not performed as only 2 closed loops coming from single trials, respectively (RA-BEAM, ATTEST)

8. ACR Response Data

Table S11 Percentage of patients achieving ACR20/50/70 response per trial and treatment arm: MTX-IR population

Trial	Treatment arm (as labelled in NMA)	Time-point (week)	Numbers analyzed	% achieving ACR20 response	% achieving ACR50 response	% achieving ACR70 response	Data used in primary model?	Exclusion reason
AIM[1]	ABA 10mg + MTX	24	424	67.9	39.9	19.8	Y	
AIM[1]	PBO + MTX	24	214	39.7	16.8	6.5	Y	
AMPLE[2]	ABA SUBCUT + MTX	24	318	65.7	40.6	21.7	Y	
AMPLE[2]	ADA 40mg + MTX	24	328	65.5	40.5	22.6	Y	
ARMADA[3]	ADA 40mg + MTX	24	67	67.2	55.2	26.9	Y	
ARMADA[3]	PBO + MTX	24	62	14.5	8.1	4.8	Y	
ATTEST[4]	ABA 10mg + MTX	28	156	66.7	40.4	20.5	Y	
ATTEST[4]	IFX 3mg + MTX	28	165	59.4	37	24.2	Y	
ATTEST[4]	PBO + MTX	28	110	41.8	20	9.1	Y	
ATTRACT[5]	IFX 3mg + MTX	26	86	48.8	-	-	Y	
ATTRACT[5]	PBO + MTX	26	88	20.5	-	-	Y	
**RA-BEAM[19]**	ADA 40mg + MTX	24	330	66.4	45.5	21.8	Y	
**RA-BEAM[19]**	BARI 4mg + MTX	24	487	73.9	50.5	29.8	Y	
**RA-BEAM[19]**	PBO + MTX	24	488	36.7	19.3	8	Y	
Edwards (2004)[6]	RTX 1000mg + MTX	24	40	72.5	42.5	22.5	Y	
Edwards (2004)[6]	PBO + MTX	24	40	37.5	12.5	5	Y	
GO-FORTH[b7]	GOL 50mg + MTX	24	86	70.9	41.9	26.7	Y	
GO-FORTH[b7]	PBO + MTX	24	88	33	14.8	5.7	Y	
GO-FORWARD[8]	GOL 50mg + MTX	24	89	59.6	37.1	20.2	Y	
GO-FORWARD[8]	PBO + MTX	24	133	27.8	13.5	5.3	Y	
Keystone (2004)[9]	ADA 40mg + MTX	24	207	63.3	39.1	20.8	Y	
Keystone (2004)[9]	PBO + MTX	24	200	29.5	9.5	2.5	Y	
Kim (2007)[b10]	ADA 40mg + MTX	24	65	61.5	43.1	21.5	Y	
Kim (2007)[b10]	PBO + MTX	24	63	36.5	14.3	7.9	Y	
Li (2013)[b11]	GOL 50mg + MTX	24	132	42.4	18.9	6.1	Y	
Li (2013)[b11]	PBO + MTX	24	132	15.9	6.8	1.5	Y	
Machado (2014)[c12]	ETN + MTX	24	279	83.2	62	34.8	Y	
Machado (2014)[c12]	cDMARD + MTX	24	142	50	23.2	11.3	Y	
MOBILITY (2015)[13]	SARI 200mg + MTX	24	399	66.4	46	25	Y	
MOBILITY (2015)[13]	PBO + MTX	24	398	33.4	17	7	Y	
RA-SCORE[14]	RTX 1000mg + MTX	24	60	51.7	26.7	8.3	Y	
RA-SCORE[14]	PBO + MTX	24	63	28.6	11.1	1.6	Y	

Trial	Treatment arm (as labelled in NMA)	Time-point (week)	Numbers analyzed	% achieving ACR20 response	% achieving ACR50 response	% achieving ACR70 response	Data used in primary model?	Exclusion reason
RAPID-C[b15]	CZP 200 mg Q2W + MTX	24	312	54.8	36.5	16.7	Y	
RAPID-C[b15]	PBO + MTX	24	113	23.9	7.1	2.7	Y	
SERENE[16]	RTX 1000mg + MTX	24	170	50.6	25.9	10	Y	
SERENE[16]	PBO + MTX	24	172	23.3	9.3	5.2	Y	
START[17]	IFX 3mg + MTX	22	343	58	32.1	14	Y	
START[17]	PBO + MTX	22	340	25.6	9.7	4.7	Y	
Weinblatt (1999)[18]	ETN + MTX	24	59	71.2	39	15.3	Y	
Weinblatt (1999)[18]	PBO + MTX	24	30	26.7	3.3	0	Y	
J-RAPID[20]	CZP + MTX	24	82	73.2	54.9	29.3	N	Up to 20% of prior
J-RAPID[20]	PBO + MTX	24	77	24.7	16.9	1.3	N	bDMARD use
Kang (2013)[21]	CZP + MTX	24	81	66.7	43.2	17.3	N	Up to 20% of prior
Kang (2013)[21]	PBO + MTX	24	40	27.5	20	2.5	N	bDMARD use
LITHE[22]	TCZ 8mg + MTX	24	398	56.5	32.2	12.3	N	Up to 20% of prior
LITHE[22]	PBO + MTX	24	393	27.7	10.2	2	N	bDMARD use
OPTION[23]	TCZ 8mg + MTX	24	205	58.5	43.9	22	N	Up to 20% of prior
OPTION[23]	PBO + MTX	24	204	26.5	10.8	2	N	bDMARD use
ORAL SCAN[24]	TOFA 5mg + MTX	26	321	51.4	32.4	14.6	N	Up to 20% of prior
ORAL SCAN[24]	PBO + MTX	26	79	25.3	8.9	1.3	N	bDMARD use
ORAL STANDARD[25]	TOFA 5mg + MTX	24	204	51.5	39.2	19.6	N	
ORAL STANDARD[25]	ADA 40mg + MTX	24	204	47.1	28.9	8.8	N	Up to 20% of prior bDMARD use
ORAL STANDARD[25]	PBO + MTX	24	108	28.7	12	1.9	N	
ORAL STRATEGY[26]	TOFA 5mg + MTX	24	376	73.1	46	25	N	Up to 20% of prior
ORAL STRATEGY[26]	ADA 40mg + MTX	24	386	71	43.8	20.7	N	bDMARD use
RACAT[27]	ETN + MTX	24	163	55.2	35.6	16	N	Up to 20% of prior
RACAT[27]	SSZ + HCQ + MTX[d]	24	159	56	25.8	5	N	bDMARD use
RAPID1[28]	CZP+ MTX	24	393	58.8	37.2	21.4	N	Up to 20% of prior
RAPID1[28]	PBO + MTX	24	199	13.6	7.5	3	N	bDMARD use
RAPID2[29]	CZP + MTX	24	246	57.3	32.5	15.9	N	Up to 20% of prior
RAPID2[29]	PBO + MTX	24	127	8.7	3.1	0.8	N	bDMARD use

a- Percentages are based on reported patient numbers.

b- Trial solely in Asia Pacific and/or with low (<7.5 mg/week) or unknown MTX dose

c- Open-label trial

d- Re-labeled to "cDMARD+MTX" in the analysis

9. Results – Primary Analyses

9.1. Primary analyses (Simultaneous Fixed – effects)

9.1.1. ACR20 Response

Table S12 Primary Analysis: Relative treatment effect of pairwise comparisons expressed as Post. Median odds ratios (with 95% CrIs) - ACR20 response at week 24: MTX-IR (Simultaneous Fixed-effects model)

Treatment 1	Treatment 2	Median OR	95% CrI Lower	95% CrI Upper
PBO + MTX	PBO + MTX	1	1	1
**BARI 4mg + MTX[1]**	**PBO + MTX**	**5.31513**	**4.10118**	**6.88964**
ADA 40mg + MTX	PBO + MTX	3.99938	3.26199	4.89624
ABA 10mg + MTX	PBO + MTX	3.64788	2.80663	4.74853
ABA SUBCUT + MTX	PBO + MTX	4.10476	2.85834	5.90041
IFX 3mg + MTX	PBO + MTX	3.25395	2.59896	4.10315
RTX 1000mg + MTX	PBO + MTX	3.25348	2.33861	4.5455
GOL 50mg + MTX	PBO + MTX	3.72863	2.72952	5.12315
cDMARD + MTX	PBO + MTX	1.93528	1.0355	3.52118
ETN + MTX	PBO + MTX	9.05622	5.28742	15.20698
SARI 200mg + MTX	PBO + MTX	4.13151	2.96458	5.7811
CZP + MTX	PBO + MTX	3.4882	2.3303	5.32786
PBO + MTX	BARI 4mg + MTX	0.18814	0.14515	0.24383
BARI 4mg + MTX	BARI 4mg + MTX	1	1	1
ADA 40mg + MTX	BARI 4mg + MTX	0.75223	0.57099	0.99305
ABA 10mg + MTX	BARI 4mg + MTX	0.68734	0.4771	0.98922
ABA SUBCUT + MTX	BARI 4mg + MTX	0.77142	0.51096	1.1677
IFX 3mg + MTX	BARI 4mg + MTX	0.61342	0.43467	0.86131
RTX 1000mg + MTX	BARI 4mg + MTX	0.61167	0.40606	0.93022
GOL 50mg + MTX	BARI 4mg + MTX	0.70191	0.46766	1.05847
cDMARD + MTX	BARI 4mg + MTX	0.36436	0.18805	0.6926
ETN + MTX	BARI 4mg + MTX	1.69883	0.95195	3.01826
SARI 200mg + MTX	BARI 4mg + MTX	0.77925	0.51198	1.18559
CZP + MTX	BARI 4mg + MTX	0.6551	0.40724	1.08158
PBO + MTX	ADA 40mg + MTX	0.25004	0.20424	0.30656
**BARI 4mg + MTX[1]**	**ADA 40mg + MTX**	**1.32938**	**1.007**	**1.75135**
ADA 40mg + MTX	ADA 40mg + MTX	1	1	1
ABA 10mg + MTX	ADA 40mg + MTX	0.91324	0.66109	1.26509
ABA SUBCUT + MTX	ADA 40mg + MTX	1.02651	0.74847	1.40938
IFX 3mg + MTX	ADA 40mg + MTX	0.81414	0.60565	1.10206
RTX 1000mg + MTX	ADA 40mg + MTX	0.81317	0.55808	1.19803
GOL 50mg + MTX	ADA 40mg + MTX	0.93203	0.64804	1.357
cDMARD + MTX	ADA 40mg + MTX	0.48456	0.25382	0.90248
ETN + MTX	ADA 40mg + MTX	2.25902	1.29139	3.90528
SARI 200mg + MTX	ADA 40mg + MTX	1.03437	0.70072	1.52599
CZP + MTX	ADA 40mg + MTX	0.87196	0.55973	1.39421
PBO + MTX	ABA 10mg + MTX	0.27413	0.21059	0.3563
**BARI 4mg + MTX[1]**	**ABA 10mg + MTX**	**1.45488**	**1.0109**	**2.09601**

Treatment 1	Treatment 2	Median OR	95% CrI Lower	95% CrI Upper
ADA 40mg + MTX	ABA 10mg + MTX	1.095	0.79046	1.51266
ABA 10mg + MTX	ABA 10mg + MTX	1	1	1
ABA SUBCUT + MTX	ABA 10mg + MTX	1.12305	0.72442	1.75091
IFX 3mg + MTX	ABA 10mg + MTX	0.89207	0.6523	1.2205
RTX 1000mg + MTX	ABA 10mg + MTX	0.88997	0.58999	1.36309
GOL 50mg + MTX	ABA 10mg + MTX	1.01978	0.68122	1.54776
cDMARD + MTX	ABA 10mg + MTX	0.52904	0.27401	1.01391
ETN + MTX	ABA 10mg + MTX	2.4704	1.38273	4.41892
SARI 200mg + MTX	ABA 10mg + MTX	1.13008	0.74836	1.72845
CZP + MTX	ABA 10mg + MTX	0.95498	0.59141	1.58038
PBO + MTX	ABA SUBCUT + MTX	0.24362	0.16948	0.34985
**BARI 4mg + MTX**	**ABA SUBCUT + MTX**	**1.29632**	**0.85638**	**1.95709**
ADA 40mg + MTX	ABA SUBCUT + MTX	0.97417	0.70953	1.33605
ABA 10mg + MTX	ABA SUBCUT + MTX	0.89043	0.57113	1.38042
ABA SUBCUT + MTX	ABA SUBCUT + MTX	1	1	1
IFX 3mg + MTX	ABA SUBCUT + MTX	0.79279	0.52366	1.21615
RTX 1000mg + MTX	ABA SUBCUT + MTX	0.79219	0.4904	1.29134
GOL 50mg + MTX	ABA SUBCUT + MTX	0.90915	0.56591	1.46675
cDMARD + MTX	ABA SUBCUT + MTX	0.47172	0.233	0.92921
ETN + MTX	ABA SUBCUT + MTX	2.20036	1.17492	4.10811
SARI 200mg + MTX	ABA SUBCUT + MTX	1.00786	0.61651	1.64583
CZP + MTX	ABA SUBCUT + MTX	0.85046	0.49833	1.48442
PBO + MTX	IFX 3mg + MTX	0.30732	0.24372	0.38477
**BARI 4mg + MTX[1]**	**IFX 3mg + MTX**	**1.63021**	**1.16103**	**2.30061**
ADA 40mg + MTX	IFX 3mg + MTX	1.22828	0.90739	1.65111
ABA 10mg + MTX	IFX 3mg + MTX	1.12099	0.81934	1.53305
ABA SUBCUT + MTX	IFX 3mg + MTX	1.26136	0.82227	1.90965
IFX 3mg + MTX	IFX 3mg + MTX	1	1	1
RTX 1000mg + MTX	IFX 3mg + MTX	0.99887	0.67272	1.48745
GOL 50mg + MTX	IFX 3mg + MTX	1.14381	0.78602	1.68279
cDMARD + MTX	IFX 3mg + MTX	0.59304	0.30658	1.12125
ETN + MTX	IFX 3mg + MTX	2.77433	1.55796	4.85694
SARI 200mg + MTX	IFX 3mg + MTX	1.26891	0.84668	1.90011
CZP + MTX	IFX 3mg + MTX	1.07259	0.67345	1.724
PBO + MTX	RTX 1000mg + MTX	0.30736	0.22	0.4276
**BARI 4mg + MTX[1]**	**RTX 1000mg + MTX**	**1.63486**	**1.07501**	**2.46269**
ADA 40mg + MTX	RTX 1000mg + MTX	1.22976	0.8347	1.79186
ABA 10mg + MTX	RTX 1000mg + MTX	1.12363	0.73363	1.69495
ABA SUBCUT + MTX	RTX 1000mg + MTX	1.26232	0.77439	2.03915
IFX 3mg + MTX	RTX 1000mg + MTX	1.00113	0.67229	1.48649
RTX 1000mg + MTX	RTX 1000mg + MTX	1	1	1
GOL 50mg + MTX	RTX 1000mg + MTX	1.14702	0.73332	1.7886
cDMARD + MTX	RTX 1000mg + MTX	0.59402	0.29507	1.16175
ETN + MTX	RTX 1000mg + MTX	2.78234	1.48963	5.08647
SARI 200mg + MTX	RTX 1000mg + MTX	1.27187	0.79162	2.03145
CZP + MTX	RTX 1000mg + MTX	1.07302	0.64179	1.81831
PBO + MTX	GOL 50mg + MTX	0.26819	0.19519	0.36637
**BARI 4mg + MTX**	**GOL 50mg + MTX**	**1.42469**	**0.94476**	**2.1383**
ADA 40mg + MTX	GOL 50mg + MTX	1.07293	0.73692	1.54312
ABA 10mg + MTX	GOL 50mg + MTX	0.9806	0.64609	1.46796
ABA SUBCUT + MTX	GOL 50mg + MTX	1.09993	0.68178	1.76706
IFX 3mg + MTX	GOL 50mg + MTX	0.87427	0.59425	1.27224
RTX 1000mg + MTX	GOL 50mg + MTX	0.87183	0.5591	1.36366
GOL 50mg + MTX	GOL 50mg + MTX	1	1	1
cDMARD + MTX	GOL 50mg + MTX	0.52203	0.25771	1.00645
ETN + MTX	GOL 50mg + MTX	2.433	1.30033	4.35891

Treatment 1	Treatment 2	Median OR	95% CrI Lower	95% CrI Upper
SARI 200mg + MTX	GOL 50mg + MTX	1.1101	0.69897	1.74907
CZP + MTX	GOL 50mg + MTX	0.93724	0.56342	1.55329
PBO + MTX	cDMARD + MTX	0.51672	0.284	0.96572
**BARI 4mg + MTX[1]**	**cDMARD + MTX**	**2.74452**	**1.44383**	**5.31782**
ADA 40mg + MTX	cDMARD + MTX	2.06371	1.10805	3.93973
ABA 10mg + MTX	cDMARD + MTX	1.89021	0.98628	3.64951
ABA SUBCUT + MTX	cDMARD + MTX	2.1199	1.07618	4.29185
IFX 3mg + MTX	cDMARD + MTX	1.68621	0.89186	3.26179
RTX 1000mg + MTX	cDMARD + MTX	1.68345	0.86077	3.38899
GOL 50mg + MTX	cDMARD + MTX	1.9156	0.99359	3.88032
cDMARD + MTX	cDMARD + MTX	1	1	1
ETN + MTX	cDMARD + MTX	4.66695	2.98845	7.30374
SARI 200mg + MTX	cDMARD + MTX	2.13635	1.08364	4.30617
CZP + MTX	cDMARD + MTX	1.80017	0.8896	3.86891
PBO + MTX	ETN + MTX	0.11042	0.06576	0.18913
**BARI 4mg + MTX**	**ETN + MTX**	**0.58864**	**0.33132**	**1.05047**
ADA 40mg + MTX	ETN + MTX	0.44267	0.25606	0.77436
ABA 10mg + MTX	ETN + MTX	0.40479	0.2263	0.72321
ABA SUBCUT + MTX	ETN + MTX	0.45447	0.24342	0.85112
IFX 3mg + MTX	ETN + MTX	0.36045	0.20589	0.64186
RTX 1000mg + MTX	ETN + MTX	0.35941	0.1966	0.67131
GOL 50mg + MTX	ETN + MTX	0.41102	0.22942	0.76904
cDMARD + MTX	ETN + MTX	0.21427	0.13692	0.33462
ETN + MTX	ETN + MTX	1	1	1
SARI 200mg + MTX	ETN + MTX	0.45792	0.24747	0.85566
CZP + MTX	ETN + MTX	0.38631	0.20455	0.76456
PBO + MTX	SARI 200mg + MTX	0.24204	0.17298	0.33732
**BARI 4mg + MTX**	**SARI 200mg + MTX**	**1.28328**	**0.84346**	**1.9532**
ADA 40mg + MTX	SARI 200mg + MTX	0.96677	0.65531	1.4271
ABA 10mg + MTX	SARI 200mg + MTX	0.88489	0.57855	1.33626
ABA SUBCUT + MTX	SARI 200mg + MTX	0.9922	0.6076	1.62204
IFX 3mg + MTX	SARI 200mg + MTX	0.78808	0.52628	1.18108
RTX 1000mg + MTX	SARI 200mg + MTX	0.78624	0.49226	1.26323
GOL 50mg + MTX	SARI 200mg + MTX	0.90082	0.57173	1.43067
cDMARD + MTX	SARI 200mg + MTX	0.46809	0.23223	0.92282
ETN + MTX	SARI 200mg + MTX	2.18378	1.16869	4.04082
SARI 200mg + MTX	SARI 200mg + MTX	1	1	1
CZP + MTX	SARI 200mg + MTX	0.84456	0.49834	1.45005
PBO + MTX	CZP + MTX	0.28668	0.18769	0.42913
**BARI 4mg + MTX**	**CZP + MTX**	**1.52648**	**0.92458**	**2.45553**
ADA 40mg + MTX	CZP + MTX	1.14684	0.71725	1.78657
ABA 10mg + MTX	CZP + MTX	1.04715	0.63276	1.69087
ABA SUBCUT + MTX	CZP + MTX	1.17583	0.67366	2.0067
IFX 3mg + MTX	CZP + MTX	0.93232	0.58005	1.48488
RTX 1000mg + MTX	CZP + MTX	0.93195	0.54996	1.55814
GOL 50mg + MTX	CZP + MTX	1.06697	0.64379	1.77489
cDMARD + MTX	CZP + MTX	0.5555	0.25847	1.12411
ETN + MTX	CZP + MTX	2.5886	1.30794	4.88878
SARI 200mg + MTX	CZP + MTX	1.18405	0.68963	2.00664
CZP + MTX	CZP + MTX	1	1	1

Abbreviations: ABA abatacept; ADA adalimumab; BARI baricitinib; ETN etanercept; GOL golimumab; IFX infliximab; MTX methotrexate; PBO placebo; RTX rituximab; SARI sarilumab; SUBCUT subcutaneous.

[1]statistically significantly favouring BARI.

Odds ratios >1 are in favour of Treatment 1; and odds ratios <1 are in favour of Treatment 2.

9.1.2. *ACR50 Response*

Table S13 Primary Analysis: Relative treatment effect of pairwise comparisons expressed as post. Median odds ratios (with 95% CrIs) - ACR50 response at week 2 - MTX-IR (Simultaneous Fixed-effects model)

Treatment 1	Treatment 2	Median OR	95% CrI Lower	95% CrI Upper
PBO + MTX	PBO + MTX	1	1	1
**BARI 4mg + MTX[1]**	**PBO + MTX**	**4.98588**	**3.82327**	**6.51445**
ADA 40mg + MTX	PBO + MTX	4.56728	3.6222	5.77669
ABA 10mg + MTX	PBO + MTX	3.52577	2.62551	4.78608
ABA SUBCUT + MTX	PBO + MTX	4.55585	3.10209	6.68819
IFX 3mg + MTX	PBO + MTX	3.49819	2.60941	4.74666
RTX 1000mg + MTX	PBO + MTX	3.28002	2.17601	5.04489
GOL 50mg + MTX	PBO + MTX	3.45909	2.36829	5.09424
cDMARD + MTX	PBO + MTX	1.50771	0.69075	3.45121
ETN + MTX	PBO + MTX	8.02507	4.10227	16.71772
SARI 200mg + MTX	PBO + MTX	4.37452	3.0949	6.22084
CZP + MTX	PBO + MTX	5.84551	3.34592	11.10324
PBO + MTX	BARI 4mg + MTX	0.20057	0.1535	0.26156
BARI 4mg + MTX	BARI 4mg + MTX	1	1	1
ADA 40mg + MTX	BARI 4mg + MTX	0.91622	0.70662	1.18552
ABA 10mg + MTX	BARI 4mg + MTX	0.70865	0.47453	1.05679
ABA SUBCUT + MTX	BARI 4mg + MTX	0.91438	0.61114	1.36788
IFX 3mg + MTX	BARI 4mg + MTX	0.70185	0.47317	1.04883
RTX 1000mg + MTX	BARI 4mg + MTX	0.65871	0.4046	1.08651
GOL 50mg + MTX	BARI 4mg + MTX	0.69426	0.44025	1.10781
cDMARD + MTX	BARI 4mg + MTX	0.30259	0.13321	0.71691
ETN + MTX	BARI 4mg + MTX	1.60929	0.79352	3.48598
SARI 200mg + MTX	BARI 4mg + MTX	0.8764	0.56844	1.35781
CZP + MTX	BARI 4mg + MTX	1.17438	0.63505	2.35272
PBO + MTX	ADA 40mg + MTX	0.21895	0.17311	0.27608
**BARI 4mg + MTX**	**ADA 40mg + MTX**	**1.09144**	**0.84351**	**1.41519**
ADA 40mg + MTX	ADA 40mg + MTX	1	1	1
ABA 10mg + MTX	ADA 40mg + MTX	0.77398	0.53107	1.12283
ABA SUBCUT + MTX	ADA 40mg + MTX	0.99869	0.72952	1.36212
IFX 3mg + MTX	ADA 40mg + MTX	0.76627	0.52942	1.11585
RTX 1000mg + MTX	ADA 40mg + MTX	0.71845	0.45229	1.15659
GOL 50mg + MTX	ADA 40mg + MTX	0.75677	0.49116	1.17391
cDMARD + MTX	ADA 40mg + MTX	0.33053	0.14804	0.77261
ETN + MTX	ADA 40mg + MTX	1.75643	0.88746	3.74289
SARI 200mg + MTX	ADA 40mg + MTX	0.95709	0.63289	1.45234
CZP + MTX	ADA 40mg + MTX	1.28005	0.70451	2.51899
PBO + MTX	ABA 10mg + MTX	0.28363	0.20894	0.38088
**BARI 4mg + MTX**	**ABA 10mg + MTX**	**1.41113**	**0.94626**	**2.10735**
ADA 40mg + MTX	ABA 10mg + MTX	1.29202	0.89061	1.88299
ABA 10mg + MTX	ABA 10mg + MTX	1	1	1
ABA SUBCUT + MTX	ABA 10mg + MTX	1.29027	0.79544	2.09266
IFX 3mg + MTX	ABA 10mg + MTX	0.99104	0.70279	1.40793
RTX 1000mg + MTX	ABA 10mg + MTX	0.92952	0.56075	1.55871

Treatment 1	Treatment 2	Median OR	95% CrI Lower	95% CrI Upper
GOL 50mg + MTX	ABA 10mg + MTX	0.97973	0.60562	1.59262
cDMARD + MTX	ABA 10mg + MTX	0.42631	0.18692	1.02712
ETN + MTX	ABA 10mg + MTX	2.27385	1.10589	5.00869
SARI 200mg + MTX	ABA 10mg + MTX	1.23956	0.78383	1.95802
CZP + MTX	ABA 10mg + MTX	1.65984	0.87975	3.35988
PBO + MTX	ABA SUBCUT + MTX	0.2195	0.14952	0.32236
**BARI 4mg + MTX**	**ABA SUBCUT + MTX**	**1.09364**	**0.73106**	**1.63628**
ADA 40mg + MTX	ABA SUBCUT + MTX	1.00131	0.73415	1.37077
ABA 10mg + MTX	ABA SUBCUT + MTX	0.77503	0.47786	1.25716
ABA SUBCUT + MTX	ABA SUBCUT + MTX	1	1	1
IFX 3mg + MTX	ABA SUBCUT + MTX	0.76757	0.47468	1.24248
RTX 1000mg + MTX	ABA SUBCUT + MTX	0.72001	0.41518	1.26053
GOL 50mg + MTX	ABA SUBCUT + MTX	0.75978	0.44937	1.29816
cDMARD + MTX	ABA SUBCUT + MTX	0.33125	0.141	0.81138
ETN + MTX	ABA SUBCUT + MTX	1.76353	0.83637	3.97621
SARI 200mg + MTX	ABA SUBCUT + MTX	0.95973	0.57374	1.60912
CZP + MTX	ABA SUBCUT + MTX	1.28743	0.65733	2.6908
PBO + MTX	IFX 3mg + MTX	0.28586	0.21067	0.38323
**BARI 4mg + MTX**	**IFX 3mg + MTX**	**1.4248**	**0.95344**	**2.11341**
ADA 40mg + MTX	IFX 3mg + MTX	1.30503	0.89618	1.88886
ABA 10mg + MTX	IFX 3mg + MTX	1.00904	0.71026	1.4229
ABA SUBCUT + MTX	IFX 3mg + MTX	1.30281	0.80484	2.1067
IFX 3mg + MTX	IFX 3mg + MTX	1	1	1
RTX 1000mg + MTX	IFX 3mg + MTX	0.93819	0.56848	1.56362
GOL 50mg + MTX	IFX 3mg + MTX	0.98788	0.61292	1.60039
cDMARD + MTX	IFX 3mg + MTX	0.43052	0.18862	1.0265
ETN + MTX	IFX 3mg + MTX	2.29326	1.11913	5.00545
SARI 200mg + MTX	IFX 3mg + MTX	1.24964	0.7906	1.96818
CZP + MTX	IFX 3mg + MTX	1.67576	0.89599	3.3616
PBO + MTX	RTX 1000mg + MTX	0.30488	0.19822	0.45956
**BARI 4mg + MTX**	**RTX 1000mg + MTX**	**1.51813**	**0.92038**	**2.47158**
ADA 40mg + MTX	RTX 1000mg + MTX	1.39188	0.86461	2.21098
ABA 10mg + MTX	RTX 1000mg + MTX	1.07583	0.64156	1.78332
ABA SUBCUT + MTX	RTX 1000mg + MTX	1.38888	0.79332	2.40861
IFX 3mg + MTX	RTX 1000mg + MTX	1.06588	0.63954	1.75909
RTX 1000mg + MTX	RTX 1000mg + MTX	1	1	1
GOL 50mg + MTX	RTX 1000mg + MTX	1.05443	0.6064	1.82295
cDMARD + MTX	RTX 1000mg + MTX	0.46083	0.19217	1.13047
ETN + MTX	RTX 1000mg + MTX	2.46032	1.12833	5.52308
SARI 200mg + MTX	RTX 1000mg + MTX	1.33517	0.76715	2.3064
CZP + MTX	RTX 1000mg + MTX	1.79062	0.89467	3.76726
PBO + MTX	GOL 50mg + MTX	0.28909	0.1963	0.42225
**BARI 4mg + MTX**	**GOL 50mg + MTX**	**1.44039**	**0.90268**	**2.27143**
ADA 40mg + MTX	GOL 50mg + MTX	1.3214	0.85185	2.036
ABA 10mg + MTX	GOL 50mg + MTX	1.02069	0.6279	1.6512
ABA SUBCUT + MTX	GOL 50mg + MTX	1.31618	0.77032	2.22535
IFX 3mg + MTX	GOL 50mg + MTX	1.01227	0.62485	1.63153
RTX 1000mg + MTX	GOL 50mg + MTX	0.94838	0.54856	1.64906
GOL 50mg + MTX	GOL 50mg + MTX	1	1	1
cDMARD + MTX	GOL 50mg + MTX	0.43704	0.18424	1.04154
ETN + MTX	GOL 50mg + MTX	2.33387	1.09127	5.11437
SARI 200mg + MTX	GOL 50mg + MTX	1.26488	0.74888	2.13111
CZP + MTX	GOL 50mg + MTX	1.69967	0.8696	3.48432
PBO + MTX	cDMARD + MTX	0.66326	0.28975	1.44771
**BARI 4mg + MTX**	**cDMARD + MTX**	**3.30477**	**1.39487**	**7.50679**
ADA 40mg + MTX	cDMARD + MTX	3.02547	1.29432	6.75504

Treatment 1	Treatment 2	Median OR	95% CrI Lower	95% CrI Upper
ABA 10mg + MTX	cDMARD + MTX	2.34573	0.9736	5.34977
ABA SUBCUT + MTX	cDMARD + MTX	3.01883	1.23246	7.09211
IFX 3mg + MTX	cDMARD + MTX	2.32279	0.97419	5.30166
RTX 1000mg + MTX	cDMARD + MTX	2.16999	0.88459	5.20384
GOL 50mg + MTX	cDMARD + MTX	2.28813	0.96012	5.42775
cDMARD + MTX	cDMARD + MTX	1	1	1
ETN + MTX	cDMARD + MTX	5.33574	3.40226	8.52667
SARI 200mg + MTX	cDMARD + MTX	2.91955	1.16856	6.75753
CZP + MTX	cDMARD + MTX	3.89857	1.50284	10.3388
PBO + MTX	ETN + MTX	0.12461	0.05982	0.24377
**BARI 4mg + MTX**	**ETN + MTX**	**0.62139**	**0.28686**	**1.26021**
ADA 40mg + MTX	ETN + MTX	0.56934	0.26717	1.12682
ABA 10mg + MTX	ETN + MTX	0.43978	0.19965	0.90425
ABA SUBCUT + MTX	ETN + MTX	0.56704	0.2515	1.19565
IFX 3mg + MTX	ETN + MTX	0.43606	0.19978	0.89355
RTX 1000mg + MTX	ETN + MTX	0.40645	0.18106	0.88626
GOL 50mg + MTX	ETN + MTX	0.42847	0.19553	0.91636
cDMARD + MTX	ETN + MTX	0.18742	0.11728	0.29392
ETN + MTX	ETN + MTX	1	1	1
SARI 200mg + MTX	ETN + MTX	0.54737	0.23831	1.14828
CZP + MTX	ETN + MTX	0.73007	0.30384	1.78808
PBO + MTX	SARI 200mg + MTX	0.2286	0.16075	0.32311
**BARI 4mg + MTX**	**SARI 200mg + MTX**	**1.14103**	**0.73648**	**1.75921**
ADA 40mg + MTX	SARI 200mg + MTX	1.04483	0.68855	1.58005
ABA 10mg + MTX	SARI 200mg + MTX	0.80674	0.51072	1.27579
ABA SUBCUT + MTX	SARI 200mg + MTX	1.04196	0.62146	1.74295
IFX 3mg + MTX	SARI 200mg + MTX	0.80023	0.50808	1.26487
RTX 1000mg + MTX	SARI 200mg + MTX	0.74897	0.43358	1.30352
GOL 50mg + MTX	SARI 200mg + MTX	0.79059	0.46924	1.33533
cDMARD + MTX	SARI 200mg + MTX	0.34252	0.14798	0.85575
ETN + MTX	SARI 200mg + MTX	1.82693	0.87087	4.19623
SARI 200mg + MTX	SARI 200mg + MTX	1	1	1
CZP + MTX	SARI 200mg + MTX	1.34191	0.68806	2.80143
PBO + MTX	CZP + MTX	0.17107	0.09006	0.29887
**BARI 4mg + MTX**	**CZP + MTX**	**0.85151**	**0.42504**	**1.57467**
ADA 40mg + MTX	CZP + MTX	0.78122	0.39698	1.41942
ABA 10mg + MTX	CZP + MTX	0.60247	0.29763	1.13669
ABA SUBCUT + MTX	CZP + MTX	0.77674	0.37164	1.5213
IFX 3mg + MTX	CZP + MTX	0.59674	0.29748	1.11609
RTX 1000mg + MTX	CZP + MTX	0.55847	0.26544	1.11773
GOL 50mg + MTX	CZP + MTX	0.58835	0.287	1.14995
cDMARD + MTX	CZP + MTX	0.2565	0.09672	0.66541
ETN + MTX	CZP + MTX	1.36973	0.55926	3.29117
SARI 200mg + MTX	CZP + MTX	0.74521	0.35696	1.45336
CZP + MTX	CZP + MTX	1	1	1

Abbreviations: ABA abatacept; ADA adalimumab; BARI baricitinib; ETN etanercept; GOL golimumab; IFX infliximab; MTX methotrexate; PBO placebo; RTX rituximab; SARI sarilumab; SUBCUT subcutaneous.

[1]statistically significantly favouring BARI.

Odds ratios >1 are in favour of Treatment 1; and odds ratios <1 are in favour of Treatment 2.

9.1.3. *ACR70 response*

Table S14 Primary Analysis: Relative treatment effect of pairwise comparisons expressed as Post. Median odds ratios (with 95% CRIs) - ACR70 response at week 24- MTX-IR (Simultaneous Fixed-effects model)

Treatment 1	Treatment 2	Median OR	95% CrI Lower	95% CrI Upper
PBO + MTX	PBO + MTX	1	1	1
**BARI 4mg + MTX[1]**	**PBO + MTX**	**5.90962**	**4.1892**	**8.46738**
ADA 40mg + MTX	PBO + MTX	4.30279	3.13894	5.97356
ABA 10mg + MTX	PBO + MTX	3.17438	2.12952	4.79003
ABA SUBCUT + MTX	PBO + MTX	4.10487	2.53588	6.68776
IFX 3mg + MTX	PBO + MTX	3.55417	2.37697	5.40304
RTX 1000mg + MTX	PBO + MTX	2.6203	1.44088	4.93619
GOL 50mg + MTX	PBO + MTX	4.33691	2.52618	7.74905
cDMARD + MTX	PBO + MTX	1.55471	0.48233	5.65011
ETN + MTX	PBO + MTX	6.61907	2.3521	21.80178
SARI 200mg + MTX	PBO + MTX	4.25584	3.01852	6.06864
CZP + MTX	PBO + MTX	5.66301	2.45465	15.43534
PBO + MTX	BARI 4mg + MTX	0.16922	0.1181	0.23871
BARI 4mg + MTX	BARI 4mg + MTX	1	1	1
ADA 40mg + MTX	BARI 4mg + MTX	0.72752	0.53526	0.9834
ABA 10mg + MTX	BARI 4mg + MTX	0.53682	0.3154	0.91589
ABA SUBCUT + MTX	BARI 4mg + MTX	0.69437	0.43106	1.11046
IFX 3mg + MTX	BARI 4mg + MTX	0.60233	0.35289	1.02472
RTX 1000mg + MTX	BARI 4mg + MTX	0.44261	0.22368	0.90395
GOL 50mg + MTX	BARI 4mg + MTX	0.73357	0.3897	1.42801
cDMARD + MTX	BARI 4mg + MTX	0.26203	0.07803	0.98069
ETN + MTX	BARI 4mg + MTX	1.11407	0.37821	3.82348
SARI 200mg + MTX	BARI 4mg + MTX	0.72011	0.43627	1.17349
CZP + MTX	BARI 4mg + MTX	0.95794	0.38804	2.76948
PBO + MTX	ADA 40mg + MTX	0.23241	0.1674	0.31858
**BARI 4mg + MTX[1]**	**ADA 40mg + MTX**	**1.37453**	**1.01688**	**1.86825**
ADA 40mg + MTX	ADA 40mg + MTX	1	1	1
ABA 10mg + MTX	ADA 40mg + MTX	0.73795	0.44408	1.23276
ABA SUBCUT + MTX	ADA 40mg + MTX	0.95444	0.65955	1.3789
IFX 3mg + MTX	ADA 40mg + MTX	0.82725	0.49446	1.37774
RTX 1000mg + MTX	ADA 40mg + MTX	0.60862	0.31315	1.21682
GOL 50mg + MTX	ADA 40mg + MTX	1.00859	0.54243	1.93386
cDMARD + MTX	ADA 40mg + MTX	0.36033	0.10901	1.33238
ETN + MTX	ADA 40mg + MTX	1.53652	0.52915	5.14112
SARI 200mg + MTX	ADA 40mg + MTX	0.99014	0.61852	1.58464
CZP + MTX	ADA 40mg + MTX	1.31606	0.54265	3.73832
PBO + MTX	ABA 10mg + MTX	0.31502	0.20877	0.46959
**BARI 4mg + MTX[1]**	**ABA 10mg + MTX**	**1.86283**	**1.09183**	**3.17055**
ADA 40mg + MTX	ABA 10mg + MTX	1.35511	0.81119	2.25187
ABA 10mg + MTX	ABA 10mg + MTX	1	1	1
ABA SUBCUT + MTX	ABA 10mg + MTX	1.2926	0.68843	2.42974
IFX 3mg + MTX	ABA 10mg + MTX	1.12181	0.72189	1.74015
RTX 1000mg + MTX	ABA 10mg + MTX	0.82524	0.40494	1.72103
GOL 50mg + MTX	ABA 10mg + MTX	1.36573	0.70389	2.75068

Treatment 1	Treatment 2	Median OR	95% CrI Lower	95% CrI Upper
cDMARD + MTX	ABA 10mg + MTX	0.489	0.14209	1.86374
ETN + MTX	ABA 10mg + MTX	2.08426	0.69437	7.20118
SARI 200mg + MTX	ABA 10mg + MTX	1.34121	0.78464	2.27303
CZP + MTX	ABA 10mg + MTX	1.78807	0.70646	5.25484
PBO + MTX	ABA SUBCUT + MTX	0.24361	0.14953	0.39434
**BARI 4mg + MTX**	**ABA SUBCUT + MTX**	**1.44015**	**0.90052**	**2.31987**
ADA 40mg + MTX	ABA SUBCUT + MTX	1.04773	0.72522	1.51618
ABA 10mg + MTX	ABA SUBCUT + MTX	0.77363	0.41157	1.45257
ABA SUBCUT + MTX	ABA SUBCUT + MTX	1	1	1
IFX 3mg + MTX	ABA SUBCUT + MTX	0.86693	0.46271	1.63136
RTX 1000mg + MTX	ABA SUBCUT + MTX	0.64112	0.29711	1.39643
GOL 50mg + MTX	ABA SUBCUT + MTX	1.05744	0.51623	2.22824
cDMARD + MTX	ABA SUBCUT + MTX	0.38036	0.10771	1.46031
ETN + MTX	ABA SUBCUT + MTX	1.61356	0.52512	5.7099
SARI 200mg + MTX	ABA SUBCUT + MTX	1.03779	0.56823	1.88664
CZP + MTX	ABA SUBCUT + MTX	1.39165	0.52714	4.16014
PBO + MTX	IFX 3mg + MTX	0.28136	0.18508	0.4207
**BARI 4mg + MTX**	**IFX 3mg + MTX**	**1.66023**	**0.97588**	**2.83374**
ADA 40mg + MTX	IFX 3mg + MTX	1.20883	0.72583	2.02242
ABA 10mg + MTX	IFX 3mg + MTX	0.89141	0.57466	1.38526
ABA SUBCUT + MTX	IFX 3mg + MTX	1.15349	0.61299	2.16117
IFX 3mg + MTX	IFX 3mg + MTX	1	1	1
RTX 1000mg + MTX	IFX 3mg + MTX	0.73684	0.35883	1.54371
GOL 50mg + MTX	IFX 3mg + MTX	1.21736	0.6252	2.44052
cDMARD + MTX	IFX 3mg + MTX	0.43707	0.12739	1.65367
ETN + MTX	IFX 3mg + MTX	1.8603	0.61942	6.41905
SARI 200mg + MTX	IFX 3mg + MTX	1.19713	0.69921	2.04126
CZP + MTX	IFX 3mg + MTX	1.59257	0.63191	4.65816
PBO + MTX	RTX 1000mg + MTX	0.38164	0.20259	0.69402
**BARI 4mg + MTX[1]**	**RTX 1000mg + MTX**	**2.25934**	**1.10626**	**4.4707**
ADA 40mg + MTX	RTX 1000mg + MTX	1.64305	0.82181	3.19337
ABA 10mg + MTX	RTX 1000mg + MTX	1.21176	0.58105	2.46948
ABA SUBCUT + MTX	RTX 1000mg + MTX	1.55977	0.71611	3.36576
IFX 3mg + MTX	RTX 1000mg + MTX	1.35714	0.64779	2.78687
RTX 1000mg + MTX	RTX 1000mg + MTX	1	1	1
GOL 50mg + MTX	RTX 1000mg + MTX	1.65435	0.74276	3.72542
cDMARD + MTX	RTX 1000mg + MTX	0.59257	0.16019	2.28887
ETN + MTX	RTX 1000mg + MTX	2.52556	0.77569	8.97035
SARI 200mg + MTX	RTX 1000mg + MTX	1.62586	0.7907	3.26308
CZP + MTX	RTX 1000mg + MTX	2.16587	0.77439	6.70475
PBO + MTX	GOL 50mg + MTX	0.23058	0.12905	0.39586
**BARI 4mg + MTX**	**GOL 50mg + MTX**	**1.3632**	**0.70027**	**2.56605**
ADA 40mg + MTX	GOL 50mg + MTX	0.99149	0.5171	1.84355
ABA 10mg + MTX	GOL 50mg + MTX	0.73221	0.36355	1.42068
ABA SUBCUT + MTX	GOL 50mg + MTX	0.94568	0.44878	1.93712
IFX 3mg + MTX	GOL 50mg + MTX	0.82145	0.40975	1.5995
RTX 1000mg + MTX	GOL 50mg + MTX	0.60447	0.26843	1.34633
GOL 50mg + MTX	GOL 50mg + MTX	1	1	1
cDMARD + MTX	GOL 50mg + MTX	0.35855	0.09995	1.36593
ETN + MTX	GOL 50mg + MTX	1.5302	0.48624	5.27018
SARI 200mg + MTX	GOL 50mg + MTX	0.98325	0.49571	1.86838
CZP + MTX	GOL 50mg + MTX	1.31051	0.48505	3.92783
PBO + MTX	cDMARD + MTX	0.64321	0.17699	2.07325
**BARI 4mg + MTX[1]**	**cDMARD + MTX**	**3.81635**	**1.01969**	**12.81611**
ADA 40mg + MTX	cDMARD + MTX	2.77524	0.75053	9.17349
ABA 10mg + MTX	cDMARD + MTX	2.045	0.53656	7.03796

Treatment 1	Treatment 2	Median OR	95% CrI Lower	95% CrI Upper
ABA SUBCUT + MTX	cDMARD + MTX	2.62911	0.68479	9.2844
IFX 3mg + MTX	cDMARD + MTX	2.28795	0.60471	7.85014
RTX 1000mg + MTX	cDMARD + MTX	1.68756	0.4369	6.24261
GOL 50mg + MTX	cDMARD + MTX	2.789	0.7321	10.00526
cDMARD + MTX	cDMARD + MTX	1	1	1
ETN + MTX	cDMARD + MTX	4.25686	2.44213	7.84486
SARI 200mg + MTX	cDMARD + MTX	2.752	0.72016	9.23747
CZP + MTX	cDMARD + MTX	3.67318	0.85659	16.46029
PBO + MTX	ETN + MTX	0.15108	0.04587	0.42515
**BARI 4mg + MTX**	**ETN + MTX**	**0.89761**	**0.26154**	**2.64401**
ADA 40mg + MTX	ETN + MTX	0.65082	0.19451	1.88983
ABA 10mg + MTX	ETN + MTX	0.47979	0.13887	1.44015
ABA SUBCUT + MTX	ETN + MTX	0.61975	0.17513	1.90433
IFX 3mg + MTX	ETN + MTX	0.53755	0.15579	1.6144
RTX 1000mg + MTX	ETN + MTX	0.39595	0.11148	1.28917
GOL 50mg + MTX	ETN + MTX	0.65351	0.18975	2.05659
cDMARD + MTX	ETN + MTX	0.23492	0.12747	0.40948
ETN + MTX	ETN + MTX	1	1	1
SARI 200mg + MTX	ETN + MTX	0.64539	0.18652	1.90243
CZP + MTX	ETN + MTX	0.85835	0.21701	3.49937
PBO + MTX	SARI 200mg + MTX	0.23497	0.16478	0.33129
**BARI 4mg + MTX**	**SARI 200mg + MTX**	**1.38867**	**0.85216**	**2.29214**
ADA 40mg + MTX	SARI 200mg + MTX	1.00996	0.63106	1.61677
ABA 10mg + MTX	SARI 200mg + MTX	0.7456	0.43994	1.27447
ABA SUBCUT + MTX	SARI 200mg + MTX	0.96359	0.53004	1.75986
IFX 3mg + MTX	SARI 200mg + MTX	0.83533	0.48989	1.43019
RTX 1000mg + MTX	SARI 200mg + MTX	0.61506	0.30646	1.2647
GOL 50mg + MTX	SARI 200mg + MTX	1.01703	0.53522	2.0173
cDMARD + MTX	SARI 200mg + MTX	0.36337	0.10825	1.38858
ETN + MTX	SARI 200mg + MTX	1.54944	0.52564	5.36149
SARI 200mg + MTX	SARI 200mg + MTX	1	1	1
CZP + MTX	SARI 200mg + MTX	1.33242	0.53458	3.87891
PBO + MTX	CZP + MTX	0.17658	0.06479	0.40739
**BARI 4mg + MTX**	**CZP + MTX**	**1.04391**	**0.36108**	**2.57707**
ADA 40mg + MTX	CZP + MTX	0.75984	0.2675	1.84281
ABA 10mg + MTX	CZP + MTX	0.55926	0.1903	1.4155
ABA SUBCUT + MTX	CZP + MTX	0.71857	0.24038	1.89703
IFX 3mg + MTX	CZP + MTX	0.62792	0.21468	1.58251
RTX 1000mg + MTX	CZP + MTX	0.46171	0.14915	1.29134
GOL 50mg + MTX	CZP + MTX	0.76306	0.25459	2.06164
cDMARD + MTX	CZP + MTX	0.27224	0.06075	1.16742
ETN + MTX	CZP + MTX	1.16502	0.28577	4.60802
SARI 200mg + MTX	CZP + MTX	0.75051	0.2578	1.87064
CZP + MTX	CZP + MTX	1	1	1

Abbreviations: ABA abatacept; ADA adalimumab; BARI baricitinib; ETN etanercept; GOL golimumab; IFX infliximab; MTX methotrexate; PBO placebo; RTX rituximab; SARI sarilumab; SUBCUT subcutaneous.

[1]statistically significantly favouring BARI.

Odds ratios >1 are in favour of Treatment 1; and odds ratios <1 are in favour of Treatment 2.

9.2. Primary analyses (Simultaneous Random – effects)

9.2.1. ACR20 Response

Table S15 Primary Analysis: Relative treatment effect of pairwise comparisons expressed as Post. Median odds ratios (with 95% CrIs) - ACR20 response at week 24: MTX-IR (Simultaneous Random-effects model)

Treatment 1	Treatment 2	Median OR	95% CrI Lower	95% CrI Upper
PBO + MTX	PBO + MTX	1	1	1
**BARI 4mg + MTX[1]**	**PBO + MTX**	**5.35584**	**3.92299**	**7.49344**
ADA 40mg + MTX	PBO + MTX	4.04785	3.24816	5.14534
ABA 10mg + MTX	PBO + MTX	3.65565	2.71153	4.94564
ABA SUBCUT + MTX	PBO + MTX	4.16303	2.74758	6.40342
IFX 3mg + MTX	PBO + MTX	3.2329	2.47406	4.16245
RTX 1000mg + MTX	PBO + MTX	3.27508	2.29895	4.71786
GOL 50mg + MTX	PBO + MTX	3.74964	2.69673	5.26016
cDMARD + MTX	PBO + MTX	1.96546	1.01442	3.63202
ETN + MTX	PBO + MTX	9.07524	5.16827	15.49234
SARI 200mg + MTX	PBO + MTX	4.11785	2.79596	6.13754
CZP + MTX	PBO + MTX	3.50218	2.21277	5.6458
PBO + MTX	BARI 4mg + MTX	0.18671	0.13345	0.25491
BARI 4mg + MTX	BARI 4mg + MTX	1	1	1
ADA 40mg + MTX	BARI 4mg + MTX	0.7553	0.53973	1.0601
ABA 10mg + MTX	BARI 4mg + MTX	0.68407	0.43409	1.05208
ABA SUBCUT + MTX	BARI 4mg + MTX	0.77689	0.47365	1.27108
IFX 3mg + MTX	BARI 4mg + MTX	0.60353	0.39056	0.89735
RTX 1000mg + MTX	BARI 4mg + MTX	0.60873	0.37919	0.98675
GOL 50mg + MTX	BARI 4mg + MTX	0.69873	0.43917	1.10684
cDMARD + MTX	BARI 4mg + MTX	0.36577	0.17613	0.73274
ETN + MTX	BARI 4mg + MTX	1.69464	0.88852	3.16414
SARI 200mg + MTX	BARI 4mg + MTX	0.768	0.4612	1.26411
CZP + MTX	BARI 4mg + MTX	0.65326	0.37074	1.15739
PBO + MTX	ADA 40mg + MTX	0.24704	0.19435	0.30787
**BARI 4mg + MTX**	**ADA 40mg + MTX**	**1.32398**	**0.94331**	**1.85279**
ADA 40mg + MTX	ADA 40mg + MTX	1	1	1
ABA 10mg + MTX	ADA 40mg + MTX	0.90221	0.61655	1.29963
ABA SUBCUT + MTX	ADA 40mg + MTX	1.02776	0.70657	1.49771
IFX 3mg + MTX	ADA 40mg + MTX	0.79855	0.55642	1.10682
RTX 1000mg + MTX	ADA 40mg + MTX	0.80694	0.53019	1.23253
GOL 50mg + MTX	ADA 40mg + MTX	0.92346	0.61765	1.37197
cDMARD + MTX	ADA 40mg + MTX	0.48391	0.24029	0.92461
ETN + MTX	ADA 40mg + MTX	2.24358	1.22536	3.97051
SARI 200mg + MTX	ADA 40mg + MTX	1.02018	0.645	1.59104
CZP + MTX	ADA 40mg + MTX	0.86538	0.51335	1.442
PBO + MTX	ABA 10mg + MTX	0.27355	0.2022	0.3688
**BARI 4mg + MTX**	**ABA 10mg + MTX**	**1.46183**	**0.9505**	**2.30367**
ADA 40mg + MTX	ABA 10mg + MTX	1.10838	0.76945	1.62194
ABA 10mg + MTX	ABA 10mg + MTX	1	1	1
ABA SUBCUT + MTX	ABA 10mg + MTX	1.13914	0.69183	1.91718

Treatment 1	Treatment 2	Median OR	95% CrI Lower	95% CrI Upper
IFX 3mg + MTX	ABA 10mg + MTX	0.88444	0.61126	1.25984
RTX 1000mg + MTX	ABA 10mg + MTX	0.89416	0.56796	1.43754
GOL 50mg + MTX	ABA 10mg + MTX	1.02256	0.66003	1.62713
cDMARD + MTX	ABA 10mg + MTX	0.53481	0.26518	1.05824
ETN + MTX	ABA 10mg + MTX	2.48334	1.34562	4.55605
SARI 200mg + MTX	ABA 10mg + MTX	1.12485	0.69254	1.84039
CZP + MTX	ABA 10mg + MTX	0.95673	0.55388	1.68935
PBO + MTX	ABA SUBCUT + MTX	0.24021	0.15617	0.36396
**BARI 4mg + MTX**	**ABA SUBCUT + MTX**	**1.28718**	**0.78673**	**2.11128**
ADA 40mg + MTX	ABA SUBCUT + MTX	0.97299	0.66768	1.41528
ABA 10mg + MTX	ABA SUBCUT + MTX	0.87785	0.5216	1.44545
ABA SUBCUT + MTX	ABA SUBCUT + MTX	1	1	1
IFX 3mg + MTX	ABA SUBCUT + MTX	0.77545	0.46822	1.24677
RTX 1000mg + MTX	ABA SUBCUT + MTX	0.78361	0.45437	1.3625
GOL 50mg + MTX	ABA SUBCUT + MTX	0.89891	0.52379	1.5316
cDMARD + MTX	ABA SUBCUT + MTX	0.47037	0.21716	0.97988
ETN + MTX	ABA SUBCUT + MTX	2.17916	1.08495	4.28379
SARI 200mg + MTX	ABA SUBCUT + MTX	0.99016	0.55548	1.73709
CZP + MTX	ABA SUBCUT + MTX	0.84322	0.44846	1.56888
PBO + MTX	IFX 3mg + MTX	0.30932	0.24024	0.40419
**BARI 4mg + MTX[1]**	**IFX 3mg + MTX**	**1.65691**	**1.11439**	**2.56043**
ADA 40mg + MTX	IFX 3mg + MTX	1.25227	0.90349	1.7972
ABA 10mg + MTX	IFX 3mg + MTX	1.13066	0.79375	1.63596
ABA SUBCUT + MTX	IFX 3mg + MTX	1.28958	0.80207	2.13575
IFX 3mg + MTX	IFX 3mg + MTX	1	1	1
RTX 1000mg + MTX	IFX 3mg + MTX	1.01241	0.66366	1.58589
GOL 50mg + MTX	IFX 3mg + MTX	1.16029	0.77625	1.77522
cDMARD + MTX	IFX 3mg + MTX	0.6089	0.30166	1.18882
ETN + MTX	IFX 3mg + MTX	2.81253	1.53142	5.10349
SARI 200mg + MTX	IFX 3mg + MTX	1.27327	0.8076	2.0551
CZP + MTX	IFX 3mg + MTX	1.08211	0.6523	1.8689
PBO + MTX	RTX 1000mg + MTX	0.30534	0.21196	0.43498
**BARI 4mg + MTX[1]**	**RTX 1000mg + MTX**	**1.64276**	**1.01343**	**2.63722**
ADA 40mg + MTX	RTX 1000mg + MTX	1.23925	0.81134	1.88612
ABA 10mg + MTX	RTX 1000mg + MTX	1.11836	0.69563	1.76068
ABA SUBCUT + MTX	RTX 1000mg + MTX	1.27615	0.73395	2.20084
IFX 3mg + MTX	RTX 1000mg + MTX	0.98774	0.63056	1.50681
RTX 1000mg + MTX	RTX 1000mg + MTX	1	1	1
GOL 50mg + MTX	RTX 1000mg + MTX	1.14542	0.71195	1.83143
cDMARD + MTX	RTX 1000mg + MTX	0.59969	0.28289	1.21739
ETN + MTX	RTX 1000mg + MTX	2.77523	1.41943	5.18231
SARI 200mg + MTX	RTX 1000mg + MTX	1.26043	0.73537	2.13838
CZP + MTX	RTX 1000mg + MTX	1.07131	0.60018	1.89118
PBO + MTX	GOL 50mg + MTX	0.26669	0.19011	0.37082
**BARI 4mg + MTX**	**GOL 50mg + MTX**	**1.43118**	**0.90347**	**2.27701**
ADA 40mg + MTX	GOL 50mg + MTX	1.08288	0.72888	1.61904
ABA 10mg + MTX	GOL 50mg + MTX	0.97794	0.61458	1.51508
ABA SUBCUT + MTX	GOL 50mg + MTX	1.11246	0.65291	1.90917
IFX 3mg + MTX	GOL 50mg + MTX	0.86186	0.56331	1.28825
RTX 1000mg + MTX	GOL 50mg + MTX	0.87305	0.54602	1.40458
GOL 50mg + MTX	GOL 50mg + MTX	1	1	1
cDMARD + MTX	GOL 50mg + MTX	0.52491	0.24797	1.05509
ETN + MTX	GOL 50mg + MTX	2.43095	1.24619	4.51476
SARI 200mg + MTX	GOL 50mg + MTX	1.10072	0.65262	1.84272
CZP + MTX	GOL 50mg + MTX	0.93755	0.5321	1.64225
PBO + MTX	cDMARD + MTX	0.50879	0.27533	0.98579

Treatment 1	Treatment 2	Median OR	95% CrI Lower	95% CrI Upper
**BARI 4mg + MTX[1]**	**cDMARD + MTX**	**2.73399**	**1.36474**	**5.67749**
ADA 40mg + MTX	cDMARD + MTX	2.06651	1.08153	4.16171
ABA 10mg + MTX	cDMARD + MTX	1.86983	0.94497	3.77104
ABA SUBCUT + MTX	cDMARD + MTX	2.12597	1.02053	4.60488
IFX 3mg + MTX	cDMARD + MTX	1.6423	0.84117	3.31503
RTX 1000mg + MTX	cDMARD + MTX	1.66752	0.82143	3.53492
GOL 50mg + MTX	cDMARD + MTX	1.9051	0.94779	4.03273
cDMARD + MTX	cDMARD + MTX	1	1	1
ETN + MTX	cDMARD + MTX	4.64788	2.82639	7.5222
SARI 200mg + MTX	cDMARD + MTX	2.1061	1.02021	4.52332
CZP + MTX	cDMARD + MTX	1.78709	0.83888	4.03127
PBO + MTX	ETN + MTX	0.11019	0.06455	0.19349
**BARI 4mg + MTX**	**ETN + MTX**	**0.59009**	**0.31604**	**1.12546**
ADA 40mg + MTX	ETN + MTX	0.44572	0.25186	0.81609
ABA 10mg + MTX	ETN + MTX	0.40268	0.21949	0.74315
ABA SUBCUT + MTX	ETN + MTX	0.45889	0.23344	0.9217
IFX 3mg + MTX	ETN + MTX	0.35555	0.19594	0.65299
RTX 1000mg + MTX	ETN + MTX	0.36033	0.19296	0.70451
GOL 50mg + MTX	ETN + MTX	0.41136	0.2215	0.80244
cDMARD + MTX	ETN + MTX	0.21515	0.13294	0.35381
ETN + MTX	ETN + MTX	1	1	1
SARI 200mg + MTX	ETN + MTX	0.45346	0.23223	0.89804
CZP + MTX	ETN + MTX	0.38446	0.19224	0.79707
PBO + MTX	SARI 200mg + MTX	0.24284	0.16293	0.35766
**BARI 4mg + MTX**	**SARI 200mg + MTX**	**1.30208**	**0.79107**	**2.16827**
ADA 40mg + MTX	SARI 200mg + MTX	0.98022	0.62852	1.55039
ABA 10mg + MTX	SARI 200mg + MTX	0.88901	0.54336	1.44396
ABA SUBCUT + MTX	SARI 200mg + MTX	1.00994	0.57568	1.80026
IFX 3mg + MTX	SARI 200mg + MTX	0.78538	0.48659	1.23824
RTX 1000mg + MTX	SARI 200mg + MTX	0.79338	0.46764	1.35985
GOL 50mg + MTX	SARI 200mg + MTX	0.9085	0.54267	1.53229
cDMARD + MTX	SARI 200mg + MTX	0.47481	0.22108	0.98019
ETN + MTX	SARI 200mg + MTX	2.20529	1.11354	4.30603
SARI 200mg + MTX	SARI 200mg + MTX	1	1	1
CZP + MTX	SARI 200mg + MTX	0.84964	0.46686	1.57357
PBO + MTX	CZP + MTX	0.28554	0.17712	0.45192
**BARI 4mg + MTX**	**CZP + MTX**	**1.53078**	**0.86401**	**2.69728**
ADA 40mg + MTX	CZP + MTX	1.15556	0.69348	1.948
ABA 10mg + MTX	CZP + MTX	1.04522	0.59194	1.80543
ABA SUBCUT + MTX	CZP + MTX	1.18593	0.6374	2.22987
IFX 3mg + MTX	CZP + MTX	0.92412	0.53507	1.53305
RTX 1000mg + MTX	CZP + MTX	0.93344	0.52877	1.66618
GOL 50mg + MTX	CZP + MTX	1.06661	0.60892	1.87936
cDMARD + MTX	CZP + MTX	0.55957	0.24806	1.19207
ETN + MTX	CZP + MTX	2.60102	1.2546	5.20184
SARI 200mg + MTX	CZP + MTX	1.17697	0.6355	2.14195
CZP + MTX	CZP + MTX	1	1	1

Abbreviations: ABA abatacept; ADA adalimumab; BARI baricitinib; ETN etanercept; GOL golimumab; IFX infliximab; MTX methotrexate; PBO placebo; RTX rituximab; SARI sarilumab; SUBCUT subcutaneous.

[1]statistically significantly favouring BARI.

Odds ratios >1 are in favour of Treatment 1; and odds ratios <1 are in favour of Treatment 2.

9.2.2. *ACR50 Response*

Table S16 Primary Analysis: Relative treatment effect of pairwise comparisons expressed as Post. Median odds ratios (with 95% CrIs) – ACR50 response at week 24: MTX-IR (Simultaneous Random effects-model)

Treatment 1	Treatment 2	Median OR	95% CrI Lower	95% CrI Upper
PBO + MTX	PBO + MTX	1	1	1
**BARI 4mg + MTX[1]**	**PBO + MTX**	**5.05962**	**3.53049**	**7.52433**
ADA 40mg + MTX	PBO + MTX	4.71494	3.60397	6.52315
ABA 10mg + MTX	PBO + MTX	3.5264	2.44954	5.07112
ABA SUBCUT + MTX	PBO + MTX	4.68506	2.93347	7.90648
IFX 3mg + MTX	PBO + MTX	3.4878	2.42097	4.98225
RTX 1000mg + MTX	PBO + MTX	3.32256	2.10945	5.3351
GOL 50mg + MTX	PBO + MTX	3.46788	2.30761	5.32052
cDMARD + MTX	PBO + MTX	1.54725	0.6731	3.89247
ETN + MTX	PBO + MTX	8.18398	3.98304	18.29486
SARI 200mg + MTX	PBO + MTX	4.37612	2.76577	6.86395
CZP + MTX	PBO + MTX	5.94439	3.11934	12.02306
PBO + MTX	BARI 4mg + MTX	0.19764	0.1329	0.28325
BARI 4mg + MTX	BARI 4mg + MTX	1	1	1
ADA 40mg + MTX	BARI 4mg + MTX	0.92822	0.65255	1.39266
ABA 10mg + MTX	BARI 4mg + MTX	0.69828	0.40804	1.15789
ABA SUBCUT + MTX	BARI 4mg + MTX	0.92354	0.53908	1.64233
IFX 3mg + MTX	BARI 4mg + MTX	0.6893	0.40011	1.14307
RTX 1000mg + MTX	BARI 4mg + MTX	0.65503	0.36327	1.19257
GOL 50mg + MTX	BARI 4mg + MTX	0.68519	0.38747	1.19079
cDMARD + MTX	BARI 4mg + MTX	0.30696	0.1233	0.81985
ETN + MTX	BARI 4mg + MTX	1.61983	0.71757	3.85302
SARI 200mg + MTX	BARI 4mg + MTX	0.86529	0.46886	1.5228
CZP + MTX	BARI 4mg + MTX	1.17462	0.55193	2.54545
PBO + MTX	ADA 40mg + MTX	0.21209	0.1533	0.27747
**BARI 4mg + MTX**	**ADA 40mg + MTX**	**1.07733**	**0.71805**	**1.53244**
ADA 40mg + MTX	ADA 40mg + MTX	1	1	1
ABA 10mg + MTX	ADA 40mg + MTX	0.74978	0.45839	1.15376
ABA SUBCUT + MTX	ADA 40mg + MTX	0.99318	0.65263	1.50163
IFX 3mg + MTX	ADA 40mg + MTX	0.73981	0.44883	1.13692
RTX 1000mg + MTX	ADA 40mg + MTX	0.70237	0.40925	1.21328
GOL 50mg + MTX	ADA 40mg + MTX	0.73496	0.43905	1.2021
cDMARD + MTX	ADA 40mg + MTX	0.32895	0.13568	0.83026
ETN + MTX	ADA 40mg + MTX	1.73026	0.79953	3.97687
SARI 200mg + MTX	ADA 40mg + MTX	0.929	0.52052	1.53316
CZP + MTX	ADA 40mg + MTX	1.26021	0.61114	2.62781
PBO + MTX	ABA 10mg + MTX	0.28358	0.1972	0.40824
**BARI 4mg + MTX**	**ABA 10mg + MTX**	**1.4321**	**0.86364**	**2.45075**
ADA 40mg + MTX	ABA 10mg + MTX	1.33373	0.86673	2.18156
ABA 10mg + MTX	ABA 10mg + MTX	1	1	1
ABA SUBCUT + MTX	ABA 10mg + MTX	1.32629	0.73838	2.49913
IFX 3mg + MTX	ABA 10mg + MTX	0.98749	0.64337	1.50654
RTX 1000mg + MTX	ABA 10mg + MTX	0.93935	0.52506	1.72004
GOL 50mg + MTX	ABA 10mg + MTX	0.98459	0.56929	1.71025

Treatment 1	Treatment 2	Median OR	95% CrI Lower	95% CrI Upper
cDMARD + MTX	ABA 10mg + MTX	0.43907	0.17766	1.1649
ETN + MTX	ABA 10mg + MTX	2.31787	1.03543	5.55429
SARI 200mg + MTX	ABA 10mg + MTX	1.2379	0.69507	2.19931
CZP + MTX	ABA 10mg + MTX	1.68478	0.80678	3.68825
PBO + MTX	ABA SUBCUT + MTX	0.21344	0.12648	0.34089
**BARI 4mg + MTX**	**ABA SUBCUT + MTX**	**1.08279**	**0.60889**	**1.85503**
ADA 40mg + MTX	ABA SUBCUT + MTX	1.00686	0.66594	1.53225
ABA 10mg + MTX	ABA SUBCUT + MTX	0.75398	0.40014	1.35431
ABA SUBCUT + MTX	ABA SUBCUT + MTX	1	1	1
IFX 3mg + MTX	ABA SUBCUT + MTX	0.7433	0.39166	1.33034
RTX 1000mg + MTX	ABA SUBCUT + MTX	0.70906	0.3587	1.38202
GOL 50mg + MTX	ABA SUBCUT + MTX	0.73946	0.3847	1.38197
cDMARD + MTX	ABA SUBCUT + MTX	0.33145	0.12784	0.89828
ETN + MTX	ABA SUBCUT + MTX	1.74963	0.73875	4.31087
SARI 200mg + MTX	ABA SUBCUT + MTX	0.93376	0.46696	1.77315
CZP + MTX	ABA SUBCUT + MTX	1.2718	0.55847	2.90257
PBO + MTX	IFX 3mg + MTX	0.28671	0.20071	0.41306
**BARI 4mg + MTX**	**IFX 3mg + MTX**	**1.45075**	**0.87483**	**2.49933**
ADA 40mg + MTX	IFX 3mg + MTX	1.3517	0.87957	2.22803
ABA 10mg + MTX	IFX 3mg + MTX	1.01267	0.66377	1.55431
ABA SUBCUT + MTX	IFX 3mg + MTX	1.34536	0.75169	2.55326
IFX 3mg + MTX	IFX 3mg + MTX	1	1	1
RTX 1000mg + MTX	IFX 3mg + MTX	0.95097	0.54175	1.73398
GOL 50mg + MTX	IFX 3mg + MTX	0.99298	0.58405	1.73865
cDMARD + MTX	IFX 3mg + MTX	0.44532	0.1786	1.19551
ETN + MTX	IFX 3mg + MTX	2.34642	1.06832	5.69848
SARI 200mg + MTX	IFX 3mg + MTX	1.25319	0.70763	2.2699
CZP + MTX	IFX 3mg + MTX	1.70722	0.83149	3.73733
PBO + MTX	RTX 1000mg + MTX	0.30097	0.18744	0.47406
**BARI 4mg + MTX**	**RTX 1000mg + MTX**	**1.52664**	**0.83853**	**2.75279**
ADA 40mg + MTX	RTX 1000mg + MTX	1.42375	0.82421	2.44351
ABA 10mg + MTX	RTX 1000mg + MTX	1.06456	0.58138	1.90455
ABA SUBCUT + MTX	RTX 1000mg + MTX	1.41032	0.72358	2.78783
IFX 3mg + MTX	RTX 1000mg + MTX	1.05155	0.57671	1.84587
RTX 1000mg + MTX	RTX 1000mg + MTX	1	1	1
GOL 50mg + MTX	RTX 1000mg + MTX	1.04306	0.56302	1.91748
cDMARD + MTX	RTX 1000mg + MTX	0.46992	0.18135	1.26209
ETN + MTX	RTX 1000mg + MTX	2.47438	1.04839	6.00596
SARI 200mg + MTX	RTX 1000mg + MTX	1.31565	0.6699	2.49993
CZP + MTX	RTX 1000mg + MTX	1.7942	0.8076	4.04698
PBO + MTX	GOL 50mg + MTX	0.28836	0.18795	0.43335
**BARI 4mg + MTX**	**GOL 50mg + MTX**	**1.45945**	**0.83978**	**2.58083**
ADA 40mg + MTX	GOL 50mg + MTX	1.36062	0.83188	2.27767
ABA 10mg + MTX	GOL 50mg + MTX	1.01565	0.58471	1.75657
ABA SUBCUT + MTX	GOL 50mg + MTX	1.35233	0.72361	2.59941
IFX 3mg + MTX	GOL 50mg + MTX	1.00707	0.57516	1.71218
RTX 1000mg + MTX	GOL 50mg + MTX	0.95872	0.52152	1.77613
GOL 50mg + MTX	GOL 50mg + MTX	1	1	1
cDMARD + MTX	GOL 50mg + MTX	0.44748	0.17702	1.16485
ETN + MTX	GOL 50mg + MTX	2.37344	1.04336	5.6369
SARI 200mg + MTX	GOL 50mg + MTX	1.2614	0.66821	2.33281
CZP + MTX	GOL 50mg + MTX	1.71654	0.8022	3.74982
PBO + MTX	cDMARD + MTX	0.64631	0.25691	1.48567
**BARI 4mg + MTX**[1]	**cDMARD + MTX**	**3.25776**	**1.21973**	**8.11059**
ADA 40mg + MTX	cDMARD + MTX	3.03994	1.20445	7.37022
ABA 10mg + MTX	cDMARD + MTX	2.27754	0.85844	5.62859

Treatment 1	Treatment 2	Median OR	95% CrI Lower	95% CrI Upper
ABA SUBCUT + MTX	cDMARD + MTX	3.01702	1.11324	7.8225
IFX 3mg + MTX	cDMARD + MTX	2.24559	0.83646	5.59925
RTX 1000mg + MTX	cDMARD + MTX	2.12804	0.79234	5.51429
GOL 50mg + MTX	cDMARD + MTX	2.23473	0.85848	5.64903
cDMARD + MTX	cDMARD + MTX	1	1	1
ETN + MTX	cDMARD + MTX	5.28318	3.09649	9.05222
SARI 200mg + MTX	cDMARD + MTX	2.82397	0.99702	7.25722
CZP + MTX	cDMARD + MTX	3.81651	1.28293	11.26545
PBO + MTX	ETN + MTX	0.12219	0.05466	0.25106
**BARI 4mg + MTX**	**ETN + MTX**	**0.61735**	**0.25954**	**1.39359**
ADA 40mg + MTX	ETN + MTX	0.57795	0.25145	1.25073
ABA 10mg + MTX	ETN + MTX	0.43143	0.18004	0.96578
ABA SUBCUT + MTX	ETN + MTX	0.57155	0.23197	1.35365
IFX 3mg + MTX	ETN + MTX	0.42618	0.17549	0.93605
RTX 1000mg + MTX	ETN + MTX	0.40414	0.1665	0.95384
GOL 50mg + MTX	ETN + MTX	0.42133	0.1774	0.95844
cDMARD + MTX	ETN + MTX	0.18928	0.11047	0.32295
ETN + MTX	ETN + MTX	1	1	1
SARI 200mg + MTX	ETN + MTX	0.53387	0.21185	1.26211
CZP + MTX	ETN + MTX	0.71999	0.26997	1.97338
PBO + MTX	SARI 200mg + MTX	0.22851	0.14569	0.36156
**BARI 4mg + MTX**	**SARI 200mg + MTX**	**1.15568**	**0.65668**	**2.13281**
ADA 40mg + MTX	SARI 200mg + MTX	1.07643	0.65225	1.92116
ABA 10mg + MTX	SARI 200mg + MTX	0.80782	0.45469	1.43871
ABA SUBCUT + MTX	SARI 200mg + MTX	1.07094	0.56397	2.14153
IFX 3mg + MTX	SARI 200mg + MTX	0.79796	0.44055	1.41316
RTX 1000mg + MTX	SARI 200mg + MTX	0.76008	0.40001	1.49276
GOL 50mg + MTX	SARI 200mg + MTX	0.79277	0.42867	1.49654
cDMARD + MTX	SARI 200mg + MTX	0.35411	0.13779	1.00299
ETN + MTX	SARI 200mg + MTX	1.8731	0.79232	4.72029
SARI 200mg + MTX	SARI 200mg + MTX	1	1	1
CZP + MTX	SARI 200mg + MTX	1.36106	0.61802	3.1824
PBO + MTX	CZP + MTX	0.16823	0.08317	0.32058
**BARI 4mg + MTX**	**CZP + MTX**	**0.85134**	**0.39286**	**1.81181**
ADA 40mg + MTX	CZP + MTX	0.79352	0.38054	1.6363
ABA 10mg + MTX	CZP + MTX	0.59355	0.27113	1.2395
ABA SUBCUT + MTX	CZP + MTX	0.78629	0.34452	1.79061
IFX 3mg + MTX	CZP + MTX	0.58575	0.26757	1.20265
RTX 1000mg + MTX	CZP + MTX	0.55735	0.2471	1.23824
GOL 50mg + MTX	CZP + MTX	0.58257	0.26668	1.24657
cDMARD + MTX	CZP + MTX	0.26202	0.08877	0.77946
ETN + MTX	CZP + MTX	1.38891	0.50674	3.70408
SARI 200mg + MTX	CZP + MTX	0.73472	0.31423	1.61807
CZP + MTX	CZP + MTX	1	1	1

Abbreviations: ABA abatacept; ADA adalimumab; BARI baricitinib; ETN etanercept; GOL golimumab; IFX infliximab; MTX methotrexate; PBO placebo; RTX rituximab; SARI sarilumab; SUBCUT subcutaneous.

[1]statistically significantly favouring BARI.

Odds ratios >1 are in favour of Treatment 1; and odds ratios <1 are in favour of Treatment 2.

9.2.3. *ACR70 Response*

Table S17 Primary Analysis: Relative treatment effect of pairwise comparisons expressed as Post. Median odds ratios (with 95% CrIs) – ACR70 response at week 24: MTX-IR (Simultaneous Random-effects model)

Treatment 1	Treatment 2	Median OR	95% CrI Lower	95% CrI Upper
PBO + MTX	PBO + MTX	1	1	1
**BARI 4mg + MTX[1]**	**PBO + MTX**	**5.99949**	**3.89412**	**9.43604**
ADA 40mg + MTX	PBO + MTX	4.45311	3.14472	6.53227
ABA 10mg + MTX	PBO + MTX	3.15344	2.0251	5.06409
ABA SUBCUT + MTX	PBO + MTX	4.25696	2.40728	7.72965
IFX 3mg + MTX	PBO + MTX	3.5556	2.26623	5.74016
RTX 1000mg + MTX	PBO + MTX	2.6467	1.4103	5.11074
GOL 50mg + MTX	PBO + MTX	4.41461	2.46602	7.99231
cDMARD + MTX	PBO + MTX	1.57919	0.46192	6.06897
ETN + MTX	PBO + MTX	6.56235	2.2271	22.95701
SARI 200mg + MTX	PBO + MTX	4.27001	2.69688	6.77139
CZP + MTX	PBO + MTX	5.76595	2.33877	16.14244
PBO + MTX	BARI 4mg + MTX	0.16668	0.10598	0.2568
BARI 4mg + MTX	BARI 4mg + MTX	1	1	1
ADA 40mg + MTX	BARI 4mg + MTX	0.73854	0.4985	1.1517
ABA 10mg + MTX	BARI 4mg + MTX	0.52596	0.2808	0.98767
ABA SUBCUT + MTX	BARI 4mg + MTX	0.70744	0.38761	1.34833
IFX 3mg + MTX	BARI 4mg + MTX	0.59242	0.31427	1.12215
RTX 1000mg + MTX	BARI 4mg + MTX	0.44083	0.20669	0.96924
GOL 50mg + MTX	BARI 4mg + MTX	0.73606	0.35154	1.54149
cDMARD + MTX	BARI 4mg + MTX	0.26297	0.07359	1.09184
ETN + MTX	BARI 4mg + MTX	1.09068	0.35497	4.12043
SARI 200mg + MTX	BARI 4mg + MTX	0.70946	0.37587	1.33512
CZP + MTX	BARI 4mg + MTX	0.96564	0.35456	2.95602
PBO + MTX	ADA 40mg + MTX	0.22456	0.15309	0.31799
**BARI 4mg + MTX**	**ADA 40mg + MTX**	**1.35402**	**0.86828**	**2.006**
ADA 40mg + MTX	ADA 40mg + MTX	1	1	1
ABA 10mg + MTX	ADA 40mg + MTX	0.70794	0.39466	1.25806
ABA SUBCUT + MTX	ADA 40mg + MTX	0.95703	0.59666	1.5291
IFX 3mg + MTX	ADA 40mg + MTX	0.80106	0.44609	1.42484
RTX 1000mg + MTX	ADA 40mg + MTX	0.59414	0.28836	1.23561
GOL 50mg + MTX	ADA 40mg + MTX	0.99195	0.49813	1.95006
cDMARD + MTX	ADA 40mg + MTX	0.35102	0.10245	1.40869
ETN + MTX	ADA 40mg + MTX	1.46089	0.48838	5.34211
SARI 200mg + MTX	ADA 40mg + MTX	0.96112	0.51901	1.68695
CZP + MTX	ADA 40mg + MTX	1.29341	0.48886	3.84633
PBO + MTX	ABA 10mg + MTX	0.31711	0.19747	0.4938
**BARI 4mg + MTX[1]**	**ABA 10mg + MTX**	**1.90129**	**1.01248**	**3.56128**
ADA 40mg + MTX	ABA 10mg + MTX	1.41255	0.79487	2.53383
ABA 10mg + MTX	ABA 10mg + MTX	1	1	1
ABA SUBCUT + MTX	ABA 10mg + MTX	1.34928	0.64478	2.83265
IFX 3mg + MTX	ABA 10mg + MTX	1.12749	0.68179	1.85542
RTX 1000mg + MTX	ABA 10mg + MTX	0.83686	0.38951	1.84333
GOL 50mg + MTX	ABA 10mg + MTX	1.39106	0.67501	2.91992

Treatment 1	Treatment 2	Median OR	95% CrI Lower	95% CrI Upper
cDMARD + MTX	ABA 10mg + MTX	0.50076	0.13904	2.01995
ETN + MTX	ABA 10mg + MTX	2.0884	0.65862	7.50109
SARI 200mg + MTX	ABA 10mg + MTX	1.35247	0.7013	2.53506
CZP + MTX	ABA 10mg + MTX	1.82239	0.65664	5.58319
PBO + MTX	ABA SUBCUT + MTX	0.23491	0.12937	0.41541
**BARI 4mg + MTX**	**ABA SUBCUT + MTX**	**1.41355**	**0.74166**	**2.57992**
ADA 40mg + MTX	ABA SUBCUT + MTX	1.0449	0.65398	1.676
ABA 10mg + MTX	ABA SUBCUT + MTX	0.74114	0.35303	1.55091
ABA SUBCUT + MTX	ABA SUBCUT + MTX	1	1	1
IFX 3mg + MTX	ABA SUBCUT + MTX	0.83475	0.39536	1.75185
RTX 1000mg + MTX	ABA SUBCUT + MTX	0.62109	0.26392	1.48116
GOL 50mg + MTX	ABA SUBCUT + MTX	1.0364	0.45598	2.33588
cDMARD + MTX	ABA SUBCUT + MTX	0.36738	0.10039	1.57529
ETN + MTX	ABA SUBCUT + MTX	1.52324	0.47262	5.88766
SARI 200mg + MTX	ABA SUBCUT + MTX	1.00238	0.46875	2.07595
CZP + MTX	ABA SUBCUT + MTX	1.36253	0.46172	4.38882
PBO + MTX	IFX 3mg + MTX	0.28125	0.17421	0.44126
**BARI 4mg + MTX**	**IFX 3mg + MTX**	**1.68798**	**0.89115**	**3.18201**
ADA 40mg + MTX	IFX 3mg + MTX	1.24835	0.70184	2.24169
ABA 10mg + MTX	IFX 3mg + MTX	0.88692	0.53896	1.46673
ABA SUBCUT + MTX	IFX 3mg + MTX	1.19797	0.57083	2.52931
IFX 3mg + MTX	IFX 3mg + MTX	1	1	1
RTX 1000mg + MTX	IFX 3mg + MTX	0.74288	0.35062	1.63201
GOL 50mg + MTX	IFX 3mg + MTX	1.23824	0.59646	2.59212
cDMARD + MTX	IFX 3mg + MTX	0.44371	0.12165	1.80291
ETN + MTX	IFX 3mg + MTX	1.84599	0.57245	6.75431
SARI 200mg + MTX	IFX 3mg + MTX	1.20341	0.61932	2.28153
CZP + MTX	IFX 3mg + MTX	1.63021	0.58152	5.07075
PBO + MTX	RTX 1000mg + MTX	0.37783	0.19567	0.70907
**BARI 4mg + MTX[1]**	**RTX 1000mg + MTX**	**2.26847**	**1.03174**	**4.83813**
ADA 40mg + MTX	RTX 1000mg + MTX	1.6831	0.80932	3.46788
ABA 10mg + MTX	RTX 1000mg + MTX	1.19494	0.5425	2.5673
ABA SUBCUT + MTX	RTX 1000mg + MTX	1.61008	0.67515	3.78907
IFX 3mg + MTX	RTX 1000mg + MTX	1.34611	0.61274	2.85205
RTX 1000mg + MTX	RTX 1000mg + MTX	1	1	1
GOL 50mg + MTX	RTX 1000mg + MTX	1.65655	0.72573	3.87633
cDMARD + MTX	RTX 1000mg + MTX	0.59683	0.15583	2.50117
ETN + MTX	RTX 1000mg + MTX	2.50407	0.74393	9.38908
SARI 200mg + MTX	RTX 1000mg + MTX	1.61628	0.70886	3.52271
CZP + MTX	RTX 1000mg + MTX	2.18702	0.72178	6.99874
PBO + MTX	GOL 50mg + MTX	0.22652	0.12512	0.40551
**BARI 4mg + MTX**	**GOL 50mg + MTX**	**1.35859**	**0.64872**	**2.84465**
ADA 40mg + MTX	GOL 50mg + MTX	1.00812	0.5128	2.0075
ABA 10mg + MTX	GOL 50mg + MTX	0.71888	0.34248	1.48147
ABA SUBCUT + MTX	GOL 50mg + MTX	0.96488	0.4281	2.19309
IFX 3mg + MTX	GOL 50mg + MTX	0.8076	0.38579	1.67656
RTX 1000mg + MTX	GOL 50mg + MTX	0.60366	0.25798	1.37793
GOL 50mg + MTX	GOL 50mg + MTX	1	1	1
cDMARD + MTX	GOL 50mg + MTX	0.36092	0.0958	1.46571
ETN + MTX	GOL 50mg + MTX	1.49195	0.44116	5.45612
SARI 200mg + MTX	GOL 50mg + MTX	0.97066	0.454	2.05147
CZP + MTX	GOL 50mg + MTX	1.31444	0.45339	4.13035
PBO + MTX	cDMARD + MTX	0.63324	0.16477	2.16487
**BARI 4mg + MTX**	**cDMARD + MTX**	**3.80276**	**0.91589**	**13.58789**
ADA 40mg + MTX	cDMARD + MTX	2.84883	0.70988	9.76039
ABA 10mg + MTX	cDMARD + MTX	1.99698	0.49506	7.19239

Treatment 1	Treatment 2	Median OR	95% CrI Lower	95% CrI Upper
ABA SUBCUT + MTX	cDMARD + MTX	2.72195	0.6348	9.9614
IFX 3mg + MTX	cDMARD + MTX	2.25373	0.55466	8.22048
RTX 1000mg + MTX	cDMARD + MTX	1.67551	0.39981	6.41705
GOL 50mg + MTX	cDMARD + MTX	2.77069	0.68226	10.43877
cDMARD + MTX	cDMARD + MTX	1	1	1
ETN + MTX	cDMARD + MTX	4.14899	2.21399	8.21638
SARI 200mg + MTX	cDMARD + MTX	2.70861	0.65193	10.05903
CZP + MTX	cDMARD + MTX	3.69516	0.76806	17.18589
PBO + MTX	ETN + MTX	0.15238	0.04356	0.44901
**BARI 4mg + MTX**	**ETN + MTX**	**0.91686**	**0.24269**	**2.81715**
ADA 40mg + MTX	ETN + MTX	0.68451	0.18719	2.04757
ABA 10mg + MTX	ETN + MTX	0.47883	0.13331	1.51832
ABA SUBCUT + MTX	ETN + MTX	0.6565	0.16985	2.11585
IFX 3mg + MTX	ETN + MTX	0.54171	0.14805	1.74687
RTX 1000mg + MTX	ETN + MTX	0.39935	0.10651	1.34421
GOL 50mg + MTX	ETN + MTX	0.67027	0.18328	2.26675
cDMARD + MTX	ETN + MTX	0.24102	0.12171	0.45167
ETN + MTX	ETN + MTX	1	1	1
SARI 200mg + MTX	ETN + MTX	0.65197	0.17464	2.08737
CZP + MTX	ETN + MTX	0.87477	0.20694	3.67255
PBO + MTX	SARI 200mg + MTX	0.23419	0.14768	0.3708
**BARI 4mg + MTX**	**SARI 200mg + MTX**	**1.40953**	**0.74899**	**2.66048**
ADA 40mg + MTX	SARI 200mg + MTX	1.04045	0.59279	1.92674
ABA 10mg + MTX	SARI 200mg + MTX	0.73939	0.39447	1.42592
ABA SUBCUT + MTX	SARI 200mg + MTX	0.99762	0.48171	2.13332
IFX 3mg + MTX	SARI 200mg + MTX	0.83097	0.4383	1.61466
RTX 1000mg + MTX	SARI 200mg + MTX	0.6187	0.28387	1.41072
GOL 50mg + MTX	SARI 200mg + MTX	1.03022	0.48746	2.20266
cDMARD + MTX	SARI 200mg + MTX	0.36919	0.09941	1.53391
ETN + MTX	SARI 200mg + MTX	1.53381	0.47907	5.72597
SARI 200mg + MTX	SARI 200mg + MTX	1	1	1
CZP + MTX	SARI 200mg + MTX	1.35235	0.48489	4.29779
PBO + MTX	CZP + MTX	0.17343	0.06195	0.42758
**BARI 4mg + MTX**	**CZP + MTX**	**1.03558**	**0.33829**	**2.8204**
ADA 40mg + MTX	CZP + MTX	0.77315	0.25999	2.04557
ABA 10mg + MTX	CZP + MTX	0.54873	0.17911	1.52291
ABA SUBCUT + MTX	CZP + MTX	0.73393	0.22785	2.16582
IFX 3mg + MTX	CZP + MTX	0.61342	0.19721	1.71963
RTX 1000mg + MTX	CZP + MTX	0.45724	0.14288	1.38546
GOL 50mg + MTX	CZP + MTX	0.76078	0.24211	2.20561
cDMARD + MTX	CZP + MTX	0.27062	0.05819	1.30198
ETN + MTX	CZP + MTX	1.14316	0.27229	4.83225
SARI 200mg + MTX	CZP + MTX	0.73945	0.23268	2.06233
CZP + MTX	CZP + MTX	1	1	1

Abbreviations: ABA abatacept; ADA adalimumab; BARI baricitinib; ETN etanercept; GOL golimumab; IFX infliximab; MTX methotrexate; PBO placebo; RTX rituximab; SARI sarilumab; SUBCUT subcutaneous.

[1]statistically significantly favouring BARI.

Odds ratios >1 are in favour of Treatment 1; and odds ratios <1 are in favour of Treatment 2.

10. Results – Sensitivity Analyses

10.1. Baseline-risk adjustment

Figure S2 Response rate in PBO+MTX arm vs. ln (Risk Ratio) - Primary analysis

(A) ACR20

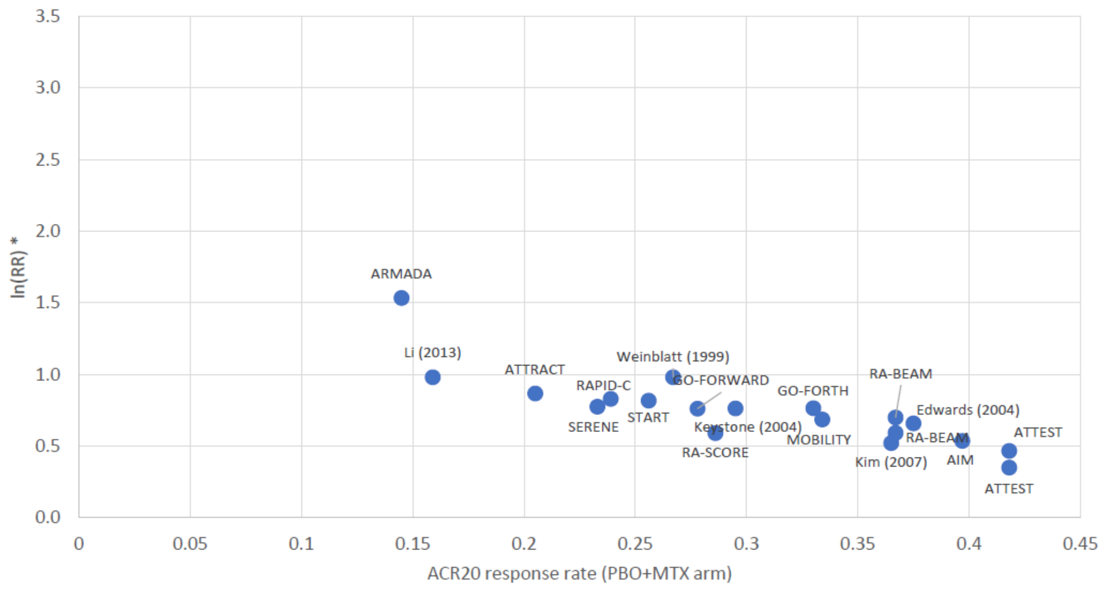

(B) ACR50

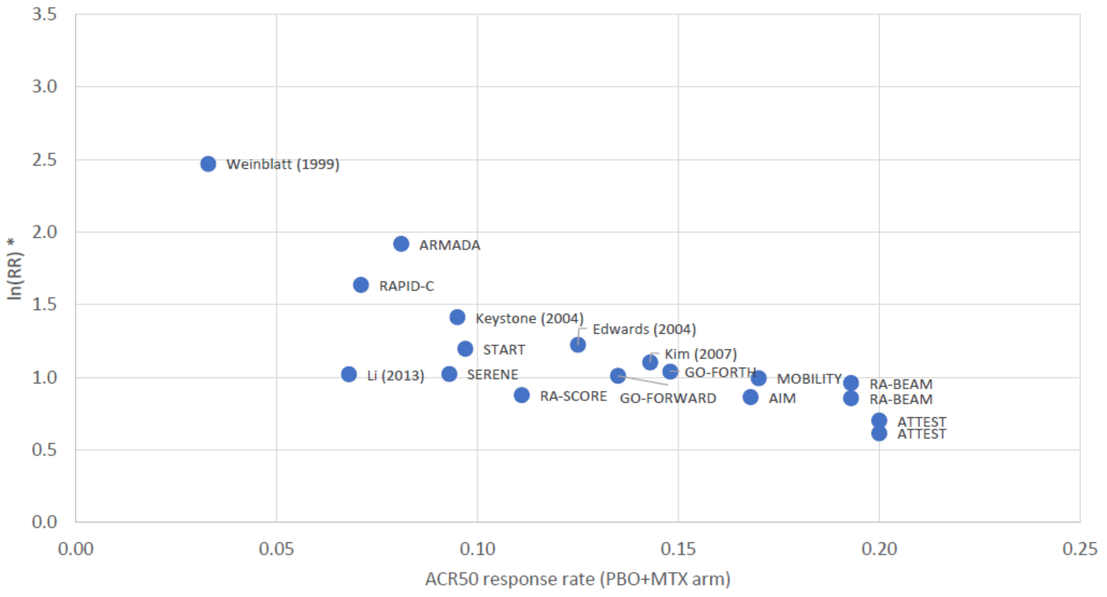

(C) ACR70

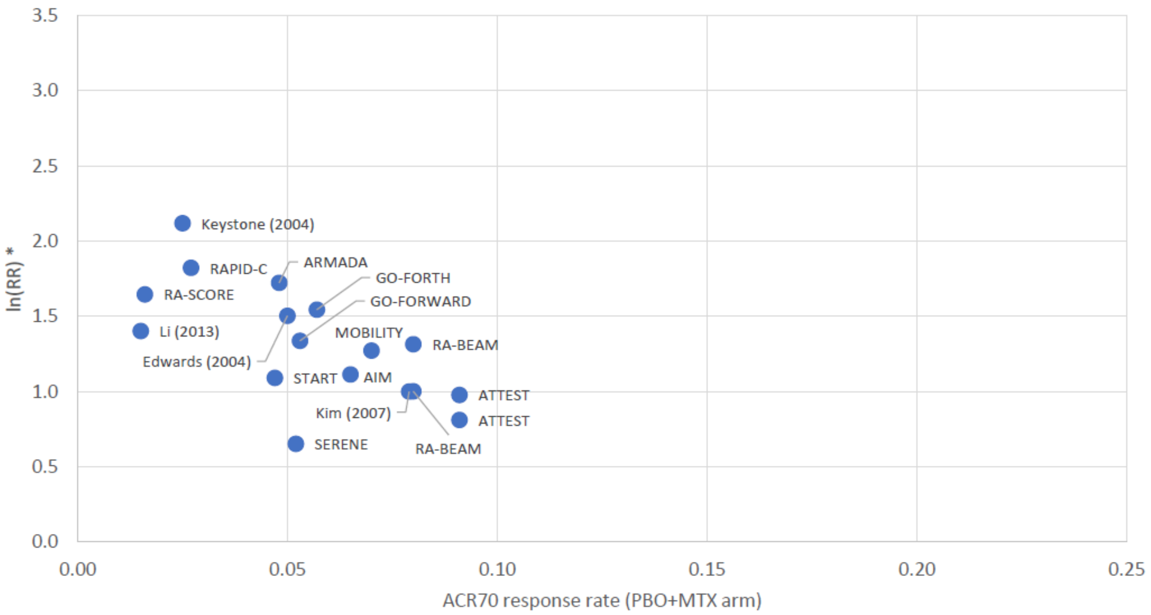

*Risk ratio of bDMARD/tsDMARD comparator over PBO+MTX arm in respective trial

Note: size of trials is not considered in these figures

10.1.1. ACR20 Response

Table S18 Baseline Risk-adjustment (Primary analysis): Relative treatment effect of pairwise comparisons expressed as Post. Median odds ratios (with 95% CrIs)

- ACR20 response at week 24: MTX-IR (Simultaneous Fixed-effects model)

Treatment 1	Treatment 2	Median OR	95% CrI Lower	95% CrI Upper
PBO + MTX	PBO + MTX	1	1	1
**BARI 4mg + MTX[1]**	**PBO + MTX**	**5.61288**	**4.22089**	**7.23663**
ADA 40mg + MTX	PBO + MTX	4.15006	3.32835	4.99813
ABA 10mg + MTX	PBO + MTX	3.90596	2.90983	5.02336
ABA SUBCUT + MTX	PBO + MTX	4.26135	2.94373	6.03455
IFX 3mg + MTX	PBO + MTX	3.26426	2.63732	4.03507
RTX 1000mg + MTX	PBO + MTX	3.1634	2.3469	4.37628
GOL 50mg + MTX	PBO + MTX	3.56656	2.67969	4.93361
cDMARD + MTX	PBO + MTX	2.03623	1.12003	3.44583
ETN + MTX	PBO + MTX	9.32367	5.63475	14.84963
SARI 200mg + MTX	PBO + MTX	4.32623	3.07169	5.86031
CZP + MTX	PBO + MTX	3.35824	2.36959	5.04304
PBO + MTX	BARI 4mg + MTX	0.17816	0.13819	0.23692
BARI 4mg + MTX	BARI 4mg + MTX	1	1	1
ADA 40mg + MTX	BARI 4mg + MTX	0.73874	0.56488	0.96999
ABA 10mg + MTX	BARI 4mg + MTX	0.69594	0.49344	0.97378
ABA SUBCUT + MTX	BARI 4mg + MTX	0.75933	0.51022	1.13152
IFX 3mg + MTX	BARI 4mg + MTX	0.58211	0.42048	0.82588
RTX 1000mg + MTX	BARI 4mg + MTX	0.56365	0.37878	0.88178
GOL 50mg + MTX	BARI 4mg + MTX	0.63635	0.42577	0.99869
cDMARD + MTX	BARI 4mg + MTX	0.36143	0.19797	0.65755
ETN + MTX	BARI 4mg + MTX	1.65451	0.97959	2.84056
SARI 200mg + MTX	BARI 4mg + MTX	0.7695	0.52257	1.1421
CZP + MTX	BARI 4mg + MTX	0.59792	0.38997	1.01254
PBO + MTX	ADA 40mg + MTX	0.24096	0.20007	0.30045
**BARI 4mg + MTX[1]**	**ADA 40mg + MTX**	**1.35366**	**1.03094**	**1.7703**
ADA 40mg + MTX	ADA 40mg + MTX	1	1	1
ABA 10mg + MTX	ADA 40mg + MTX	0.94385	0.68706	1.26349
ABA SUBCUT + MTX	ADA 40mg + MTX	1.02832	0.75524	1.40491
IFX 3mg + MTX	ADA 40mg + MTX	0.78766	0.60071	1.06449
RTX 1000mg + MTX	ADA 40mg + MTX	0.76372	0.53758	1.14076
GOL 50mg + MTX	ADA 40mg + MTX	0.8605	0.60831	1.29224
cDMARD + MTX	ADA 40mg + MTX	0.49033	0.27293	0.8594
ETN + MTX	ADA 40mg + MTX	2.24396	1.35757	3.71403
SARI 200mg + MTX	ADA 40mg + MTX	1.04386	0.72554	1.49221
CZP + MTX	ADA 40mg + MTX	0.80847	0.54952	1.3115
PBO + MTX	ABA 10mg + MTX	0.25602	0.19907	0.34366
**BARI 4mg + MTX[1]**	**ABA 10mg + MTX**	**1.4369**	**1.02692**	**2.02659**
ADA 40mg + MTX	ABA 10mg + MTX	1.05949	0.79146	1.45548
ABA 10mg + MTX	ABA 10mg + MTX	1	1	1
ABA SUBCUT + MTX	ABA 10mg + MTX	1.08921	0.72486	1.66977
IFX 3mg + MTX	ABA 10mg + MTX	0.83738	0.61741	1.17258
RTX 1000mg + MTX	ABA 10mg + MTX	0.8097	0.54452	1.28672
GOL 50mg + MTX	ABA 10mg + MTX	0.91286	0.60968	1.45515
cDMARD + MTX	ABA 10mg + MTX	0.51946	0.28586	0.94094
ETN + MTX	ABA 10mg + MTX	2.37521	1.43211	4.10002

Treatment 1	Treatment 2	Median OR	95% CrI Lower	95% CrI Upper
SARI 200mg + MTX	ABA 10mg + MTX	1.10663	0.75606	1.64792
CZP + MTX	ABA 10mg + MTX	0.85829	0.56218	1.47782
PBO + MTX	ABA SUBCUT + MTX	0.23467	0.16571	0.33971
**BARI 4mg + MTX**	**ABA SUBCUT + MTX**	**1.31695**	**0.88377**	**1.95993**
ADA 40mg + MTX	ABA SUBCUT + MTX	0.97246	0.71179	1.32408
ABA 10mg + MTX	ABA SUBCUT + MTX	0.9181	0.59889	1.37957
ABA SUBCUT + MTX	ABA SUBCUT + MTX	1	1	1
IFX 3mg + MTX	ABA SUBCUT + MTX	0.76704	0.51302	1.16374
RTX 1000mg + MTX	ABA SUBCUT + MTX	0.74287	0.46861	1.22628
GOL 50mg + MTX	ABA SUBCUT + MTX	0.83832	0.53145	1.39131
cDMARD + MTX	ABA SUBCUT + MTX	0.4761	0.24833	0.89536
ETN + MTX	ABA SUBCUT + MTX	2.18015	1.22487	3.89872
SARI 200mg + MTX	ABA SUBCUT + MTX	1.01283	0.63748	1.6072
CZP + MTX	ABA SUBCUT + MTX	0.78779	0.48485	1.38824
PBO + MTX	IFX 3mg + MTX	0.30635	0.24783	0.37917
**BARI 4mg + MTX[1]**	**IFX 3mg + MTX**	**1.71789**	**1.21083**	**2.37822**
ADA 40mg + MTX	IFX 3mg + MTX	1.26959	0.93942	1.6647
ABA 10mg + MTX	IFX 3mg + MTX	1.1942	0.85282	1.61968
ABA SUBCUT + MTX	IFX 3mg + MTX	1.30372	0.8593	1.94925
IFX 3mg + MTX	IFX 3mg + MTX	1	1	1
RTX 1000mg + MTX	IFX 3mg + MTX	0.96869	0.67567	1.42289
GOL 50mg + MTX	IFX 3mg + MTX	1.09025	0.77195	1.6069
cDMARD + MTX	IFX 3mg + MTX	0.62351	0.33251	1.0934
ETN + MTX	IFX 3mg + MTX	2.84925	1.66636	4.73111
SARI 200mg + MTX	IFX 3mg + MTX	1.32428	0.88717	1.90837
CZP + MTX	IFX 3mg + MTX	1.0293	0.68708	1.62337
PBO + MTX	RTX 1000mg + MTX	0.31612	0.2285	0.42609
**BARI 4mg + MTX[1]**	**RTX 1000mg + MTX**	**1.77415**	**1.13407**	**2.64006**
ADA 40mg + MTX	RTX 1000mg + MTX	1.30938	0.87661	1.86017
ABA 10mg + MTX	RTX 1000mg + MTX	1.23502	0.77717	1.83647
ABA SUBCUT + MTX	RTX 1000mg + MTX	1.34612	0.81547	2.13395
IFX 3mg + MTX	RTX 1000mg + MTX	1.03232	0.70279	1.48
RTX 1000mg + MTX	RTX 1000mg + MTX	1	1	1
GOL 50mg + MTX	RTX 1000mg + MTX	1.12652	0.75278	1.71388
cDMARD + MTX	RTX 1000mg + MTX	0.6438	0.32245	1.17526
ETN + MTX	RTX 1000mg + MTX	2.94537	1.61273	5.03143
SARI 200mg + MTX	RTX 1000mg + MTX	1.3681	0.83679	2.09181
CZP + MTX	RTX 1000mg + MTX	1.06379	0.66779	1.71125
PBO + MTX	GOL 50mg + MTX	0.28038	0.20269	0.37318
**BARI 4mg + MTX[1]**	**GOL 50mg + MTX**	**1.57146**	**1.00132**	**2.34868**
ADA 40mg + MTX	GOL 50mg + MTX	1.16212	0.77385	1.64389
ABA 10mg + MTX	GOL 50mg + MTX	1.09546	0.68721	1.64019
ABA SUBCUT + MTX	GOL 50mg + MTX	1.19286	0.71875	1.88164
IFX 3mg + MTX	GOL 50mg + MTX	0.91722	0.62232	1.29542
RTX 1000mg + MTX	GOL 50mg + MTX	0.88769	0.58347	1.32842
GOL 50mg + MTX	GOL 50mg + MTX	1	1	1
cDMARD + MTX	GOL 50mg + MTX	0.57106	0.28406	1.03623
ETN + MTX	GOL 50mg + MTX	2.61746	1.41488	4.42977
SARI 200mg + MTX	GOL 50mg + MTX	1.21249	0.73969	1.86216
CZP + MTX	GOL 50mg + MTX	0.94586	0.59217	1.49091
PBO + MTX	cDMARD + MTX	0.4911	0.29021	0.89283
**BARI 4mg + MTX[1]**	**cDMARD + MTX**	**2.76679**	**1.5208**	**5.05114**
ADA 40mg + MTX	cDMARD + MTX	2.03943	1.1636	3.66398
ABA 10mg + MTX	cDMARD + MTX	1.92509	1.06277	3.49822
ABA SUBCUT + MTX	cDMARD + MTX	2.1004	1.11687	4.02687
IFX 3mg + MTX	cDMARD + MTX	1.60383	0.91458	3.0074

Treatment 1	Treatment 2	Median OR	95% CrI Lower	95% CrI Upper
RTX 1000mg + MTX	cDMARD + MTX	1.55329	0.85088	3.10123
GOL 50mg + MTX	cDMARD + MTX	1.75112	0.96503	3.52043
cDMARD + MTX	cDMARD + MTX	1	1	1
ETN + MTX	cDMARD + MTX	4.59128	2.96425	7.1646
SARI 200mg + MTX	cDMARD + MTX	2.13044	1.14463	4.02233
CZP + MTX	cDMARD + MTX	1.64639	0.88811	3.46441
PBO + MTX	ETN + MTX	0.10725	0.06734	0.17747
**BARI 4mg + MTX**	**ETN + MTX**	**0.60441**	**0.35204**	**1.02084**
ADA 40mg + MTX	ETN + MTX	0.44564	0.26925	0.73661
ABA 10mg + MTX	ETN + MTX	0.42101	0.2439	0.69827
ABA SUBCUT + MTX	ETN + MTX	0.45868	0.25649	0.81641
IFX 3mg + MTX	ETN + MTX	0.35097	0.21137	0.60011
RTX 1000mg + MTX	ETN + MTX	0.33952	0.19875	0.62007
GOL 50mg + MTX	ETN + MTX	0.38205	0.22575	0.70678
cDMARD + MTX	ETN + MTX	0.2178	0.13958	0.33735
ETN + MTX	ETN + MTX	1	1	1
SARI 200mg + MTX	ETN + MTX	0.4658	0.26152	0.81517
CZP + MTX	ETN + MTX	0.35983	0.2043	0.70098
PBO + MTX	SARI 200mg + MTX	0.23115	0.17064	0.32555
**BARI 4mg + MTX**	**SARI 200mg + MTX**	**1.29954**	**0.87558**	**1.91363**
ADA 40mg + MTX	SARI 200mg + MTX	0.95798	0.67015	1.37828
ABA 10mg + MTX	SARI 200mg + MTX	0.90365	0.60683	1.32264
ABA SUBCUT + MTX	SARI 200mg + MTX	0.98733	0.6222	1.56867
IFX 3mg + MTX	SARI 200mg + MTX	0.75513	0.52401	1.12719
RTX 1000mg + MTX	SARI 200mg + MTX	0.73094	0.47805	1.19504
GOL 50mg + MTX	SARI 200mg + MTX	0.82475	0.53701	1.35191
cDMARD + MTX	SARI 200mg + MTX	0.46939	0.24861	0.87365
ETN + MTX	SARI 200mg + MTX	2.14683	1.22674	3.82379
SARI 200mg + MTX	SARI 200mg + MTX	1	1	1
CZP + MTX	SARI 200mg + MTX	0.77682	0.48954	1.35288
PBO + MTX	CZP + MTX	0.29777	0.19829	0.42201
**BARI 4mg + MTX**	**CZP + MTX**	**1.67246**	**0.98761**	**2.56433**
ADA 40mg + MTX	CZP + MTX	1.23691	0.76248	1.81978
ABA 10mg + MTX	CZP + MTX	1.1651	0.67667	1.77877
ABA SUBCUT + MTX	CZP + MTX	1.26938	0.72034	2.06251
IFX 3mg + MTX	CZP + MTX	0.97153	0.616	1.45543
RTX 1000mg + MTX	CZP + MTX	0.94004	0.58437	1.49748
GOL 50mg + MTX	CZP + MTX	1.05724	0.67073	1.68872
cDMARD + MTX	CZP + MTX	0.60739	0.28865	1.12599
ETN + MTX	CZP + MTX	2.77906	1.42657	4.89484
SARI 200mg + MTX	CZP + MTX	1.2873	0.73916	2.04272
CZP + MTX	CZP + MTX	1	1	1

Abbreviations: ABA abatacept; ADA adalimumab; BARI baricitinib; ETN etanercept; GOL golimumab; IFX infliximab; MTX methotrexate; PBO placebo; RTX rituximab; SARI sarilumab; SUBCUT subcutaneous.

[1]statistically significantly favouring BARI.

Odds ratios >1 are in favour of Treatment 1; and odds ratios <1 are in favour of Treatment 2.

10.1.2. ACR50 Response

Table S19 Baseline Risk-adjustment (Primary analysis): Relative treatment effect of pairwise comparisons expressed as Post. Median odds ratios (with 95% CrIs)

– ACR50 response at week 24: MTX-IR (Simultaneous Fixed-effects model)

Treatment 1	Treatment 2	Median OR	95% CrI Lower	95% CrI Upper
PBO + MTX	PBO + MTX	1	1	1
**BARI 4mg + MTX[1]**	**PBO + MTX**	**3.39252**	**2.23534**	**4.78553**
ADA 40mg + MTX	PBO + MTX	3.19596	2.16428	4.34566
ABA 10mg + MTX	PBO + MTX	2.72293	1.9526	3.61762
ABA SUBCUT + MTX	PBO + MTX	3.14516	2.11087	4.60369
IFX 3mg + MTX	PBO + MTX	2.59997	1.90052	3.4192
RTX 1000mg + MTX	PBO + MTX	2.37222	1.73028	3.34283
GOL 50mg + MTX	PBO + MTX	2.51628	1.83186	3.47054
cDMARD + MTX	PBO + MTX	1.43807	0.80879	2.4282
ETN + MTX	PBO + MTX	4.1003	2.39357	7.05307
SARI 200mg + MTX	PBO + MTX	3.69429	2.21377	6.05666
CZP + MTX	PBO + MTX	3.25418	2.1391	5.16891
PBO + MTX	BARI 4mg + MTX	0.29477	0.20896	0.44736
BARI 4mg + MTX	BARI 4mg + MTX	1	1	1
ADA 40mg + MTX	BARI 4mg + MTX	0.94328	0.80538	1.08759
ABA 10mg + MTX	BARI 4mg + MTX	0.81128	0.63441	0.98284
ABA SUBCUT + MTX	BARI 4mg + MTX	0.93838	0.72989	1.17049
IFX 3mg + MTX	BARI 4mg + MTX	0.7751	0.59546	0.96274
RTX 1000mg + MTX	BARI 4mg + MTX	0.713	0.5079	0.95438
GOL 50mg + MTX	BARI 4mg + MTX	0.75471	0.5517	0.97694
cDMARD + MTX	BARI 4mg + MTX	0.43134	0.22385	0.73805
ETN + MTX	BARI 4mg + MTX	1.20527	0.9164	1.73047
SARI 200mg + MTX	BARI 4mg + MTX	1.10683	0.78969	1.43133
CZP + MTX	BARI 4mg + MTX	0.96726	0.71682	1.36975
PBO + MTX	ADA 40mg + MTX	0.3129	0.23011	0.46205
**BARI 4mg + MTX**	**ADA 40mg + MTX**	**1.06013**	**0.91947**	**1.24164**
ADA 40mg + MTX	ADA 40mg + MTX	1	1	1
ABA 10mg + MTX	ADA 40mg + MTX	0.86217	0.68665	1.02952
ABA SUBCUT + MTX	ADA 40mg + MTX	0.99409	0.81842	1.18936
IFX 3mg + MTX	ADA 40mg + MTX	0.82232	0.64806	1.00096
RTX 1000mg + MTX	ADA 40mg + MTX	0.75747	0.55262	0.99765
GOL 50mg + MTX	ADA 40mg + MTX	0.80098	0.60288	1.01929
cDMARD + MTX	ADA 40mg + MTX	0.45926	0.24158	0.76967
ETN + MTX	ADA 40mg + MTX	1.28301	0.97642	1.83843
SARI 200mg + MTX	ADA 40mg + MTX	1.17196	0.83391	1.55956
CZP + MTX	ADA 40mg + MTX	1.02483	0.77883	1.44433
PBO + MTX	ABA 10mg + MTX	0.36725	0.27643	0.51214
**BARI 4mg + MTX[1]**	**ABA 10mg + MTX**	**1.23262**	**1.01746**	**1.57627**
ADA 40mg + MTX	ABA 10mg + MTX	1.15986	0.97132	1.45634
ABA 10mg + MTX	ABA 10mg + MTX	1	1	1
ABA SUBCUT + MTX	ABA 10mg + MTX	1.15154	0.89718	1.54095
IFX 3mg + MTX	ABA 10mg + MTX	0.95634	0.77232	1.18448
RTX 1000mg + MTX	ABA 10mg + MTX	0.87992	0.64935	1.19336

Treatment 1	Treatment 2	Median OR	95% CrI Lower	95% CrI Upper
GOL 50mg + MTX	ABA 10mg + MTX	0.93022	0.70333	1.23406
cDMARD + MTX	ABA 10mg + MTX	0.53595	0.28639	0.89636
ETN + MTX	ABA 10mg + MTX	1.49181	1.10017	2.29094
SARI 200mg + MTX	ABA 10mg + MTX	1.36199	0.96175	1.90528
CZP + MTX	ABA 10mg + MTX	1.18783	0.89798	1.77349
PBO + MTX	ABA SUBCUT + MTX	0.31795	0.21722	0.47374
**BARI 4mg + MTX**	**ABA SUBCUT + MTX**	**1.06566**	**0.85434**	**1.37007**
ADA 40mg + MTX	ABA SUBCUT + MTX	1.00595	0.84079	1.22187
ABA 10mg + MTX	ABA SUBCUT + MTX	0.8684	0.64895	1.1146
ABA SUBCUT + MTX	ABA SUBCUT + MTX	1	1	1
IFX 3mg + MTX	ABA SUBCUT + MTX	0.82928	0.61312	1.08021
RTX 1000mg + MTX	ABA SUBCUT + MTX	0.76379	0.53121	1.05268
GOL 50mg + MTX	ABA SUBCUT + MTX	0.80822	0.57542	1.08612
cDMARD + MTX	ABA SUBCUT + MTX	0.46273	0.2373	0.79801
ETN + MTX	ABA SUBCUT + MTX	1.29022	0.94438	1.94264
SARI 200mg + MTX	ABA SUBCUT + MTX	1.17643	0.80922	1.67194
CZP + MTX	ABA SUBCUT + MTX	1.03273	0.74783	1.51692
PBO + MTX	IFX 3mg + MTX	0.38462	0.29247	0.52617
**BARI 4mg + MTX[1]**	**IFX 3mg + MTX**	**1.29016**	**1.0387**	**1.67938**
ADA 40mg + MTX	IFX 3mg + MTX	1.21607	0.99904	1.54306
ABA 10mg + MTX	IFX 3mg + MTX	1.04565	0.84425	1.29479
ABA SUBCUT + MTX	IFX 3mg + MTX	1.20586	0.92575	1.63101
IFX 3mg + MTX	IFX 3mg + MTX	1	1	1
RTX 1000mg + MTX	IFX 3mg + MTX	0.92118	0.68464	1.23403
GOL 50mg + MTX	IFX 3mg + MTX	0.97325	0.74153	1.27497
cDMARD + MTX	IFX 3mg + MTX	0.56128	0.29676	0.92896
ETN + MTX	IFX 3mg + MTX	1.56284	1.13014	2.40814
SARI 200mg + MTX	IFX 3mg + MTX	1.42307	0.96456	2.09261
CZP + MTX	IFX 3mg + MTX	1.24673	0.9426	1.82252
PBO + MTX	RTX 1000mg + MTX	0.42155	0.29915	0.57794
**BARI 4mg + MTX[1]**	**RTX 1000mg + MTX**	**1.40253**	**1.0478**	**1.9689**
ADA 40mg + MTX	RTX 1000mg + MTX	1.32019	1.00236	1.80957
ABA 10mg + MTX	RTX 1000mg + MTX	1.13647	0.83797	1.54001
ABA SUBCUT + MTX	RTX 1000mg + MTX	1.30927	0.94996	1.88251
IFX 3mg + MTX	RTX 1000mg + MTX	1.08557	0.81035	1.46063
RTX 1000mg + MTX	RTX 1000mg + MTX	1	1	1
GOL 50mg + MTX	RTX 1000mg + MTX	1.0559	0.7711	1.45824
cDMARD + MTX	RTX 1000mg + MTX	0.61133	0.31561	1.02864
ETN + MTX	RTX 1000mg + MTX	1.70512	1.16625	2.72863
SARI 200mg + MTX	RTX 1000mg + MTX	1.54657	0.96731	2.45403
CZP + MTX	RTX 1000mg + MTX	1.35702	0.98572	2.04072
PBO + MTX	GOL 50mg + MTX	0.39741	0.28814	0.54589
**BARI 4mg + MTX[1]**	**GOL 50mg + MTX**	**1.32501**	**1.0236**	**1.81258**
ADA 40mg + MTX	GOL 50mg + MTX	1.24847	0.98107	1.65869
ABA 10mg + MTX	GOL 50mg + MTX	1.07501	0.81033	1.42181
ABA SUBCUT + MTX	GOL 50mg + MTX	1.23728	0.92071	1.73786
IFX 3mg + MTX	GOL 50mg + MTX	1.02749	0.78433	1.34856
RTX 1000mg + MTX	GOL 50mg + MTX	0.94706	0.68576	1.29685
GOL 50mg + MTX	GOL 50mg + MTX	1	1	1
cDMARD + MTX	GOL 50mg + MTX	0.57802	0.30135	0.96385
ETN + MTX	GOL 50mg + MTX	1.61243	1.12849	2.52955
SARI 200mg + MTX	GOL 50mg + MTX	1.46044	0.95247	2.26043
CZP + MTX	GOL 50mg + MTX	1.28368	0.94778	1.90323
PBO + MTX	cDMARD + MTX	0.69538	0.41183	1.23641
**BARI 4mg + MTX[1]**	**cDMARD + MTX**	**2.31837**	**1.35492**	**4.46736**
ADA 40mg + MTX	cDMARD + MTX	2.17741	1.29926	4.13937

Treatment 1	Treatment 2	Median OR	95% CrI Lower	95% CrI Upper
ABA 10mg + MTX	cDMARD + MTX	1.86585	1.11562	3.49176
ABA SUBCUT + MTX	cDMARD + MTX	2.16107	1.25311	4.21411
IFX 3mg + MTX	cDMARD + MTX	1.78165	1.07647	3.36978
RTX 1000mg + MTX	cDMARD + MTX	1.63577	0.97215	3.16842
GOL 50mg + MTX	cDMARD + MTX	1.73005	1.03751	3.31838
cDMARD + MTX	cDMARD + MTX	1	1	1
ETN + MTX	cDMARD + MTX	2.85382	1.71201	4.99605
SARI 200mg + MTX	cDMARD + MTX	2.56682	1.3512	5.12597
CZP + MTX	cDMARD + MTX	2.23345	1.29891	4.62453
PBO + MTX	ETN + MTX	0.24388	0.14178	0.41779
**BARI 4mg + MTX**	**ETN + MTX**	**0.82969**	**0.57788**	**1.09123**
ADA 40mg + MTX	ETN + MTX	0.77942	0.54394	1.02415
ABA 10mg + MTX	ETN + MTX	0.67033	0.4365	0.90895
ABA SUBCUT + MTX	ETN + MTX	0.77506	0.51476	1.0589
IFX 3mg + MTX	ETN + MTX	0.63986	0.41526	0.88485
RTX 1000mg + MTX	ETN + MTX	0.58647	0.36648	0.85745
GOL 50mg + MTX	ETN + MTX	0.62018	0.39533	0.88614
cDMARD + MTX	ETN + MTX	0.35041	0.20016	0.58411
ETN + MTX	ETN + MTX	1	1	1
SARI 200mg + MTX	ETN + MTX	0.92459	0.55741	1.24906
CZP + MTX	ETN + MTX	0.80195	0.5317	1.16588
PBO + MTX	SARI 200mg + MTX	0.27069	0.16511	0.45172
**BARI 4mg + MTX**	**SARI 200mg + MTX**	**0.90348**	**0.69865**	**1.26633**
ADA 40mg + MTX	SARI 200mg + MTX	0.85327	0.64121	1.19917
ABA 10mg + MTX	SARI 200mg + MTX	0.73422	0.52486	1.03977
ABA SUBCUT + MTX	SARI 200mg + MTX	0.85003	0.59811	1.23576
IFX 3mg + MTX	SARI 200mg + MTX	0.70271	0.47787	1.03674
RTX 1000mg + MTX	SARI 200mg + MTX	0.64659	0.40749	1.0338
GOL 50mg + MTX	SARI 200mg + MTX	0.68473	0.44239	1.0499
cDMARD + MTX	SARI 200mg + MTX	0.38959	0.19509	0.74008
ETN + MTX	SARI 200mg + MTX	1.08156	0.8006	1.79402
SARI 200mg + MTX	SARI 200mg + MTX	1	1	1
CZP + MTX	SARI 200mg + MTX	0.87537	0.5796	1.49622
PBO + MTX	CZP + MTX	0.3073	0.19346	0.46749
**BARI 4mg + MTX**	**CZP + MTX**	**1.03385**	**0.73006**	**1.39506**
ADA 40mg + MTX	CZP + MTX	0.97577	0.69236	1.28398
ABA 10mg + MTX	CZP + MTX	0.84187	0.56386	1.11361
ABA SUBCUT + MTX	CZP + MTX	0.96831	0.65923	1.3372
IFX 3mg + MTX	CZP + MTX	0.8021	0.54869	1.06089
RTX 1000mg + MTX	CZP + MTX	0.73691	0.49002	1.01449
GOL 50mg + MTX	CZP + MTX	0.77901	0.52542	1.05509
cDMARD + MTX	CZP + MTX	0.44774	0.21624	0.76988
ETN + MTX	CZP + MTX	1.24696	0.85772	1.88076
SARI 200mg + MTX	CZP + MTX	1.14238	0.66835	1.72532
CZP + MTX	CZP + MTX	1	1	1

Abbreviations: ABA abatacept; ADA adalimumab; BARI baricitinib; ETN etanercept; GOL golimumab; IFX infliximab; MTX methotrexate; PBO placebo; RTX rituximab; SARI sarilumab; SUBCUT subcutaneous.

[1]statistically significantly favouring BARI.

Odds ratios >1 are in favour of Treatment 1; and odds ratios <1 are in favour of Treatment 2.

10.1.3. ACR70 Response

Table S20 Baseline Risk-adjustment (Primary analysis): Relative treatment effect of pairwise comparisons expressed as Post. Median odds ratios (with 95% CrIs) – ACR70 response at week 24: MTX-IR (Simultaneous Fixed-effects model)

Treatment 1	Treatment 2	Median OR	95% CrI Lower	95% CrI Upper
PBO + MTX	PBO + MTX	1	1	1
**BARI 4mg + MTX[1]**	**PBO + MTX**	**5.37039**	**3.80805**	**7.34374**
ADA 40mg + MTX	PBO + MTX	3.91128	2.81506	5.17215
ABA 10mg + MTX	PBO + MTX	3.02855	2.00323	4.32636
ABA SUBCUT + MTX	PBO + MTX	3.5559	2.22037	5.5719
IFX 3mg + MTX	PBO + MTX	3.21707	2.23195	4.74214
RTX 1000mg + MTX	PBO + MTX	2.25232	1.31318	4.01331
GOL 50mg + MTX	PBO + MTX	3.66982	2.2815	6.16858
cDMARD + MTX	PBO + MTX	1.02396	0.39914	2.43945
ETN + MTX	PBO + MTX	4.30089	1.87691	9.12553
SARI 200mg + MTX	PBO + MTX	5.54668	1.41529	12.83707
CZP + MTX	PBO + MTX	3.93328	2.0289	8.53184
PBO + MTX	BARI 4mg + MTX	0.18621	0.13617	0.2626
BARI 4mg + MTX	BARI 4mg + MTX	1	1	1
ADA 40mg + MTX	BARI 4mg + MTX	0.72682	0.5391	0.97834
ABA 10mg + MTX	BARI 4mg + MTX	0.56331	0.34207	0.90605
ABA SUBCUT + MTX	BARI 4mg + MTX	0.66427	0.41508	1.05198
IFX 3mg + MTX	BARI 4mg + MTX	0.59979	0.37219	1.00288
RTX 1000mg + MTX	BARI 4mg + MTX	0.41954	0.22825	0.82304
GOL 50mg + MTX	BARI 4mg + MTX	0.6838	0.38948	1.28118
cDMARD + MTX	BARI 4mg + MTX	0.19105	0.07265	0.47647
ETN + MTX	BARI 4mg + MTX	0.80072	0.33846	1.78609
SARI 200mg + MTX	BARI 4mg + MTX	1.02695	0.26927	2.4107
CZP + MTX	BARI 4mg + MTX	0.73479	0.35676	1.71888
PBO + MTX	ADA 40mg + MTX	0.25567	0.19334	0.35523
**BARI 4mg + MTX[1]**	**ADA 40mg + MTX**	**1.37585**	**1.02214**	**1.85496**
ADA 40mg + MTX	ADA 40mg + MTX	1	1	1
ABA 10mg + MTX	ADA 40mg + MTX	0.77351	0.48305	1.22868
ABA SUBCUT + MTX	ADA 40mg + MTX	0.91339	0.63375	1.30918
IFX 3mg + MTX	ADA 40mg + MTX	0.8223	0.52642	1.36541
RTX 1000mg + MTX	ADA 40mg + MTX	0.57712	0.31817	1.12706
GOL 50mg + MTX	ADA 40mg + MTX	0.94056	0.5475	1.74803
cDMARD + MTX	ADA 40mg + MTX	0.26273	0.10082	0.64417
ETN + MTX	ADA 40mg + MTX	1.10245	0.47216	2.41817
SARI 200mg + MTX	ADA 40mg + MTX	1.41359	0.38518	3.25349
CZP + MTX	ADA 40mg + MTX	1.00706	0.498	2.35264
PBO + MTX	ABA 10mg + MTX	0.33019	0.23114	0.49919
**BARI 4mg + MTX[1]**	**ABA 10mg + MTX**	**1.77521**	**1.10369**	**2.92341**
ADA 40mg + MTX	ABA 10mg + MTX	1.2928	0.81388	2.07017
ABA 10mg + MTX	ABA 10mg + MTX	1	1	1
ABA SUBCUT + MTX	ABA 10mg + MTX	1.18236	0.66541	2.09828
IFX 3mg + MTX	ABA 10mg + MTX	1.06993	0.70215	1.66386
RTX 1000mg + MTX	ABA 10mg + MTX	0.74303	0.39433	1.5244
GOL 50mg + MTX	ABA 10mg + MTX	1.21583	0.67518	2.37555

Treatment 1	Treatment 2	Median OR	95% CrI Lower	95% CrI Upper
cDMARD + MTX	ABA 10mg + MTX	0.33966	0.12809	0.85696
ETN + MTX	ABA 10mg + MTX	1.4248	0.59649	3.2626
SARI 200mg + MTX	ABA 10mg + MTX	1.83622	0.4865	4.16198
CZP + MTX	ABA 10mg + MTX	1.30166	0.627	3.17597
PBO + MTX	ABA SUBCUT + MTX	0.28122	0.17947	0.45038
**BARI 4mg + MTX**	**ABA SUBCUT + MTX**	**1.50542**	**0.95059**	**2.40917**
ADA 40mg + MTX	ABA SUBCUT + MTX	1.09482	0.76384	1.5779
ABA 10mg + MTX	ABA SUBCUT + MTX	0.84577	0.47658	1.50284
ABA SUBCUT + MTX	ABA SUBCUT + MTX	1	1	1
IFX 3mg + MTX	ABA SUBCUT + MTX	0.9047	0.51104	1.65393
RTX 1000mg + MTX	ABA SUBCUT + MTX	0.63313	0.31648	1.34542
GOL 50mg + MTX	ABA SUBCUT + MTX	1.03171	0.5412	2.08277
cDMARD + MTX	ABA SUBCUT + MTX	0.28729	0.10472	0.75502
ETN + MTX	ABA SUBCUT + MTX	1.20471	0.48891	2.84945
SARI 200mg + MTX	ABA SUBCUT + MTX	1.53721	0.40246	3.85169
CZP + MTX	ABA SUBCUT + MTX	1.1063	0.50045	2.76322
PBO + MTX	IFX 3mg + MTX	0.31084	0.21088	0.44804
**BARI 4mg + MTX**	**IFX 3mg + MTX**	**1.66724**	**0.99713**	**2.68677**
ADA 40mg + MTX	IFX 3mg + MTX	1.2161	0.73238	1.89961
ABA 10mg + MTX	IFX 3mg + MTX	0.93464	0.60101	1.4242
ABA SUBCUT + MTX	IFX 3mg + MTX	1.10534	0.60462	1.95681
IFX 3mg + MTX	IFX 3mg + MTX	1	1	1
RTX 1000mg + MTX	IFX 3mg + MTX	0.6994	0.36382	1.3685
GOL 50mg + MTX	IFX 3mg + MTX	1.14041	0.62558	2.13688
cDMARD + MTX	IFX 3mg + MTX	0.31706	0.11576	0.79948
ETN + MTX	IFX 3mg + MTX	1.33166	0.5375	3.04323
SARI 200mg + MTX	IFX 3mg + MTX	1.70803	0.41044	4.32007
CZP + MTX	IFX 3mg + MTX	1.22334	0.57233	2.86981
PBO + MTX	RTX 1000mg + MTX	0.44399	0.24917	0.76151
**BARI 4mg + MTX[1]**	**RTX 1000mg + MTX**	**2.38356**	**1.21501**	**4.38113**
ADA 40mg + MTX	RTX 1000mg + MTX	1.73275	0.88726	3.14299
ABA 10mg + MTX	RTX 1000mg + MTX	1.34584	0.656	2.53597
ABA SUBCUT + MTX	RTX 1000mg + MTX	1.57945	0.74326	3.15977
IFX 3mg + MTX	RTX 1000mg + MTX	1.4298	0.73072	2.74858
RTX 1000mg + MTX	RTX 1000mg + MTX	1	1	1
GOL 50mg + MTX	RTX 1000mg + MTX	1.63224	0.79596	3.3749
cDMARD + MTX	RTX 1000mg + MTX	0.45448	0.15061	1.24607
ETN + MTX	RTX 1000mg + MTX	1.90866	0.68915	4.68683
SARI 200mg + MTX	RTX 1000mg + MTX	2.44256	0.52517	6.82748
CZP + MTX	RTX 1000mg + MTX	1.75717	0.7376	4.3872
PBO + MTX	GOL 50mg + MTX	0.27249	0.16211	0.43831
**BARI 4mg + MTX**	**GOL 50mg + MTX**	**1.46241**	**0.78053**	**2.56755**
ADA 40mg + MTX	GOL 50mg + MTX	1.06319	0.57207	1.82649
ABA 10mg + MTX	GOL 50mg + MTX	0.82249	0.42096	1.48108
ABA SUBCUT + MTX	GOL 50mg + MTX	0.96926	0.48013	1.84773
IFX 3mg + MTX	GOL 50mg + MTX	0.87688	0.46797	1.59851
RTX 1000mg + MTX	GOL 50mg + MTX	0.61266	0.29631	1.25634
GOL 50mg + MTX	GOL 50mg + MTX	1	1	1
cDMARD + MTX	GOL 50mg + MTX	0.27891	0.09419	0.74006
ETN + MTX	GOL 50mg + MTX	1.17213	0.4335	2.77635
SARI 200mg + MTX	GOL 50mg + MTX	1.48871	0.3356	4.07866
CZP + MTX	GOL 50mg + MTX	1.07761	0.46751	2.59276
PBO + MTX	cDMARD + MTX	0.9766	0.40993	2.50541
**BARI 4mg + MTX[1]**	**cDMARD + MTX**	**5.23431**	**2.09877**	**13.76403**
ADA 40mg + MTX	cDMARD + MTX	3.80614	1.55237	9.91831
ABA 10mg + MTX	cDMARD + MTX	2.94408	1.16691	7.80695

Treatment 1	Treatment 2	Median OR	95% CrI Lower	95% CrI Upper
ABA SUBCUT + MTX	cDMARD + MTX	3.4808	1.32446	9.54948
IFX 3mg + MTX	cDMARD + MTX	3.15397	1.25082	8.63824
RTX 1000mg + MTX	cDMARD + MTX	2.20033	0.80252	6.63947
GOL 50mg + MTX	cDMARD + MTX	3.58542	1.35125	10.61633
cDMARD + MTX	cDMARD + MTX	1	1	1
ETN + MTX	cDMARD + MTX	4.1695	2.42734	7.49767
SARI 200mg + MTX	cDMARD + MTX	5.34794	1.22074	16.34544
CZP + MTX	cDMARD + MTX	3.86171	1.31893	13.2175
PBO + MTX	ETN + MTX	0.23251	0.10958	0.53279
**BARI 4mg + MTX**	**ETN + MTX**	**1.24888**	**0.55988**	**2.95459**
ADA 40mg + MTX	ETN + MTX	0.90707	0.41354	2.11791
ABA 10mg + MTX	ETN + MTX	0.70185	0.3065	1.67647
ABA SUBCUT + MTX	ETN + MTX	0.83008	0.35094	2.04536
IFX 3mg + MTX	ETN + MTX	0.75094	0.3286	1.86047
RTX 1000mg + MTX	ETN + MTX	0.52393	0.21336	1.45106
GOL 50mg + MTX	ETN + MTX	0.85315	0.36019	2.30681
cDMARD + MTX	ETN + MTX	0.23984	0.13337	0.41197
ETN + MTX	ETN + MTX	1	1	1
SARI 200mg + MTX	ETN + MTX	1.28379	0.3081	3.48771
CZP + MTX	ETN + MTX	0.91828	0.34305	2.87751
PBO + MTX	SARI 200mg + MTX	0.18029	0.0779	0.70657
**BARI 4mg + MTX**	**SARI 200mg + MTX**	**0.97376**	**0.41482**	**3.7137**
ADA 40mg + MTX	SARI 200mg + MTX	0.70742	0.30736	2.59622
ABA 10mg + MTX	SARI 200mg + MTX	0.5446	0.24027	2.05549
ABA SUBCUT + MTX	SARI 200mg + MTX	0.65053	0.25963	2.48473
IFX 3mg + MTX	SARI 200mg + MTX	0.58547	0.23148	2.43639
RTX 1000mg + MTX	SARI 200mg + MTX	0.40941	0.14647	1.90413
GOL 50mg + MTX	SARI 200mg + MTX	0.67172	0.24518	2.97977
cDMARD + MTX	SARI 200mg + MTX	0.18699	0.06118	0.81918
ETN + MTX	SARI 200mg + MTX	0.77894	0.28672	3.24574
SARI 200mg + MTX	SARI 200mg + MTX	1	1	1
CZP + MTX	SARI 200mg + MTX	0.72157	0.24352	3.64066
PBO + MTX	CZP + MTX	0.25424	0.11721	0.49288
**BARI 4mg + MTX**	**CZP + MTX**	**1.36094**	**0.58177**	**2.80297**
ADA 40mg + MTX	CZP + MTX	0.99299	0.42505	2.00804
ABA 10mg + MTX	CZP + MTX	0.76825	0.31486	1.59491
ABA SUBCUT + MTX	CZP + MTX	0.90391	0.3619	1.9982
IFX 3mg + MTX	CZP + MTX	0.81743	0.34845	1.74724
RTX 1000mg + MTX	CZP + MTX	0.5691	0.22794	1.35574
GOL 50mg + MTX	CZP + MTX	0.92798	0.38569	2.13901
cDMARD + MTX	CZP + MTX	0.25895	0.07566	0.75819
ETN + MTX	CZP + MTX	1.089	0.34752	2.91499
SARI 200mg + MTX	CZP + MTX	1.38586	0.27468	4.1064
CZP + MTX	CZP + MTX	1	1	1

Abbreviations: ABA abatacept; ADA adalimumab; BARI baricitinib; ETN etanercept; GOL golimumab; IFX infliximab; MTX methotrexate; PBO placebo; RTX rituximab; SARI sarilumab; SUBCUT subcutaneous.

[1]statistically significantly favouring BARI.

Odds ratios >1 are in favour of Treatment 1; and odds ratios <1 are in favour of Treatment 2.

10.2. Inclusion of Trials with Prior bDMARD use of up to 20%

10.2.1. ACR20 Response

Table S21 Sensitivity analysis including trials with prior bDMARD use of up to 20%: Relative treatment effect of pairwise comparisons expressed as Post. Median odds ratios (with 95% CrIs) - ACR20 response at week 24: MTX-IR (Simultaneous Fixed-effects model)

Treatment 1	Treatment 2	Median OR	95% CrI Lower	95% CrI Upper
PBO + MTX	PBO + MTX	1	1	1
**BARI 4mg + MTX[1]**	**PBO + MTX**	**5.14605**	**3.98323**	**6.66296**
ADA 40mg + MTX	PBO + MTX	3.695	3.08144	4.42057
ABA 10mg + MTX	PBO + MTX	3.63351	2.80299	4.74228
ABA SUBCUT + MTX	PBO + MTX	3.83972	2.67139	5.47883
IFX 3mg + MTX	PBO + MTX	3.33178	2.64076	4.19838
RTX 1000mg + MTX	PBO + MTX	3.39909	2.42594	4.77651
GOL 50mg + MTX	PBO + MTX	3.95352	2.87804	5.47676
CZP + MTX	PBO + MTX	6.29142	4.9836	8.0343
TCZ 8mg + MTX	PBO + MTX	3.56961	2.83119	4.51707
cDMARD + MTX	PBO + MTX	3.0954	1.86082	5.19699
ETN + MTX	PBO + MTX	6.78865	4.22745	10.99119
SARI 200mg + MTX	PBO + MTX	4.13448	2.94363	5.81072
TOFA 5mg + MTX	PBO + MTX	3.81824	2.99152	4.85959
PBO + MTX	BARI 4mg + MTX	0.19432	0.15008	0.25105
BARI 4mg + MTX	BARI 4mg + MTX	1	1	1
ADA 40mg + MTX	BARI 4mg + MTX	0.71719	0.54509	0.94123
ABA 10mg + MTX	BARI 4mg + MTX	0.70529	0.48981	1.01946
ABA SUBCUT + MTX	BARI 4mg + MTX	0.74506	0.49049	1.12596
IFX 3mg + MTX	BARI 4mg + MTX	0.64642	0.45798	0.91216
RTX 1000mg + MTX	BARI 4mg + MTX	0.66035	0.43425	1.00754
GOL 50mg + MTX	BARI 4mg + MTX	0.76846	0.51257	1.16187
CZP + MTX	BARI 4mg + MTX	1.22187	0.86067	1.74395
TCZ 8mg + MTX	BARI 4mg + MTX	0.69383	0.49226	0.98196
cDMARD + MTX	BARI 4mg + MTX	0.60181	0.33928	1.06752
ETN + MTX	BARI 4mg + MTX	1.32074	0.77283	2.27778
SARI 200mg + MTX	BARI 4mg + MTX	0.80136	0.52597	1.22853
TOFA 5mg + MTX	BARI 4mg + MTX	0.74096	0.53312	1.02875
PBO + MTX	ADA 40mg + MTX	0.27064	0.22622	0.32452
**BARI 4mg + MTX[1]**	**ADA 40mg + MTX**	**1.39433**	**1.06244**	**1.83455**
ADA 40mg + MTX	ADA 40mg + MTX	1	1	1
ABA 10mg + MTX	ADA 40mg + MTX	0.98354	0.72173	1.34947
ABA SUBCUT + MTX	ADA 40mg + MTX	1.03861	0.75418	1.42491
IFX 3mg + MTX	ADA 40mg + MTX	0.90044	0.67326	1.20553
RTX 1000mg + MTX	ADA 40mg + MTX	0.92005	0.6318	1.34921
GOL 50mg + MTX	ADA 40mg + MTX	1.07145	0.74825	1.54809
CZP + MTX	ADA 40mg + MTX	1.70305	1.26974	2.3152
TCZ 8mg + MTX	ADA 40mg + MTX	0.96586	0.72291	1.29729
cDMARD + MTX	ADA 40mg + MTX	0.83693	0.4948	1.44061
ETN + MTX	ADA 40mg + MTX	1.83699	1.12805	3.04914

Treatment 1	Treatment 2	Median OR	95% CrI Lower	95% CrI Upper
SARI 200mg + MTX	ADA 40mg + MTX	1.11736	0.76509	1.64239
TOFA 5mg + MTX	ADA 40mg + MTX	1.03326	0.83087	1.28233
PBO + MTX	ABA 10mg + MTX	0.27522	0.21087	0.35676
**BARI 4mg + MTX**	**ABA 10mg + MTX**	**1.41786**	**0.98092**	**2.04161**
ADA 40mg + MTX	ABA 10mg + MTX	1.01674	0.74103	1.38557
ABA 10mg + MTX	ABA 10mg + MTX	1	1	1
ABA SUBCUT + MTX	ABA 10mg + MTX	1.05697	0.67628	1.63348
IFX 3mg + MTX	ABA 10mg + MTX	0.91543	0.66852	1.25547
RTX 1000mg + MTX	ABA 10mg + MTX	0.93437	0.60982	1.43697
GOL 50mg + MTX	ABA 10mg + MTX	1.08788	0.72105	1.65015
CZP + MTX	ABA 10mg + MTX	1.73091	1.20653	2.48518
TCZ 8mg + MTX	ABA 10mg + MTX	0.982	0.69056	1.39299
cDMARD + MTX	ABA 10mg + MTX	0.84836	0.48282	1.51665
ETN + MTX	ABA 10mg + MTX	1.86915	1.09767	3.23373
SARI 200mg + MTX	ABA 10mg + MTX	1.13401	0.74169	1.74126
TOFA 5mg + MTX	ABA 10mg + MTX	1.04936	0.73669	1.48654
PBO + MTX	ABA SUBCUT + MTX	0.26044	0.18252	0.37434
**BARI 4mg + MTX**	**ABA SUBCUT + MTX**	**1.34217**	**0.88813**	**2.03879**
ADA 40mg + MTX	ABA SUBCUT + MTX	0.96282	0.7018	1.32594
ABA 10mg + MTX	ABA SUBCUT + MTX	0.9461	0.61219	1.47867
ABA SUBCUT + MTX	ABA SUBCUT + MTX	1	1	1
IFX 3mg + MTX	ABA SUBCUT + MTX	0.86737	0.56688	1.32944
RTX 1000mg + MTX	ABA SUBCUT + MTX	0.88698	0.54589	1.44116
GOL 50mg + MTX	ABA SUBCUT + MTX	1.03093	0.64212	1.667
CZP + MTX	ABA SUBCUT + MTX	1.63984	1.07016	2.54286
TCZ 8mg + MTX	ABA SUBCUT + MTX	0.93006	0.60996	1.42913
cDMARD + MTX	ABA SUBCUT + MTX	0.80501	0.44036	1.50306
ETN + MTX	ABA SUBCUT + MTX	1.77082	0.99976	3.20037
SARI 200mg + MTX	ABA SUBCUT + MTX	1.07609	0.65989	1.76545
TOFA 5mg + MTX	ABA SUBCUT + MTX	0.9937	0.67935	1.46316
PBO + MTX	IFX 3mg + MTX	0.30014	0.23819	0.37868
**BARI 4mg + MTX[1]**	**IFX 3mg + MTX**	**1.54697**	**1.0963**	**2.18348**
ADA 40mg + MTX	IFX 3mg + MTX	1.11057	0.82951	1.48531
ABA 10mg + MTX	IFX 3mg + MTX	1.09238	0.79651	1.49585
ABA SUBCUT + MTX	IFX 3mg + MTX	1.15291	0.7522	1.76405
IFX 3mg + MTX	IFX 3mg + MTX	1	1	1
RTX 1000mg + MTX	IFX 3mg + MTX	1.02033	0.68095	1.53516
GOL 50mg + MTX	IFX 3mg + MTX	1.18904	0.80453	1.76781
CZP + MTX	IFX 3mg + MTX	1.88948	1.36374	2.63193
TCZ 8mg + MTX	IFX 3mg + MTX	1.07217	0.77513	1.48737
cDMARD + MTX	IFX 3mg + MTX	0.92836	0.53516	1.63085
ETN + MTX	IFX 3mg + MTX	2.03998	1.2085	3.45108
SARI 200mg + MTX	IFX 3mg + MTX	1.24125	0.82493	1.87317
TOFA 5mg + MTX	IFX 3mg + MTX	1.14728	0.82039	1.59071
PBO + MTX	RTX 1000mg + MTX	0.2942	0.20936	0.41221
**BARI 4mg + MTX**	**RTX 1000mg + MTX**	**1.51435**	**0.99252**	**2.30284**
ADA 40mg + MTX	RTX 1000mg + MTX	1.0869	0.74118	1.58278
ABA 10mg + MTX	RTX 1000mg + MTX	1.07024	0.69591	1.63982
ABA SUBCUT + MTX	RTX 1000mg + MTX	1.12742	0.69389	1.83189
IFX 3mg + MTX	RTX 1000mg + MTX	0.98007	0.6514	1.46854
RTX 1000mg + MTX	RTX 1000mg + MTX	1	1	1
GOL 50mg + MTX	RTX 1000mg + MTX	1.16293	0.73728	1.84862
CZP + MTX	RTX 1000mg + MTX	1.84968	1.23537	2.77505
TCZ 8mg + MTX	RTX 1000mg + MTX	1.0497	0.69749	1.5787
cDMARD + MTX	RTX 1000mg + MTX	0.9111	0.49808	1.66289
ETN + MTX	RTX 1000mg + MTX	1.99957	1.12836	3.54835

Treatment 1	Treatment 2	Median OR	95% CrI Lower	95% CrI Upper
SARI 200mg + MTX	RTX 1000mg + MTX	1.21641	0.75306	1.95597
TOFA 5mg + MTX	RTX 1000mg + MTX	1.12244	0.74158	1.68833
PBO + MTX	GOL 50mg + MTX	0.25294	0.18259	0.34746
**BARI 4mg + MTX**	**GOL 50mg + MTX**	**1.3013**	**0.86068**	**1.95095**
ADA 40mg + MTX	GOL 50mg + MTX	0.93332	0.64596	1.33645
ABA 10mg + MTX	GOL 50mg + MTX	0.91922	0.60601	1.38687
ABA SUBCUT + MTX	GOL 50mg + MTX	0.96999	0.59988	1.55735
IFX 3mg + MTX	GOL 50mg + MTX	0.84102	0.56567	1.24296
RTX 1000mg + MTX	GOL 50mg + MTX	0.8599	0.54094	1.35634
GOL 50mg + MTX	GOL 50mg + MTX	1	1	1
CZP + MTX	GOL 50mg + MTX	1.58914	1.07779	2.34946
TCZ 8mg + MTX	GOL 50mg + MTX	0.90295	0.60805	1.32911
cDMARD + MTX	GOL 50mg + MTX	0.7819	0.43197	1.41916
ETN + MTX	GOL 50mg + MTX	1.7199	0.97529	3.00735
SARI 200mg + MTX	GOL 50mg + MTX	1.04373	0.65167	1.66993
TOFA 5mg + MTX	GOL 50mg + MTX	0.96512	0.64669	1.42595
PBO + MTX	CZP + MTX	0.15895	0.12447	0.20066
**BARI 4mg + MTX**	**CZP + MTX**	**0.81842**	**0.57341**	**1.16189**
ADA 40mg + MTX	CZP + MTX	0.58718	0.43193	0.78756
ABA 10mg + MTX	CZP + MTX	0.57773	0.40239	0.82882
ABA SUBCUT + MTX	CZP + MTX	0.60982	0.39326	0.93444
IFX 3mg + MTX	CZP + MTX	0.52925	0.37995	0.73328
RTX 1000mg + MTX	CZP + MTX	0.54063	0.36035	0.80947
GOL 50mg + MTX	CZP + MTX	0.62927	0.42563	0.92783
CZP + MTX	CZP + MTX	1	1	1
TCZ 8mg + MTX	CZP + MTX	0.56758	0.40657	0.79157
cDMARD + MTX	CZP + MTX	0.49204	0.28078	0.84771
ETN + MTX	CZP + MTX	1.08097	0.63714	1.80931
SARI 200mg + MTX	CZP + MTX	0.65672	0.43034	0.99834
TOFA 5mg + MTX	CZP + MTX	0.6067	0.43154	0.84505
PBO + MTX	TCZ 8mg + MTX	0.28014	0.22138	0.35321
**BARI 4mg + MTX[1]**	**TCZ 8mg + MTX**	**1.44127**	**1.01838**	**2.03144**
ADA 40mg + MTX	TCZ 8mg + MTX	1.03534	0.77084	1.3833
ABA 10mg + MTX	TCZ 8mg + MTX	1.01833	0.71788	1.4481
ABA SUBCUT + MTX	TCZ 8mg + MTX	1.0752	0.69973	1.63944
IFX 3mg + MTX	TCZ 8mg + MTX	0.93269	0.67233	1.29011
RTX 1000mg + MTX	TCZ 8mg + MTX	0.95265	0.63343	1.43371
GOL 50mg + MTX	TCZ 8mg + MTX	1.10749	0.75238	1.64461
CZP + MTX	TCZ 8mg + MTX	1.76188	1.26331	2.4596
TCZ 8mg + MTX	TCZ 8mg + MTX	1	1	1
cDMARD + MTX	TCZ 8mg + MTX	0.8648	0.49644	1.51521
ETN + MTX	TCZ 8mg + MTX	1.90306	1.13013	3.22819
SARI 200mg + MTX	TCZ 8mg + MTX	1.15629	0.76606	1.74478
TOFA 5mg + MTX	TCZ 8mg + MTX	1.06889	0.76493	1.48863
PBO + MTX	cDMARD + MTX	0.32306	0.19242	0.5374
**BARI 4mg + MTX**	**cDMARD + MTX**	**1.66165**	**0.93675**	**2.9474**
ADA 40mg + MTX	cDMARD + MTX	1.19485	0.69415	2.02102
ABA 10mg + MTX	cDMARD + MTX	1.17874	0.65935	2.07117
ABA SUBCUT + MTX	cDMARD + MTX	1.24222	0.66531	2.27088
IFX 3mg + MTX	cDMARD + MTX	1.07717	0.61318	1.86859
RTX 1000mg + MTX	cDMARD + MTX	1.09758	0.60136	2.00769
GOL 50mg + MTX	cDMARD + MTX	1.27894	0.70464	2.31498
CZP + MTX	cDMARD + MTX	2.03235	1.17965	3.56145
TCZ 8mg + MTX	cDMARD + MTX	1.15634	0.65997	2.01435
cDMARD + MTX	cDMARD + MTX	1	1	1
ETN + MTX	cDMARD + MTX	2.19566	1.61672	3.00106

Treatment 1	Treatment 2	Median OR	95% CrI Lower	95% CrI Upper
SARI 200mg + MTX	cDMARD + MTX	1.33686	0.7167	2.44778
TOFA 5mg + MTX	cDMARD + MTX	1.2353	0.70797	2.13365
PBO + MTX	ETN + MTX	0.1473	0.09098	0.23655
**BARI 4mg + MTX**	**ETN + MTX**	**0.75715**	**0.43902**	**1.29395**
ADA 40mg + MTX	ETN + MTX	0.54437	0.32796	0.88648
ABA 10mg + MTX	ETN + MTX	0.535	0.30924	0.91102
ABA SUBCUT + MTX	ETN + MTX	0.56471	0.31246	1.00024
IFX 3mg + MTX	ETN + MTX	0.4902	0.28976	0.82747
RTX 1000mg + MTX	ETN + MTX	0.50011	0.28182	0.88625
GOL 50mg + MTX	ETN + MTX	0.58143	0.33252	1.02533
CZP + MTX	ETN + MTX	0.9251	0.5527	1.56952
TCZ 8mg + MTX	ETN + MTX	0.52547	0.30977	0.88486
cDMARD + MTX	ETN + MTX	0.45544	0.33322	0.61854
ETN + MTX	ETN + MTX	1	1	1
SARI 200mg + MTX	ETN + MTX	0.60872	0.33836	1.07547
TOFA 5mg + MTX	ETN + MTX	0.56259	0.33335	0.93953
PBO + MTX	SARI 200mg + MTX	0.24187	0.1721	0.33972
**BARI 4mg + MTX**	**SARI 200mg + MTX**	**1.24788**	**0.81398**	**1.90127**
ADA 40mg + MTX	SARI 200mg + MTX	0.89497	0.60887	1.30703
ABA 10mg + MTX	SARI 200mg + MTX	0.88183	0.5743	1.34827
ABA SUBCUT + MTX	SARI 200mg + MTX	0.92929	0.56643	1.5154
IFX 3mg + MTX	SARI 200mg + MTX	0.80564	0.53386	1.21223
RTX 1000mg + MTX	SARI 200mg + MTX	0.82209	0.51126	1.32791
GOL 50mg + MTX	SARI 200mg + MTX	0.9581	0.59883	1.53453
CZP + MTX	SARI 200mg + MTX	1.52272	1.00167	2.32373
TCZ 8mg + MTX	SARI 200mg + MTX	0.86483	0.57314	1.30537
cDMARD + MTX	SARI 200mg + MTX	0.74802	0.40853	1.39529
ETN + MTX	SARI 200mg + MTX	1.6428	0.92982	2.95541
SARI 200mg + MTX	SARI 200mg + MTX	1	1	1
TOFA 5mg + MTX	SARI 200mg + MTX	0.92446	0.6094	1.39578
PBO + MTX	TOFA 5mg + MTX	0.2619	0.20578	0.33428
**BARI 4mg + MTX**	**TOFA 5mg + MTX**	**1.34959**	**0.97206**	**1.87576**
ADA 40mg + MTX	TOFA 5mg + MTX	0.96781	0.77983	1.20356
ABA 10mg + MTX	TOFA 5mg + MTX	0.95297	0.6727	1.35742
ABA SUBCUT + MTX	TOFA 5mg + MTX	1.00634	0.68345	1.47199
IFX 3mg + MTX	TOFA 5mg + MTX	0.87163	0.62865	1.21893
RTX 1000mg + MTX	TOFA 5mg + MTX	0.89092	0.5923	1.34846
GOL 50mg + MTX	TOFA 5mg + MTX	1.03614	0.70129	1.54634
CZP + MTX	TOFA 5mg + MTX	1.64825	1.18336	2.3173
TCZ 8mg + MTX	TOFA 5mg + MTX	0.93555	0.67176	1.30731
cDMARD + MTX	TOFA 5mg + MTX	0.80952	0.46868	1.41249
ETN + MTX	TOFA 5mg + MTX	1.77749	1.06437	2.99987
SARI 200mg + MTX	TOFA 5mg + MTX	1.08171	0.71645	1.64096
TOFA 5mg + MTX	TOFA 5mg + MTX	1	1	1

Abbreviations: ABA abatacept; ADA adalimumab; BARI baricitinib; ETN etanercept; GOL golimumab; IFX infliximab; MTX methotrexate; PBO placebo; RTX rituximab; SARI sarilumab; SUBCUT subcutaneous; TCZ tocilizumab; TOFA tofacitinib.

[1]statistically significantly favouring BARI.

Odds ratios >1 are in favour of Treatment 1; and odds ratios <1 are in favour of Treatment 2.

10.2.2. ACR50 Response

Table S22 Sensitivity analysis including trials with prior bDMARD use of up to 20%: Relative treatment effect of pairwise comparisons expressed as Post. Median odds ratios (with 95% CrIs) – ACR50 response at week 24: MTX-IR (Simultaneous Fixed-effects model)

Treatment 1	Treatment 2	Median OR	95% CrI Lower	95% CrI Upper
PBO + MTX	PBO + MTX	1	1	1
**BARI 4mg + MTX[1]**	**PBO + MTX**	**4.92284**	**3.80091**	**6.38747**
ADA 40mg + MTX	PBO + MTX	4.4108	3.59902	5.41705
ABA 10mg + MTX	PBO + MTX	3.6226	2.69699	4.89807
ABA SUBCUT + MTX	PBO + MTX	4.44791	3.09176	6.40423
IFX 3mg + MTX	PBO + MTX	3.57207	2.65987	4.80206
RTX 1000mg + MTX	PBO + MTX	3.37328	2.25	5.1324
GOL 50mg + MTX	PBO + MTX	3.55103	2.45449	5.18692
CZP + MTX	PBO + MTX	6.07995	4.6487	8.09874
TCZ 8mg + MTX	PBO + MTX	4.81264	3.60205	6.49512
cDMARD + MTX	PBO + MTX	2.21111	1.23228	4.04475
ETN + MTX	PBO + MTX	6.90245	4.07536	12.02721
SARI 200mg + MTX	PBO + MTX	4.48144	3.17723	6.37388
TOFA 5mg + MTX	PBO + MTX	5.16828	3.96623	6.78726
PBO + MTX	BARI 4mg + MTX	0.20313	0.15656	0.26309
BARI 4mg + MTX	BARI 4mg + MTX	1	1	1
ADA 40mg + MTX	BARI 4mg + MTX	0.89609	0.69411	1.15565
ABA 10mg + MTX	BARI 4mg + MTX	0.73589	0.49877	1.0928
ABA SUBCUT + MTX	BARI 4mg + MTX	0.9048	0.60339	1.34518
IFX 3mg + MTX	BARI 4mg + MTX	0.72532	0.49013	1.0727
RTX 1000mg + MTX	BARI 4mg + MTX	0.68561	0.42426	1.12108
GOL 50mg + MTX	BARI 4mg + MTX	0.7214	0.46295	1.12754
CZP + MTX	BARI 4mg + MTX	1.23667	0.85545	1.80824
TCZ 8mg + MTX	BARI 4mg + MTX	0.97867	0.6663	1.45581
cDMARD + MTX	BARI 4mg + MTX	0.44888	0.23897	0.85552
ETN + MTX	BARI 4mg + MTX	1.40231	0.7886	2.5587
SARI 200mg + MTX	BARI 4mg + MTX	0.9094	0.59491	1.40896
TOFA 5mg + MTX	BARI 4mg + MTX	1.05117	0.76128	1.45157
PBO + MTX	ADA 40mg + MTX	0.22672	0.1846	0.27785
**BARI 4mg + MTX**	**ADA 40mg + MTX**	**1.11596**	**0.86531**	**1.4407**
ADA 40mg + MTX	ADA 40mg + MTX	1	1	1
ABA 10mg + MTX	ADA 40mg + MTX	0.82114	0.5761	1.17107
ABA SUBCUT + MTX	ADA 40mg + MTX	1.0089	0.73968	1.36986
IFX 3mg + MTX	ADA 40mg + MTX	0.81	0.56947	1.15375
RTX 1000mg + MTX	ADA 40mg + MTX	0.76507	0.48911	1.20547
GOL 50mg + MTX	ADA 40mg + MTX	0.80348	0.53252	1.22401
CZP + MTX	ADA 40mg + MTX	1.37873	0.99631	1.93743
TCZ 8mg + MTX	ADA 40mg + MTX	1.09144	0.77095	1.56067
cDMARD + MTX	ADA 40mg + MTX	0.50082	0.27474	0.92864
ETN + MTX	ADA 40mg + MTX	1.56353	0.90734	2.77454
SARI 200mg + MTX	ADA 40mg + MTX	1.01735	0.6841	1.52205
TOFA 5mg + MTX	ADA 40mg + MTX	1.17222	0.94438	1.45672
PBO + MTX	ABA 10mg + MTX	0.27605	0.20416	0.37078
**BARI 4mg + MTX**	**ABA 10mg + MTX**	**1.3589**	**0.91508**	**2.00491**

Treatment 1	Treatment 2	Median OR	95% CrI Lower	95% CrI Upper
ADA 40mg + MTX	ABA 10mg + MTX	1.21782	0.85392	1.73581
ABA 10mg + MTX	ABA 10mg + MTX	1	1	1
ABA SUBCUT + MTX	ABA 10mg + MTX	1.22674	0.76788	1.95755
IFX 3mg + MTX	ABA 10mg + MTX	0.98526	0.69937	1.39538
RTX 1000mg + MTX	ABA 10mg + MTX	0.93132	0.56196	1.54313
GOL 50mg + MTX	ABA 10mg + MTX	0.97873	0.61414	1.58276
CZP + MTX	ABA 10mg + MTX	1.67751	1.12524	2.53607
TCZ 8mg + MTX	ABA 10mg + MTX	1.32944	0.88014	2.02991
cDMARD + MTX	ABA 10mg + MTX	0.60913	0.32071	1.1868
ETN + MTX	ABA 10mg + MTX	1.90447	1.0505	3.56155
SARI 200mg + MTX	ABA 10mg + MTX	1.23737	0.78521	1.95407
TOFA 5mg + MTX	ABA 10mg + MTX	1.4265	0.96279	2.12469
PBO + MTX	ABA SUBCUT + MTX	0.22482	0.15615	0.32344
**BARI 4mg + MTX**	**ABA SUBCUT + MTX**	**1.10522**	**0.74339**	**1.65732**
ADA 40mg + MTX	ABA SUBCUT + MTX	0.99118	0.73	1.35194
ABA 10mg + MTX	ABA SUBCUT + MTX	0.81517	0.51084	1.30228
ABA SUBCUT + MTX	ABA SUBCUT + MTX	1	1	1
IFX 3mg + MTX	ABA SUBCUT + MTX	0.80382	0.50421	1.28067
RTX 1000mg + MTX	ABA SUBCUT + MTX	0.75773	0.44445	1.312
GOL 50mg + MTX	ABA SUBCUT + MTX	0.79768	0.48006	1.34176
CZP + MTX	ABA SUBCUT + MTX	1.36911	0.87903	2.15469
TCZ 8mg + MTX	ABA SUBCUT + MTX	1.08445	0.68586	1.72942
cDMARD + MTX	ABA SUBCUT + MTX	0.49689	0.25508	0.98441
ETN + MTX	ABA SUBCUT + MTX	1.55083	0.83994	2.96651
SARI 200mg + MTX	ABA SUBCUT + MTX	1.00843	0.61287	1.67343
TOFA 5mg + MTX	ABA SUBCUT + MTX	1.16308	0.80083	1.69437
PBO + MTX	IFX 3mg + MTX	0.27995	0.20824	0.37596
**BARI 4mg + MTX**	**IFX 3mg + MTX**	**1.3787**	**0.93223**	**2.04029**
ADA 40mg + MTX	IFX 3mg + MTX	1.23457	0.86674	1.75602
ABA 10mg + MTX	IFX 3mg + MTX	1.01496	0.71665	1.42987
ABA SUBCUT + MTX	IFX 3mg + MTX	1.24405	0.78084	1.98329
IFX 3mg + MTX	IFX 3mg + MTX	1	1	1
RTX 1000mg + MTX	IFX 3mg + MTX	0.94452	0.57359	1.56298
GOL 50mg + MTX	IFX 3mg + MTX	0.99468	0.62054	1.59289
CZP + MTX	IFX 3mg + MTX	1.70285	1.15004	2.55664
TCZ 8mg + MTX	IFX 3mg + MTX	1.34883	0.89188	2.04576
cDMARD + MTX	IFX 3mg + MTX	0.62032	0.32617	1.19851
ETN + MTX	IFX 3mg + MTX	1.93269	1.06856	3.58053
SARI 200mg + MTX	IFX 3mg + MTX	1.25532	0.79664	1.98886
TOFA 5mg + MTX	IFX 3mg + MTX	1.44794	0.97942	2.14531
PBO + MTX	RTX 1000mg + MTX	0.29645	0.19484	0.44444
**BARI 4mg + MTX**	**RTX 1000mg + MTX**	**1.45854**	**0.89199**	**2.35705**
ADA 40mg + MTX	RTX 1000mg + MTX	1.30707	0.82955	2.04452
ABA 10mg + MTX	RTX 1000mg + MTX	1.07374	0.64804	1.77947
ABA SUBCUT + MTX	RTX 1000mg + MTX	1.31974	0.7622	2.24997
IFX 3mg + MTX	RTX 1000mg + MTX	1.05874	0.6398	1.74342
RTX 1000mg + MTX	RTX 1000mg + MTX	1	1	1
GOL 50mg + MTX	RTX 1000mg + MTX	1.05323	0.60636	1.81817
CZP + MTX	RTX 1000mg + MTX	1.80122	1.11696	2.91667
TCZ 8mg + MTX	RTX 1000mg + MTX	1.42642	0.86808	2.36546
cDMARD + MTX	RTX 1000mg + MTX	0.65717	0.32754	1.32075
ETN + MTX	RTX 1000mg + MTX	2.04799	1.06692	3.97474
SARI 200mg + MTX	RTX 1000mg + MTX	1.32663	0.77032	2.28573
TOFA 5mg + MTX	RTX 1000mg + MTX	1.53329	0.94717	2.47234
PBO + MTX	GOL 50mg + MTX	0.28161	0.19279	0.40742
**BARI 4mg + MTX**	**GOL 50mg + MTX**	**1.38619**	**0.88688**	**2.16005**

Treatment 1	Treatment 2	Median OR	95% CrI Lower	95% CrI Upper
ADA 40mg + MTX	GOL 50mg + MTX	1.24458	0.81699	1.87786
ABA 10mg + MTX	GOL 50mg + MTX	1.02173	0.63181	1.6283
ABA SUBCUT + MTX	GOL 50mg + MTX	1.25363	0.74529	2.08308
IFX 3mg + MTX	GOL 50mg + MTX	1.00534	0.62779	1.61149
RTX 1000mg + MTX	GOL 50mg + MTX	0.94946	0.55	1.64918
GOL 50mg + MTX	GOL 50mg + MTX	1	1	1
CZP + MTX	GOL 50mg + MTX	1.71448	1.09551	2.70376
TCZ 8mg + MTX	GOL 50mg + MTX	1.35578	0.8498	2.16762
cDMARD + MTX	GOL 50mg + MTX	0.62187	0.3149	1.23578
ETN + MTX	GOL 50mg + MTX	1.94412	1.03205	3.70368
SARI 200mg + MTX	GOL 50mg + MTX	1.26104	0.75305	2.11303
TOFA 5mg + MTX	GOL 50mg + MTX	1.45644	0.92728	2.27755
PBO + MTX	CZP + MTX	0.16447	0.12348	0.21511
**BARI 4mg + MTX**	**CZP + MTX**	**0.80862**	**0.55302**	**1.16898**
ADA 40mg + MTX	CZP + MTX	0.72531	0.51615	1.0037
ABA 10mg + MTX	CZP + MTX	0.59612	0.39431	0.8887
ABA SUBCUT + MTX	CZP + MTX	0.7304	0.4641	1.13762
IFX 3mg + MTX	CZP + MTX	0.58725	0.39114	0.86954
RTX 1000mg + MTX	CZP + MTX	0.55518	0.34286	0.89528
GOL 50mg + MTX	CZP + MTX	0.58327	0.36985	0.91282
CZP + MTX	CZP + MTX	1	1	1
TCZ 8mg + MTX	CZP + MTX	0.79151	0.53255	1.17142
cDMARD + MTX	CZP + MTX	0.3646	0.19294	0.68112
ETN + MTX	CZP + MTX	1.13694	0.63847	2.02104
SARI 200mg + MTX	CZP + MTX	0.73631	0.46765	1.15689
TOFA 5mg + MTX	CZP + MTX	0.85048	0.58286	1.23035
PBO + MTX	TCZ 8mg + MTX	0.20779	0.15396	0.27762
**BARI 4mg + MTX**	**TCZ 8mg + MTX**	**1.02179**	**0.6869**	**1.50083**
ADA 40mg + MTX	TCZ 8mg + MTX	0.91622	0.64075	1.2971
ABA 10mg + MTX	TCZ 8mg + MTX	0.7522	0.49263	1.13618
ABA SUBCUT + MTX	TCZ 8mg + MTX	0.92213	0.57823	1.45803
IFX 3mg + MTX	TCZ 8mg + MTX	0.74138	0.48881	1.12123
RTX 1000mg + MTX	TCZ 8mg + MTX	0.70105	0.42275	1.15197
GOL 50mg + MTX	TCZ 8mg + MTX	0.73758	0.46133	1.17675
CZP + MTX	TCZ 8mg + MTX	1.26341	0.85366	1.87775
TCZ 8mg + MTX	TCZ 8mg + MTX	1	1	1
cDMARD + MTX	TCZ 8mg + MTX	0.4592	0.24022	0.88222
ETN + MTX	TCZ 8mg + MTX	1.4335	0.78914	2.63642
SARI 200mg + MTX	TCZ 8mg + MTX	0.93048	0.58856	1.47132
TOFA 5mg + MTX	TCZ 8mg + MTX	1.0731	0.72387	1.57837
PBO + MTX	cDMARD + MTX	0.45226	0.24723	0.81151
**BARI 4mg + MTX[1]**	**cDMARD + MTX**	**2.22775**	**1.16888**	**4.18461**
ADA 40mg + MTX	cDMARD + MTX	1.99672	1.07684	3.63984
ABA 10mg + MTX	cDMARD + MTX	1.64168	0.8426	3.11804
ABA SUBCUT + MTX	cDMARD + MTX	2.01251	1.01584	3.92029
IFX 3mg + MTX	cDMARD + MTX	1.61206	0.83437	3.06592
RTX 1000mg + MTX	cDMARD + MTX	1.52167	0.75715	3.05303
GOL 50mg + MTX	cDMARD + MTX	1.60804	0.80921	3.17566
CZP + MTX	cDMARD + MTX	2.74276	1.46816	5.18299
TCZ 8mg + MTX	cDMARD + MTX	2.17768	1.1335	4.16291
cDMARD + MTX	cDMARD + MTX	1	1	1
ETN + MTX	cDMARD + MTX	3.12087	2.2574	4.32991
SARI 200mg + MTX	cDMARD + MTX	2.03566	1.01224	3.92905
TOFA 5mg + MTX	cDMARD + MTX	2.34048	1.23516	4.35614
PBO + MTX	ETN + MTX	0.14488	0.08314	0.24538
**BARI 4mg + MTX**	**ETN + MTX**	**0.71311**	**0.39082**	**1.26806**

Treatment 1	Treatment 2	Median OR	95% CrI Lower	95% CrI Upper
ADA 40mg + MTX	ETN + MTX	0.63958	0.36042	1.10213
ABA 10mg + MTX	ETN + MTX	0.52508	0.28078	0.95192
ABA SUBCUT + MTX	ETN + MTX	0.64481	0.3371	1.19057
IFX 3mg + MTX	ETN + MTX	0.51741	0.27929	0.93584
RTX 1000mg + MTX	ETN + MTX	0.48828	0.25159	0.93727
GOL 50mg + MTX	ETN + MTX	0.51437	0.27	0.96894
CZP + MTX	ETN + MTX	0.87955	0.49479	1.56624
TCZ 8mg + MTX	ETN + MTX	0.69759	0.3793	1.2672
cDMARD + MTX	ETN + MTX	0.32042	0.23095	0.44299
ETN + MTX	ETN + MTX	1	1	1
SARI 200mg + MTX	ETN + MTX	0.65088	0.33482	1.20984
TOFA 5mg + MTX	ETN + MTX	0.74823	0.41359	1.32875
PBO + MTX	SARI 200mg + MTX	0.22314	0.15689	0.31474
**BARI 4mg + MTX**	**SARI 200mg + MTX**	**1.09963**	**0.70974**	**1.68094**
ADA 40mg + MTX	SARI 200mg + MTX	0.98295	0.65701	1.46178
ABA 10mg + MTX	SARI 200mg + MTX	0.80817	0.51175	1.27355
ABA SUBCUT + MTX	SARI 200mg + MTX	0.99164	0.59758	1.63168
IFX 3mg + MTX	SARI 200mg + MTX	0.79661	0.5028	1.25527
RTX 1000mg + MTX	SARI 200mg + MTX	0.75379	0.4375	1.29817
GOL 50mg + MTX	SARI 200mg + MTX	0.793	0.47325	1.32793
CZP + MTX	SARI 200mg + MTX	1.35812	0.86438	2.13836
TCZ 8mg + MTX	SARI 200mg + MTX	1.07471	0.67966	1.69905
cDMARD + MTX	SARI 200mg + MTX	0.49124	0.25451	0.9879
ETN + MTX	SARI 200mg + MTX	1.53638	0.82656	2.9867
SARI 200mg + MTX	SARI 200mg + MTX	1	1	1
TOFA 5mg + MTX	SARI 200mg + MTX	1.15364	0.74323	1.78556
PBO + MTX	TOFA 5mg + MTX	0.19349	0.14733	0.25213
**BARI 4mg + MTX**	**TOFA 5mg + MTX**	**0.95132**	**0.68891**	**1.31358**
ADA 40mg + MTX	TOFA 5mg + MTX	0.85308	0.68647	1.05889
ABA 10mg + MTX	TOFA 5mg + MTX	0.70102	0.47066	1.03865
ABA SUBCUT + MTX	TOFA 5mg + MTX	0.85979	0.59019	1.24871
IFX 3mg + MTX	TOFA 5mg + MTX	0.69064	0.46613	1.02101
RTX 1000mg + MTX	TOFA 5mg + MTX	0.65219	0.40447	1.05578
GOL 50mg + MTX	TOFA 5mg + MTX	0.6866	0.43907	1.07842
CZP + MTX	TOFA 5mg + MTX	1.17581	0.81278	1.71568
TCZ 8mg + MTX	TOFA 5mg + MTX	0.93188	0.63357	1.38146
cDMARD + MTX	TOFA 5mg + MTX	0.42726	0.22956	0.80961
ETN + MTX	TOFA 5mg + MTX	1.3365	0.75259	2.41785
SARI 200mg + MTX	TOFA 5mg + MTX	0.86682	0.56005	1.34548
TOFA 5mg + MTX	TOFA 5mg + MTX	1	1	1

Abbreviations: ABA abatacept; ADA adalimumab; BARI baricitinib; ETN etanercept; GOL golimumab; IFX infliximab; MTX methotrexate; PBO placebo; RTX rituximab; SARI sarilumab; SUBCUT subcutaneous; TCZ tocilizumab; TOFA tofacitinib.

[1]statistically significantly favouring BARI.

Odds ratios >1 are in favour of Treatment 1; and odds ratios <1 are in favour of Treatment 2.

10.2.3. ACR70 Response

Table S23 Sensitivity analysis including trials with prior bDMARD use of up to 20%: Relative treatment effect of pairwise comparisons expressed as Post. Median odds ratios (with 95% CrIs) – ACR70 response at week 24: MTX-IR (Simultaneous Fixed-effects model)

Treatment 1	Treatment 2	Median OR	95% CrI Lower	95% CrI Upper
PBO + MTX	PBO + MTX	1	1	1
**BARI 4mg + MTX[1]**	**PBO + MTX**	**6.21014**	**4.42976**	**8.81452**
ADA 40mg + MTX	PBO + MTX	4.58825	3.42443	6.18615
ABA 10mg + MTX	PBO + MTX	3.32687	2.22952	5.04195
ABA SUBCUT + MTX	PBO + MTX	4.42208	2.76583	7.07758
IFX 3mg + MTX	PBO + MTX	3.71296	2.47455	5.68175
RTX 1000mg + MTX	PBO + MTX	2.90505	1.56695	5.52338
GOL 50mg + MTX	PBO + MTX	4.74344	2.78178	8.34182
CZP + MTX	PBO + MTX	8.32686	5.42892	13.58279
TCZ 8mg + MTX	PBO + MTX	7.7413	4.6842	13.56946
cDMARD + MTX	PBO + MTX	1.69706	0.64449	4.68677
ETN + MTX	PBO + MTX	6.87779	2.86605	17.41077
SARI 200mg + MTX	PBO + MTX	4.31033	3.05637	6.13804
TOFA 5mg + MTX	PBO + MTX	6.98925	4.7874	10.29918
PBO + MTX	BARI 4mg + MTX	0.16103	0.11345	0.22575
BARI 4mg + MTX	BARI 4mg + MTX	1	1	1
ADA 40mg + MTX	BARI 4mg + MTX	0.73899	0.54471	0.99842
ABA 10mg + MTX	BARI 4mg + MTX	0.53563	0.31546	0.91053
ABA SUBCUT + MTX	BARI 4mg + MTX	0.71069	0.44051	1.14128
IFX 3mg + MTX	BARI 4mg + MTX	0.5987	0.34951	1.02381
RTX 1000mg + MTX	BARI 4mg + MTX	0.46731	0.23244	0.96101
GOL 50mg + MTX	BARI 4mg + MTX	0.76394	0.41057	1.45986
CZP + MTX	BARI 4mg + MTX	1.34424	0.78146	2.3955
TCZ 8mg + MTX	BARI 4mg + MTX	1.24548	0.67596	2.39299
cDMARD + MTX	BARI 4mg + MTX	0.27222	0.09944	0.78376
ETN + MTX	BARI 4mg + MTX	1.10323	0.43711	2.91861
SARI 200mg + MTX	BARI 4mg + MTX	0.69367	0.42641	1.13232
TOFA 5mg + MTX	BARI 4mg + MTX	1.12606	0.74676	1.68326
PBO + MTX	ADA 40mg + MTX	0.21795	0.16165	0.29202
**BARI 4mg + MTX[1]**	**ADA 40mg + MTX**	**1.35321**	**1.00159**	**1.83585**
ADA 40mg + MTX	ADA 40mg + MTX	1	1	1
ABA 10mg + MTX	ADA 40mg + MTX	0.72514	0.44105	1.19788
ABA SUBCUT + MTX	ADA 40mg + MTX	0.96279	0.66275	1.38928
IFX 3mg + MTX	ADA 40mg + MTX	0.81021	0.4901	1.34816
RTX 1000mg + MTX	ADA 40mg + MTX	0.63177	0.32443	1.26628
GOL 50mg + MTX	ADA 40mg + MTX	1.03482	0.5672	1.93573
CZP + MTX	ADA 40mg + MTX	1.81387	1.09684	3.14225
TCZ 8mg + MTX	ADA 40mg + MTX	1.68681	0.9459	3.15286
cDMARD + MTX	ADA 40mg + MTX	0.36912	0.13743	1.03661
ETN + MTX	ADA 40mg + MTX	1.49331	0.60899	3.8783
SARI 200mg + MTX	ADA 40mg + MTX	0.94037	0.5948	1.48988
TOFA 5mg + MTX	ADA 40mg + MTX	1.52386	1.1514	2.01755
PBO + MTX	ABA 10mg + MTX	0.30058	0.19834	0.44853
**BARI 4mg + MTX[1]**	**ABA 10mg + MTX**	**1.86697**	**1.09826**	**3.17**

Treatment 1	Treatment 2	Median OR	95% CrI Lower	95% CrI Upper
ADA 40mg + MTX	ABA 10mg + MTX	1.37904	0.83481	2.26734
ABA 10mg + MTX	ABA 10mg + MTX	1	1	1
ABA SUBCUT + MTX	ABA 10mg + MTX	1.32614	0.71544	2.46851
IFX 3mg + MTX	ABA 10mg + MTX	1.1183	0.71823	1.72602
RTX 1000mg + MTX	ABA 10mg + MTX	0.87564	0.41671	1.85469
GOL 50mg + MTX	ABA 10mg + MTX	1.42562	0.72586	2.83534
CZP + MTX	ABA 10mg + MTX	2.50669	1.38285	4.67101
TCZ 8mg + MTX	ABA 10mg + MTX	2.32693	1.21334	4.62482
cDMARD + MTX	ABA 10mg + MTX	0.50771	0.17903	1.5208
ETN + MTX	ABA 10mg + MTX	2.05802	0.78726	5.66625
SARI 200mg + MTX	ABA 10mg + MTX	1.29648	0.75744	2.20831
TOFA 5mg + MTX	ABA 10mg + MTX	2.09959	1.20454	3.6667
PBO + MTX	ABA SUBCUT + MTX	0.22614	0.14129	0.36155
**BARI 4mg + MTX**	**ABA SUBCUT + MTX**	**1.40708**	**0.87621**	**2.27012**
ADA 40mg + MTX	ABA SUBCUT + MTX	1.03865	0.7198	1.50886
ABA 10mg + MTX	ABA SUBCUT + MTX	0.75407	0.4051	1.39773
ABA SUBCUT + MTX	ABA SUBCUT + MTX	1	1	1
IFX 3mg + MTX	ABA SUBCUT + MTX	0.84295	0.4514	1.57895
RTX 1000mg + MTX	ABA SUBCUT + MTX	0.65767	0.30851	1.44292
GOL 50mg + MTX	ABA SUBCUT + MTX	1.07727	0.53102	2.22381
CZP + MTX	ABA SUBCUT + MTX	1.89571	1.0115	3.62642
TCZ 8mg + MTX	ABA SUBCUT + MTX	1.75643	0.88873	3.61398
cDMARD + MTX	ABA SUBCUT + MTX	0.38285	0.13412	1.15269
ETN + MTX	ABA SUBCUT + MTX	1.54975	0.59801	4.33397
SARI 200mg + MTX	ABA SUBCUT + MTX	0.97733	0.54639	1.75442
TOFA 5mg + MTX	ABA SUBCUT + MTX	1.58011	0.998	2.52479
PBO + MTX	IFX 3mg + MTX	0.26933	0.176	0.40411
**BARI 4mg + MTX**	**IFX 3mg + MTX**	**1.67029**	**0.97674**	**2.86112**
ADA 40mg + MTX	IFX 3mg + MTX	1.23425	0.74175	2.04038
ABA 10mg + MTX	IFX 3mg + MTX	0.89422	0.57937	1.3923
ABA SUBCUT + MTX	IFX 3mg + MTX	1.18631	0.63333	2.21534
IFX 3mg + MTX	IFX 3mg + MTX	1	1	1
RTX 1000mg + MTX	IFX 3mg + MTX	0.78261	0.37205	1.664
GOL 50mg + MTX	IFX 3mg + MTX	1.27638	0.64666	2.56369
CZP + MTX	IFX 3mg + MTX	2.24302	1.23868	4.18801
TCZ 8mg + MTX	IFX 3mg + MTX	2.08821	1.08183	4.15328
cDMARD + MTX	IFX 3mg + MTX	0.45509	0.1611	1.34926
ETN + MTX	IFX 3mg + MTX	1.83743	0.71284	5.05965
SARI 200mg + MTX	IFX 3mg + MTX	1.16119	0.67462	1.9854
TOFA 5mg + MTX	IFX 3mg + MTX	1.87668	1.07225	3.28402
PBO + MTX	RTX 1000mg + MTX	0.34423	0.18105	0.63818
**BARI 4mg + MTX[1]**	**RTX 1000mg + MTX**	**2.1399**	**1.04057**	**4.30218**
ADA 40mg + MTX	RTX 1000mg + MTX	1.58287	0.78972	3.08235
ABA 10mg + MTX	RTX 1000mg + MTX	1.14202	0.53917	2.39974
ABA SUBCUT + MTX	RTX 1000mg + MTX	1.52053	0.69304	3.24135
IFX 3mg + MTX	RTX 1000mg + MTX	1.27778	0.60096	2.68781
RTX 1000mg + MTX	RTX 1000mg + MTX	1	1	1
GOL 50mg + MTX	RTX 1000mg + MTX	1.63562	0.72024	3.71365
CZP + MTX	RTX 1000mg + MTX	2.87632	1.3584	6.09418
TCZ 8mg + MTX	RTX 1000mg + MTX	2.66991	1.18472	6.00828
cDMARD + MTX	RTX 1000mg + MTX	0.58256	0.18728	1.83563
ETN + MTX	RTX 1000mg + MTX	2.35472	0.8256	6.98465
SARI 200mg + MTX	RTX 1000mg + MTX	1.48131	0.71229	3.05109
TOFA 5mg + MTX	RTX 1000mg + MTX	2.40889	1.15987	4.89592
PBO + MTX	GOL 50mg + MTX	0.21082	0.11988	0.35948
**BARI 4mg + MTX**	**GOL 50mg + MTX**	**1.30899**	**0.685**	**2.43563**

Treatment 1	Treatment 2	Median OR	95% CrI Lower	95% CrI Upper
ADA 40mg + MTX	GOL 50mg + MTX	0.96635	0.5166	1.76306
ABA 10mg + MTX	GOL 50mg + MTX	0.70145	0.35269	1.37767
ABA SUBCUT + MTX	GOL 50mg + MTX	0.92827	0.44968	1.88317
IFX 3mg + MTX	GOL 50mg + MTX	0.78347	0.39006	1.5464
RTX 1000mg + MTX	GOL 50mg + MTX	0.61139	0.26928	1.38842
GOL 50mg + MTX	GOL 50mg + MTX	1	1	1
CZP + MTX	GOL 50mg + MTX	1.75685	0.88286	3.52941
TCZ 8mg + MTX	GOL 50mg + MTX	1.63659	0.77318	3.50045
cDMARD + MTX	GOL 50mg + MTX	0.35671	0.11972	1.0907
ETN + MTX	GOL 50mg + MTX	1.44747	0.52758	4.12747
SARI 200mg + MTX	GOL 50mg + MTX	0.90941	0.46668	1.72805
TOFA 5mg + MTX	GOL 50mg + MTX	1.47017	0.75467	2.80173
PBO + MTX	CZP + MTX	0.12009	0.07362	0.1842
**BARI 4mg + MTX**	**CZP + MTX**	**0.74391**	**0.41745**	**1.27966**
ADA 40mg + MTX	CZP + MTX	0.55131	0.31824	0.91171
ABA 10mg + MTX	CZP + MTX	0.39893	0.21409	0.72314
ABA SUBCUT + MTX	CZP + MTX	0.52751	0.27575	0.98863
IFX 3mg + MTX	CZP + MTX	0.44583	0.23878	0.80731
RTX 1000mg + MTX	CZP + MTX	0.34767	0.16409	0.73616
GOL 50mg + MTX	CZP + MTX	0.5692	0.28333	1.13269
CZP + MTX	CZP + MTX	1	1	1
TCZ 8mg + MTX	CZP + MTX	0.92861	0.47681	1.83099
cDMARD + MTX	CZP + MTX	0.20291	0.07175	0.57852
ETN + MTX	CZP + MTX	0.82369	0.31731	2.17246
SARI 200mg + MTX	CZP + MTX	0.51753	0.28282	0.90726
TOFA 5mg + MTX	CZP + MTX	0.83765	0.46628	1.45809
PBO + MTX	TCZ 8mg + MTX	0.12918	0.07369	0.21348
**BARI 4mg + MTX**	**TCZ 8mg + MTX**	**0.8029**	**0.41789**	**1.47939**
ADA 40mg + MTX	TCZ 8mg + MTX	0.59284	0.31717	1.05719
ABA 10mg + MTX	TCZ 8mg + MTX	0.42975	0.21622	0.82417
ABA SUBCUT + MTX	TCZ 8mg + MTX	0.56934	0.2767	1.12521
IFX 3mg + MTX	TCZ 8mg + MTX	0.47888	0.24077	0.92436
RTX 1000mg + MTX	TCZ 8mg + MTX	0.37454	0.16644	0.84408
GOL 50mg + MTX	TCZ 8mg + MTX	0.61103	0.28568	1.29336
CZP + MTX	TCZ 8mg + MTX	1.07688	0.54615	2.09726
TCZ 8mg + MTX	TCZ 8mg + MTX	1	1	1
cDMARD + MTX	TCZ 8mg + MTX	0.21801	0.07374	0.65852
ETN + MTX	TCZ 8mg + MTX	0.88432	0.32122	2.46692
SARI 200mg + MTX	TCZ 8mg + MTX	0.55674	0.28702	1.03638
TOFA 5mg + MTX	TCZ 8mg + MTX	0.90268	0.4643	1.6837
PBO + MTX	cDMARD + MTX	0.58925	0.21337	1.55162
**BARI 4mg + MTX[1]**	**cDMARD + MTX**	**3.67348**	**1.27591**	**10.05613**
ADA 40mg + MTX	cDMARD + MTX	2.70915	0.96469	7.27646
ABA 10mg + MTX	cDMARD + MTX	1.96962	0.65755	5.58578
ABA SUBCUT + MTX	cDMARD + MTX	2.61196	0.86753	7.45598
IFX 3mg + MTX	cDMARD + MTX	2.19736	0.74114	6.2075
RTX 1000mg + MTX	cDMARD + MTX	1.71655	0.54477	5.33966
GOL 50mg + MTX	cDMARD + MTX	2.80343	0.91684	8.35283
CZP + MTX	cDMARD + MTX	4.92823	1.72854	13.93764
TCZ 8mg + MTX	cDMARD + MTX	4.5869	1.51855	13.56166
cDMARD + MTX	cDMARD + MTX	1	1	1
ETN + MTX	cDMARD + MTX	4.03019	2.56495	6.64515
SARI 200mg + MTX	cDMARD + MTX	2.54929	0.86369	7.14762
TOFA 5mg + MTX	cDMARD + MTX	4.12254	1.43655	11.39452
PBO + MTX	ETN + MTX	0.1454	0.05744	0.34891
**BARI 4mg + MTX**	**ETN + MTX**	**0.90643**	**0.34263**	**2.28773**

Treatment 1	Treatment 2	Median OR	95% CrI Lower	95% CrI Upper
ADA 40mg + MTX	ETN + MTX	0.66966	0.25785	1.64207
ABA 10mg + MTX	ETN + MTX	0.4859	0.17648	1.27023
ABA SUBCUT + MTX	ETN + MTX	0.64527	0.23074	1.67223
IFX 3mg + MTX	ETN + MTX	0.54424	0.19764	1.40284
RTX 1000mg + MTX	ETN + MTX	0.42468	0.14317	1.21124
GOL 50mg + MTX	ETN + MTX	0.69086	0.24228	1.89546
CZP + MTX	ETN + MTX	1.21405	0.46031	3.15146
TCZ 8mg + MTX	ETN + MTX	1.13081	0.40536	3.11316
cDMARD + MTX	ETN + MTX	0.24813	0.15049	0.38987
ETN + MTX	ETN + MTX	1	1	1
SARI 200mg + MTX	ETN + MTX	0.62698	0.2312	1.61207
TOFA 5mg + MTX	ETN + MTX	1.01977	0.38195	2.5614
PBO + MTX	SARI 200mg + MTX	0.232	0.16292	0.32719
**BARI 4mg + MTX**	**SARI 200mg + MTX**	**1.44161**	**0.88314**	**2.34518**
ADA 40mg + MTX	SARI 200mg + MTX	1.06341	0.67119	1.68123
ABA 10mg + MTX	SARI 200mg + MTX	0.77132	0.45283	1.32023
ABA SUBCUT + MTX	SARI 200mg + MTX	1.02319	0.56999	1.83021
IFX 3mg + MTX	SARI 200mg + MTX	0.86119	0.50368	1.48231
RTX 1000mg + MTX	SARI 200mg + MTX	0.67508	0.32775	1.40393
GOL 50mg + MTX	SARI 200mg + MTX	1.09961	0.57869	2.14282
CZP + MTX	SARI 200mg + MTX	1.93225	1.10223	3.53582
TCZ 8mg + MTX	SARI 200mg + MTX	1.79618	0.9649	3.48409
cDMARD + MTX	SARI 200mg + MTX	0.39227	0.13991	1.15782
ETN + MTX	SARI 200mg + MTX	1.59495	0.62032	4.32526
SARI 200mg + MTX	SARI 200mg + MTX	1	1	1
TOFA 5mg + MTX	SARI 200mg + MTX	1.62155	0.96764	2.71326
PBO + MTX	TOFA 5mg + MTX	0.14308	0.0971	0.20888
**BARI 4mg + MTX**	**TOFA 5mg + MTX**	**0.88805**	**0.59409**	**1.33912**
ADA 40mg + MTX	TOFA 5mg + MTX	0.65623	0.49565	0.86851
ABA 10mg + MTX	TOFA 5mg + MTX	0.47628	0.27272	0.83019
ABA SUBCUT + MTX	TOFA 5mg + MTX	0.63287	0.39607	1.002
IFX 3mg + MTX	TOFA 5mg + MTX	0.53285	0.3045	0.93262
RTX 1000mg + MTX	TOFA 5mg + MTX	0.41513	0.20425	0.86217
GOL 50mg + MTX	TOFA 5mg + MTX	0.68019	0.35692	1.32509
CZP + MTX	TOFA 5mg + MTX	1.19382	0.68583	2.14463
TCZ 8mg + MTX	TOFA 5mg + MTX	1.10782	0.59393	2.15376
cDMARD + MTX	TOFA 5mg + MTX	0.24257	0.08776	0.69611
ETN + MTX	TOFA 5mg + MTX	0.98062	0.39041	2.61816
SARI 200mg + MTX	TOFA 5mg + MTX	0.61669	0.36856	1.03344
TOFA 5mg + MTX	TOFA 5mg + MTX	1	1	1

Abbreviations: ABA abatacept; ADA adalimumab; BARI baricitinib; ETN etanercept; GOL golimumab; IFX infliximab; MTX methotrexate; PBO placebo; RTX rituximab; SARI sarilumab; SUBCUT subcutaneous; TCZ tocilizumab; TOFA tofacitinib.

[1]statistically significantly favouring BARI.

Odds ratios >1 are in favour of Treatment 1; and odds ratios <1 are in favour of Treatment 2.

10.3. Trials Conducted in Asia Pacific/Low Dose MTX

10.3.1. ACR20 response

Figure S3 Sensitivity analysis excluding trials conducted solely in Asia-Pacific and/or low MTX dose: Network of Evidence - Simultaneous fixed effects: ACR20

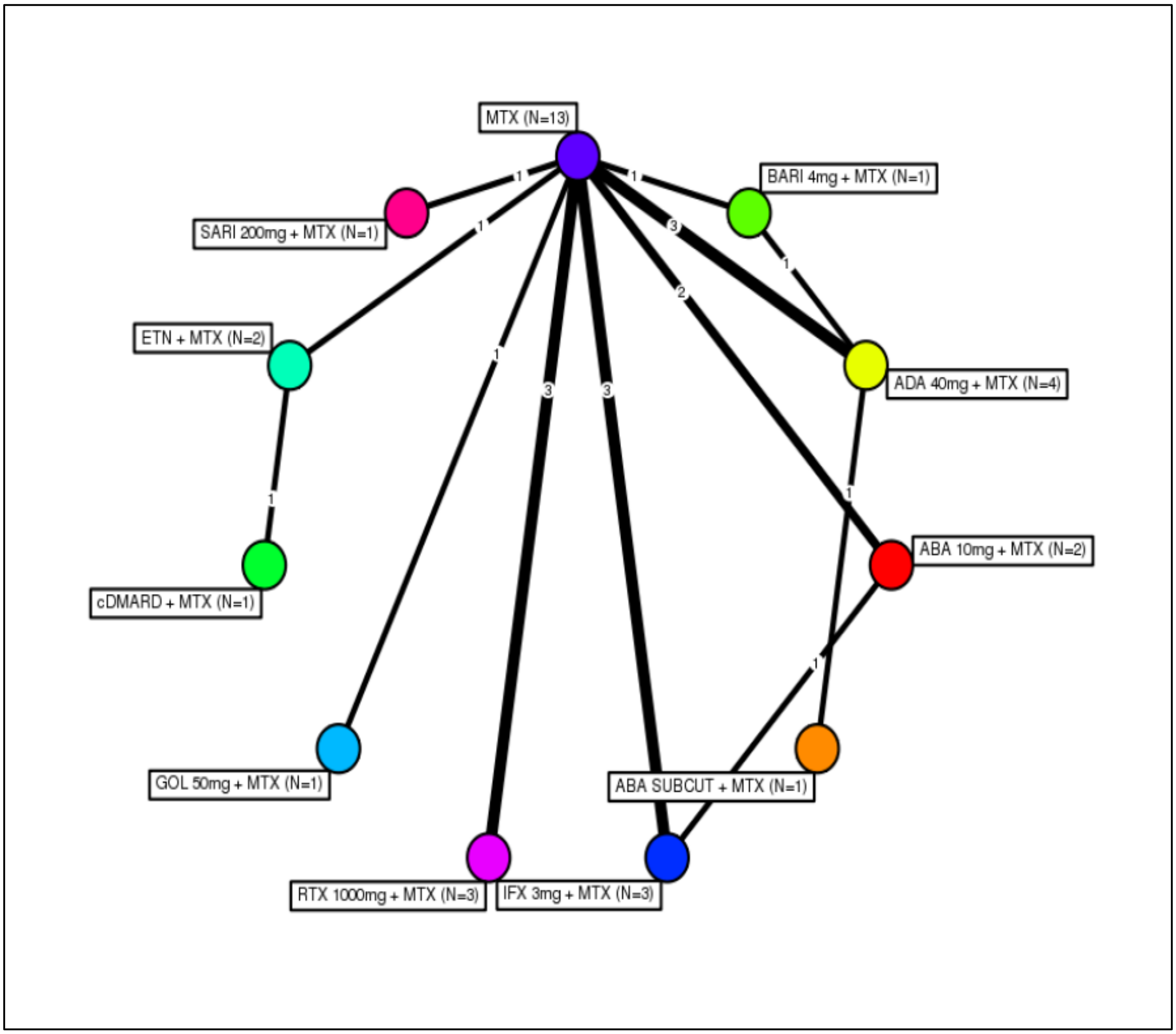

Note: Sensitivity excluding trials conducted solely in Asia-Pacific and/or low MTX dose (N=15 trials for ACR20 and N=14 trials for ACR50/70 (minus ATTRACT [IFX])

Table S24 Sensitivity analysis excluding trials conducted solely in Asia-Pacific and/or low MTX dose: Relative treatment effect of pairwise comparisons expressed as Post. Median odds ratios (with 95% CrIs) – ACR20 response at week 24: MTX-IR (Simultaneous Fixed-effects model)

Treatment 1	Treatment 2	Median OR	95% CrI Lower	95% CrI Upper
MTX	MTX	1	1	1
**BARI 4mg + MTX**	**MTX**	**5.37048**	**4.16293**	**6.97437**
ADA 40mg + MTX	MTX	4.04653	3.30839	4.96635
ABA 10mg + MTX	MTX	3.73632	2.86435	4.84977
ABA SUBCUT + MTX	MTX	4.14362	2.90908	5.89238
IFX 3mg + MTX	MTX	3.19234	2.55836	3.99711
RTX 1000mg + MTX	MTX	3.10623	2.26495	4.33549
GOL 50mg + MTX	MTX	3.62315	2.17581	6.12803
cDMARD + MTX	MTX	1.95595	1.10581	3.29082
ETN + MTX	MTX	8.9441	5.53275	14.09385
SARI 200mg + MTX	MTX	4.15888	3.00172	5.75745
MTX	BARI 4mg + MTX	0.1862	0.14338	0.24022
BARI 4mg + MTX	BARI 4mg + MTX	1	1	1
ADA 40mg + MTX	BARI 4mg + MTX	0.75281	0.5718	0.99825
ABA 10mg + MTX	BARI 4mg + MTX	0.69561	0.48486	0.98986
ABA SUBCUT + MTX	BARI 4mg + MTX	0.77098	0.51537	1.15413
IFX 3mg + MTX	BARI 4mg + MTX	0.59349	0.42563	0.83334
RTX 1000mg + MTX	BARI 4mg + MTX	0.57916	0.38661	0.87746
GOL 50mg + MTX	BARI 4mg + MTX	0.6743	0.3831	1.20797
cDMARD + MTX	BARI 4mg + MTX	0.36341	0.1975	0.6459
ETN + MTX	BARI 4mg + MTX	1.66131	0.98309	2.78207
SARI 200mg + MTX	BARI 4mg + MTX	0.7746	0.51413	1.1619
MTX	ADA 40mg + MTX	0.24713	0.20136	0.30226
**BARI 4mg + MTX**	**ADA 40mg + MTX**	**1.32836**	**1.00175**	**1.74886**
ADA 40mg + MTX	ADA 40mg + MTX	1	1	1
ABA 10mg + MTX	ADA 40mg + MTX	0.92378	0.66352	1.26569
ABA SUBCUT + MTX	ADA 40mg + MTX	1.02325	0.75395	1.39196
IFX 3mg + MTX	ADA 40mg + MTX	0.7877	0.58913	1.05786
RTX 1000mg + MTX	ADA 40mg + MTX	0.76894	0.53386	1.12031
GOL 50mg + MTX	ADA 40mg + MTX	0.89495	0.52492	1.56451
cDMARD + MTX	ADA 40mg + MTX	0.48361	0.26706	0.83406
ETN + MTX	ADA 40mg + MTX	2.20984	1.33392	3.57495
SARI 200mg + MTX	ADA 40mg + MTX	1.02854	0.70288	1.50034
MTX	ABA 10mg + MTX	0.26764	0.2062	0.34912
**BARI 4mg + MTX**	**ABA 10mg + MTX**	**1.43759**	**1.01025**	**2.06244**
ADA 40mg + MTX	ABA 10mg + MTX	1.08251	0.79008	1.50711
ABA 10mg + MTX	ABA 10mg + MTX	1	1	1
ABA SUBCUT + MTX	ABA 10mg + MTX	1.10821	0.72674	1.70557
IFX 3mg + MTX	ABA 10mg + MTX	0.85461	0.62356	1.17594
RTX 1000mg + MTX	ABA 10mg + MTX	0.83263	0.55116	1.27331
GOL 50mg + MTX	ABA 10mg + MTX	0.97046	0.54768	1.75083
cDMARD + MTX	ABA 10mg + MTX	0.52148	0.28706	0.92376
ETN + MTX	ABA 10mg + MTX	2.38602	1.433	4.01141
SARI 200mg + MTX	ABA 10mg + MTX	1.11273	0.74206	1.68621
MTX	ABA SUBCUT + MTX	0.24134	0.16971	0.34375
**BARI 4mg + MTX**	**ABA SUBCUT + MTX**	**1.29705**	**0.86645**	**1.94037**
ADA 40mg + MTX	ABA SUBCUT + MTX	0.97728	0.71841	1.32635
ABA 10mg + MTX	ABA SUBCUT + MTX	0.90235	0.58631	1.376
ABA SUBCUT + MTX	ABA SUBCUT + MTX	1	1	1
IFX 3mg + MTX	ABA SUBCUT + MTX	0.7698	0.51369	1.15968
RTX 1000mg + MTX	ABA SUBCUT + MTX	0.75093	0.47582	1.20294
GOL 50mg + MTX	ABA SUBCUT + MTX	0.87461	0.47519	1.64567

Treatment 1	Treatment 2	Median OR	95% CrI Lower	95% CrI Upper
cDMARD + MTX	ABA SUBCUT + MTX	0.47109	0.24668	0.8694
ETN + MTX	ABA SUBCUT + MTX	2.15673	1.21811	3.7406
SARI 200mg + MTX	ABA SUBCUT + MTX	1.00468	0.62345	1.60418
MTX	IFX 3mg + MTX	0.31325	0.25018	0.39087
**BARI 4mg + MTX**	**IFX 3mg + MTX**	**1.68494**	**1.19999**	**2.34948**
ADA 40mg + MTX	IFX 3mg + MTX	1.26952	0.9453	1.69742
ABA 10mg + MTX	IFX 3mg + MTX	1.17012	0.85038	1.60371
ABA SUBCUT + MTX	IFX 3mg + MTX	1.29904	0.86231	1.94671
IFX 3mg + MTX	IFX 3mg + MTX	1	1	1
RTX 1000mg + MTX	IFX 3mg + MTX	0.97507	0.67149	1.42778
GOL 50mg + MTX	IFX 3mg + MTX	1.13585	0.65765	1.99493
cDMARD + MTX	IFX 3mg + MTX	0.61382	0.33277	1.06373
ETN + MTX	IFX 3mg + MTX	2.80805	1.66408	4.5755
SARI 200mg + MTX	IFX 3mg + MTX	1.30365	0.87628	1.92489
MTX	RTX 1000mg + MTX	0.32193	0.23065	0.44151
**BARI 4mg + MTX**	**RTX 1000mg + MTX**	**1.72665**	**1.13965**	**2.58658**
ADA 40mg + MTX	RTX 1000mg + MTX	1.30049	0.89261	1.87313
ABA 10mg + MTX	RTX 1000mg + MTX	1.20101	0.78535	1.81434
ABA SUBCUT + MTX	RTX 1000mg + MTX	1.33169	0.8313	2.10162
IFX 3mg + MTX	RTX 1000mg + MTX	1.02557	0.70039	1.48923
RTX 1000mg + MTX	RTX 1000mg + MTX	1	1	1
GOL 50mg + MTX	RTX 1000mg + MTX	1.16663	0.64197	2.12786
cDMARD + MTX	RTX 1000mg + MTX	0.62863	0.3254	1.14171
ETN + MTX	RTX 1000mg + MTX	2.88301	1.62059	4.91039
SARI 200mg + MTX	RTX 1000mg + MTX	1.33724	0.84281	2.10091
MTX	GOL 50mg + MTX	0.276	0.16318	0.4596
**BARI 4mg + MTX**	**GOL 50mg + MTX**	**1.48303**	**0.82783**	**2.61028**
ADA 40mg + MTX	GOL 50mg + MTX	1.11738	0.63918	1.90507
ABA 10mg + MTX	GOL 50mg + MTX	1.03044	0.57116	1.82588
ABA SUBCUT + MTX	GOL 50mg + MTX	1.14337	0.60765	2.10443
IFX 3mg + MTX	GOL 50mg + MTX	0.8804	0.50127	1.52057
RTX 1000mg + MTX	GOL 50mg + MTX	0.85717	0.46996	1.55772
GOL 50mg + MTX	GOL 50mg + MTX	1	1	1
cDMARD + MTX	GOL 50mg + MTX	0.53894	0.25093	1.10286
ETN + MTX	GOL 50mg + MTX	2.4639	1.22371	4.79625
SARI 200mg + MTX	GOL 50mg + MTX	1.14785	0.62058	2.08488
MTX	cDMARD + MTX	0.51126	0.30388	0.90431
**BARI 4mg + MTX**	**cDMARD + MTX**	**2.75168**	**1.54824**	**5.06338**
ADA 40mg + MTX	cDMARD + MTX	2.06779	1.19895	3.74449
ABA 10mg + MTX	cDMARD + MTX	1.91764	1.08253	3.48364
ABA SUBCUT + MTX	cDMARD + MTX	2.12274	1.15022	4.05383
IFX 3mg + MTX	cDMARD + MTX	1.62914	0.94008	3.00511
RTX 1000mg + MTX	cDMARD + MTX	1.59075	0.87588	3.07315
GOL 50mg + MTX	cDMARD + MTX	1.8555	0.90673	3.98519
cDMARD + MTX	cDMARD + MTX	1	1	1
ETN + MTX	cDMARD + MTX	4.58387	2.98054	7.15383
SARI 200mg + MTX	cDMARD + MTX	2.1313	1.15538	4.04219
MTX	ETN + MTX	0.11181	0.07095	0.18074
**BARI 4mg + MTX**	**ETN + MTX**	**0.60193**	**0.35944**	**1.0172**
ADA 40mg + MTX	ETN + MTX	0.45252	0.27972	0.74967
ABA 10mg + MTX	ETN + MTX	0.41911	0.24929	0.69783
ABA SUBCUT + MTX	ETN + MTX	0.46366	0.26734	0.82095
IFX 3mg + MTX	ETN + MTX	0.35612	0.21856	0.60093
RTX 1000mg + MTX	ETN + MTX	0.34686	0.20365	0.61706
GOL 50mg + MTX	ETN + MTX	0.40586	0.2085	0.81718
cDMARD + MTX	ETN + MTX	0.21816	0.13979	0.33551

Treatment 1	Treatment 2	Median OR	95% CrI Lower	95% CrI Upper
ETN + MTX	ETN + MTX	1	1	1
SARI 200mg + MTX	ETN + MTX	0.46684	0.26562	0.81718
MTX	SARI 200mg + MTX	0.24045	0.17369	0.33314
**BARI 4mg + MTX**	**SARI 200mg + MTX**	**1.29099**	**0.86066**	**1.94505**
ADA 40mg + MTX	SARI 200mg + MTX	0.97225	0.66652	1.42272
ABA 10mg + MTX	SARI 200mg + MTX	0.89869	0.59304	1.34761
ABA SUBCUT + MTX	SARI 200mg + MTX	0.99534	0.62337	1.60399
IFX 3mg + MTX	SARI 200mg + MTX	0.76708	0.51951	1.14118
RTX 1000mg + MTX	SARI 200mg + MTX	0.74781	0.47599	1.1865
GOL 50mg + MTX	SARI 200mg + MTX	0.87119	0.47964	1.61139
cDMARD + MTX	SARI 200mg + MTX	0.4692	0.24739	0.86552
ETN + MTX	SARI 200mg + MTX	2.14208	1.22373	3.76474
SARI 200mg + MTX	SARI 200mg + MTX	1	1	1

10.3.2. ACR50 response

Table S25 Sensitivity analysis excluding trials conducted solely in Asia-Pacific and/or low MTX dose: Relative treatment effect of pairwise comparisons expressed as Post. Median odds ratios (with 95% CrIs) – ACR50 response at week 24: MTX-IR (Simultaneous Fixed-effects model)

Treatment 1	Treatment 2	Median OR	95% CrI Lower	95% CrI Upper
MTX	MTX	1	1	1
**BARI 4mg + MTX**	**MTX**	**4.91902**	**3.75386**	**6.46411**
ADA 40mg + MTX	MTX	4.46572	3.51362	5.70599
ABA 10mg + MTX	MTX	3.48194	2.58922	4.70285
ABA SUBCUT + MTX	MTX	4.43214	3.01747	6.51707
IFX 3mg + MTX	MTX	3.44204	2.56947	4.6451
RTX 1000mg + MTX	MTX	3.13834	2.07935	4.84871
GOL 50mg + MTX	MTX	3.70393	2.02353	6.82704
cDMARD + MTX	MTX	1.38322	0.64269	3.1433
ETN + MTX	MTX	7.35481	3.84743	15.37692
SARI 200mg + MTX	MTX	4.39488	3.1141	6.2253
MTX	BARI 4mg + MTX	0.20329	0.1547	0.26639
BARI 4mg + MTX	BARI 4mg + MTX	1	1	1
ADA 40mg + MTX	BARI 4mg + MTX	0.90752	0.69779	1.1789
ABA 10mg + MTX	BARI 4mg + MTX	0.70844	0.47417	1.0588
ABA SUBCUT + MTX	BARI 4mg + MTX	0.90093	0.60026	1.34672
IFX 3mg + MTX	BARI 4mg + MTX	0.7001	0.47011	1.04351
RTX 1000mg + MTX	BARI 4mg + MTX	0.63908	0.39234	1.06024
GOL 50mg + MTX	BARI 4mg + MTX	0.7536	0.38906	1.46185
cDMARD + MTX	BARI 4mg + MTX	0.28136	0.12626	0.66247
ETN + MTX	BARI 4mg + MTX	1.49391	0.75062	3.22924
SARI 200mg + MTX	BARI 4mg + MTX	0.89404	0.57709	1.38573
MTX	ADA 40mg + MTX	0.22393	0.17525	0.28461
**BARI 4mg + MTX**	**ADA 40mg + MTX**	**1.1019**	**0.84824**	**1.43311**
ADA 40mg + MTX	ADA 40mg + MTX	1	1	1
ABA 10mg + MTX	ADA 40mg + MTX	0.7815	0.5329	1.13377
ABA SUBCUT + MTX	ADA 40mg + MTX	0.99333	0.72713	1.35705
IFX 3mg + MTX	ADA 40mg + MTX	0.77041	0.53121	1.12594
RTX 1000mg + MTX	ADA 40mg + MTX	0.70352	0.44247	1.14264
GOL 50mg + MTX	ADA 40mg + MTX	0.83152	0.43202	1.58855
cDMARD + MTX	ADA 40mg + MTX	0.31006	0.14077	0.71576
ETN + MTX	ADA 40mg + MTX	1.64609	0.84125	3.46877
SARI 200mg + MTX	ADA 40mg + MTX	0.98503	0.64435	1.50432
MTX	ABA 10mg + MTX	0.2872	0.21264	0.38622
**BARI 4mg + MTX**	**ABA 10mg + MTX**	**1.41155**	**0.94447**	**2.10893**
ADA 40mg + MTX	ABA 10mg + MTX	1.2796	0.88201	1.87653
ABA 10mg + MTX	ABA 10mg + MTX	1	1	1
ABA SUBCUT + MTX	ABA 10mg + MTX	1.27182	0.78634	2.06184
IFX 3mg + MTX	ABA 10mg + MTX	0.98738	0.69842	1.4006
RTX 1000mg + MTX	ABA 10mg + MTX	0.90207	0.54673	1.51893
GOL 50mg + MTX	ABA 10mg + MTX	1.06442	0.53927	2.09933
cDMARD + MTX	ABA 10mg + MTX	0.39693	0.17707	0.94152
ETN + MTX	ABA 10mg + MTX	2.11145	1.0493	4.60216
SARI 200mg + MTX	ABA 10mg + MTX	1.26079	0.7974	1.99003
MTX	ABA SUBCUT + MTX	0.22562	0.15344	0.3314

Treatment 1	Treatment 2	Median OR	95% CrI Lower	95% CrI Upper
**BARI 4mg + MTX**	**ABA SUBCUT + MTX**	**1.10996**	**0.74254**	**1.66596**
ADA 40mg + MTX	ABA SUBCUT + MTX	1.00671	0.73689	1.37528
ABA 10mg + MTX	ABA SUBCUT + MTX	0.78627	0.485	1.27172
ABA SUBCUT + MTX	ABA SUBCUT + MTX	1	1	1
IFX 3mg + MTX	ABA SUBCUT + MTX	0.77615	0.48202	1.26144
RTX 1000mg + MTX	ABA SUBCUT + MTX	0.70823	0.41129	1.25565
GOL 50mg + MTX	ABA SUBCUT + MTX	0.83691	0.40963	1.71735
cDMARD + MTX	ABA SUBCUT + MTX	0.31332	0.13438	0.75612
ETN + MTX	ABA SUBCUT + MTX	1.6601	0.79646	3.67486
SARI 200mg + MTX	ABA SUBCUT + MTX	0.99144	0.59026	1.6594
MTX	IFX 3mg + MTX	0.29053	0.21528	0.38919
**BARI 4mg + MTX**	**IFX 3mg + MTX**	**1.42837**	**0.95831**	**2.12716**
ADA 40mg + MTX	IFX 3mg + MTX	1.29801	0.88815	1.88249
ABA 10mg + MTX	IFX 3mg + MTX	1.01278	0.71398	1.4318
ABA SUBCUT + MTX	IFX 3mg + MTX	1.28841	0.79275	2.07458
IFX 3mg + MTX	IFX 3mg + MTX	1	1	1
RTX 1000mg + MTX	IFX 3mg + MTX	0.91347	0.555	1.52066
GOL 50mg + MTX	IFX 3mg + MTX	1.0766	0.54676	2.11349
cDMARD + MTX	IFX 3mg + MTX	0.40233	0.17828	0.94492
ETN + MTX	IFX 3mg + MTX	2.13778	1.05255	4.61504
SARI 200mg + MTX	IFX 3mg + MTX	1.27657	0.80548	2.00377
MTX	RTX 1000mg + MTX	0.31864	0.20624	0.48092
**BARI 4mg + MTX**	**RTX 1000mg + MTX**	**1.56474**	**0.94319**	**2.54883**
ADA 40mg + MTX	RTX 1000mg + MTX	1.42141	0.87517	2.26006
ABA 10mg + MTX	RTX 1000mg + MTX	1.10856	0.65836	1.82907
ABA SUBCUT + MTX	RTX 1000mg + MTX	1.41197	0.7964	2.43135
IFX 3mg + MTX	RTX 1000mg + MTX	1.09473	0.65761	1.80179
RTX 1000mg + MTX	RTX 1000mg + MTX	1	1	1
GOL 50mg + MTX	RTX 1000mg + MTX	1.17927	0.56903	2.42582
cDMARD + MTX	RTX 1000mg + MTX	0.44167	0.18697	1.05828
ETN + MTX	RTX 1000mg + MTX	2.35378	1.10568	5.14459
SARI 200mg + MTX	RTX 1000mg + MTX	1.39665	0.79521	2.4151
MTX	GOL 50mg + MTX	0.26998	0.14648	0.49419
**BARI 4mg + MTX**	**GOL 50mg + MTX**	**1.32696**	**0.68406**	**2.57033**
ADA 40mg + MTX	GOL 50mg + MTX	1.20261	0.62951	2.31471
ABA 10mg + MTX	GOL 50mg + MTX	0.93948	0.47634	1.85436
ABA SUBCUT + MTX	GOL 50mg + MTX	1.19487	0.58229	2.44122
IFX 3mg + MTX	GOL 50mg + MTX	0.92885	0.47315	1.82897
RTX 1000mg + MTX	GOL 50mg + MTX	0.84798	0.41223	1.75739
GOL 50mg + MTX	GOL 50mg + MTX	1	1	1
cDMARD + MTX	GOL 50mg + MTX	0.37404	0.14113	1.01528
ETN + MTX	GOL 50mg + MTX	1.99218	0.82878	5.01129
SARI 200mg + MTX	GOL 50mg + MTX	1.18692	0.58417	2.39409
MTX	cDMARD + MTX	0.72295	0.31814	1.55595
**BARI 4mg + MTX**	**cDMARD + MTX**	**3.55418**	**1.50949**	**7.92017**
ADA 40mg + MTX	cDMARD + MTX	3.22523	1.39711	7.10359
ABA 10mg + MTX	cDMARD + MTX	2.51936	1.06212	5.6474
ABA SUBCUT + MTX	cDMARD + MTX	3.19167	1.32255	7.44135
IFX 3mg + MTX	cDMARD + MTX	2.4855	1.05829	5.60925
RTX 1000mg + MTX	cDMARD + MTX	2.26415	0.94493	5.34845
GOL 50mg + MTX	cDMARD + MTX	2.67348	0.98495	7.08553
cDMARD + MTX	cDMARD + MTX	1	1	1
ETN + MTX	cDMARD + MTX	5.32332	3.38902	8.45493
SARI 200mg + MTX	cDMARD + MTX	3.18289	1.29418	7.24838
MTX	ETN + MTX	0.13597	0.06503	0.25991
**BARI 4mg + MTX**	**ETN + MTX**	**0.66939**	**0.30967**	**1.33224**

Treatment 1	Treatment 2	Median OR	95% CrI Lower	95% CrI Upper
ADA 40mg + MTX	ETN + MTX	0.6075	0.28829	1.1887
ABA 10mg + MTX	ETN + MTX	0.47361	0.21729	0.95301
ABA SUBCUT + MTX	ETN + MTX	0.60237	0.27212	1.25555
IFX 3mg + MTX	ETN + MTX	0.46777	0.21668	0.95007
RTX 1000mg + MTX	ETN + MTX	0.42485	0.19438	0.90442
GOL 50mg + MTX	ETN + MTX	0.50196	0.19955	1.20659
cDMARD + MTX	ETN + MTX	0.18785	0.11827	0.29507
ETN + MTX	ETN + MTX	1	1	1
SARI 200mg + MTX	ETN + MTX	0.59788	0.26308	1.22316
MTX	SARI 200mg + MTX	0.22754	0.16063	0.32112
**BARI 4mg + MTX**	**SARI 200mg + MTX**	**1.11852**	**0.72164**	**1.73284**
ADA 40mg + MTX	SARI 200mg + MTX	1.0152	0.66475	1.55195
ABA 10mg + MTX	SARI 200mg + MTX	0.79315	0.50251	1.25407
ABA SUBCUT + MTX	SARI 200mg + MTX	1.00863	0.60263	1.69416
IFX 3mg + MTX	SARI 200mg + MTX	0.78335	0.49906	1.2415
RTX 1000mg + MTX	SARI 200mg + MTX	0.716	0.41406	1.25753
GOL 50mg + MTX	SARI 200mg + MTX	0.84251	0.4177	1.71183
cDMARD + MTX	SARI 200mg + MTX	0.31418	0.13796	0.77269
ETN + MTX	SARI 200mg + MTX	1.67257	0.81755	3.80115
SARI 200mg + MTX	SARI 200mg + MTX	1	1	1

10.3.3. ACR70 response

Table S26 Sensitivity analysis excluding trials conducted solely in Asia-Pacific and/or low MTX dose: Relative treatment effect of pairwise comparisons expressed as Post. Median odds ratios (with 95% CrIs) – ACR70 response at week 24: MTX-IR (Simultaneous Fixed-effects model)

Treatment 1	Treatment 2	Median OR	95% CrI Lower	95% CrI Upper
MTX	MTX	1	1	1
**BARI 4mg + MTX**	**MTX**	**5.88738**	**4.13599**	**8.46163**
ADA 40mg + MTX	MTX	4.291	3.07796	6.0451
ABA 10mg + MTX	MTX	3.10864	2.08679	4.68187
ABA SUBCUT + MTX	MTX	4.06656	2.49117	6.70514
IFX 3mg + MTX	MTX	3.46965	2.3322	5.24931
RTX 1000mg + MTX	MTX	2.48715	1.35321	4.71134
GOL 50mg + MTX	MTX	4.32118	1.9055	10.36569
cDMARD + MTX	MTX	1.36336	0.42985	4.9867
ETN + MTX	MTX	5.78645	2.08325	19.43516
SARI 200mg + MTX	MTX	4.27494	3.01698	6.08
MTX	BARI 4mg + MTX	0.16985	0.11818	0.24178
BARI 4mg + MTX	BARI 4mg + MTX	1	1	1
ADA 40mg + MTX	BARI 4mg + MTX	0.72977	0.53584	0.98664
ABA 10mg + MTX	BARI 4mg + MTX	0.52792	0.31078	0.90004
ABA SUBCUT + MTX	BARI 4mg + MTX	0.69117	0.42872	1.11327
IFX 3mg + MTX	BARI 4mg + MTX	0.59	0.34577	1.0111
RTX 1000mg + MTX	BARI 4mg + MTX	0.42243	0.21188	0.86685
GOL 50mg + MTX	BARI 4mg + MTX	0.73614	0.30038	1.85482
cDMARD + MTX	BARI 4mg + MTX	0.23171	0.07005	0.858
ETN + MTX	BARI 4mg + MTX	0.98501	0.33681	3.32157
SARI 200mg + MTX	BARI 4mg + MTX	0.72614	0.43852	1.18727
MTX	ADA 40mg + MTX	0.23305	0.16542	0.32489
**BARI 4mg + MTX**	**ADA 40mg + MTX**	**1.37029**	**1.01354**	**1.86624**
ADA 40mg + MTX	ADA 40mg + MTX	1	1	1
ABA 10mg + MTX	ADA 40mg + MTX	0.72315	0.43217	1.21345
ABA SUBCUT + MTX	ADA 40mg + MTX	0.94728	0.65393	1.37147
IFX 3mg + MTX	ADA 40mg + MTX	0.80864	0.4844	1.36075
RTX 1000mg + MTX	ADA 40mg + MTX	0.57881	0.2948	1.17028
GOL 50mg + MTX	ADA 40mg + MTX	1.00763	0.4161	2.52501
cDMARD + MTX	ADA 40mg + MTX	0.31728	0.09723	1.17116
ETN + MTX	ADA 40mg + MTX	1.34838	0.47036	4.5089
SARI 200mg + MTX	ADA 40mg + MTX	0.99567	0.60774	1.61547
MTX	ABA 10mg + MTX	0.32168	0.21359	0.4792
**BARI 4mg + MTX**	**ABA 10mg + MTX**	**1.89424**	**1.11106**	**3.21776**
ADA 40mg + MTX	ABA 10mg + MTX	1.38283	0.8241	2.31393
ABA 10mg + MTX	ABA 10mg + MTX	1	1	1
ABA SUBCUT + MTX	ABA 10mg + MTX	1.31278	0.69759	2.45599
IFX 3mg + MTX	ABA 10mg + MTX	1.11797	0.72628	1.73653
RTX 1000mg + MTX	ABA 10mg + MTX	0.7989	0.3911	1.69242
GOL 50mg + MTX	ABA 10mg + MTX	1.39261	0.56216	3.65692
cDMARD + MTX	ABA 10mg + MTX	0.4393	0.13026	1.6535
ETN + MTX	ABA 10mg + MTX	1.86273	0.63315	6.51865
SARI 200mg + MTX	ABA 10mg + MTX	1.37509	0.80625	2.34229

Treatment 1	Treatment 2	Median OR	95% CrI Lower	95% CrI Upper
MTX	ABA SUBCUT + MTX	0.24591	0.14914	0.40142
**BARI 4mg + MTX**	**ABA SUBCUT + MTX**	**1.44682**	**0.89826**	**2.33253**
ADA 40mg + MTX	ABA SUBCUT + MTX	1.05565	0.72914	1.52922
ABA 10mg + MTX	ABA SUBCUT + MTX	0.76174	0.40717	1.4335
ABA SUBCUT + MTX	ABA SUBCUT + MTX	1	1	1
IFX 3mg + MTX	ABA SUBCUT + MTX	0.85535	0.45278	1.59743
RTX 1000mg + MTX	ABA SUBCUT + MTX	0.61058	0.28577	1.34177
GOL 50mg + MTX	ABA SUBCUT + MTX	1.06638	0.41394	2.84328
cDMARD + MTX	ABA SUBCUT + MTX	0.33545	0.0972	1.28433
ETN + MTX	ABA SUBCUT + MTX	1.42589	0.47046	4.98796
SARI 200mg + MTX	ABA SUBCUT + MTX	1.05136	0.57141	1.91605
MTX	IFX 3mg + MTX	0.28821	0.1905	0.42878
**BARI 4mg + MTX**	**IFX 3mg + MTX**	**1.69493**	**0.98903**	**2.89213**
ADA 40mg + MTX	IFX 3mg + MTX	1.23664	0.73489	2.06441
ABA 10mg + MTX	IFX 3mg + MTX	0.89448	0.57586	1.37688
ABA SUBCUT + MTX	IFX 3mg + MTX	1.16911	0.62601	2.20859
IFX 3mg + MTX	IFX 3mg + MTX	1	1	1
RTX 1000mg + MTX	IFX 3mg + MTX	0.71571	0.34923	1.51086
GOL 50mg + MTX	IFX 3mg + MTX	1.24469	0.50152	3.23029
cDMARD + MTX	IFX 3mg + MTX	0.39219	0.11662	1.50147
ETN + MTX	IFX 3mg + MTX	1.66469	0.56047	5.80853
SARI 200mg + MTX	IFX 3mg + MTX	1.2316	0.71633	2.09185
MTX	RTX 1000mg + MTX	0.40207	0.21225	0.73898
**BARI 4mg + MTX**	**RTX 1000mg + MTX**	**2.36727**	**1.1536**	**4.71956**
ADA 40mg + MTX	RTX 1000mg + MTX	1.72767	0.8545	3.39209
ABA 10mg + MTX	RTX 1000mg + MTX	1.25172	0.59087	2.55691
ABA SUBCUT + MTX	RTX 1000mg + MTX	1.63778	0.74529	3.49934
IFX 3mg + MTX	RTX 1000mg + MTX	1.39722	0.66187	2.86348
RTX 1000mg + MTX	RTX 1000mg + MTX	1	1	1
GOL 50mg + MTX	RTX 1000mg + MTX	1.74587	0.63129	4.93995
cDMARD + MTX	RTX 1000mg + MTX	0.55082	0.15311	2.14165
ETN + MTX	RTX 1000mg + MTX	2.33626	0.73524	8.32762
SARI 200mg + MTX	RTX 1000mg + MTX	1.72083	0.82628	3.50401
MTX	GOL 50mg + MTX	0.23142	0.09647	0.5248
**BARI 4mg + MTX**	**GOL 50mg + MTX**	**1.35844**	**0.53914**	**3.32909**
ADA 40mg + MTX	GOL 50mg + MTX	0.99243	0.39604	2.40328
ABA 10mg + MTX	GOL 50mg + MTX	0.71807	0.27345	1.77886
ABA SUBCUT + MTX	GOL 50mg + MTX	0.93775	0.35171	2.41582
IFX 3mg + MTX	GOL 50mg + MTX	0.80341	0.30957	1.99393
RTX 1000mg + MTX	GOL 50mg + MTX	0.57278	0.20243	1.58405
GOL 50mg + MTX	GOL 50mg + MTX	1	1	1
cDMARD + MTX	GOL 50mg + MTX	0.31525	0.07646	1.37442
ETN + MTX	GOL 50mg + MTX	1.34106	0.36143	5.49629
SARI 200mg + MTX	GOL 50mg + MTX	0.99042	0.38411	2.41258
MTX	cDMARD + MTX	0.73348	0.20053	2.32637
**BARI 4mg + MTX**	**cDMARD + MTX**	**4.31578**	**1.1655**	**14.27601**
ADA 40mg + MTX	cDMARD + MTX	3.15177	0.85385	10.28475
ABA 10mg + MTX	cDMARD + MTX	2.27635	0.60478	7.67709
ABA SUBCUT + MTX	cDMARD + MTX	2.98104	0.77862	10.28779
IFX 3mg + MTX	cDMARD + MTX	2.54976	0.66601	8.5752
RTX 1000mg + MTX	cDMARD + MTX	1.81548	0.46693	6.53132
GOL 50mg + MTX	cDMARD + MTX	3.17213	0.72758	13.07879
cDMARD + MTX	cDMARD + MTX	1	1	1
ETN + MTX	cDMARD + MTX	4.22977	2.42282	7.80838
SARI 200mg + MTX	cDMARD + MTX	3.14407	0.80513	10.54994
MTX	ETN + MTX	0.17282	0.05145	0.48002

Treatment 1	Treatment 2	Median OR	95% CrI Lower	95% CrI Upper
**BARI 4mg + MTX**	**ETN + MTX**	**1.01522**	**0.30106**	**2.96902**
ADA 40mg + MTX	ETN + MTX	0.74163	0.22178	2.12602
ABA 10mg + MTX	ETN + MTX	0.53685	0.15341	1.57941
ABA SUBCUT + MTX	ETN + MTX	0.70132	0.20048	2.1256
IFX 3mg + MTX	ETN + MTX	0.60071	0.17216	1.78421
RTX 1000mg + MTX	ETN + MTX	0.42803	0.12008	1.36009
GOL 50mg + MTX	ETN + MTX	0.74568	0.18194	2.76681
cDMARD + MTX	ETN + MTX	0.23642	0.12807	0.41274
ETN + MTX	ETN + MTX	1	1	1
SARI 200mg + MTX	ETN + MTX	0.74056	0.20704	2.16986
MTX	SARI 200mg + MTX	0.23392	0.16447	0.33146
**BARI 4mg + MTX**	**SARI 200mg + MTX**	**1.37715**	**0.84227**	**2.28042**
ADA 40mg + MTX	SARI 200mg + MTX	1.00435	0.61902	1.64543
ABA 10mg + MTX	SARI 200mg + MTX	0.72723	0.42693	1.24031
ABA SUBCUT + MTX	SARI 200mg + MTX	0.95115	0.52191	1.75005
IFX 3mg + MTX	SARI 200mg + MTX	0.81195	0.47805	1.39601
RTX 1000mg + MTX	SARI 200mg + MTX	0.58112	0.28539	1.21025
GOL 50mg + MTX	SARI 200mg + MTX	1.00967	0.41449	2.60342
cDMARD + MTX	SARI 200mg + MTX	0.31806	0.09479	1.24204
ETN + MTX	SARI 200mg + MTX	1.35034	0.46086	4.82999
SARI 200mg + MTX	SARI 200mg + MTX	1	1	1

11. Model Fit

Table S27 presents model fit statistics as per the Deviance Information Criterion (DIC) and the overall residual deviance for the primary analyses and main sensitivity analyses.

Table S27 Model Fit Summary

Type of analysis	# of trials [1]	# of data points[1]	ACR20		ACR50		ACR70	
			DIC	$\bar{\mathbf{D}}_{res}$	DIC	$\bar{\mathbf{D}}_{res}$	DIC	$\bar{\mathbf{D}}_{res}$
Primary analysis (Sim-FE)	19 / 18	40 / 38	71.42	46.00	67.78	42.39	62.31	36.70
Primary analysis (Sim-RE)	19 / 18	40 / 38	71.35	44.67	67.19	40.21	61.98	35.64
Baseline-risk adjustment (Sim-FE)	19 / 18	40 / 38	72.50	45.90	68.19	41.85	64.47	38.92
Primary analysis (Ind-FE)	19 / 18	40 / 38	75.67	45.67	71.76	42.60	65.73	37.51
Primary analysis (Ind-RE)	19 / 18	40 / 38	75.57	43.38	71.02	39.66	65.59	36.29
Inclusion of trials with prior bDMARD use of up to 20% (Sim-FE)	29 / 28	61 / 58	137.90	100.65	114.02	78.00	95.31	58.59
Removal of Asian-Pacific trials/low MTX dose (Sim-FE)	15 / 14	32 / 30	60.89	40.54	58.46	37.32	52.45	31.10

Abbreviations: DIC Deviance Information Criterion; $\bar{\mathrm{D}}_{res}$ overall residual deviance; MTX methotrexate; Sim-FE simultaneous fixed-effects model; Sim-RE simultaneous random-effects model; Ind-FE independent fixed-effects model; Ind-RE independent random-effects model.

1- numbers before "/" are for ACR20, numbers after "/" are for ACR50 and ACR70.

12. Posterior Median ACR response, by Treatment and Analysis (Forest Plots)

The following figures present the estimated median ACR response rates, plus corresponding 95% CrIs, across the primary and main sensitivity analyses, separately for each of the treatments.

Figure S4 BARI 4mg + MTX: Estimated posterior median ACR response rates across primary and main sensitivity analyses

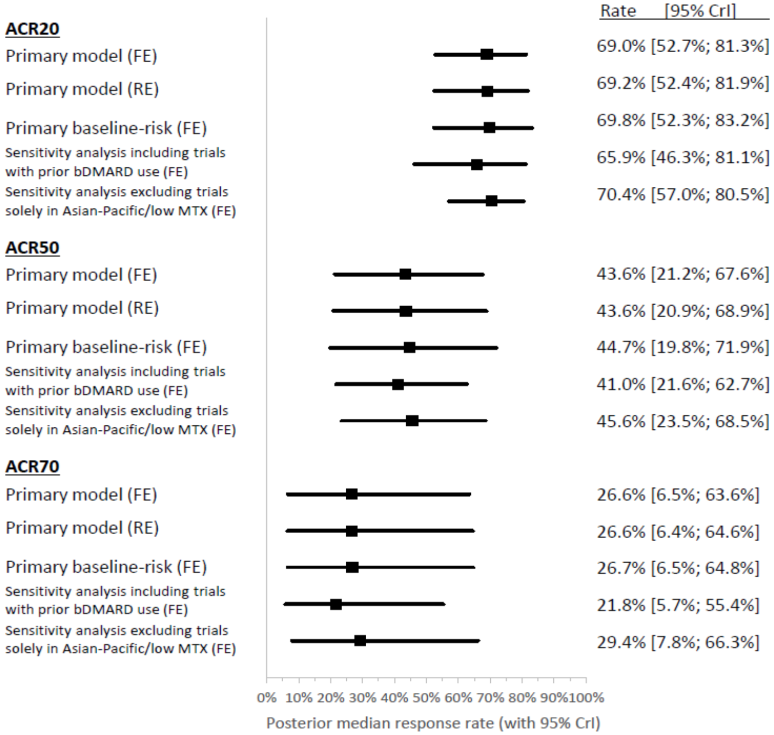

Note: Overall, for baricitinib, there was 1 trial (RA-BEAM). It was part of the primary and all sensitivity analyses.

Figure S5 TOFA 5mg + MTX: Estimated posterior median ACR response rates across primary and main sensitivity analyses

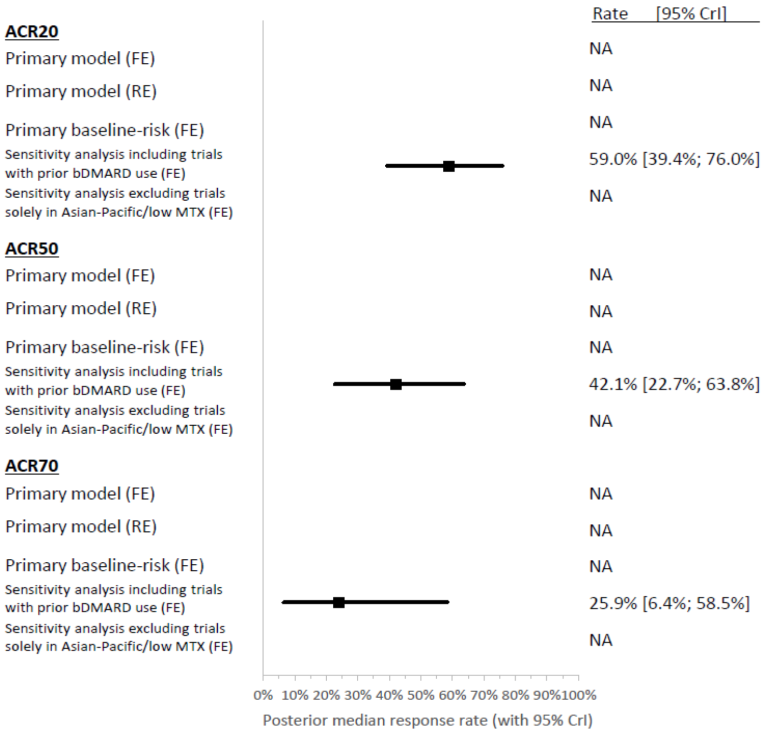

Note: Overall, for tofacitinib there were 3 trials (ORAL SCAN, ORAL STANDARD, ORAL STRATEGY). All allowed for prior bDMARD use of up to 20%, and thus could only be analyzed via the corresponding sensitivity analysis.

Abbreviations: BARI baricitinib; Cr-Int Credible Interval; FE fixed-effects model; MTX methotrexate; RE random-effects model; TOFA tofacitinib.

Figure S6 ADA 40mg + MTX: Estimated posterior median ACR response rates across primary and main sensitivity analyses

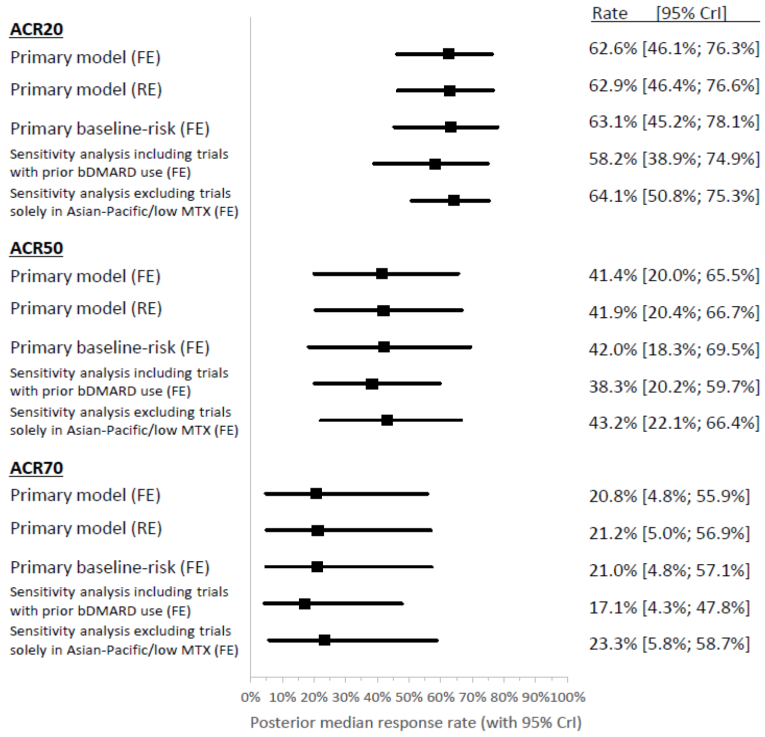

Figure S7 CZP + MTX: Estimated posterior median ACR response rates across primary and main sensitivity analyses

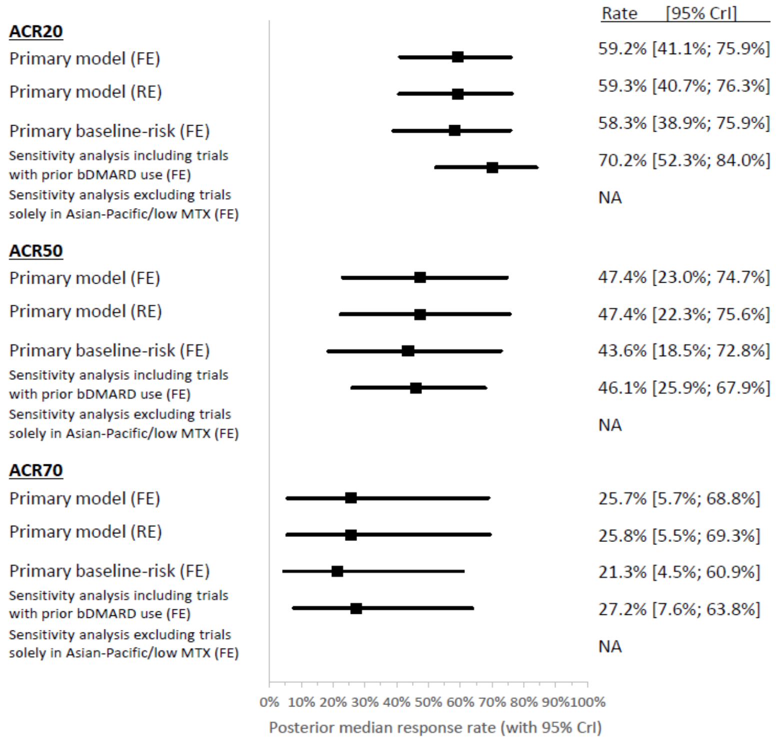

Note: For certolizumab there was only 1 trial in the primary analysis (RAPID-C, Asian-Pacific), and 4 trials (J-RAPID, Kang 2013, RAPID1, RAPID2) that were added via the sensitivity analysis allowing for trials with prior bDMARD use of up to 20% of patients.

Abbreviations: ADA adalimumab; Cr-Int Credible Interval; CZP certolizumab; FE fixed-effects model; MTX methotrexate; RE random-effects model.

Figure S8 ETN + MTX: Estimated posterior median ACR response rates across primary and main sensitivity analyses

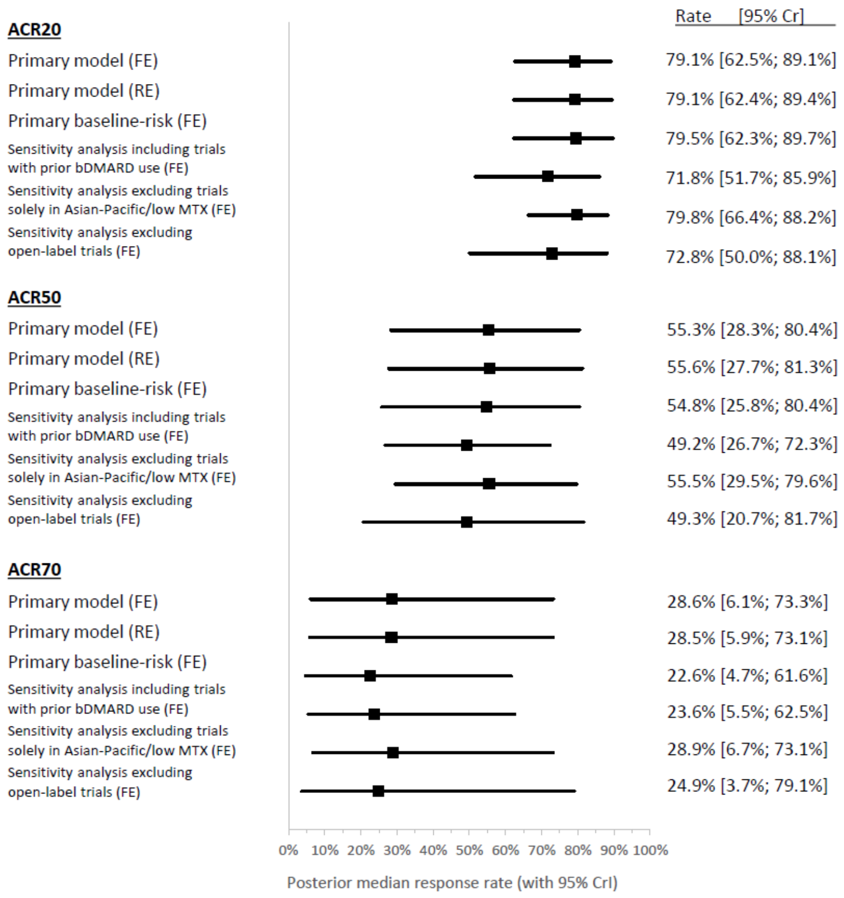

Note: The primary analysis included 2 trials (Machado 2014, Weinblatt 1999), one of which was an open-label trial (Machado 2014) not directly connected to the reference treatment (PBO+MTX). Overall, there were 3 ETN trials, and 2 of them compared ETN+MTX against cDMARD+MTX.

Figure S9 GOL 50mg + MTX: Estimated posterior median ACR response rates across primary and main sensitivity analyses

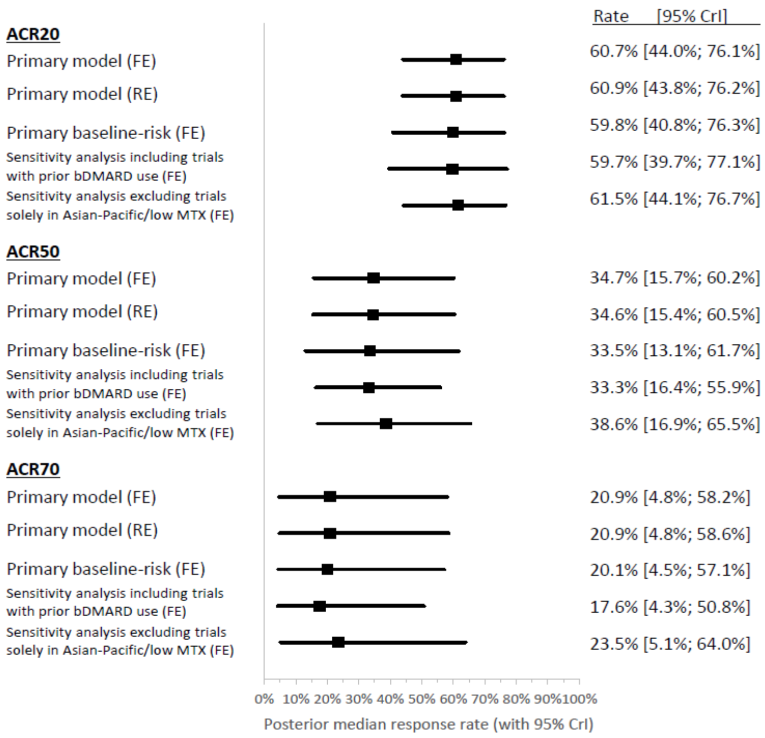

Note: The primary analysis included only 1 trial (GO-FORWARD). Two additional trials (GO-FORTH, Li 2013) were added via the sensitivity analysis allowing for prior bDMARD use of up to 20%.

Abbreviations: Cr-Int Credible Interval; ETN eternacept; FE fixed-effects model; GOL golimumab; MTX methotrexate; RE random-effects model.

Figure S10 IFX 3mg + MTX: Estimated posterior median ACR response rates across primary and main sensitivity analyses

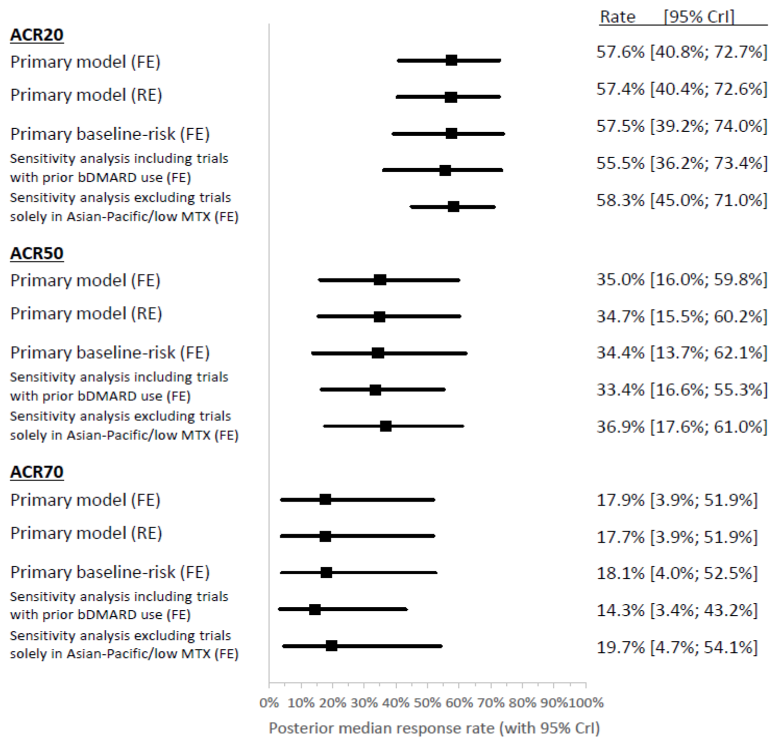

Note: Overall, for infliximab, there were 2 trials (ATTRACT, START), which were part of the primary and all sensitivity analyses. In ATTRACT, only ACR20 was reported.

Figure S11 ABA 10mg + MTX: Estimated posterior median ACR response rates across primary and main sensitivity analyses

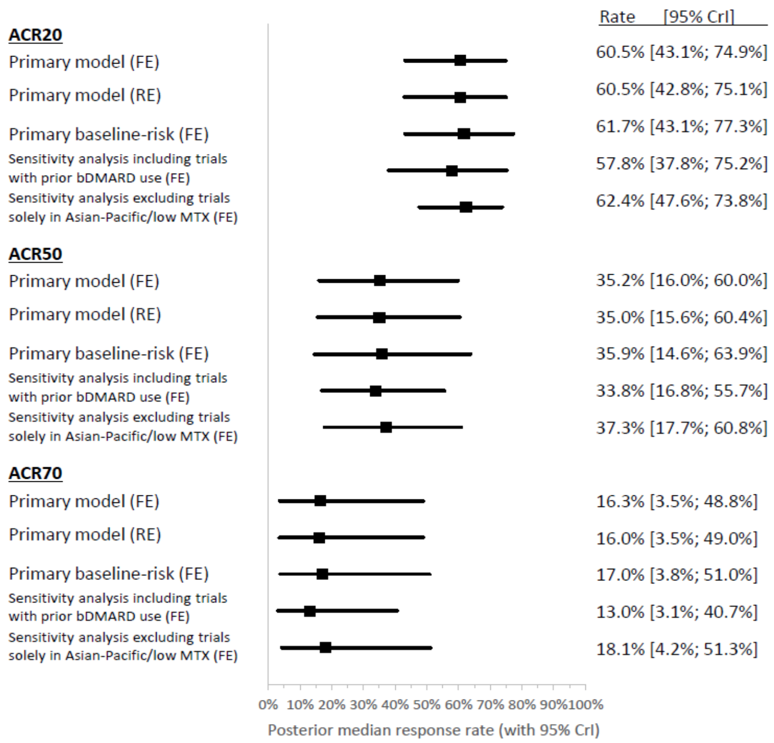

Note: Overall, for abatacept 10mg, there were 2 trials (AIM, ATTEST), which were part of the primary and all sensitivity analyses.

Abbreviations: ABA abatacept; Cr-Int Credible Interval; FE fixed-effects model; IFX infliximab; MTX methotrexate; RE random-effects model.

Figure S12 ABA SUBCUT + MTX: Estimated posterior median ACR response rates across primary and main sensitivity analyses

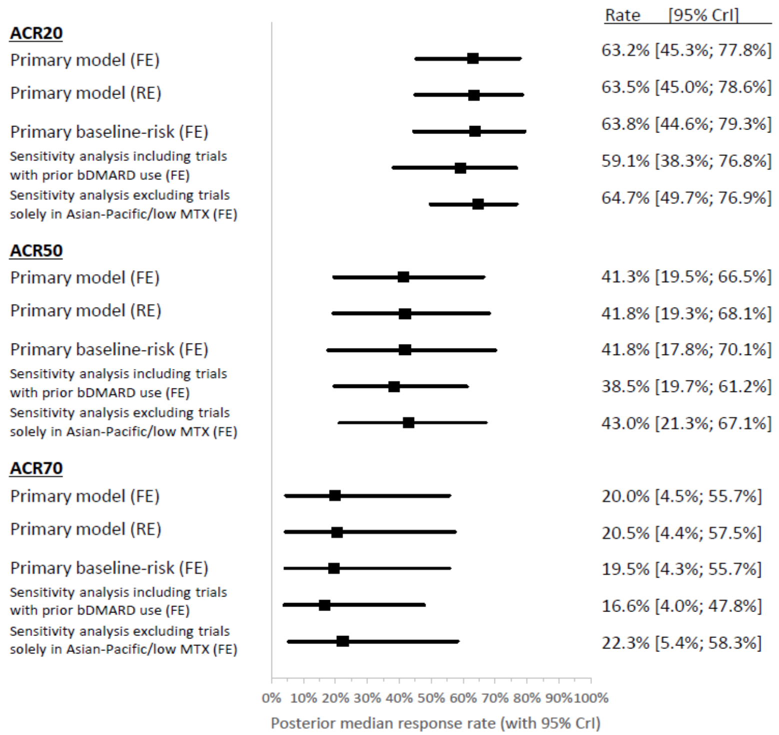

Figure S13 RTX + MTX: Estimated posterior median ACR response rates across primary and main sensitivity analyses

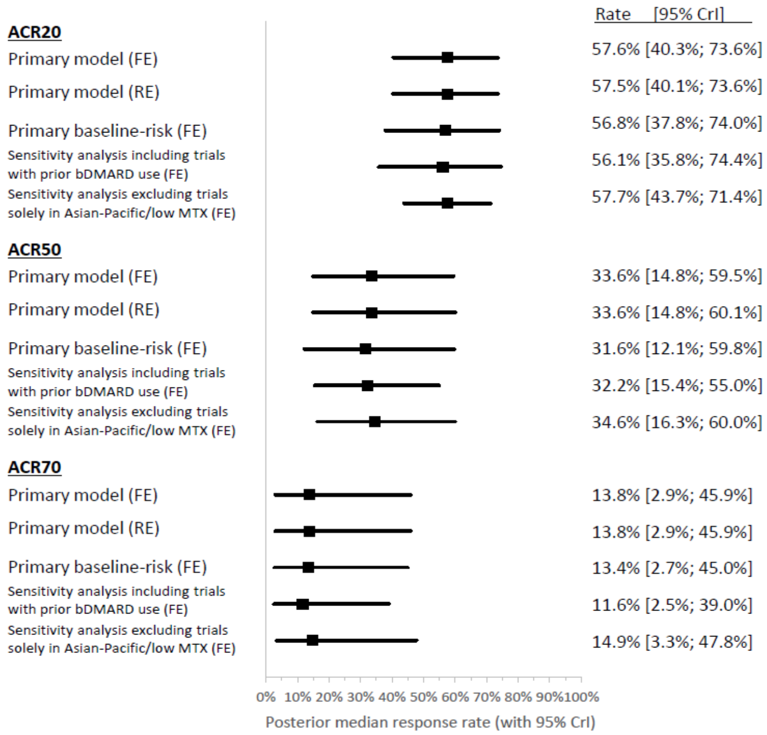

Note: Overall, for abatacept subcutaneous, there was 1 trial (AMPLE). It compared ADA SUBCUT + MTX vs ADA 40mg + MTX and was part of the primary and all sensitivity analyses.

Abbreviations: ABA abatacept; Cr-Int Credible Interval; FE fixed-effects model; MTX methotrexate; RE random-effects model; RTX rituximab; SUBCUT subcutaneous.

Figure S14 TCZ + MTX: Estimated posterior median ACR response rates across primary and main sensitivity analyses

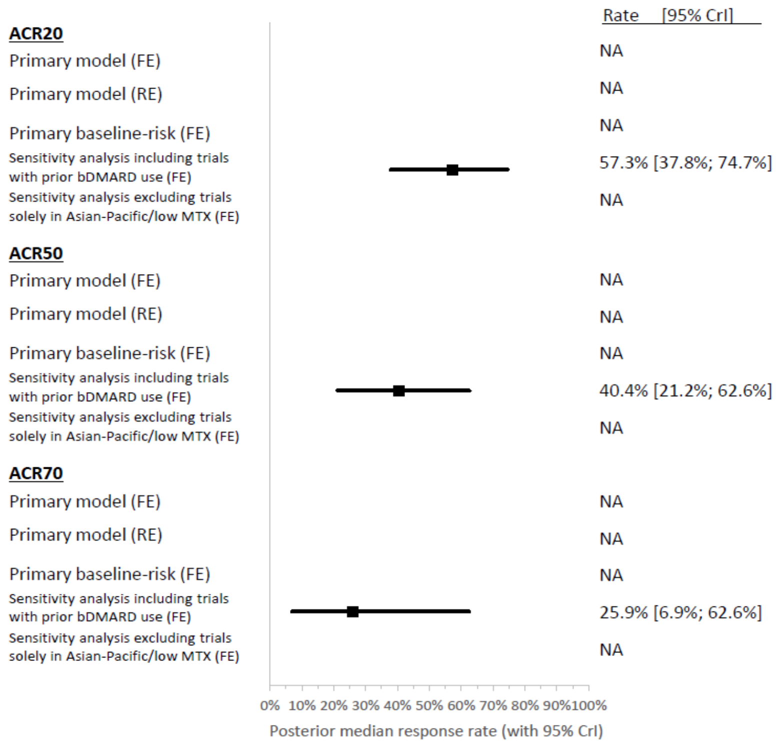

Note: Overall, for tocilizumab there were only 2 trials (LITHE, OPTION). Both allowed for prior bDMARD use of up to 20%, and thus could only be analyzed via the corresponding sensitivity analysis.

Figure S15 SARI 200mg + MTX: Estimated posterior median ACR response rates across primary and main sensitivity analyses

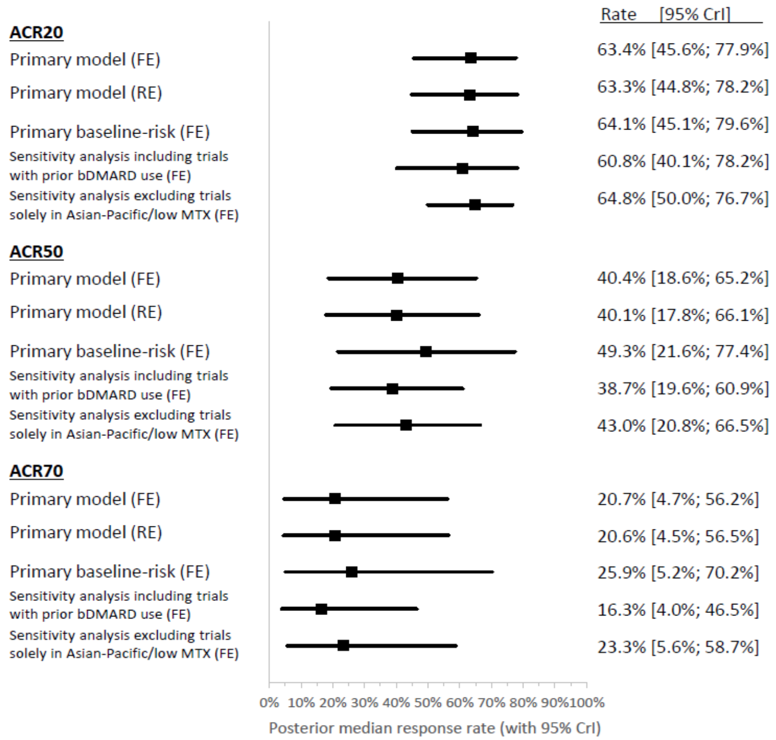

Note: Overall, for sarilumab, there was 1 trial (MOBILITY). Only results from the subpopulation of MOBILITY patients who were bDMARD-naïve were used in all analyses (primary and all sensitivity).

Abbreviations: Cr-Int Credible Interval; FE fixed-effects model; MTX methotrexate; RE random-effects model; SARI sarilumab; TCZ tocilizumab.

Figure S16 PBO + MTX: Estimated posterior median ACR response rates across primary and main sensitivity analyses

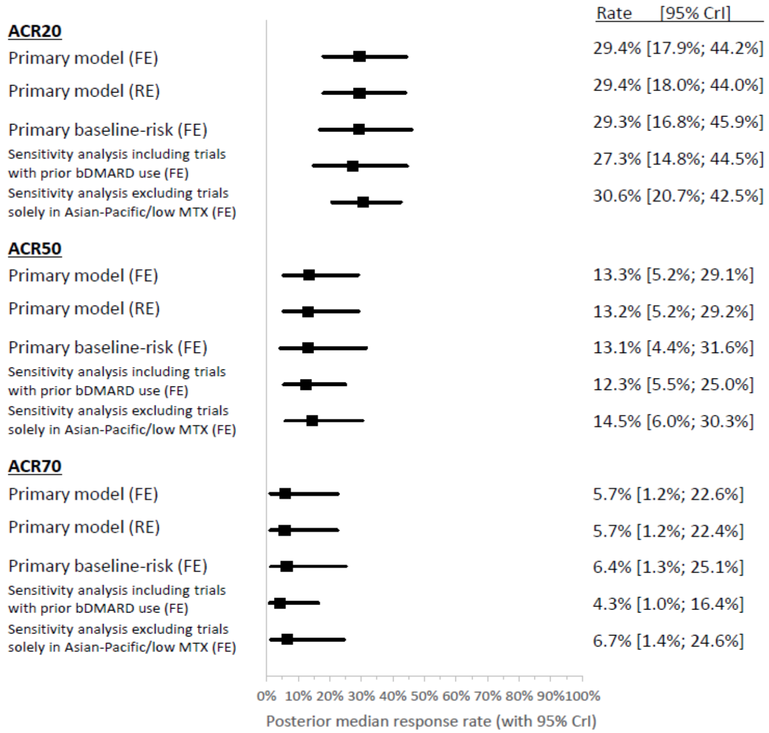

Abbreviations: Cr-Int Credible Interval; FE fixed-effects model; PBO placebo; MTX methotrexate; RE random-effects model.

13. References

1. Kremer JM, Genant HK, Moreland LW, et al. Effects of abatacept in patients with methotrexate-resistant active rheumatoid arthritis: a randomized trial. Ann Intern Med. 2006;144(12):865-876.

2. Schiff M, Weinblatt ME, Valente R, et al. Head-to-head comparison of subcutaneous abatacept versus adalimumab for rheumatoid arthritis: two-year efficacy and safety findings from AMPLE trial. Ann Rheum Dis. 2014;73(1):86-94.

3. Weinblatt ME, Keystone EC, Furst DE, et al. Adalimumab, a fully human anti-tumor necrosis factor alpha monoclonal antibody, for the treatment of rheumatoid arthritis in patients taking concomitant methotrexate: the ARMADA trial. Arthritis Rheum. 2003;48(1):35-45.

4. Schiff M, Keiserman M, Codding C, et al. Efficacy and safety of abatacept or infliximab vs placebo in ATTEST: a phase III, multi-centre, randomised, double-blind, placebo-controlled study in patients with rheumatoid arthritis and an inadequate response to methotrexate. Ann Rheum Dis. 2008;67(8):1096-1103.

5. Maini R, St Clair EW, Breedveld F, et al. Infliximab (chimeric anti-tumour necrosis factor alpha monoclonal antibody) versus placebo in rheumatoid arthritis patients receiving concomitant methotrexate: a randomised phase III trial. ATTRACT Study Group. Lancet. 1999;354(9194):1932-1939.

6. Edwards JC, Szczepanski L, Szechinski J, et al. Efficacy of B-cell-targeted therapy with rituximab in patients with rheumatoid arthritis. N Engl J Med. 2004;350(25):2572-2581.

7. Tanaka Y, Harigai M, Takeuchi T, et al. Golimumab in combination with methotrexate in Japanese patients with active rheumatoid arthritis: results of the GO-FORTH study. Ann Rheum Dis. 2012;71(6):817-824.

8. Keystone EC, Genovese MC, Klareskog L, et al. Golimumab, a human antibody to tumour necrosis factor {alpha} given by monthly subcutaneous injections, in active rheumatoid arthritis despite methotrexate therapy: the GO-FORWARD Study. Ann Rheum Dis. 2009;68(6):789-796.

9. Keystone EC, Kavanaugh AF, Sharp JT, et al. Radiographic, clinical, and functional outcomes of treatment with adalimumab (a human anti-tumor necrosis factor monoclonal antibody) in patients with active rheumatoid arthritis receiving concomitant methotrexate therapy: a randomized, placebo-controlled, 52-week trial. Arthritis Rheum. 2004;50(5):1400-1411.

10. Kim HY LS, Song YW, Yoo DH, Koh EM, Yoo B. A randomized, double-blind, placebo-controlled, phase III study of the human anti-tumor necrosis factor antibody adalimumab administered as subcutaneous injections in Korean rheumatoid arthritis patients treated with methotrexate. APLAR Journal of Rheumatology 2007;10(1):9-16. 2007.

11. Li Z ZF, Kay J, Fei K, Han C, Zhuang Y. Safety and efficacy of subcutaneous golimumab in Chinese patients with active rheumatoid arthritis despite MTX therapy: Results from a randomized, placebo-controlled, phase 3 trial. Arthritis and Rheumatism. Arthritis and Rheumatism 2013;65(Suppl 10):S598-S9. 2013.

12. Machado DA, Guzman RM, Xavier RM, et al. Open-label observation of addition of etanercept versus a conventional disease-modifying antirheumatic drug in subjects with

active rheumatoid arthritis despite methotrexate therapy in the Latin American region. J Clin Rheumatol. 2014;20(1):25-33.

13. Genovese MC, Fleischmann R, Kivitz AJ, et al. Sarilumab Plus Methotrexate in Patients With Active Rheumatoid Arthritis and Inadequate Response to Methotrexate: Results of a Phase III Study. Arthritis Rheumatol. 2015;67(6):1424-1437.

14. Peterfy C, Emery P, Tak PP, et al. MRI assessment of suppression of structural damage in patients with rheumatoid arthritis receiving rituximab: results from the randomised, placebo-controlled, double-blind RA-SCORE study. Ann Rheum Dis. 2016;75(1):170-177.

15. Bi L, Li Y, He L, et al. Efficacy and safety of certolizumab pegol in combination with methotrexate in methotrexate-inadequate responder Chinese patients with active rheumatoid arthritis: 24-week results from a randomised, double-blind, placebo-controlled phase 3 study. Clin Exp Rheumatol. 2018.

16. Emery P, Deodhar A, Rigby WF, et al. Efficacy and safety of different doses and retreatment of rituximab: a randomised, placebo-controlled trial in patients who are biological naive with active rheumatoid arthritis and an inadequate response to methotrexate (Study Evaluating Rituximab's Efficacy in MTX iNadequate rEsponders (SERENE)). Ann Rheum Dis. 2010;69(9):1629-1635.

17. Westhovens R, Cole JC, Li T, et al. Improved health-related quality of life for rheumatoid arthritis patients treated with abatacept who have inadequate response to anti-TNF therapy in a double-blind, placebo-controlled, multicentre randomized clinical trial. Rheumatology (Oxford). 2006;45(10):1238-1246.

18. Weinblatt ME, Kremer JM, Bankhurst AD, et al. A trial of etanercept, a recombinant tumor necrosis factor receptor:Fc fusion protein, in patients with rheumatoid arthritis receiving methotrexate. N Engl J Med. 1999;340(4):253-259.

19. Taylor PC KE, Van Der Heijde D, Weinblatt ME, Del Carmen Morales L, Reyes Gonzaga J. Baricitinib versus placebo or adalimumab in rheumatoid arthritis. . N Engl J Med 2017 Feb 16;376(7):652-62. 2017.

20. Yamamoto K, Takeuchi T, Yamanaka H, et al. Efficacy and safety of certolizumab pegol plus methotrexate in Japanese rheumatoid arthritis patients with an inadequate response to methotrexate: the J-RAPID randomized, placebo-controlled trial. Mod Rheumatol. 2014;24(5):715-724.

21. Kang YM PW, Park YE, Choe JY, Bae SC, Cho CS. Efficacy and safety of certolizumab pegol (CZP) with concomitant methotrexate (MTX) in korean rheumatoid arthritis (RA) patients (PTS) with an inadequate response to MTX. Annals of the Rheumatic Disease 2013;71(Suppl 3):666.

22. Kremer JM, Blanco R, Brzosko M, et al. Tocilizumab inhibits structural joint damage in rheumatoid arthritis patients with inadequate responses to methotrexate: results from the double-blind treatment phase of a randomized placebo-controlled trial of tocilizumab safety and prevention of structural joint damage at one year. Arthritis Rheum. 2011;63(3):609-621.

23. Smolen JS, Beaulieu A, Rubbert-Roth A, et al. Effect of interleukin-6 receptor inhibition with tocilizumab in patients with rheumatoid arthritis (OPTION study): a double-blind, placebo-controlled, randomised trial. Lancet. 2008;371(9617):987-997.

24. van der Heijde D, Tanaka Y, Fleischmann R, et al. Tofacitinib (CP-690,550) in patients with rheumatoid arthritis receiving methotrexate: twelve-month data from a twenty-four-month phase III randomized radiographic study. Arthritis Rheum. 2013;65(3):559-570.

25. van Vollenhoven RF, Fleischmann R, Cohen S, et al. Tofacitinib or adalimumab versus placebo in rheumatoid arthritis. N Engl J Med. 2012;367(6):508-519.

26. Fleischmann R, Mysler E, Hall S, et al. Efficacy and safety of tofacitinib monotherapy, tofacitinib with methotrexate, and adalimumab with methotrexate in patients with rheumatoid arthritis (ORAL Strategy): a phase 3b/4, double-blind, head-to-head, randomised controlled trial. Lancet. 2017;390(10093):457-468.

27. O'Dell JR, Mikuls TR, Taylor TH, et al. Therapies for active rheumatoid arthritis after methotrexate failure. N Engl J Med. 2013;369(4):307-318.

28. Keystone E, Burmester GR, Furie R, et al. Improvement in patient-reported outcomes in a rituximab trial in patients with severe rheumatoid arthritis refractory to anti-tumor necrosis factor therapy. Arthritis Rheum. 2008;59(6):785-793.

29. Smolen J, Landewe RB, Mease P, et al. Efficacy and safety of certolizumab pegol plus methotrexate in active rheumatoid arthritis: the RAPID 2 study. A randomised controlled trial. Ann Rheum Dis. 2009;68(6):797-804.

30. Felson DT, Anderson JJ, Boers M, et al. American College of Rheumatology. Preliminary definition of improvement in rheumatoid arthritis. Arthritis Rheum. 1995;38(6):727-735.

31. NICE-DSU. (2012). NICE Decision Unit: evidence synthesis technical support documents serie. http://nicedsu.org.uk/technical-support-documents/. .

32. Dias, S., Welton, N.J., Sutton, A.J., Ades, A.E. NICE DSU Technical Support Document 5: Evidence synthesis in the baseline natural history model. 2011c; last updated April 2012; available from http://www.nicedsu.org.uk.

33. Rücker, G. Network meta-analysis, electrical networks and graph theory. Res Synth Methods. 2012;3(4):312-324.

34. Higgins JP, Whitehead A. Borrowing strength from external trials in a meta-analysis.Stat Med. 1996;15(24): 2733-2749.

35. Lu G, Ades AE. Combination of direct and indirect evidence in mixed treatment comparisons. Stat Med. 2004;23(20): 3105-3124.

36. Jansen JP, Crawford B, Bergman G, Stam W. Bayesian meta-analysis of multiple treatment comparisons: an introduction to mixed treatment comparisons. Value Health. 2008;11(5):956-964.

37. Jansen, J. P., Crawford, B., Bergman, G., & Stam, W. (2008). Bayesian meta-analysis of multiple treatment comparisons: an introduction to mixed treatment comparisons. Value Health, 11(5), 956-964. doi: 10.1111/j.1524-4733.2008.00347.

38. Dias, S., Sutton, A.J., Welton, N.J., Ades, A.E. NICE DSU Technical Support Document 3: Heterogeneity: subgroups, meta-regression, bias and bias-adjustment. 2011a; last updated April 2012; available from http://www.nicedsu.org.uk.

39. Dias, S., Welton, N.J., Sutton, A.J. & Ades, A.E. NICE DSU Technical Support Document 2: A Generalised Linear Modelling Framework for Pairwise and Network Meta-Analysis of Randomised Controlled Trials. 2011b; last updated September 2016; available from http://www.nicedsu.org.uk.

40. Jonas DE, Wilkins TM, Bangdiwala S, Bann CM, Morgan LC, Thaler KJ, Amick HR, Gartlehner G. Findings of bayesian mixed treatment comparison meta-analysis: Comparison and exploration using real-world trial data and simulation. Report No.: 13-EHC039-EF. Rockville (MD): Agency for Healthcare Research and Quality; 2013. Available at: http://www.ncbi.nlm.nih.gov/books/NBK126109/.

41. Turner, R. M., Jackson D., Wei Y., Thompson S. G., & Higgins J. P. T. (2014). Predictive distributions for between-study heterogeneity and simple methods for their application in Bayesian meta-analysis. Statist. Med. 34, 984–998. doi: 10.1002/sim.6381.

42. R Core Team (2018). R: A language and environment for statistical computing. R Foundation for Statistical Computing, Vienna, Austria. URL https://www.R-project.org/.

43. Gerta Rücker, Ulrike Krahn, Jochem König, Orestis Efthimiou and Guido Schwarzer (2019). netmeta: Network Meta-Analysis using Frequentist Methods. R package version 1.0-1. https://CRAN.R-project.org/package=netmeta.

44. Sweeting, M. J., Sutton, A. J., & Lambert, P. C. (2004). What to add to nothing? Use and avoidance of continuity corrections in meta-analysis of sparse data. Stat Med, 23(9), 1351-1375. doi: 10.1002/sim.1761.

45. Bradburn MJ, Deeks JJ, Berlin JA, Russell Localio A. Much ado about nothing: a comparison of the performance of meta-analytical methods with rare events. Stat Med. 2007;26(1): 53-77.

46. Dias, S., Welton, N. J., Sutton, A. J., & Ades, A. E. (2013). Evidence synthesis for decision making 5: the baseline natural history model. Med Decis Making, 33(5), 657-670. doi: 10.1177/0272989X13485155.

47. Spiegelhalter DJ, Best NG, Carlin BP, van der Linde A. Bayesian measures of model complexity and fit. J Royal Statistical Society. 2002:64(4):583-639.

48. Carlin BP, Louis TA. Bayesian methods for data analysis. 3rd ed. Boca Raton: Chapman & Hall; 2008.

49. Gelman A, Rubin DB. Inference from iterative simulation using multiple sequences. Statistical Science. 1992;7(4):457-511.

50. Achana FA, Cooper NJ, Dias S, Lu G, Rice SJC, Kendrick D and Sutton AJ. Extending methods for investigating the relationship between treatment effect and baseline risk from pairwise meta-analysis to network meta-analysis. Statist. Med. 2013; 32: 752–771.
